# High dimensional predictions of suicide risk in 4.2 million US Veterans using ensemble transfer learning

**DOI:** 10.1038/s41598-024-51762-9

**Published:** 2024-01-20

**Authors:** Sayera Dhaubhadel, Kumkum Ganguly, Ruy M. Ribeiro, Judith D. Cohn, James M. Hyman, Nicolas W. Hengartner, Beauty Kolade, Anna Singley, Tanmoy Bhattacharya, Patrick Finley, Drew Levin, Haedi Thelen, Kelly Cho, Lauren Costa, Yuk-Lam Ho, Amy C. Justice, John Pestian, Daniel Santel, Rafael Zamora-Resendiz, Silvia Crivelli, Suzanne Tamang, Susana Martins, Jodie Trafton, David W. Oslin, Jean C. Beckham, Nathan A. Kimbrel, Million Veteran Program Suicide Exemplar Work Group, Khushbu Agarwal, Allison E. Ashley-Koch, Mihaela Aslan, Edmond Begoli, Ben Brown, Patrick S. Calhoun, Kei-Hoi Cheung, Sutanay Choudhury, Ashley M. Cliff, Leticia Cuellar-Hengartner, Haedi E. Deangelis, Michelle F. Dennis, Patrick D. Finley, Michael R. Garvin, Joel E. Gelernter, Lauren P. Hair, Colby Ham, Phillip D. Harvey, Elizabeth R. Hauser, Michael A. Hauser, Nick W. Hengartner, Daniel A. Jacobson, Jessica Jones, Piet C. Jones, David Kainer, Alan D. Kaplan, Ira R. Katz, Rachel L. Kember, Angela C. Kirby, John C. Ko, John Lagergren, Matthew Lane, Daniel F. Levey, Jennifer H. Lindquist, Xianlian Liu, Ravi K. Madduri, Carrie Manore, Carianne Martinez, John F. McCarthy, Mikaela McDevitt Cashman, J. Izaak Miller, Destinee Morrow, Mirko Pavicic-Venegas, Saiju Pyarajan, Xue J. Qin, Nallakkandi Rajeevan, Christine M. Ramsey, Ruy Ribeiro, Alex Rodriguez, Jonathon Romero, Yunling Shi, Murray B. Stein, Kyle A. Sullivan, Ning Sun, Suzanne R. Tamang, Alice Townsend, Jodie A. Trafton, Angelica Walker, Xiange Wang, Victoria Wangia-Anderson, Renji Yang, Shinjae Yoo, Hongyu Zhao, Benjamin H. McMahon

**Affiliations:** 1 Los Alamos National Laboratory, Los Alamos, NM 87545 USA; 2 Sandia National Laboratory, Albuquerque, NM 87123 USA; 3 Massachusetts Veterans Epidemiology and Research Information Center (MAVERIC), VA Boston Healthcare System, Boston, USA; 4 Department of Medicine, Brigham and Women’s Hospital and Harvard Medical School, Boston, USA; 5 VA Connecticut Healthcare System, Yale Schools of Medicine and Public Health, Yale University, West Haven, CT USA; 6 Cincinnati Children’s Hospital Medical Center, Cincinnati, Ohio USA; 7 Applied Mathematics and Computational Research Division, Lawrence Berkeley National Laboratory, 1 Cyclotron Rd, Berkeley, CA 94720 USA; 8 Program Evaluation and Resource Center, Office of Mental Health and Suicide Prevention, Veterans Affairs Palo Alto Health Care System, Menlo Park, CA USA; 9 Department of Medicine, Stanford University, Stanford, California USA; 10 Cpl Michael J Crescenz VA Medical Center, VISN 4 Mental Illness Research, Education, and Clinical Center; Department of Psychiatry, Perelman School of Medicine, University of Pennsylvania, 3535 Market Street, Philadelphia, PA 19104 USA; 11 Durham Veterans Affairs (VA) Health Care System, Durham, NC USA; 12 VA Mid-Atlantic Mental Illness Research, Education and Clinical Center, Durham, NC USA; 13 VA Health Services Research and Development Center of Innovation to Accelerate Discovery and Practice Transformation, Durham, NC USA; 14 Department of Psychiatry and Behavioral Sciences, Duke University School of Medicine, Durham, NC USA; 15 Pacific Northwest National Laboratory, Richland, WA 99354 USA; 16 VA Connecticut Healthcare System, West Haven, CT 06516 USA; 17 Oak Ridge National Laboratory, Oak Ridge, TN 37870 USA; 18 Miami VA Medical Center, Miami, FA 33125 USA; 19 Lawrence Livermore National Laboratory, Livermore, CA 94550 USA; 20 Argonne National Laboratory, Lemont, IL 60439 USA; 21 University of California at San Diego, La Jolla, CA 92093 USA; 22 Brookhaven National Laboratory, Upton, NY 11973 USA; 23 Office of Mental Health and Suicide Prevention, Department of Veterans Affairs, Washington, DC, USA; 24 Department of Psychiatry, Veterans Affairs Connecticut Healthcare System, West Haven, CT, USA

**Keywords:** Machine learning, Outcomes research

## Abstract

We present an ensemble transfer learning method to predict suicide from Veterans Affairs (VA) electronic medical records (EMR). A diverse set of base models was trained to predict a binary outcome constructed from reported suicide, suicide attempt, and overdose diagnoses with varying choices of study design and prediction methodology. Each model used twenty cross-sectional and 190 longitudinal variables observed in eight time intervals covering 7.5 years prior to the time of prediction. Ensembles of seven base models were created and fine-tuned with ten variables expected to change with study design and outcome definition in order to predict suicide and combined outcome in a prospective cohort. The ensemble models achieved c-statistics of 0.73 on 2-year suicide risk and 0.83 on the combined outcome when predicting on a prospective cohort of $\sim$ 4.2 M veterans. The ensembles rely on nonlinear base models trained using a matched retrospective nested case-control (Rcc) study cohort and show good calibration across a diversity of subgroups, including risk strata, age, sex, race, and level of healthcare utilization. In addition, a linear Rcc base model provided a rich set of biological predictors, including indicators of suicide, substance use disorder, mental health diagnoses and treatments, hypoxia and vascular damage, and demographics.

## Introduction

Predicting suicide risk is complicated by the diverse pathways leading to this outcome^1–7^, the desire for acute risk prediction^8,9^, the lack of available biomarkers^10^, and the frequent ambiguity surrounding the cause of death^11–15^. The relatively low absolute incidence of death by suicide in the US population^16^, approximately 14.0/100,000 population per year or 1–2% of all US deaths^17^, amplifies these difficulties. Even with a favorable trade-off between sensitivity and specificity, the majority of patients identified as high-risk do not go on to die by suicide. These issues lead to models with poor predictive value and thus can create a significant, potentially unsustainable, clinical burden. We hope to enable quantitative analysis of strategies to reach deeper into the risk pool by exploring the use of combined outcomes and facilitating the sub-grouping of patients for whom interventions could be more-specifically targeted^3^. This requires a well-calibrated model characterized by a wide variety of variables indicating mechanisms.

ReachVet^18,19^ and STORM^20,21^ models developed by the Veterans Affairs Office of Mental Health and Suicide Prevention (VA/OMHSP) target limited clinical resources for suicide prevention and reducing overdose death, respectively. ReachVet is based on the extensive EMRs available in the VA, and was designed to intervene specifically for veterans predicted to be in the top 0.1% of risk for suicide at each of the 150 VA medical centers. A recent program evaluation found this intervention to be associated with greater treatment engagement and fewer mental health admissions, emergency department visits, and suicide attempts, but not with a reduction in suicides or all-cause mortality^22^. In this work, we build from these pioneering studies, which provide both a rich set of hand-curated variables and an overall workflow developed through years of operational use^22^. We expanded the time dependence of our predictor variables and introduced new input variables for vital signs and common laboratory metrics^23^ as well as census data from the American Community Survey^24^ matched to patients by zip code. To make more effective use of the limited number of cases available to train our statistical models, we explored a variety of study designs and predictive modeling methodologies.

Several strategies aid the evaluation of complex medical outcomes, including the use of multiple related outcomes^25^, longitudinal analysis^26^, and case–control study designs^27^ with matching^28^. We especially utilized Ernster’s description^29^ of the numerous advantages of retrospective nested case–control (Rcc) and case-cohort studies for identifying biological precursors of disease and ascertaining the incidence of disease and its risk factors prospectively in a population, respectively, and Justice et al.’s discussion^30^ of how to define, incorporate, and assess generalizability for predictive models of medical outcomes. The ambiguity surrounding suicide and its association with suicide attempts and overdoses suggests using a combined outcome, although one must be mindful of how this technique has been misapplied in the past^31^.

Transfer learning^32–34^ is a flexible machine learning approach that involves training a predictive model on a prevalent outcome, then tailoring (or fine-tuning) it to a more specific problem of interest with less abundant examples. Ensemble modeling^35,36^ is a complementary technique that creates a robust model by combining a diverse set of base models created with appropriate types of diversity. These techniques have recently been combined^37^ to predict a variety of healthcare outcomes^35^, breast cancer histopathology^38^, COVID-19 from chest CT scans^39^, and anti-cancer drug responses^40^.

In this work we guide our development of an ensemble transfer learning model to characterize the risk factors of suicide related behaviors and to accurately predict suicide in a prospective cohort of patients.

## Results

We first define our two types of study design, retrospective (Rcc) and prospective (C15, C17, CoxMH, and CoxVisit), then compare model coefficients across these cohorts and provide c-statistics for the eight base models and the composition of the ensemble model, which is fine-tuned to predict either suicide or combined outcome on the C17 cohort. We then present the biological drivers of the combined outcome as captured by the coefficients from the Rcc logistic regression base model and calibration plots for important patient subgroups, noting that our ensemble models successfully reproduce both the strong effect of healthcare utilization on the model score for the combined outcome and the much smaller dependence when predicting suicide. We then characterize the dependence of selected variables on age and the confounding effect of the level of healthcare utilization and compare the relative risk for suicide and suicide attempt outcomes of subgroups defined by each predictor variable. Finally, we illustrate how our model performs in identifying high-risk patients for targeted clinical care.

### Base and ensemble models; transfer learning

**Figure 1:**
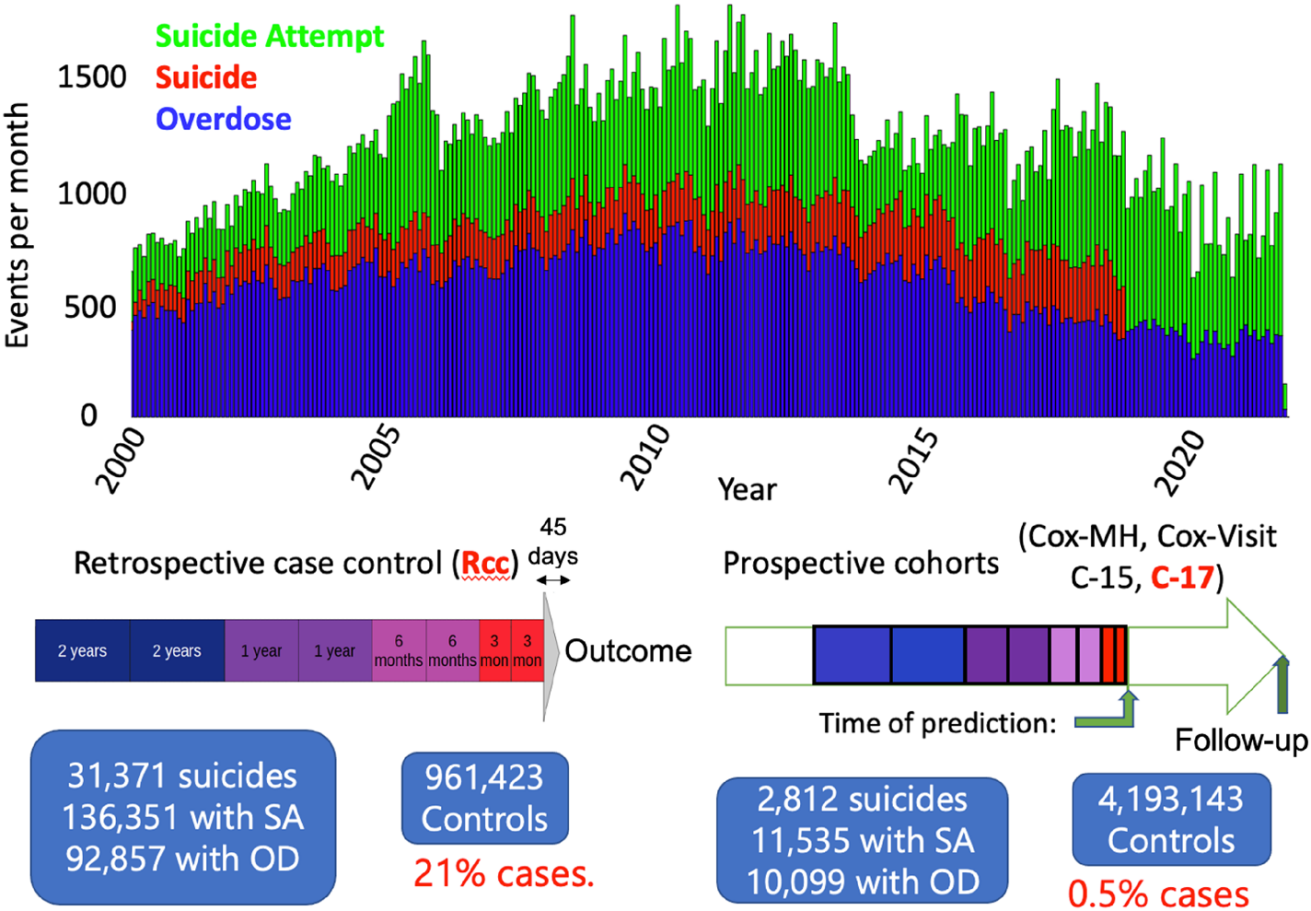
Three important aspects of study design for suicide prediction. At the top is a histogram of the first reported instance of each of the three related outcomes. The lack of deaths by suicide after 2018 is a result of a lag in available data from the National Death Index (NDI)^41^. At the bottom left is a diagram describing our retrospective case–control (Rcc) study design, with eight time windows of increasing width as the time before the outcome increases. At the bottom right is a diagram describing our four prospective cohorts, where our eight-time windows, defined as in Rcc, are aligned with a psychiatric visit (Cox-MH), an office visit (Cox-Visit), January 1, 2015 (C15), or January 1, 2017 (C17). Below the arrow are the numbers of patients with each outcome and the number of controls included in the cohort for Rcc (left) and C17 (right). Further details for all study designs are provided in the Methods section, and demographics are provided in Supplementary Table S2.

Figure 1 shows a histogram of the 45,000 suicides that occurred between 2000 and 2019 in veterans who have EMRs within the VA healthcare system, together with first-recorded instances of suicide attempts and overdoses. Suicides are sourced from National Death Index (NDI)^41^ and first-recorded suicide attempts and overdoses for each patient are sourced from the VA EMRs. Below this histogram are diagrams describing our retrospective nested case-control (Rcc) and prospective cohort study designs. We see that the number of modeled outcomes increases from 2812 suicides and 24,446 combined outcomes in a prospective two-year (C17) cohort to 31,371 suicides and 260,579 combined outcomes in our retrospective case–control (Rcc) study. Because we used the same longitudinal data pattern of eight time bins prior to the time of prediction across all cohorts and both outcomes, we can predict across both models and outcomes. In this way, we aim to combine the mechanistic insights of Rcc with the operational need to compute suicide risk in a prospective cohort.

**Figure 2:**
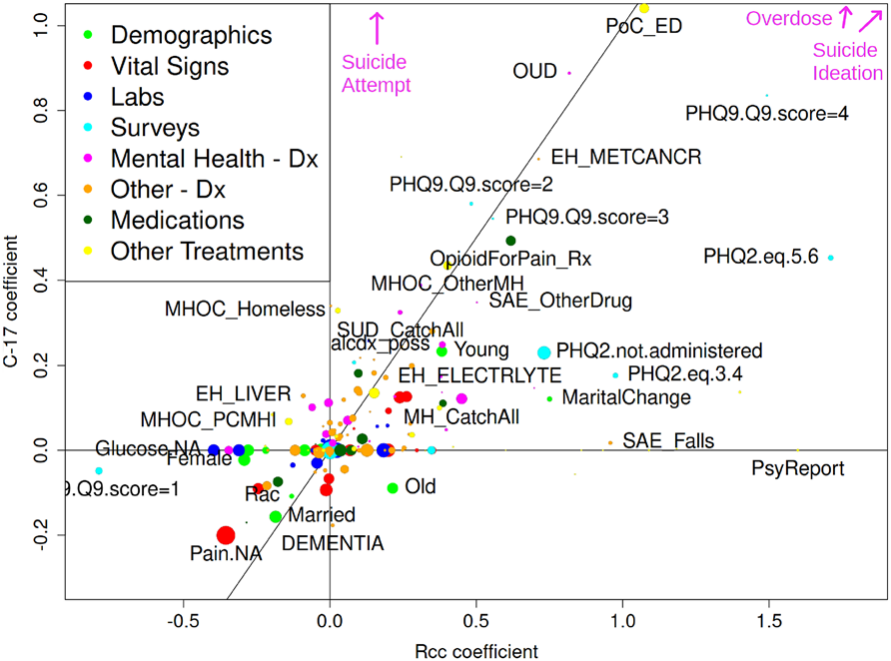
Comparison of model coefficients between Rcc and C17 logistic regression calculations. The x-axis designates the Rcc coefficients for each variable, while C17 coefficients are plotted along the y-axis, in both cases summed across the eight time bins. The area of the symbol is proportional to the number of cases with each variable present in at least one time bin, while the color is coded according to the category of variables, as described in the figure legend. The diagonal line is of slope one and can be used to assess the level of agreement in model coefficients across study designs. Suicide attempt (0.2, 5.1), suicide ideation (3.2, 1.7), and opioid overdose (1.8, 3.8) are indicated with arrows because they are off-scale.

Figure 2 compares the 210 logistic regression coefficients from our Rcc model to those from our C17 model. Many important variables, such as emergency department visit (PoC_ED), opioid use disorder (OUD), metastatic cancer (EH_METCANCR), and being married, have similar coefficients in the two models, and correspond to plausible risk factors. Also evident are large differences for variables expected to be strongly dependent on study design and exclusion criteria, such as suicide attempts, suicide ideation, and overdoses, with the latter provided in Supplementary Table S3. Another reason for the differences in coefficients is the greater statistical power of the Rcc model (260,579 cases in Rcc vs. 22,446 cases in C17, from Fig. 1); indeed, the number of model selected predictor variables for the Rcc cohort was almost double the number for the C17 cohort. These variables appear along the x-axis in Fig. 2, including female sex, psychiatric evaluation, and diagnosis of severe adverse effects from falls.

**Figure 3:**
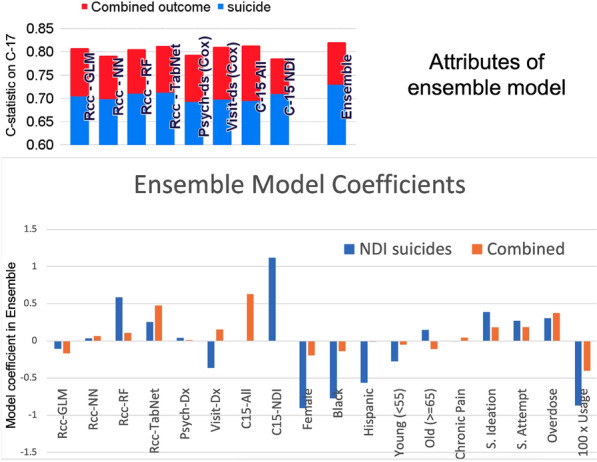
Attributes of ensemble models: (top) C-statistic scores for eight base models and our ensemble models, fine-tuned for both the combined (red bars) and suicide (blue bars) outcomes, all evaluated for the C17 cohort. (bottom) Logistic regression coefficients define the ensemble model component amplitudes and their fine-tuning to predict outcomes for the C17 cohort. The eight left-most coefficients define each base model’s contribution to the ensemble and typically span a range of five units. The ten coefficients to the right define the contributions of the other variables used in the fine-tuning process. Nine of these are binary (0/1) variables, and usage ranges from 0 to 100 and is multiplied by 100 in this plot for ease of comparison. The models were fine-tuned with logistic regression and without cross-validation or model selection. More details of the fine-tuning process are provided in the Methods section.

Comparing c-statistics across cohorts is complicated by differences in the fraction of cases, the case–control matching done in Rcc, and different treatments of overdose and suicide attempt as outcome vs. predictor variables. Because of this, in the top panel of Fig. 3, we compared c-statistic scores on our C17 cohort after performing a transfer learning process where we fine-tuned each of our base models (Rcc, Cox-MH, Cox-Visit, or C15) with a logistic regression optimization including the ten variables named in the bottom panel of Fig. 3. This process was done for both the combined outcome and suicide. C-statistics for the five prediction methodologies ranged from 0.69 to 0.71 for predicting suicide and 0.79–0.81 for predicting the combined outcome.

The situation where a wide variety of distinct models provide similar prediction performance is one in which ensemble models are often most useful^35,36^. Hence, we next created two ensemble models from the eight base models (seven in each ensemble), EnsNDI for suicide (excluding C15 All), and EnsAll for the combined outcome (excluding C15 NDI) and fine-tuned them as described above for scoring the base models. This resulted in a 1 percentage point improvement over any of the base models for both outcomes, as shown in the right-most bar at the top of Fig. 3, and improved model calibration, described below.

Parameters of our fine-tuned ensemble models are provided at the bottom of Fig. 3 with an additional description in the Methods section. The bar graph shows the logistic regression coefficients for each component of our ensemble transfer learning models, EnsNDI and EnsAll. Note that all of the base models were trained on the combined outcome with the exception of C15 NDI, which was trained on suicide as an outcome. We see that approximately half of the weight to the EnsNDI model came from the C15 model, and half from the combinations of non-linear Rcc-trained models. Also evident in this figure, the Cox models did not contribute much in either ensemble model, despite having relatively high c-statistics (see top panel of Fig. 3). The relatively large coefficients for overdose and suicide ideation and attempt are consistent with their strong dependence on study design, as discussed in Fig. 2. It is important to realize that the base model scores have roughly five times the range of the other ten variables when interpreting Fig. 3, because the ensemble model score has a broader range than the binary (0/1) predictor variables.

### Model coefficients

**Figure 4:**
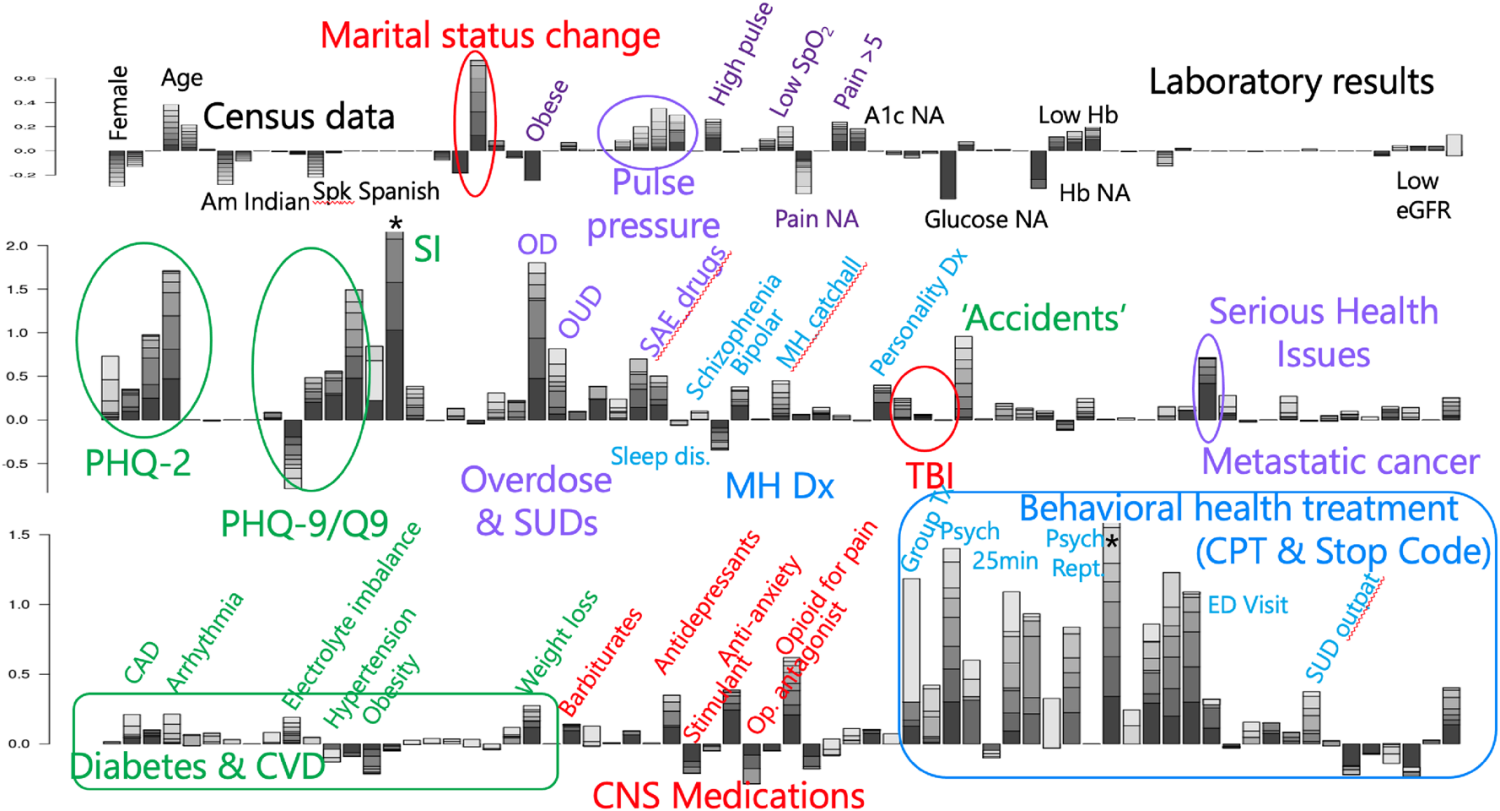
Model coefficients for the Rcc study design and the logistic regression model predicting our combined outcome. Stacked bar charts indicate model coefficients, with darker shades indicating acute predictors (proximal to the event in Fig. 1), and lighter colors from time bins 3–7 years earlier. Upward direction indicates variables that are predictive of outcome events. The * above suicide ideation and psychiatric report indicate off-scale values of 3.2 and 1.6, respectively. Values of coefficients, prevalence, and other attributes are provided in Supplementary Table S3 for selected predictors. Individual labels are omitted from the figure, but the names of all coefficients can be seen together with prevalence information in the bar charts in Supplementary Figures S1–S8. The demographics and census data were split into eight even compartments, as their value does not change across the time bins.

The VA EMR dataset boasts sufficient statistical power for LASSO model selection^42^ of hundreds of time-dependent predictor variables. Figure 4 provides an overview of these results from our Rcc study design as a stacked bar chart. We see a wide variety of variables that are associated with our combined outcome, including demographics, vital signs, laboratory results, PHQ2 and PHQ9 mental health survey results^43^, opioid use, mental health diagnoses, serious health conditions, certain aspects of cardiovascular disease, and behavioral health treatments. We provide more detailed definitions of the variable definitions in the Methods section, and compare them to existing literature in a Supplementary Discussion section, providing here several illustrative results.

The behavioral health treatment variables have many of the largest model coefficients. With the exception of ED visits, however, the variables with large coefficients are only present in a small fraction of patients, typically less than 1%, as can be seen in the bottom panels of Supplementary Figures S1–S8. The results of PHQ-2 and PHQ-9 surveys, circled in green in Fig. 4, show positive associations with increasing scores for both. For each survey, the ’NA’ category, indicating the survey was not given during the 7.5-year prediction window, is shown first, followed by categories of increasing scores, with the low score used as a reference category. For the case of PHQ-9 surveys, both the total score and the score for question 9 were used, with the latter selected over the former by the LASSO model selection process. Similar associations between model coefficients and the magnitude of indicators were also seen for pulse pressure, hemoglobin levels, and the three categories of head injury: headache, concussion, and TBI. Metastatic cancer diagnoses had a much stronger association than non-metastatic cancer diagnoses. Accidents due to falls had a much stronger association than those due to motor vehicles or other causes. Specific categories of mental health diagnoses, medications, and CVD diagnoses were more strongly associated than others.

An important aspect of our Rcc study design is that acute and distal predictor variables are separated across eight time bins before each event. This is indicated in Fig. 4 by the shading of time bins, with darker colors indicating more acute predictors. Thus, we can see that metastatic cancer diagnoses, use of anti-anxiety medications, and diagnosis with suicide ideation or personality disorder are more relevant 3–6 months before the outcome, while pulse pressure, accidental falls, and CVD diagnoses are more relevant 3–7 years before the event.

Model coefficients for Cox-MH, Cox-Visit, and C17 study designs are shown in Supplementary Figures S3–S8. The clearest difference between study designs, already visible in Fig. 2, is the reliance of the prospective cohorts on fewer variables, and those that tend to be indicators of outcome, rather than indicative of particular pathways to these outcomes. This tendency is also quantified in the ’DoF’ column of Supplementary Table S1, which lists the number of degrees of freedom surviving model selection in Rcc, Cox-MH, Cox-Visit, and C17 as 607, 279, 439, and 308, respectively, out of a maximum possible of 1,520. The Rcc constrains more variables both because of the greater number of cases and because acute information is available for all of the cases. Cox-MH constrains fewer variables, presumably because of the lower contrast between cases and controls when all cases and controls have a behavioral health diagnosis code. Training on suicide as an outcome drops the number of surviving predictor variables to 51 in the C17 cohort.

### Model calibration

**Figure 5:**
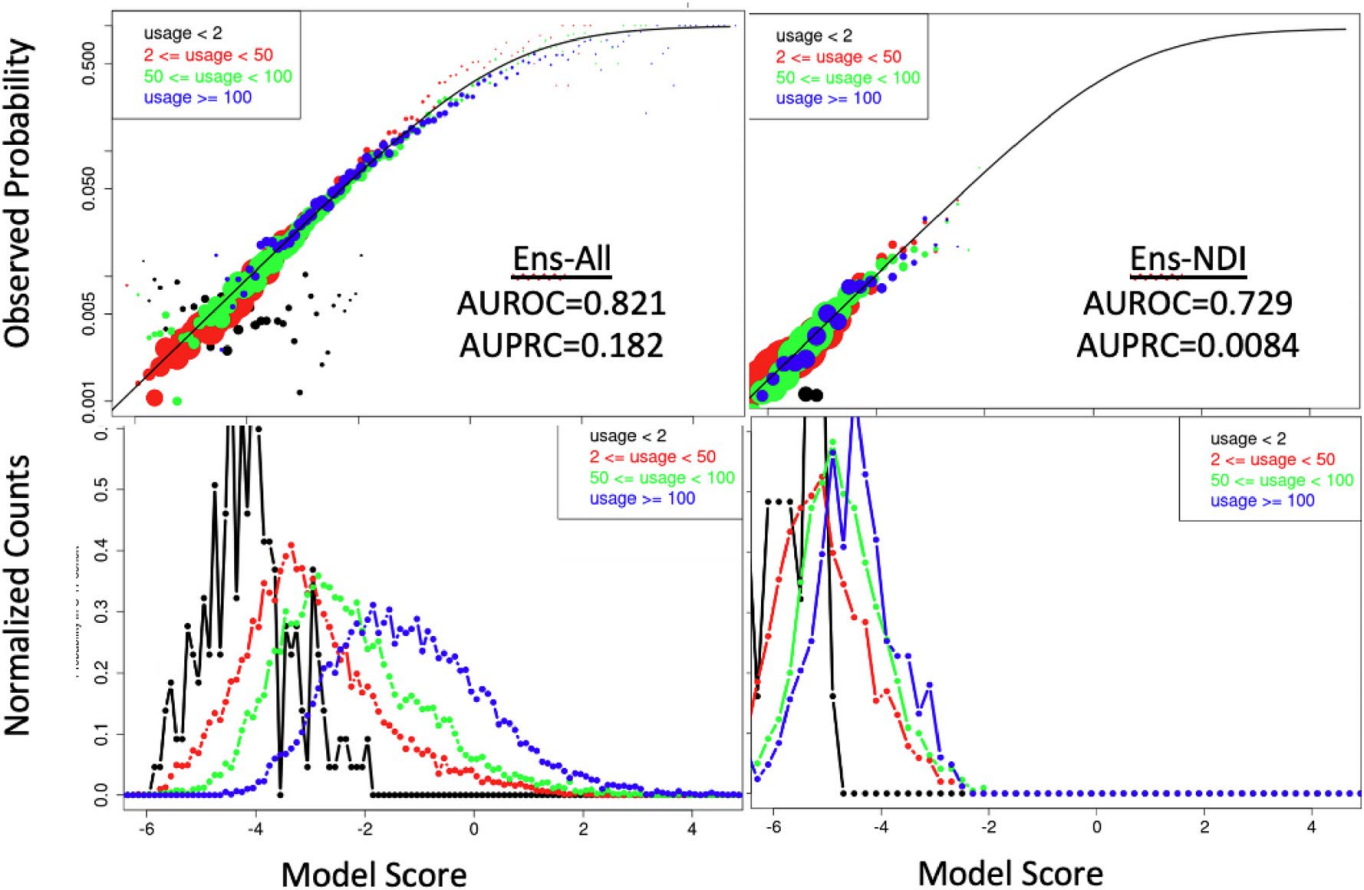
Calibration curves (top) and normalized histograms of scores for cases only (bottom) evaluated on the C17 cohort, with subgroups defined by their level of healthcare utilization according to the color code in the legend. The calibration curves stratify patients in a test set by score (x-axis) and plot the observed fraction of patients with an event on the y-axis, in this case on a logarithmic scale. The line indicates the relationship expected between a logistic regression score and the observed probability, so a model is well-calibrated when the symbols fall on the line as they do here. Results for EnsAll are at the left and EnsNDI are to the right. Healthcare utilization for a given patient is defined by summing all of the time bins with a diagnosis-coded variable present and is separated into low (red), medium (green), and high (blue) utilization. Symbol areas in the top graphs are proportional to the number of patients in a given subgroup at each score. Comparison with calibration curves for models trained on the C15 cohort is shown in Supplementary Figure S9, and analysis for other subgroups are provided as as Supplementary Figures S10–S12.

Calibration curves in subgroups complement c-statistics in assessing model performance^44^. Such curves stratify a test group of patients by their model score and assess the observed fraction having an outcome. Figure 5 shows calibration curves (top) together with associated histograms of scores for cases (bottom) for our EnsAll and EnsNDI models, both broken down into subgroups according to the level of healthcare utilization.

From the top panels, we see nearly perfect calibration for the medium (green) and high (blue) levels of healthcare utilization, with the circles largely falling at the expected probability of outcome (solid line) across all scores while the low utilization (red curve) under-predicts the 30% group (with a score of $-$ 0.3) by $\sim$ ten percentage points. Patients with zero or one diagnosis-coded variable (black) are well-calibrated for suicide prediction, but do not show increasing events with increasing scores, potentially indicating a confounding effect (patients with zero or one Dx code are unlikely to have their suicide attempt or overdose recorded). The average fraction of cases in C17 is 2.3% for the combined outcome and 0.27% for suicides, consistent with the estimated centroid of circles in Fig. 5. Also evident is the strong dependence of the histogram of scores for the EnsAll model (left) on healthcare utilization level, and the much weaker dependence for the EnsNDI model (right).

The relatively good calibration of our ensemble models is compared in Supplementary Figure S9 to models trained on C15, and then fine-tuned to predict the respective outcome for the C17 cohort. Consistent with the lower AUROC and AUPRC scores, we see that the C15-derived models have much poorer performance at the low end of the risk spectrum, poorer performance at higher scores, and noticeably poorer calibration for all levels of healthcare utilization for the combined outcome. The particular pattern of miscalibration evident for the C15-GLM-All model in Supplementary Figure S9 is consistent with the linear model being unable to account for the nonlinearities of redundant indicators, which should shift to lower coefficients when more information is present. This would lead to the observed underestimation of outcome probability for the red (low usage) curves and overestimation for the blue (high usage) curves.

Better subgroup calibration in the ensemble than the C15 trained models is also evident in Supplementary Figures S10–S12 for indicators of suicide, selected mental health diagnoses and treatments, and the demographic variables of race, ethnicity, gender, and age. When models are well-calibrated, the score histograms will accurately reflect the risk histogram of observed cases for each subgroup. Thus we can observe in Supplementary Figure S10 that a diagnosis of suicide ideation in any time bin increases the risk score by $\sim$ 2.5 units for the EnsAll model and $\sim$ one unit for the EnsNDI model. Smaller shifts are evident for the other three indicators shown. From Supplementary Figure S11, we observe similar shifts in risk for both EnsAll and EnsNDI for schizophrenia and bipolar diagnoses, despite their differing coefficients in the Rcc linear model. Shifts for diagnosis of major depressive disorder and anxiolytic prescriptions are about half as large, and the shift in risk for patients receiving opioid medication for pain is minimal, even though the Rcc linear model coefficient is 0.6. Several features are important in the demographic subgroups presented in Supplementary Figure S12. Female, Black, Hispanic, and young (<55) subgroups all have similar risk profiles to the average for our combined outcome, while old (>65) patients show much lower risk. In the case of suicide, however, the risk is much lower for females and Blacks, and elevated for younger patients.

### Variation with age and sub-outcome

**Figure 6:**
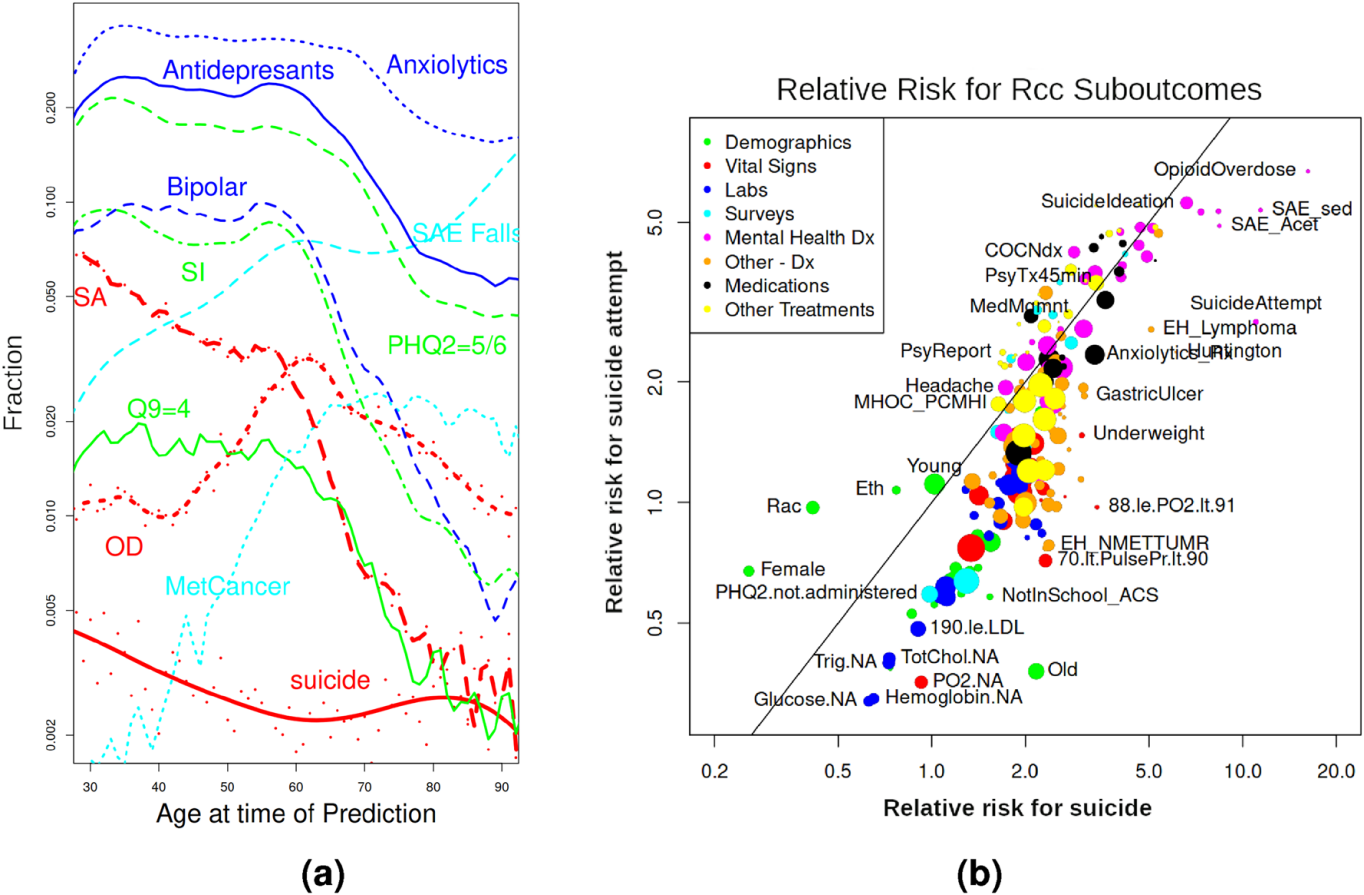
(**a**) Age dependence of the fraction of total patients in the C17 cohort with the indicated components of outcome (red) and selected predictor variables, plotted on a logarithmic y-axis. Lines are smoothing splines, while red curves also show the raw data as points. Note the stability of suicide across the age range, relative to the other studied sub-outcomes of suicide attempt (SA) and overdose (OD), as well as the selected predictor variables shown. (**b**) Comparison of the relative risk for subgroups defined by each of the model variables (summed over time) in the Rcc cohort, compared to all patients, for the two sub-outcomes of suicide and suicide attempt.

Motivated in part by the large differences in suicide and combined outcome risk among demographic subgroups, we present in Fig. 6a the age dependence of our outcome components and selected predictor variables for the C17 cohort. Evident in this figure is the predominance of suicide attempts below age 60 years and overdoses above, with a decrease in overall age-dependent rates by five-fold as age increases from 30 to 80 years. Suicide rates, by contrast, are relatively constant above the age of 45 years. Reported instances of suicide ideation, diagnosis of bipolar disorder, the prescription of antidepressants and anxiolytics, and results from PHQ2 and PHQ9 surveys all follow a similar trend, of being highest for the under-60 patients and dropping off ten-fold or more for older patients. Reported instances of severe adverse effects from falls and the diagnosis of metastatic cancer increase sharply with age.

We are also interested in the differential predictors of suicide and suicide attempt. Figure 6b compares the relative risk for each of our predictor variables. Patients are included as cases for a given variable if that variable was present in at least one of the eight time bins. Mental health diagnoses show a relative risk ranging from 1.5 to 5, and are roughly equally enriched in suicides as suicide attempts (magenta circles). Notable differences are evident for severe adverse effects of sedatives and acetaminophen, as well as opioid overdoses, which are two to three-fold more enriched in suicide than suicide attempts. Another notable feature is the cluster of ’NA’ variables that are also two-fold more enriched in suicide than suicide attempts. This effect likely reflects confounding because both predictor variables and the suicide attempt outcome depend on healthcare utilization, while NDI-reported suicides do not.

Several predictors are outliers in being more indicative of suicide than suicide attempts, including elevated pulse pressure, cancer and lymphoma diagnosis, being underweight, and use of anxiety medications. Note that the association of previous suicide attempts with suicide over suicide attempt is likely strongly influenced by the use of first suicide attempt in the combined outcome in the Rcc cohort. Increased relative risk for suicide attempts over suicide is indicated by several demographic groups, such as being female, Black, and to a lesser extent, being Hispanic. A listing of attributes of selected variables is provided in Supplementary Table S3, and a complete list in Supplementary Information SI-5.

### Implications for screening

**Figure 7:**
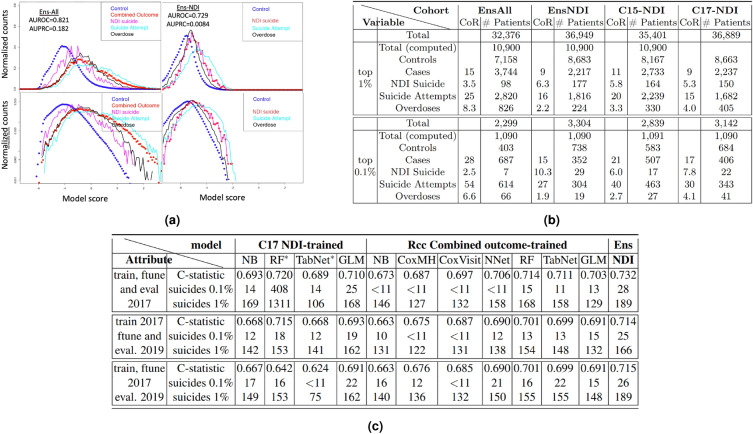
Implications for patient screening: **(a)** Model performance for component outcome, by model score. Normalized histograms of scores are shown for (left) EnsAll and (right) EnsNDI models predicting outcomes for the C17 cohort. Top panels have a linear y-axis and bottom a logarithmic y-axis. Cases (red) and controls (blue) are plotted as circles, while individual sub-outcomes are shown as lines colored magenta (suicide), cyan (suicide attempt), or black (overdoses). **(b)** Model performance in high risk screening, by sub-outcome. Comparison of the two ensemble models and two models directly optimized C15 and C17 cohorts on NDI deaths, at identifying the top 1% and 0.1% risk tiers in the C17 cohort for each component of our outcome. Rows in the table report the total number of controls and cases, broken down by component outcome. Total (computed) is the total sample size evaluated in the top 1% and 0.1%, while the rows labeled Total are corrected for the four-fold enrichment of cases in our calculation, thus reflecting the denominator in the original C17 cohort, that of all patients with a visit in the four months prior to the time of prediction. **(c)** Assessment of model drift by predicting on holdout (unseen future) NDI-reported suicides for two years after January 1, 2019 for several models trained on 2017 suicide risk or on our Rcc cohort. Models were either fine-tuned on 2017 NDI suicides (thus having model parameters frozen before presenting with 2017 NDI data) or 2019 NDI suicides, as indicated. The two NDI-trained models marked with ($^*$) were known by comparison of test (odd cohorts) and train (even cohorts) data to be overfit. Their incorporation into our ensemble models degraded performance and they are kept in the table primarily to illustrate how overfitting propagates through our evaluation protocol. C-statistics for the fine-tuning of models are the average for training on even cohorts and testing on odd and vice-versa. Number of suicides predicted to be in the top 1% and 0.1% of risk are indicated for all models, and will require screening of a similar number of patients as indicated in Fig. 7b; a four-fold random down-sampling of controls occurred for this (and all other) prospective study design (3,300 for top 0.1% and 35,000 for top 1% after correcting for down-sampling of controls). We see that the EnsNDI model is robust against two years of model drift and outperforms the other models presented. There were only 2,689 suicides in C19, down 5% from 2812 in C19. Specific patient numbers below 11 are not provided, per VA privacy protection policy.

Figure 7 quantifies several aspects of our ensemble models for the screening of at-risk patients. The normalized risk histograms in Fig. 7a show that cases are shifted to higher risk scores throughout the distribution, with the suicide attempt curve (cyan) shifted more than that for suicides (red), and with a greater shift at high scores than low. This improved discrimination at higher scores can be better seen in the bottom panel, where the logarithmic y-axis means that the vertical distance between curves is proportional to the ratio of the fraction of patients with each outcome at each risk score along the x-axis. At the tail of the distribution, this ratio is similar to what McCarthy et al.^18^ called the concentration of risk. For suicide deaths (magenta line vs. blue dots), these ratios are 6.3 for the top 1% and 10.3 for the top 0.1%. These and similar values for other output components can be found in the table of Fig. 7b.

Figure 7a shows that it is much easier to identify suicide attempts than suicides, even for a model optimized for identifying suicides (the cyan curves are both shifted more to higher risk than the red curves), and all models show a 2–20 fold higher concentration of risk (CoR) for suicide attempts than suicides. Also, our model distinguishes cases from controls throughout the risk spectrum, showing an increasing positive predictive value in the high-risk tail of the risk distribution, most readily seen with the logarithmic scale. Overdoses are the most poorly predicted component in three of the four models, as indicated by CoR. This could be because overdoses have very different predictors than suicide attempts or because our combined outcome definition assigns patients with both suicide attempts and overdoses to the suicide attempt category.

Figure 7b compares four model training strategies in predicting C17 cohort outcomes. The first model comparison shows that EnsAll identifies 614 patients attempting suicide out of the 1090 patients in the top 0.1% risk in the down-sampled sub-cohort, corresponding to a 20% positive predictive value (PPV) for our combined outcome across the active VA patient population. When optimizing the ensemble model to identify NDI-reported suicide, the most important metric for the ReachVet program, EnsNDI identified 29 suicides in the top 0.1% risk strata, shown in the third row from the bottom of Fig. 7b, or a PPV of 1% in the original C17 prospective cohort. In addition to our EnsNDI and EnsAll models, the table provides results for models trained on suicides (C15-NDI and C17-NDI) before fine-tuning on C17. These two models correspond to two logical ways of making prospective cohort predictions. The C15-NDI model is trained on the most recent two-year period for which data are available in time to prospectively evaluate on the C17 NDI cohort. The C17-NDI column split the C17 cohort into even and odd date of birth cohorts, training on each half while evaluating on the other, then reporting the sum (or the average, for the rates) result in the table. Both prospectively trained models were less sensitive than the EnsNDI model at predicting suicide within the high-risk strata, with the C15 model performing more poorly than C17, even though C17 was effectively trained on only half of the data. This speaks to a lack of model generalizability across time or model drift.

In Figure 7c, we assess this two-year time drift more carefully—taking advantage of a holdout set of NDI-reported suicides for 2019 and 2020 and made available to us after the development of all of the above-presented models. The top group of numbers provides a baseline performance of twelve models predicting two-year suicide risk from January 1, 2017. The first four models were trained directly on two-year suicide risk in the C17 cohort, then fine-tuned with the ten (time-independent) variables shown in Fig. 2. For all linear models (GLM, Cox, and all fine-tuning) two models were trained (even and odd cohorts as training) and the average of model coefficients was used as the final model. For non-linear models, the model trained on odd cohorts was used to evaluate all data. We note that the non-linear NDI-prospectively trained models were seen to be overfit during training, with the NNet model not converging (and so not shown), the RF strongly overfit, and the TabNet model simply performing poorly. The GLM model performed best, but its LASSO model selection down-selected to 51 variables, compared to 607 for the GLM combined outcome model. The naive Bayes model was not overfit but also performed relatively poorly. Training on the combined outcome and fine-tuning to the NDI-suicide outcome provided better performance than direct training on NDI outcomes (where the fine-tuning was somewhat superfluous), with the non-linear models consistently outperforming the linear models. As noted in Fig. 2, the EnsNDI model performance is two percentage points better than any of the component models.

The bottom set of numbers in Fig. 7c evaluate how the models trained without ever seeing the NDI-suicide data after January 1, 2019 perform on two-year suicide prediction from that date (C19 cohort). We observe first that the NB, RF, and TabNet models trained directly on the C17 NDI-suicide outcome perform much worse on the C19 data, with 6–8 percentage point decreases in c-statistic. The rest of the models, including EnsNDI, dropped by 1–2 percentage points, with the linear models losing less than the non-linear ones. It should be noted that three of the eight time bins of the predictor variables moved from ICD-9 to ICD10-coded variables in moving from C17 to C19 outcomes. To assess whether the decrease in performance was due to the base-model training or fine-tuning, the middle set of numbers kept the 2017 predictions but enabled fine-tuning on 2019 data. We see that the updated fine-tuning improved neither EnsNDI nor base model performance. Perhaps surprisingly, updating the fine-tuning appears to have decreased the ability of most base models to identify the top 0.1% suicide risk.

## Discussion

We find that study design, variable definition, prediction methodology, model characterization, and identification of cases in a prospective cohort are intertwined, with choices made in one aspect impacting another. Our workflow utilized the observation, explained in detail by Ernster^29^, that retrospective nested case–control (our Rcc) and case-cohort (our C17) cohorts are well-suited for identifying biological drivers of outcome and predicting cases in a prospective population, respectively. Although primarily motivated by efficiency in sampling patients, she also noted that matching can alleviate the impacts of confounding variables and push the model toward more mechanistic and generalizable models, while maintaining a well-defined relationship to a cohort facilitates comparison across analyses. Ensemble transfer learning exploits these designs in several ways. First, the Rcc cohort is structured to maximize statistical power in training non-linear models, and is matched in order to minimize the role of age and healthcare utilization in the Rcc model. Second, ensemble learning provides a systematic way to identify and leverage the ML models with differing choices in outcome variable definitions. Third, because both Rcc and C17 models are derived from the same initial cohort, the transfer learning has a simpler task in fine-tuning the predictive model from Rcc to C17; the variables that need to be fine-tuned are those involved in matching in Rcc (age and level of healthcare utilization), those changing with outcome definition, and other outlying data points in Fig. 2, such as demographics.

While the above arguments should seem plausible, any ML-based workflow should be considered heuristic and be carefully characterized for discrimination and calibration in important subgroups defined by demographics, potentially confounded variables, risk strata, important indicators, risk factors, treatments, and sub-components of the outcome. For linear models, training on outcomes that combine common, less serious, outcomes with those that are rare but more serious can be problematic^31^. We see from our calibration curves in subgroups and the sensitivity for predicting suicide in Fig. 6b that our nonlinear EnsNDI model for suicide did not suffer from subgroup-specific biases.

Having a model of reasonable accuracy that is calibrated across the above set of subgroups should enable two distinct surveillance strategies to be examined. First, one could reach deeper into the risk pool by subgrouping on patients likely to benefit more from intervention, such as suggested by Pisani et al.^3^. Second, one could lump together patients from often-misidentified outcomes to establish need, then subgroup based on the type of need. We expect that a single, non-linear model that is robust against the multitude of complex variables will outperform stratified or separate models by making more effective use of the limited number of cases observed for suicide as an outcome.

Figure 4 provides a rich overview of logistic regression coefficients, developed on a date of birth- and age-matched Rcc model predicting on 961,423 controls and a combined outcome with 31,371 suicides, 136,351 first reported suicide attempts, and 92,857 first reported overdoses. While our linear Rcc model ignores interaction terms^45^, uses incomplete data, and contains incompletely controlled biases, these findings, shown in Fig. 4, provide a rich set of predictive coefficients of our combined outcome, and, to the extent that Fig. 6b is well-correlated, also suicide. In our Supplementary Discussion section, we compare predictors in the areas of indicators of suicide, substance use disorder, mental health diagnoses, and treatments, indicators of hypoxia and vascular damage, and demographics to the literature, finding a broad degree of support.

Because our EnsAll and EnsNDI models are well calibrated across a wide variety of subgroups, it should be possible to use explainability techniques such as LIME^46^, SHAP values^47^, and saliency maps^48^ to probe details of interdependent risk factors of suicide. This has been illustrated for some of these methods for a predictive model of suicide attempts^49^.

Extensive prior work predicts suicide risk from EMRs^5,6,9,15,18,19,50^, yet demonstration of suicide reduction as an endpoint has been difficult^7,22^. In this work, we suggest that the $\sim$ 3,000 NDI-reported suicides that occur in a two-year period among active users of VA medical care are insufficient to constrain a predictive model of suicide without relying heavily on coarse-level indicators, and that this has the side-effect of identifying primarily patients who are already well-known as suicide risks in the EMR system, as observed by McCarthy^18^. We present an ensemble transfer learning approach that enables us to effectively utilize a combined outcome and aggregate across fifteen years of outcome data, while accounting for the interaction terms and confounding effects related to age and level of healthcare utilization. Critical to our effort is extensive calibration analysis in a wide variety of subgroups, evaluation of accuracy metrics in prospective cohorts nested into the cohort of all active VA patients, and comparison of risk factors emerging from our matched Rcc cohort to those obtained from literature.

We illustrate the difficulty of comparing models across reported studies with a few recent examples. Alemi et al.^15^ report a c-statistic of 0.77 for predicting ’suicide or intentional self-harm’ from a cohort of veterans similar to our own. When we applied the naive Bayes methodology that they used to our C17 cohort, we obtained a c-statistic 0.741 for predicting our combined outcome and 0.665 for predicting NDI suicides. This is well below our EnsAll model performance of 0.83, highlighting the caution needed to avoid conflating a serious and rare outcome with a less-serious and more common outcome, as explained in Pocock et al.^25^. Two articles examining ninety-day suicide risk in non-veteran cohorts with similar size and available predictor variables^6,9^ provide another useful comparison. In broad terms, their list of important predictor variables is similar, highlighting in particular the value of PHQ9 scores. Their reported c-statistic for predicting ninety day suicide risk, based on a cohort with 1240 cases, is 0.833, much larger than our value of 0.714 for two-year risk derived from a data set with 31,371 cases in our Rcc cohort. These values cannot be directly compared, however, for several reasons. First, their choice to predict across patient-visits rather than patients creates a more unbalanced data set, which directly affects the c-statistic, as explained by Cook^44^. It also biases the sample towards patients who visit more frequently, thus having more extensive medical records, providing a stronger basis for identification. Finally, their process of randomizing training and test sets across visits, particularly the highly-correlated multiple visits in the ninety days before a reported suicide, may strongly impact the generalizability of their model. While generalizability to independent healthcare systems^30^ is a reasonable standard for general adoption of a medical outcomes model, which historically has been problematic for predictive models of suicide^7^, both Justice *et al.*^30^ and Ernster^29^ suggest prospective validation in an easily defined (nested) cohort as a valuable intermediate step to assess the value of models predicting medical outcomes.

In terms of the clinical implications of our study, we have not changed the underlying facts that suicide is relatively rare^16^ with a broad array of non-clinical drivers^2^ and risk factors that are difficult^51^ or very difficult^52^ to quantify. We have, however, provided in Fig. 4 a set of self-consistent, time-resolved, linear risk factors for our combined outcome, together with a discussion of how predictor variables differ in their relative risk of suicide and suicide attempt, in Fig. 6b. One feature evident in these risk factors is epidemiological support for the role of vascular damage and hypoxia in suicide and suicide-related behavior, which has support in the literature, discussed in the Supplementary Discussion section, along with the other risk factors. Another feature is the importance of indicators of suicide, such as the answer to question 9 of the PHQ-9 survey and diagnoses for suicide ideation, suicide attempts, or visits to the emergency department. While the linear risk associated with indicators presented here are unlikely to generalize well to other populations (this is the nature of indicators), they are indicative of what is possible. If PHQ-9 surveys are not regularly given or suicide ideation not diagnosed, this evidence suggests they may be of significant value. Also, we have provided a model, based substantially on non-linear drivers of suicide described in Fig. 4, that predicts suicide better than models based entirely on demographics and indicators and is well-calibrated for many important subgroups of patients, such as those defined by age, gender, and healthcare utilization. This provides a path forward to reaching deeper into the risk pool and identifying patient subgroups who may be well-suited to particular interventions, as suggested by Pisani et al.^3^ and Sorter et al.^53^. Finally, we have provided a rich baseline model from which we can evaluate the utility of novel predictors of suicide and suicidal behavior, such as genetic markers^54^ or thought markers, described in Pestian et al.^55^.

This study suffers from many important limitations. Primary among these are missingness of information and difficulty in quantifying the diverse drivers of suicide-related behavior. We have primarily relied on structured clinical data from the VA EMRs, and not utilized Centers for Medicare and Medicaid Services (CMS) data, service records from the Department of Defense, Veteran’s Benefits Administration, or specialized databases on veteran suicide. Additionally, we have not utilized clinical notes, which would require natural language processing, or genetic information in our analysis. While the diverse trajectories leading to suicide suggest the need for diverse predictors, our broad base of information, explicit treatment of data missingness, and the existence of studies suggesting minimal impacts on mortality prediction when adding Medicare claims data to VA EMRs^56^ suggest our results can serve as a firm baseline for predicting suicide.

Our cohort was composed of US Veterans who are adults, 90% male, and have a median age of 65 years, (see Supplementary Table S2), and so the pathways towards suicide may not generalize to other populations. Our study has utilized only representative ML predictive models, with a focus on simpler and more generally understood methods, to more clearly elucidate the interplay between study design, ensemble transfer learning, and model performance. It appears likely that more elaborate base models will further improve model prediction accuracy.

## Conclusion

We present predictive models of suicide and a combined outcome of suicide, suicide attempt, and overdose that use 190 longitudinal and 20 cross-sectional hand-curated variables. To identify mechanistic predictors of our outcome, we use a retrospective nested case-control (Rcc) study design^29^ to predict our combined outcome on 260,579 cases and 961,423 controls matched on date of birth and overall health care utilization, with variables defined retrospectively from the date of the event. This creates enough statistical power for LASSO model selection to identify a broad range of risk factors involving demographics, vital signs, laboratory results, PHQ surveys, a wide range of medical diagnoses, medications, and behavioral health treatments.

We found that the application of ensemble transfer learning techniques combined several nonlinear models using our Rcc cohort with prospective cohort models to yield predictions with c-statistics of 0.73 for predicting suicide and 0.82 for predicting our combined outcome, and were well calibrated across a wide variety of subgroups. EnsNDI outperformed the base models in placing NDI-reported suicides in the top 0.1% of risk, and suffered a two percentage point drop in c-statistic with two-years of model drift. The number of patients identified at the top 0.1% risk strata for two-year risk suggests a 1% positive predictive value (PPP) for suicides in the active VA patient population using our EnsNDI model and 20% PPV for our combined outcome using our EnsAll model.

## Methods

The VA EMRs contain multimodal data for 24 million patients, so we created a workflow that supported iterative steps of pulling and linking the data from the patient database, defining the study cohorts, defining the variables, encoding the variables, training the predictive models, creating the ensemble models and fine-tuning, and finally the evaluation and visualization of results. Two R scripts and one Python script are provided in the Supplementary Materials that were used to perform all of these analyses. To facilitate the analysis, the data were pulled from the patient database into 100 cohorts of 240,000 patients each, sorted according to their date of birth. All patients with a birth date on or after January 01, 1901, and one or more visit recorded after June 30, 2007, was considered in our studies. The different study designs imposed additional inclusion and exclusion criteria.

### Definition of outcome variables

Outcome variables were constructed from three component outcomes: suicides, suicide attempts, and overdoses. Suicides were reported through the National Death Index matched by patient to VA medical records, and then using regular expressions ’${^{\hat{\,}}}$e95’ to search for ICD9 codes, and ’${^{\hat{\,}}}$T14.91’ or ’${^{\hat{\,}}}$T3[6789]..X2’ or ’${^{\hat{\,}}}$T[456]..X2’ or ’${^{\hat{\,}}}$X[678]..XX’ or ’${^{\hat{\,}}}$Y87.0’ for ICD10 codes, in either underlying or contributing causes of death. Suicide attempts were identified using the same set of regular expressions from outpatient visits, inpatient admission, and discharge diagnoses, and from the fees table, reflecting reimbursed care provided outside of the VA system. Overdoses were identified from the same set (outpatient, inpatient, and fees) of diagnosis codes, but using regular expressions ’${^{\hat{\,}}}$e850’ or ’${^{\hat{\,}}}$e8[67]’ or ’${^{\hat{\,}}}$e935’ for ICD9 codes and ’${^{\hat{\,}}}$T40’ for ICD 10 codes.

For the Rcc cohort, we allowed the outcome dates only from Aug 16, 2007 to ensure opportunity for the full 7.5 years of longitudinal information in the predictor variables, as the EMRs consisted of data from Jan 01, 2000 onwards. Likewise, the time of prediction was set to 45 days (fixed gap) before the outcome date to ensure that there was no outcome information leakage in the predictor variables. This creates one of several arbitrary choices associated with the Rcc study design: including patients with suicide attempts and overdoses before this cutoff will enable suicide attempts to predict suicide attempts across this boundary, while this will not occur after 2007 because the outcome is defined as the first instance of a suicide attempt outcome. Ultimately, this problem is resolved here by including suicide attempts and overdoses in the fine-tuning process and evaluating accuracy scores only on prospective cohorts that have been fine-tuned. Similarly, the time of prediction for the prospective cohorts (C15, C17, and C19) was set to be Jan 01, 2015, Jan 01, 2017, and Jan 01, 2019, respectively. To ensure no outcome information leakage, we excluded the outcome in the first week after the time of prediction. A shorter gap was used than for Rcc because most events are more than several week after the time of prediction, and we wanted to keep as many events as possible, for statistical power.

Definition of cases for our combined outcome was done as follows: if there was a death by suicide in the allowed time window, then this was used as the outcome. If not, the outcome was defined as the first recorded suicide attempt within the allowed window. If there was no documentation of suicide or suicide attempt, then the first recorded instance of an overdose in the window was used as the outcome. This heuristic enables the model to learn the predictive values of death by suicide, suicide attempt, and overdose while utilizing all recorded suicides and not repeatedly counting patients with multiple events.

### Treatment of outcome variables

Suicide provides a binary outcome and cannot recur, so is straightforward to use as an outcome for each of our models. Our motivation to combine suicide attempts and overdoses with suicide is partly the relatively low incidence of suicide, but also the ambiguity surrounding the cause of death and ascertaining intent for potentially suicidal behavior. Additionally, this study’s goal of comparing study designs and transfer learning is greatly facilitated by creating a binary variable. Hence, we enrich in more serious events (prioritizing suicide, then suicide attempts, then overdoses), and as a secondary criterion, choose first-recorded events after the time of prediction over subsequent events.

Constructing our combined outcome by prioritizing suicide, then suicide attempts, and then overdoses creates side effects in how suicide attempts and overdoses appear as predictor variables in the different study designs. The calendar-based cohorts (C15, C17, and C19) are the most straightforward, where any suicide attempt and overdose before the time of prediction can be used as a predictor variable to predict the combined outcome obtained as described above. Our matched, nested, retrospective, case-control (Rcc) study design shows the strongest effect, where our use of first-recorded component outcome (in accord with the rules described above) to define the time of prediction, from Aug 16, 2007 means that while overdoses will still predict both suicide attempts and suicide, and suicide attempts will predict suicides, very few suicide attempt will predict other suicide attempts and very few overdose will predict another overdose.

The event-driven time-to-event (Cox) models are intermediate, where the time of prediction is triggered by either a psychiatric evaluation (Cox-MH) or an office visit (Cox-Visit). Since the first such visit in a three-year window starting from July 1, 2007 is used, this study design also has the potential to skew the presence of predictor variables. This complex interplay of suicide attempts and overdoses as predictor variables is rooted in the distinct study designs of Rcc and prospective cohort studies^29^. This dependence motivated their inclusion in our transfer learning process where we utilize knowledge learned from all the other cohorts to improve learning and prediction in our C17/9 cohorts.

### Study cohorts

We analyzed 24,558,158 distinct patients from the VA electronic medical records (EMRs), with a date of birth on or after January 01, 1901. Our study was focused on patients with the outcomes of interest, including death by suicide (as recorded in the National Death Index or NDI), suicide attempt, or overdose (the latter two based on diagnosis codes). The top panel of Fig. 1 shows histograms of the first occurrences (since there can be more than one suicide attempt or overdose for a given patient) of individual components of the combined outcome vs. time for all cases between January 2000 and December 2021.

Five study cohorts were used in this work as characterized in Supplementary Table S2. First is a retrospective case-control (Rcc) cohort that is defined by time-dependent variables in the indicated windows before an outcome event (cases) or a randomly selected diagnosis code (control). For each case, four control patients were chosen by randomly selecting medical visits among patients without an outcome event, but over the same period as the outcome date for the cases, and then selecting one visit for each of the four chosen controls. The controls were chosen from the same date-of-birth cohort as the cases to ensure that cases and controls have roughly the same age distribution and their randomly selected medical visit was treated as their ’outcome date’. This allows people with more medical visits to be preferentially chosen as controls, which both balances cases and controls on the overall level of healthcare utilization and selects an outcome time for controls corresponding to a period of healthcare utilization. Matching of variables in a case-control study design is a well-established procedure^28^ which is often motivated by modeling of rare events, but can also help in mitigation of confounded variables and those with strong, non-biological, interaction terms^29^. Matching can also introduce biases and becomes progressively more complex as the number of matched variables increases^28^. Consequently, we chose to match on only two of the most important confounding and interacting variables, healthcare utilization (see Fig. 5) and age (see Fig. 6a) and do so with simple algorithms.

In addition to Rcc, we used several prospective cohorts. Time to event (Cox) models were constructed for patients with a psychiatric evaluation (StopCode 502, Cox-MH) or office visits (StopCode 323, Cox-Visit) in the three years starting on July 1, 2007, with the time of prediction being the date of the psychiatric evaluation or office visit, respectively. For both of the time-to-event (Cox) models, the goal was to mimic a clinical decision point and compare the information content in these models to the other study designs. We chose a three-year window for the initiation event to allow a lengthy (ten year) follow-up period for patients. Time to event models were right-censored on deaths, but not on last visit to the VA, specifically to include patients who may have dropped out of care. In the end, Rcc provided a richer set of mechanistic predictors and interval prediction of prospective two-year risk provided a more flexible characterization of prospective risk, so relatively little analysis of these study designs occurred here. Finally, we used two prospective calendar-based cohorts for all patients with a recorded visit in the three-month period before the time of prediction, which is either January 01, 2015 (C15) or January 01, 2017 (C17). For each cohort, data were binned in the eight time periods, and the number of controls and cases for each sub-outcome are indicated. These cohorts capture different aspects of the study with the Rcc matched on age and overall level of healthcare utilization, and outcome events spread across 15 years and the prospective cohorts designed to accurately predict suicide risk in a prospective cohort.

A gap between the time of prediction (which is the end of the observation period) and the event was incorporated to prevent inappropriate leakage of outcome-related information into the predictor variables. For the retrospective design (Rcc), the event (for cases and controls) could occur at any time between August 15, 2007, and January 1, 2022, and the gap between the time of prediction and the event is exactly 45 days. For prospective design (C15/17, Psych-ds, and Visit-ds) the gap was only seven days.

The time bins in the observation period were organized identically for all study designs. They increased in width as they moved more distant (earlier) from the time of prediction, on a logarithmic scale in two periods of three months, two periods of six months, two periods of one year, and two periods of two years (see bottom of Fig. 1). This was done to enable cross-prediction of models, whereby they are trained on one cohort (typically Rcc) and evaluated on another typically C17). The fine-tuning of the handful of variables involving demographics, overlapping with outcome definitions, and matching, enabled accuracy scores of Rcc-trained models to be competitive with prospectively-trained models, when predicting in prospective cohorts, as summarized in Supplementary Table S1. As shown in this table, the Rcc cohort was used to train logistic regression, a fully connected neural network with three hidden layers, random forest, and TabNet models for the combined outcome. The Cox-MH and Cox-Visit cohorts were used to train Cox models for the combined outcome, and the C15 cohort was used to train two logistic regression models, one for the combined outcome and another for suicide. All the models were then individually fine-tuned on the training set of the C17 cohort and their c-statistic on the test set of the C17 cohort was reported. This process created base models, to be used in our ensemble transfer learning approach, discussed below in the Prediction Methodologies section.

Supplementary Table S1 shows c-statistics for our base models, evaluated both the Rcc and C17 cohorts, before and after an early version of our fine-tuning procedure. Several results evident in this table were important in developing our EnsNDI and EnsAll models. In this table, the rows correspond to ten models trained on our combined outcome plus one C17 logistic regression model trained on two-year NDI suicide risk, while the columns refer to cohorts and outcomes (combined or NDI suicide) that each of the models was evaluated against. For each model/cohort pair, the top number is the accuracy score before fine-tuning and the bottom number after. For cases where c-statistics differed more than 0.2 percentage points between test and train cohorts, they are provided as train/test pairs. Important observations from this table include: Rcc models transfer to prospective cohorts much better than in the reverse direction. Combined outcomes can be predicted more accurately than suicides. Nonlinear models applied to prospective cohorts predicting NDI suicide are prone to overfitting. None of the base models performed as well as our ensemble models, defined in Fig. 3 and characterized through this work. The difficulty in training models of the dimensionality used here against prospective suicide risk is evident from: 1) the very low number of degrees of freedom (in ’DoF’ column), of 51, making it through LASSO model selection when training C17 for suicide outcome, 2) the 1 percentage point difference between test and train for the Rcc-trained models predicting on C17 NDI suicides, and 3) the significant difference between test and train accuracies evident for the non-linear models when evaluated against NDI suicide outcome after training prospectively on our combined outcome. If accuracy scores alone were used to assess models, fewer differences would be seen among models.

### Predictor variable definitions

Once the date-of-birth cohorts were defined, we worked systematically across 42 specific tables, subsetting by date-of-birth cohort, linking to national patient identifiers, unique inpatient or outpatient visit identifiers, and standard ontologies for each data domain.

#### Variables from diagnosis codes

The ReachVet program, administered by the VA OMHSP, has developed a list of hand-curated diagnosis codes, building on McCarthy et al.^18^. These definitions have evolved over the past ten years with significant input from clinicians and continuous evaluation of the ReachVet and other behavioral health intervention programs as part of VA operations. We mapped International Classification of Diseases (ICD9 and ICD10) diagnosis codes onto variables relevant to their operational experience. To harmonize the definitions, we accounted for the differences between these two ICD systems by inspecting the histograms of code usage over time and lists of the most-used ICD9 and ICD10 codes for each variable, supplied as Supplementary Information SI-2. Diagnosis codes for predictor variables, as with outcome variables, are obtained from outpatient, inpatient, inpatient discharge, and fees tables of the VA EMRs. All the variables based on the diagnosis codes, medications, and procedure codes were binned into the time bins described in 1 and coded as ’absent’ (zero) or ‘present’ (one).

**Figure 8:**
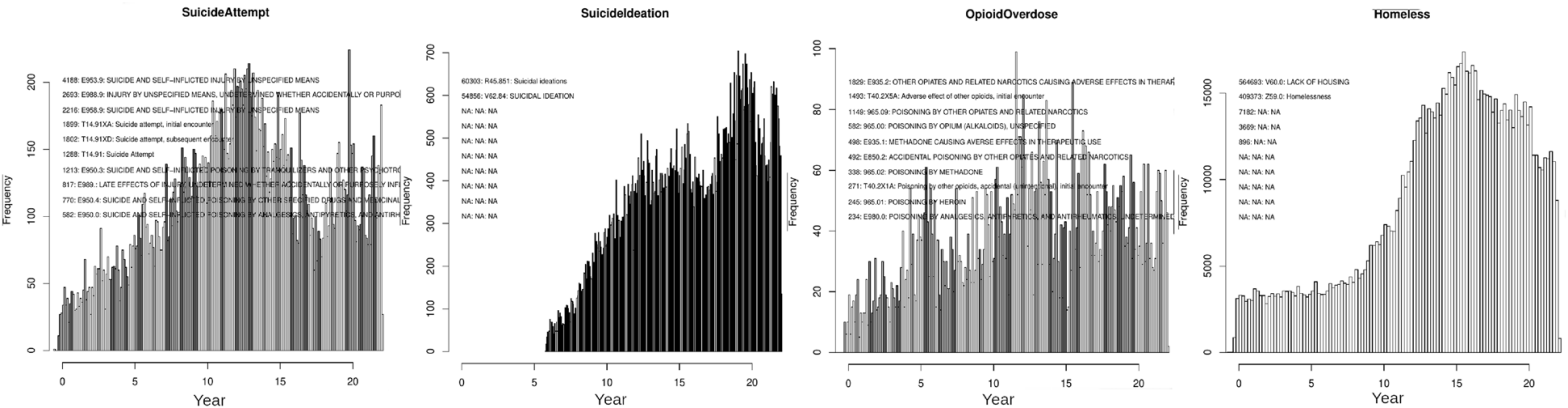
Histogram of monthly incidence of four representative variables in our model, illustrating representative time-dependent behavior of the recorded variables. Histograms similar to these, of each of the 76 Dx-code-based variables together with the ten most prevalent of the component ICD9 and ICD10 codes comprising them are provided as Supplementary Information SI-2.

An essential consideration of a study cohort is to account for the evolution of the diagnostic code assignments over the entire study period. Changes in clinical coding practices and the ICD-9 to ICD-10 changeover (October 2015) are documented in the yearly updates to coding manuals. The impact of these changes can be seen in Fig. 8, where the first panel shows the monthly suicide attempts from 2000 until January 2022. Notice the two-fold drop-off in recorded suicide attempts at the ICD-9 to ICD-10 changeover (Oct 2015).

Individual panels in Fig. 8 also include the ICD-9 and ICD-10 codes contributing the most to each variable based on the number of occurrences in a 3% sample of the patients in the legends. These include patients in the 30th, 50th, and 70th date of birth cohorts. Examination of the specific codes involved shows the difficulty in mapping the ICD-9 and ICD-10 codes to *suicide attempts*. The top three ICD-9 codes (E953.9, E988.9, and E958.9) lump together suicide and self-inflicted injury, while the ICD-10 version of this variable, T14.91, is specific to suicide attempts.

The second panel in Fig. 8 shows that suicide ideation was not used before 2006, and its prevalence increased from 2006 until 2015 as clinicians were encouraged more and more to record it. The component ICD-9 and ICD-10 codes both indicate *suicide ideation*. The Cox-MH and Cox-Visit cohorts were designed to fit time-to-event models, with a time of prediction most frequently in 2007.

The third panel in Fig. 8, for opioid overdose, shows a three-fold increase from 2000 until 2015, broadly similar to that shown for heroin usage in reference^57^, although the VA data peak several years earlier than the national average. The fourth panel shows homelessness, which became an area of focus for the VA from 2008–2016. Over time, the increased prevalence of this variable likely reflects the greater attention this problem received by VA rather than an actual increase in homelessness.

#### Medications, procedures, and PHQ surveys

The medications were selected from the list of medications previously used by Ref.^18^, supplemented by selected drug categories targeting the central nervous system in the VA Drug Classification system, which can be found, for example, under the *Drug Products by VA Class* tab at reference^58^.

The procedure codes were selected from the list of the 25 most common Current Procedural Terminology (CPT) codes related to mental health in a sample of VA patients, and variables can be found from lines 557 to 571 of rv10.r, in Supplementary Information SI-3.

PHQ-2 and PHQ-9 surveys^43^ were encoded as total scores, along with the specific answer to question 9 of the PHQ-9 questionnaire.

#### Laboratory results and vital signs

The laboratory results and records of individual patients were linked to Logical Observation Identifiers Names and Codes (LOINC) codes for laboratory tests, MVP Core Data Team’s ’ShortNames’ for 108 of the most common laboratory tests^59^, and a customized mapping based on the VA’s LabChemTestName, VA station, and a substantial amount of hand curation. Although LOINC codes were found to be missing and mis-assigned to laboratory results in the VA EMRs, we recorded the frequency with which LOINC codes were associated with each of our laboratory results (A1c, Glucose, HDLC, LDLC, Total Cholesterol, Triglycerides, Hemoglobin, and eGFR) in the fifth tab of Supplementary Information SI-5, the ModelStats.xlsx spreadsheet.

Clinical categories were used to assign numerical values for the labs and vital signs (lines 874-1058 in the R code provided in Supplementary Information SI-3). The laboratory results and vitals signs were then defined to be categorical variables based on their ranges as ‘very low’, ‘low’, ‘normal’, ‘high’, and/or ‘very high’. Not available (NA) was included as a category for laboratory results and vital signs. The ‘normal’ range was used as the reference category and hence not included in the input matrix. For example, pulse pressure values were computed by subtracting diastolic from systolic pressures, then encoding them into four categories (< 30, from 50 to 70,>70 mm Hg, or NA if no blood pressures are reported in the indicated time interval). Pulse pressures between 30 and 50 mm Hg were not included, and used as a reference category in the logistic regression. Labs and vital signs were first carried forward (imputed) over time when missing, and if needed, values were then carried (imputed) backward. This approach resulted in the lab or vital signs being assigned ’NA’ only in the case of patients having no measurements across the entire 7.5 years of the prediction window.

#### Census data

The 2019 five-year aggregation of the American Community Survey was downloaded from the United States Census website^24^ and matched to US zip codes downloaded from the US Housing and Urban Development website^60^ and derived by the method described in reference^61^. Patient zip codes are requested at each outpatient visit to the VA and recorded in the outpatient workload table in the EMRs. The most frequently reported zip code for each of the eight time bins was matched to the ACS variable’s value in that zip code. Census variables are defined and normalized in line 801 of rv10.R, distributed in Supplementary Information SI-3.

### Prediction methodologies

Five different predictive modeling algorithms were used: logistic regression implemented in the glmnet package of R^62,63^ with LASSO^42^ penalty for model selection^64^, Cox proportional hazard model^65^, random forest^66^, a simple fully connected (vanilla) neural network with three hidden layers^67–69^, and a transformer-based neural network called TabNet^70^ to predict the occurrence of the combined outcome, which included death by suicide, suicide attempt, or drug overdose. Using 7.5 years of longitudinal EMR data for each patient, we applied these methods to the four study designs (Rcc, Psych-ds, Visit-ds, and C15/17 cohorts) discussed above.

For random forest, we used 500 trees, the square root of the number of input features for the number of features to consider for the best split, and required a minimum of 100 samples at a leaf node. The fully connected neural network had 3 hidden layers with 1024, 512, and 256 neurons, respectively, and ReLu nonlinearity^71^. The Tabnet algorithm used the PyTorch implementation^72^ with the default settings for the hyperparameters. These included the width of the decision prediction layer (8), the width of the attention embedding (8), the number of steps in the architecture (3), and gamma (1.3).

### Fine tuning and ensemble transfer learning

To test the robustness and generalizability of the methods, we tested models trained on one study cohort to cross-predict on another cohort. For example, we used the logistic regression coefficients fitted on the Rcc study design to predict outcomes in all the prospective cohorts and calculated the corresponding c-statistics. We did this across all the prediction algorithms and study cohorts. Results of this analysis are presented in Supplementary Table S1. We then performed transfer learning^32,33,73,74^ on a selected subset of these models (mostly trained on Rcc) for making use of the knowledge learned from one study cohort to facilitate learning in a different cohort. This is done by fine-tuning the models trained on Rcc or C15 cohorts on the C17 cohort to predict either suicide or the combined outcome, as described in Fig. 3. In keeping with the goals of fine-tuning, only a minimal number of variables were included, and these were selected to be ones expected to change with study design (eg matched variables or components closely related to outcome definition). Such variables will be well away from the diagonal in Fig. 2. We found these variables to include gender, race, ethnicity, young or old, and recorded presence of chronic pain, suicide ideation, suicide attempt, and overdose during the 7.5 years of the observation period. The number of degrees of freedom fine-tuned was further restricted by aggregating the presence/absence across time bins such that presence in any time period counted as present. Additionally, we created one additional variable not directly used in the base models, to describe the aggregate level of healthcare utilization, which we constructed by summing across al 75 diagnosis-defined variables across all eight time bins, for a possible range of zero to 75 x 8 = 600. In practice this number rarely exceeds 100, so we scaled the coefficient presented at the far right in Fig. 3 by 100 and labeled it ’100 x usage’.

Next, we used an ensemble transfer learning approach^37^ to obtain our best model in terms of predictive performance and generalizability for our C17 cohort, with one model predicting suicide (EnsNDI) and another predicting our combined outcome (EnsAll). We used an ensemble of seven base models including the four models (logistic regression, random forest, neural network, and TabNet) trained on Rcc cohort, two Cox models trained on Psych-ds and Visit-ds cohorts, and one logistic regression model trained on the C17 cohort. This ensemble of base models was fine-tuned as described in Fig. 3. The code for performing the ensemble transfer learning appears from line 1340 to line 1425. Trained models are used to score the patients in C17 and the ten variables are created for fine-tuning by aggregating across all eight time bins. Even and odd cohorts are fit without model selection and resulting scores are computed on the half of C17 not trained on. These scores can then be used for model evaluation for EnsAll and EnsNDI models. This portion of the code also implements the C17 predictions for C15 and C17-trained models, again taking care to do all the training on even (or odd) cohorts so that reported calibration and discrimination results are on test sets of patients.

### Training and testing data sets

All data sets used for training and evaluating base models and fine-tuning across a multitude of study designs, prediction methodologies, and outcomes were based on a separation of patients into 100 cohorts in order of date of birth, allowing us to use even and odd cohorts to separate test data from training data. For two-stage training, both trainings occurred on the same set (eg. even) of patients, leaving the other set (eg. odd) for evaluation. For all models except those reported in Fig. 7c, reported accuracy scores are the average of those trained on even cohorts and evaluated on odd and vice-versa. For linear models (logistic regression and Cox base models and all fine-tuned models, reported coefficients are the average of even- and odd-trained models.

For Fig. 7c, we used a holdout set of NDI outcomes from Jan 1, 2019–Jan 1, 2021 to assess model drift across two years of suicide reporting. In this instance, we created our best-possible models of C17 suicide risk, with outcomes of suicides through the end of 2018 and combined outcomes through the end of 2021, shown in the top of Fig. 1. NDI-specific models were trained on two-year suicide risk during 2017 and 2018. We then examined the drift in performance of a dozen models in predicting the holdout NDI suicides occurring from Jan 1, 2019–Jan 1, 2021, under two sets of assumptions. In the first instance, we fine-tuned each of the models on a test set of the NDI suicide data, while in the second instance, we fine-tuned the models on two-year 2017 NDI suicides, using these coefficients in predicting 2019 suicides. This provided an independent evaluation of model generalizability, with the two years of model drift allowing more independence of training and test data than the separation into even and odd date of birth cohorts.

### Evaluation of model performance

Beyond such metrics as c-statistics and various accuracy metrics, there are no standard metrics for visualizing predictive model performance for medical outcomes analysis. We have presented a variety of figures in this work that we found useful in evaluating and iteratively improving many aspects of our predictive models. In the sections above we have described each aspect of the creation of predictive models in relatively structured blocks of code. For the various calibration plots, histograms, and scatter plots that we made, much of the code is straightforward joining and subgrouping operations using the data table package^75^, and efforts were not made to modularize the code. Nevertheless, the reader may find it valuable to see the code fragments used in our analysis.

Interval predictions were evaluated in part using c-statistics, which is the area under the Receiver Operating Characteristic Curve (AUROC) or the area under the precision-recall curve. These areas were either integrated directly (lines 1321–1337 of Supplementary Information SI-3) or computed with the ROCR R package^76^. The goodness of fit for the Cox models is estimated by computing Harrell’s c-index^77^. The c-index gives the probability that a randomly selected patient who experienced an event had a higher risk score than a patient who had not experienced an event.

The calibration plots and case score histograms for various subgroups can be found in lines 1560–1776, using subgroup variables created on lines 1777–1839. This section of code reads in scores from each of the predictive models that we evaluated, together with the predictor variables that were used to define subgroups of patients. The code then splits cases and controls into histograms based on scores within each subgroup, computes probabilities of cases, and plots the calibration curves. Linear and logarithmic histograms of score histograms, subgrouped by the components of the outcome, were made in a similar manner as the calibration curves, as described in lines 1514–1542.

Age histograms, comparisons of coefficients, and comparisons of relative risk for sub-outcomes are encoded in lines 1839–1906.

### Ethics declarations

All analyses were carried out in accordance with the relevant guidelines and regulations.

### Consent to participate

Department of Veterans Affairs Central Institutional Review Board (IRB) approved this research project MVP011 using a waiver of informed consent and waiver of HIPAA authorization. No direct contact with research participants was made.

### Supplementary Information

Supplementary Information 1.

Supplementary Information 2.

Supplementary Information 3.

Supplementary Information 4.

Supplementary Information 5.

Supplementary Information 6.

## Data Availability

The data is owned by the Veterans Health Administration and contains patient-level medical data that are highly sensitive and protected by multiple regulations. The data cannot be made available to others without an inter-agency data sharing agreement between the requestor’s agency and the VA. Please contact Benjamin H.McMahon (mcmahon@lanl.gov) for further questions on the data availability.
