# Supplementary material for: High dimensional predictions of suicide risk in 4.2 million US Veterans using ensemble transfer learning
Source: Sci Rep. 2024 Jan 20;14:1793. doi: 10.1038/s41598-024-51762-9 (PMC10799879; doi:10.1038/s41598-024-51762-9)
Supplement: Supplementary file 1 — Supplementary Information 1. [file 41598_2024_51762_MOESM1_ESM.pdf]

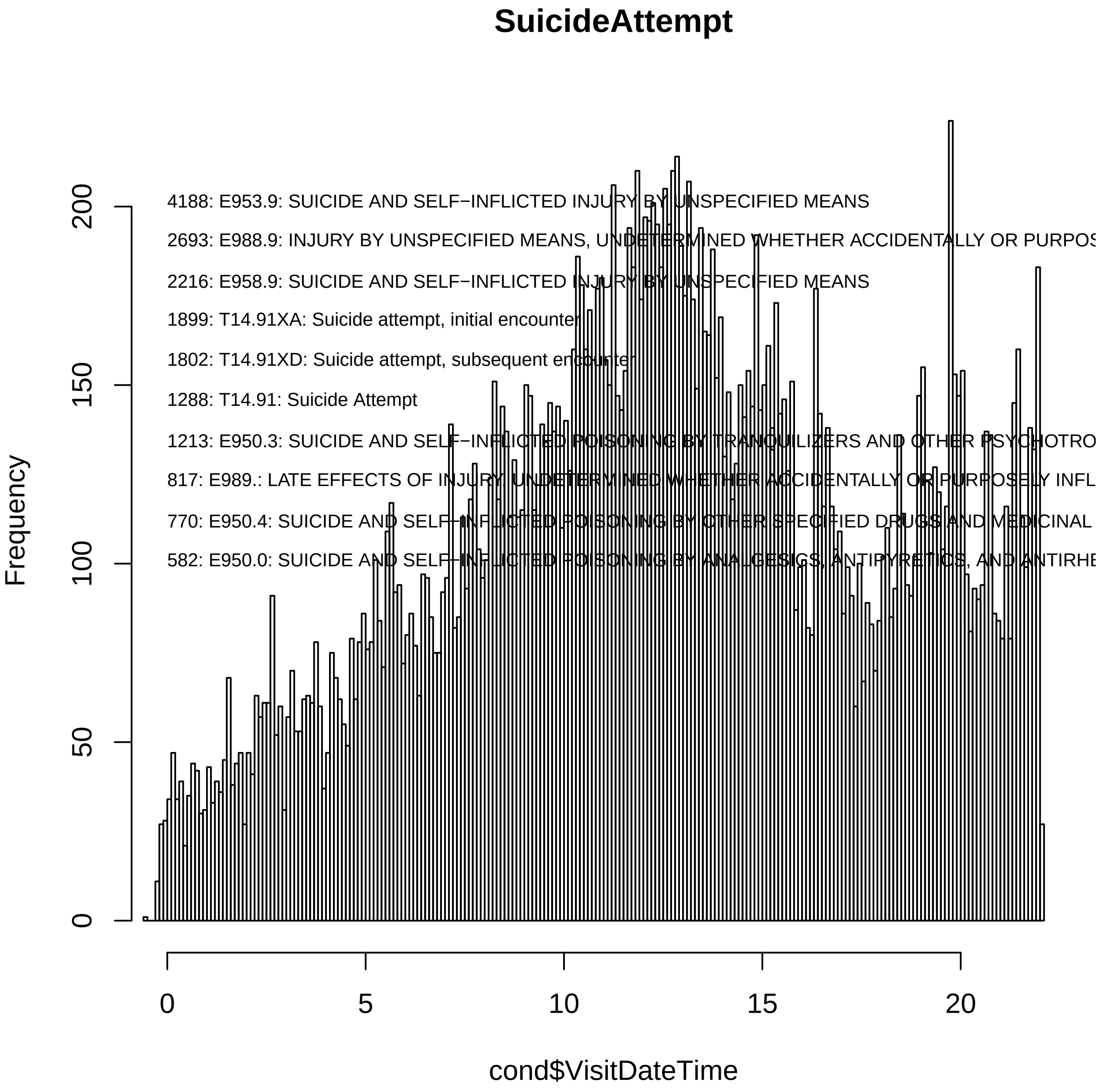
SuicideAttempt
4188: E953.9: SUICIDE AND SELF−INFLICTED INJURY BY UNSPECIFIED MEANS
2693: E988.9: INJURY BY UNSPECIFIED MEANS, UNDETERMINED WHETHER ACCIDENTALLY OR PURPOS
2216: E958.9: SUICIDE AND SELF−INFLICTED INJURY BY UNSPECIFIED MEANS
1899: T14.91XA: Suicide attempt, initial encounter
1802: T14.91XD: Suicide attempt, subsequent encounter
1288: T14.91: Suicide Attempt
1213: E950.3: SUICIDE AND SELF−INFLICTED POISONING BY TRANQUILIZERS AND OTHER PSYCHOTRO
817: E989.: LATE EFFECTS OF INJURY, UNDETERMINED WHETHER ACCIDENTALLY OR PURPOSELY INFL
770: E950.4: SUICIDE AND SELF−INFLICTED POISONING BY OTHER SPECIFIED DRUGS AND MEDICINAL
582: E950.0: SUICIDE AND SELF−INFLICTED POISONING BY ANALGESICS, ANTIPYRETICS, AND ANTIRHE
Frequency
0
50
100
150
200
0
5
10
15
20
cond$VisitDateTime

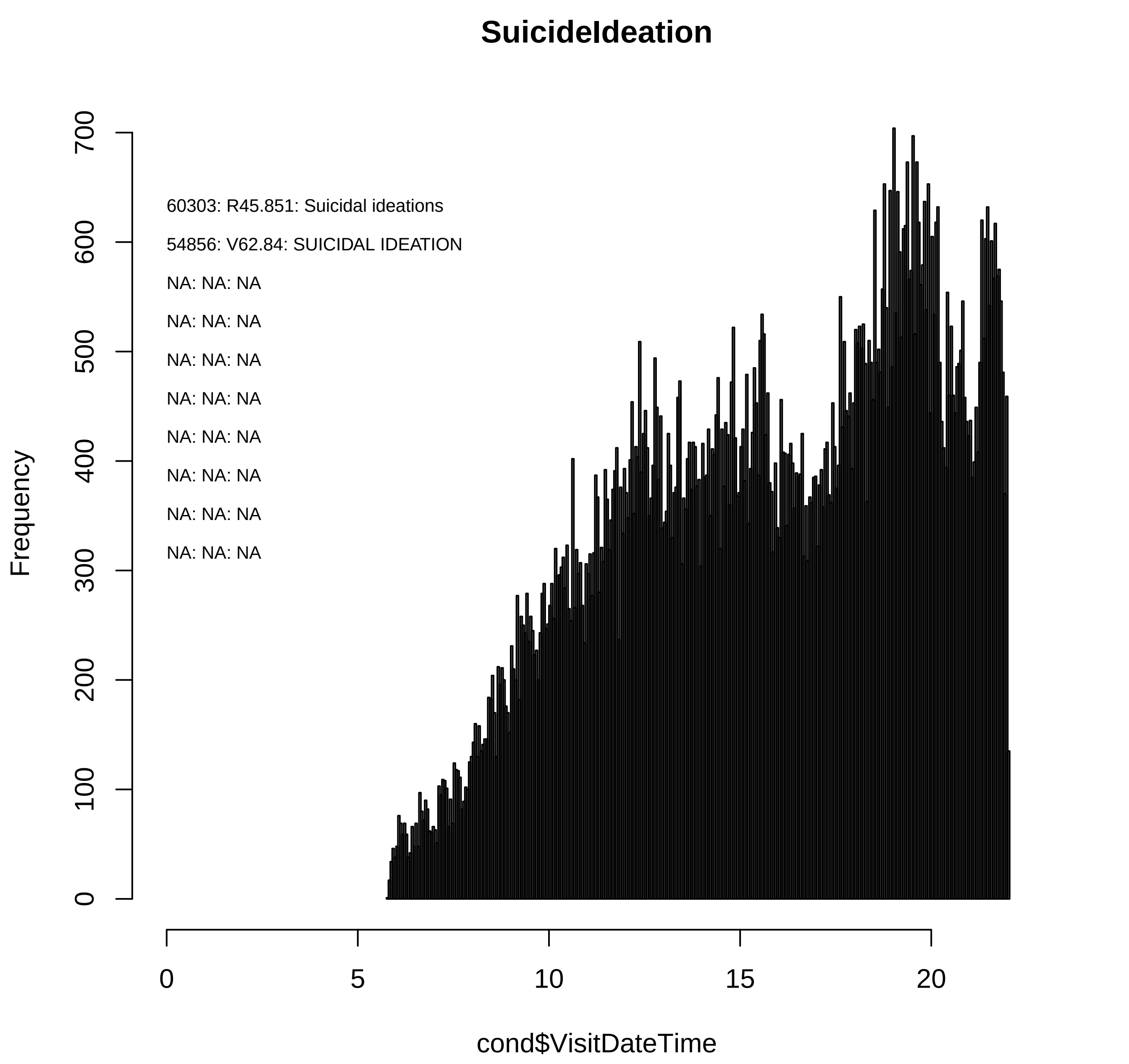
SuicideIdeation
60303: R45.851: Suicidal ideations
54856: V62.84: SUICIDAL IDEATION
NA: NA: NA
NA: NA: NA
NA: NA: NA
NA: NA: NA
NA: NA: NA
NA: NA: NA
NA: NA: NA
NA: NA: NA
Frequency
0
100
200
300
400
500
600
700
0
5
10
15
20
cond$VisitDateTime

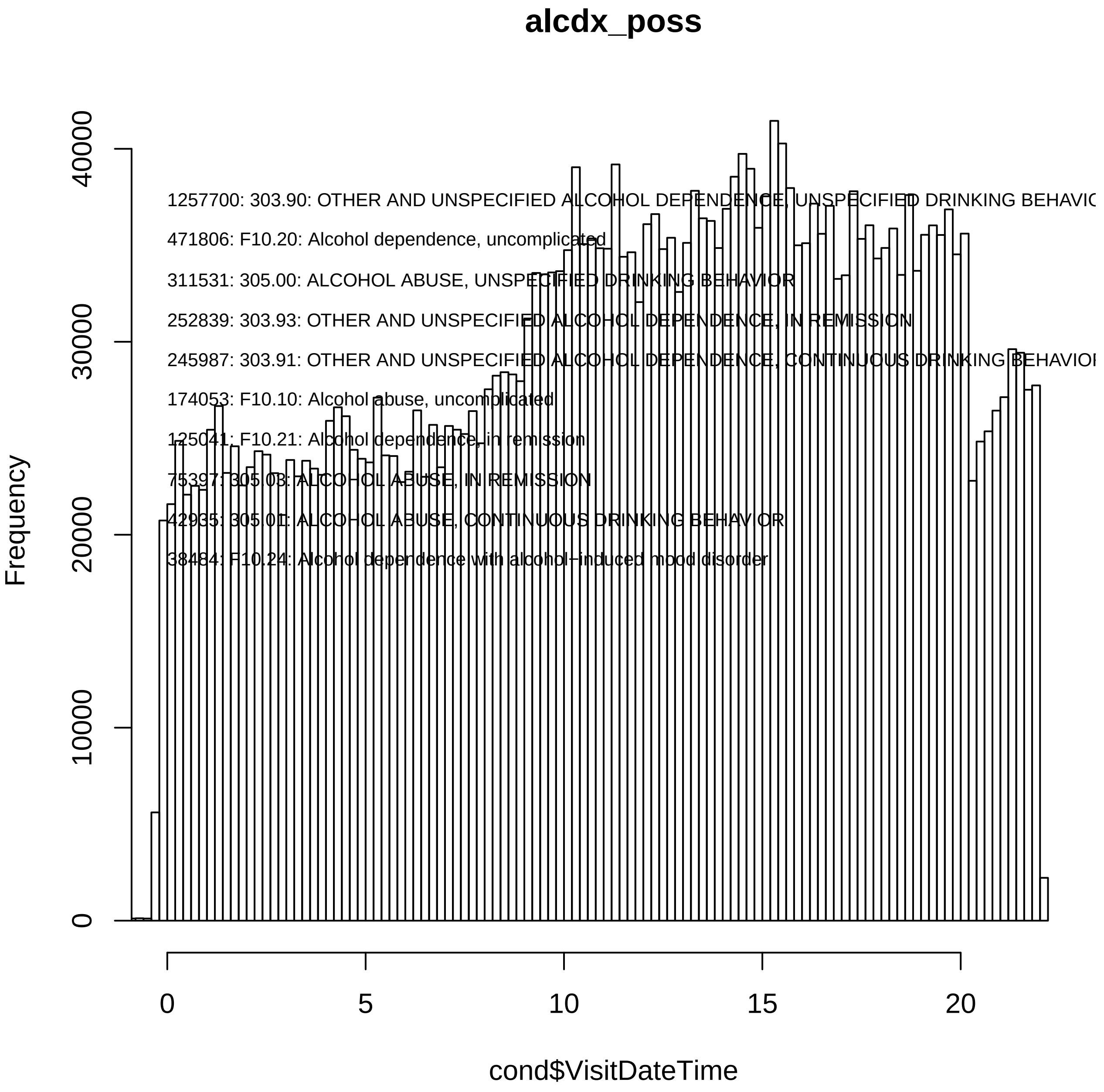
alcdx_poss
Frequency
0
10000
20000
30000
40000
1257700: 303.90: OTHER AND UNSPECIFIED ALCOHOL DEPENDENCE, UNSPECIFIED DRINKING BEHAVIO
471806: F10.20: Alcohol dependence, uncomplicated
311531: 305.00: ALCOHOL ABUSE, UNSPECIFIED DRINKING BEHAVIOR
252839: 303.93: OTHER AND UNSPECIFIED ALCOHOL DEPENDENCE, IN REMISSION
245987: 303.91: OTHER AND UNSPECIFIED ALCOHOL DEPENDENCE, CONTINUOUS DRINKING BEHAVIOI
174053: F10.10: Alcohol abuse, uncomplicated
125041: F10.21: Alcohol dependence, in remission
75397: 305.03: ALCOHOL ABUSE, IN REMISSION
42935: 305.01: ALCOHOL ABUSE, CONTINUOUS DRINKING BEHAVIOR
38484: F10.24: Alcohol dependence with alcohol−induced mood disorder
0
5
10
15
20
cond$VisitDateTime

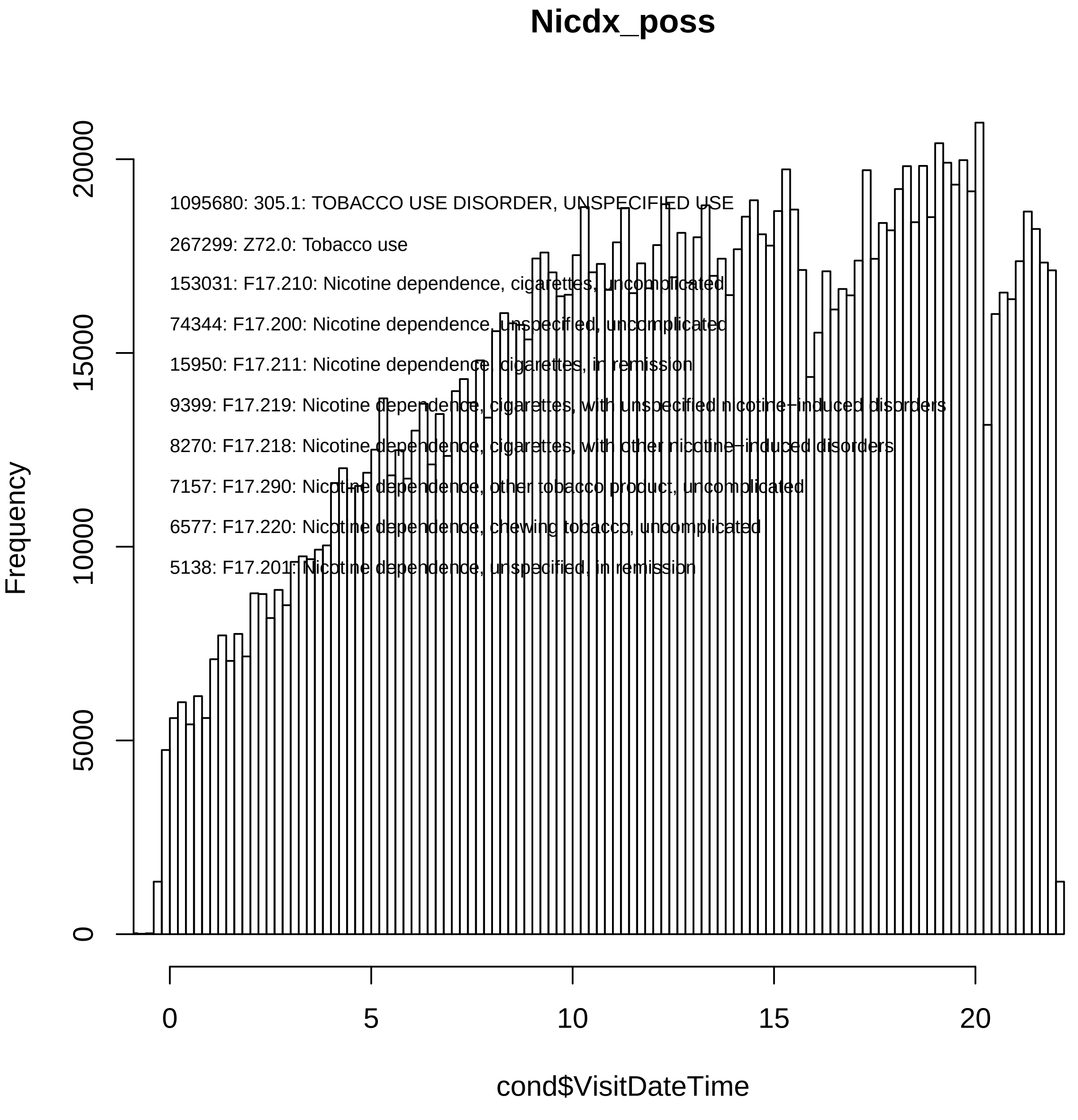

Nicdx_poss
1095680: 305.1: TOBACCO USE DISORDER, UNSPECIFIED USE
267299: Z72.0: Tobacco use
153031: F17.210: Nicotine dependence, cigarettes, uncomplicated
74344: F17.200: Nicotine dependence, unspecified, uncomplicated
15950: F17.211: Nicotine dependence, cigarettes, in remission
9399: F17.219: Nicotine dependence, cigarettes, with unspecified nicotine−induced disorders
8270: F17.218: Nicotine dependence, cigarettes, with other nicotine−induced disorders
7157: F17.290: Nicotine dependence, other tobacco product, uncomplicated
6577: F17.220: Nicotine dependence, chewing tobacco, uncomplicated
5138: F17.201: Nicotine dependence, unspecified, in remission
Frequency
0
5000
10000
15000
20000
0
5
10
15
20
cond$VisitDateTime

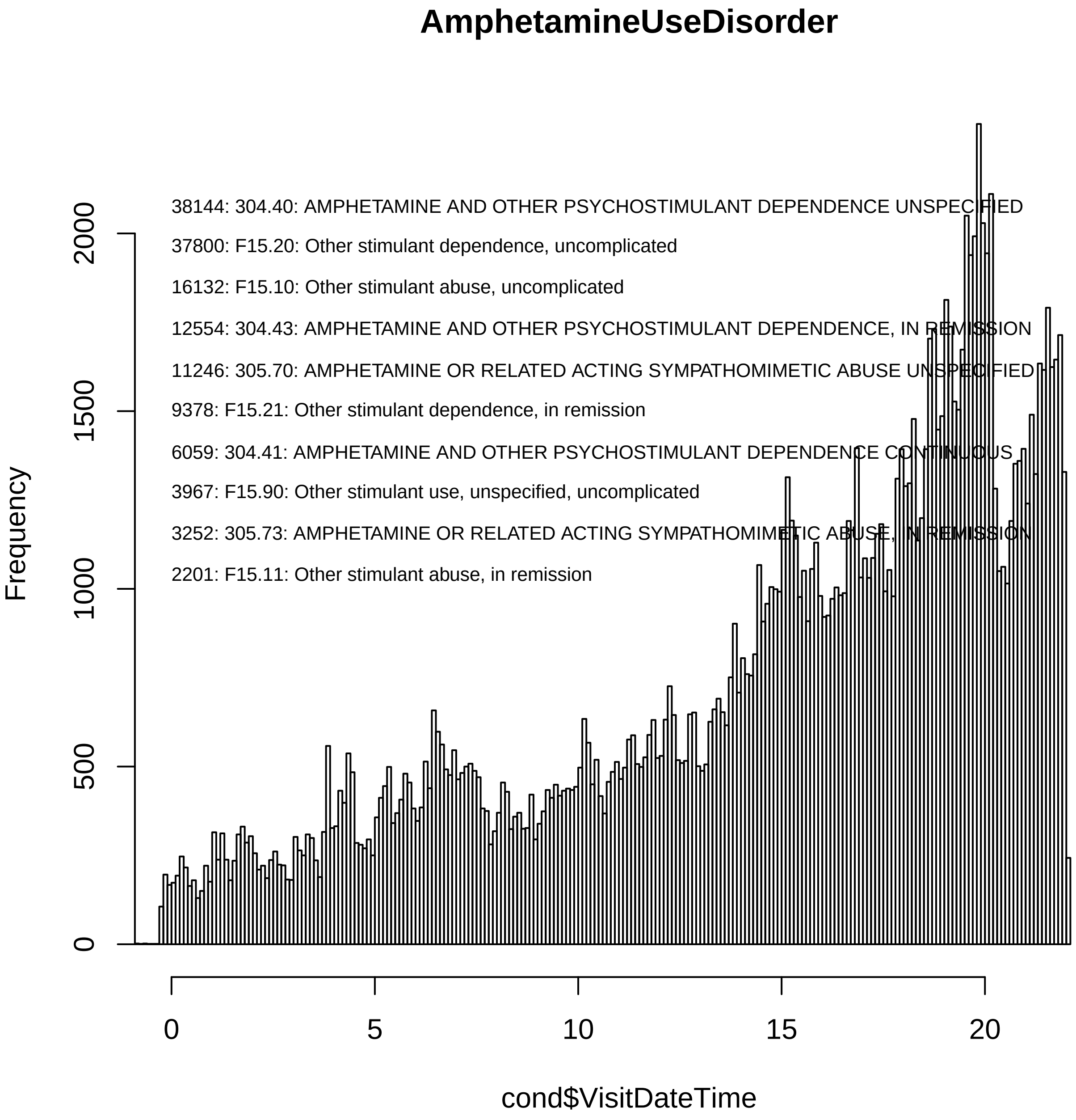
AmphetamineUseDisorder
38144: 304.40: AMPHETAMINE AND OTHER PSYCHOSTIMULANT DEPENDENCE UNSPECIFIED
37800: F15.20: Other stimulant dependence, uncomplicated
16132: F15.10: Other stimulant abuse, uncomplicated
12554: 304.43: AMPHETAMINE AND OTHER PSYCHOSTIMULANT DEPENDENCE, IN REMISSION
11246: 305.70: AMPHETAMINE OR RELATED ACTING SYMPATHOMIMETIC ABUSE UNSPECIFIED
9378: F15.21: Other stimulant dependence, in remission
6059: 304.41: AMPHETAMINE AND OTHER PSYCHOSTIMULANT DEPENDENCE CONTINUOUS
3967: F15.90: Other stimulant use, unspecified, uncomplicated
3252: 305.73: AMPHETAMINE OR RELATED ACTING SYMPATHOMIMETIC ABUSE, IN REMISSION
2201: F15.11: Other stimulant abuse, in remission
Frequency
0
500
1000
1500
2000
0
5
10
15
20
cond$VisitDateTime

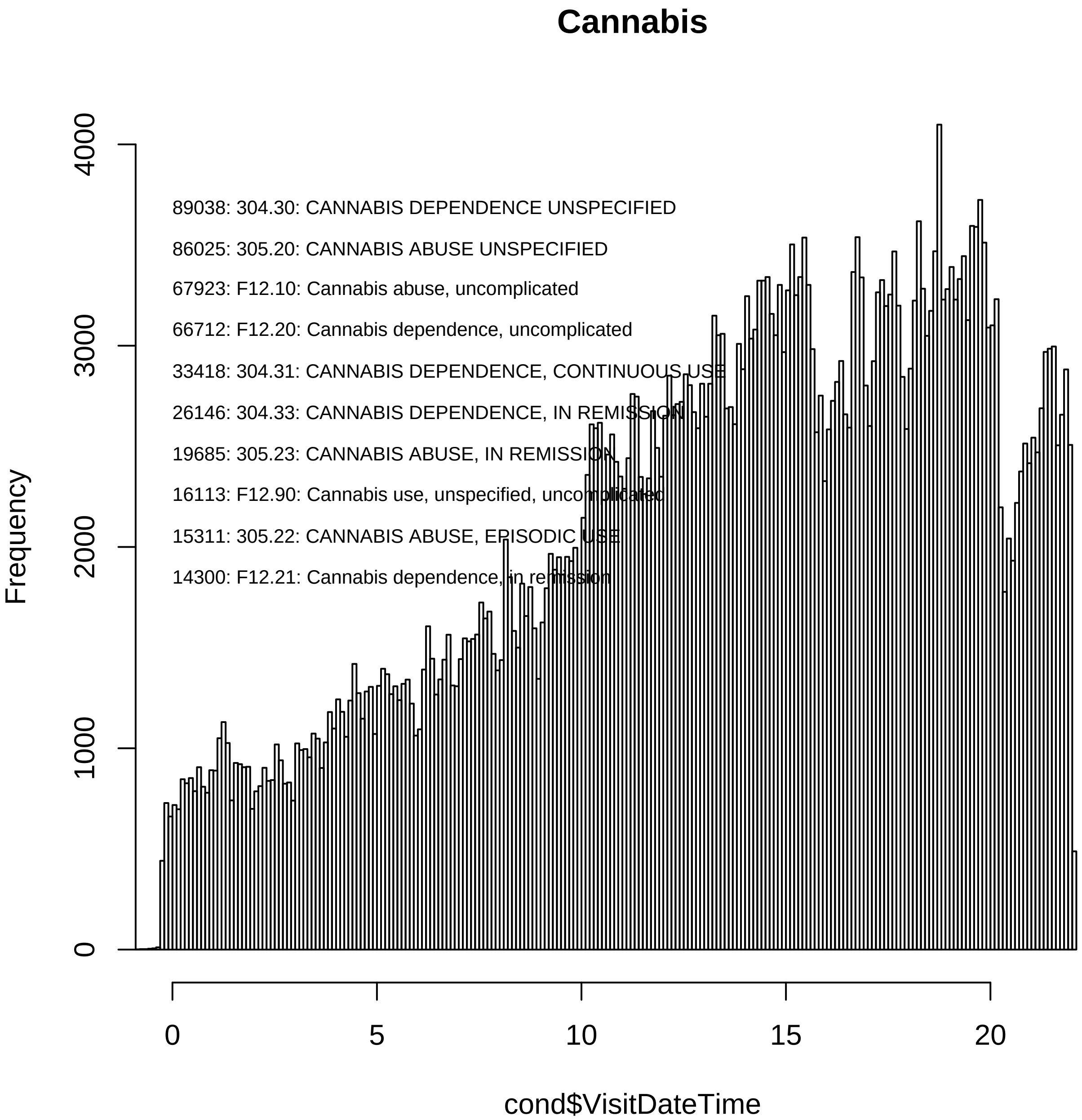

Cannabis
89038: 304.30: CANNABIS DEPENDENCE UNSPECIFIED
86025: 305.20: CANNABIS ABUSE UNSPECIFIED
67923: F12.10: Cannabis abuse, uncomplicated
66712: F12.20: Cannabis dependence, uncomplicated
33418: 304.31: CANNABIS DEPENDENCE, CONTINUOUS USE
26146: 304.33: CANNABIS DEPENDENCE, IN REMISSION
19685: 305.23: CANNABIS ABUSE, IN REMISSION
16113: F12.90: Cannabis use, unspecified, uncomplicated
15311: 305.22: CANNABIS ABUSE, EPISODIC USE
14300: F12.21: Cannabis dependence, in remission
Frequency
0
1000
2000
3000
4000
0
5
10
15
20
cond$VisitDateTime

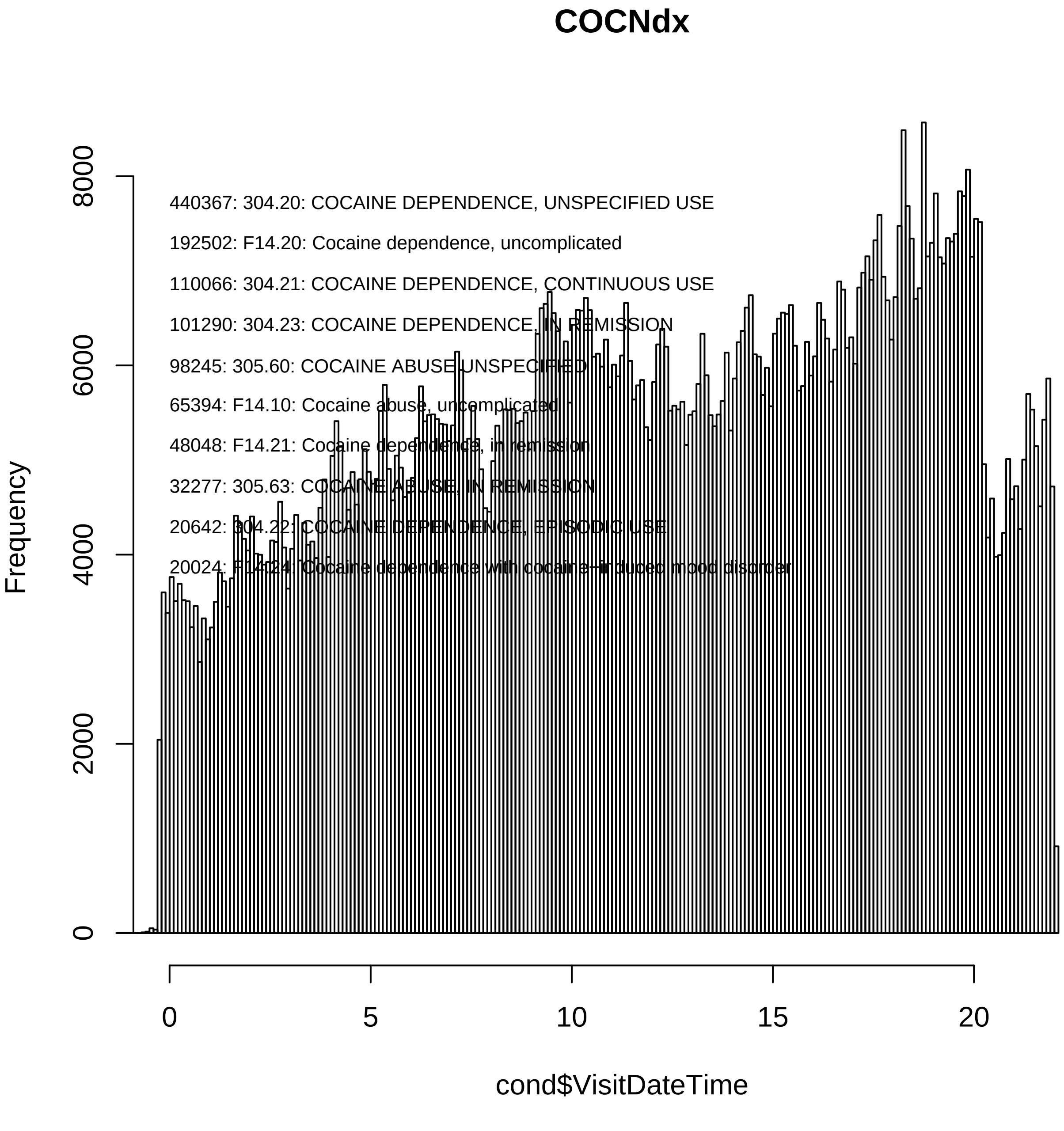
COCNdx
440367: 304.20: COCAINE DEPENDENCE, UNSPECIFIED USE
192502: F14.20: Cocaine dependence, uncomplicated
110066: 304.21: COCAINE DEPENDENCE, CONTINUOUS USE
101290: 304.23: COCAINE DEPENDENCE, IN REMISSION
98245: 305.60: COCAINE ABUSE UNSPECIFIED
65394: F14.10: Cocaine abuse, uncomplicated
48048: F14.21: Cocaine dependence, in remission
32277: 305.63: COCAINE ABUSE, IN REMISSION
20642: 304.22: COCAINE DEPENDENCE, EPISODIC USE
20024: F14.24: Cocaine dependence with cocaine-induced mood disorder
Frequency
0
2000
4000
6000
8000
0
5
10
15
20
cond$VisitDateTime

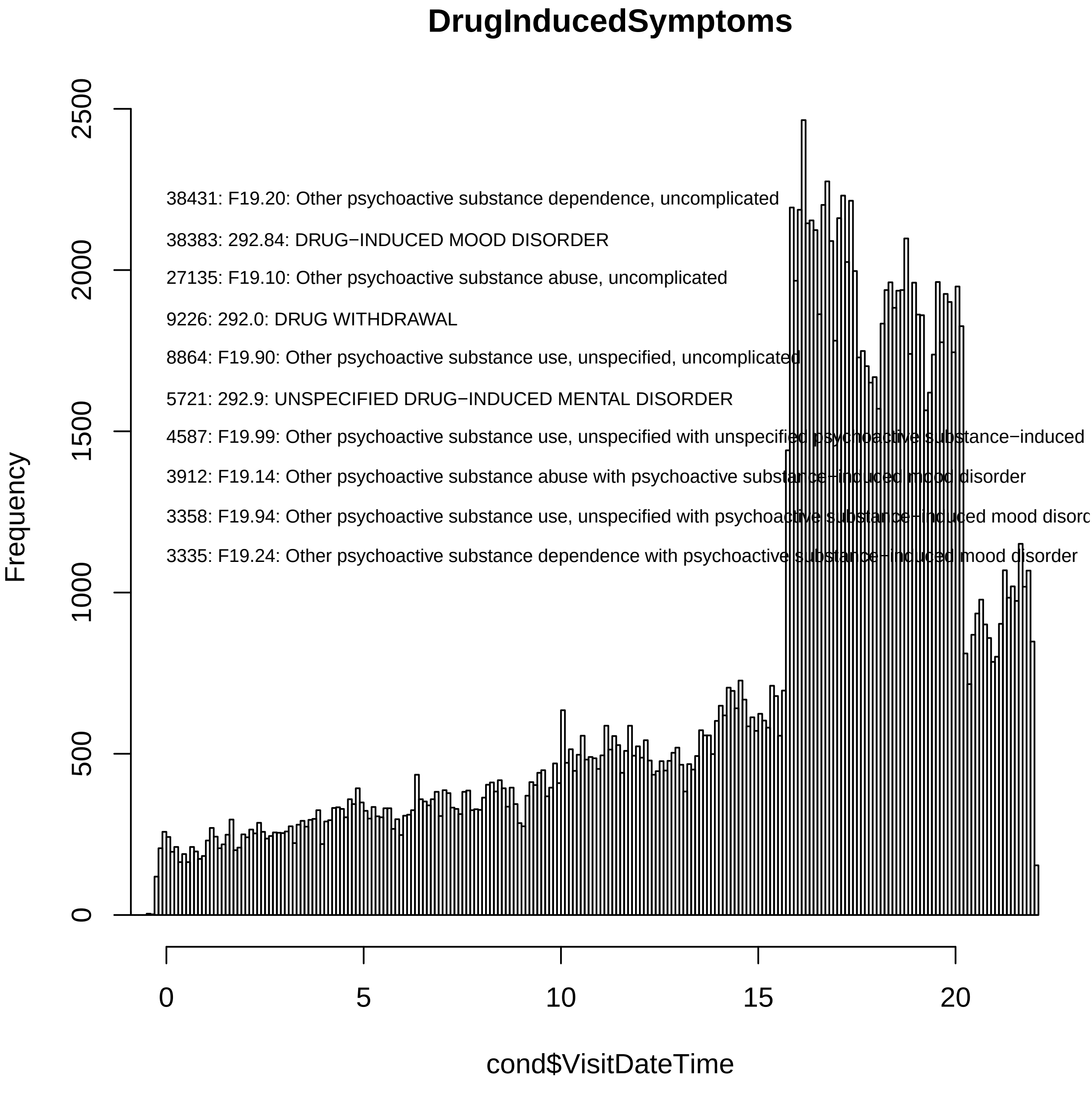
DrugInducedSymptoms
38431: F19.20: Other psychoactive substance dependence, uncomplicated
38383: 292.84: DRUG−INDUCED MOOD DISORDER
27135: F19.10: Other psychoactive substance abuse, uncomplicated
9226: 292.0: DRUG WITHDRAWAL
8864: F19.90: Other psychoactive substance use, unspecified, uncomplicated
5721: 292.9: UNSPECIFIED DRUG−INDUCED MENTAL DISORDER
4587: F19.99: Other psychoactive substance use, unspecified with unspecified psychoactive substance−induced
3912: F19.14: Other psychoactive substance abuse with psychoactive substance−induced mood disorder
3358: F19.94: Other psychoactive substance use, unspecified with psychoactive substance−induced mood disorc
3335: F19.24: Other psychoactive substance dependence with psychoactive substance−induced mood disorder
Frequency
0
500
1000
1500
2000
2500
0
5
10
15
20
cond$VisitDateTime

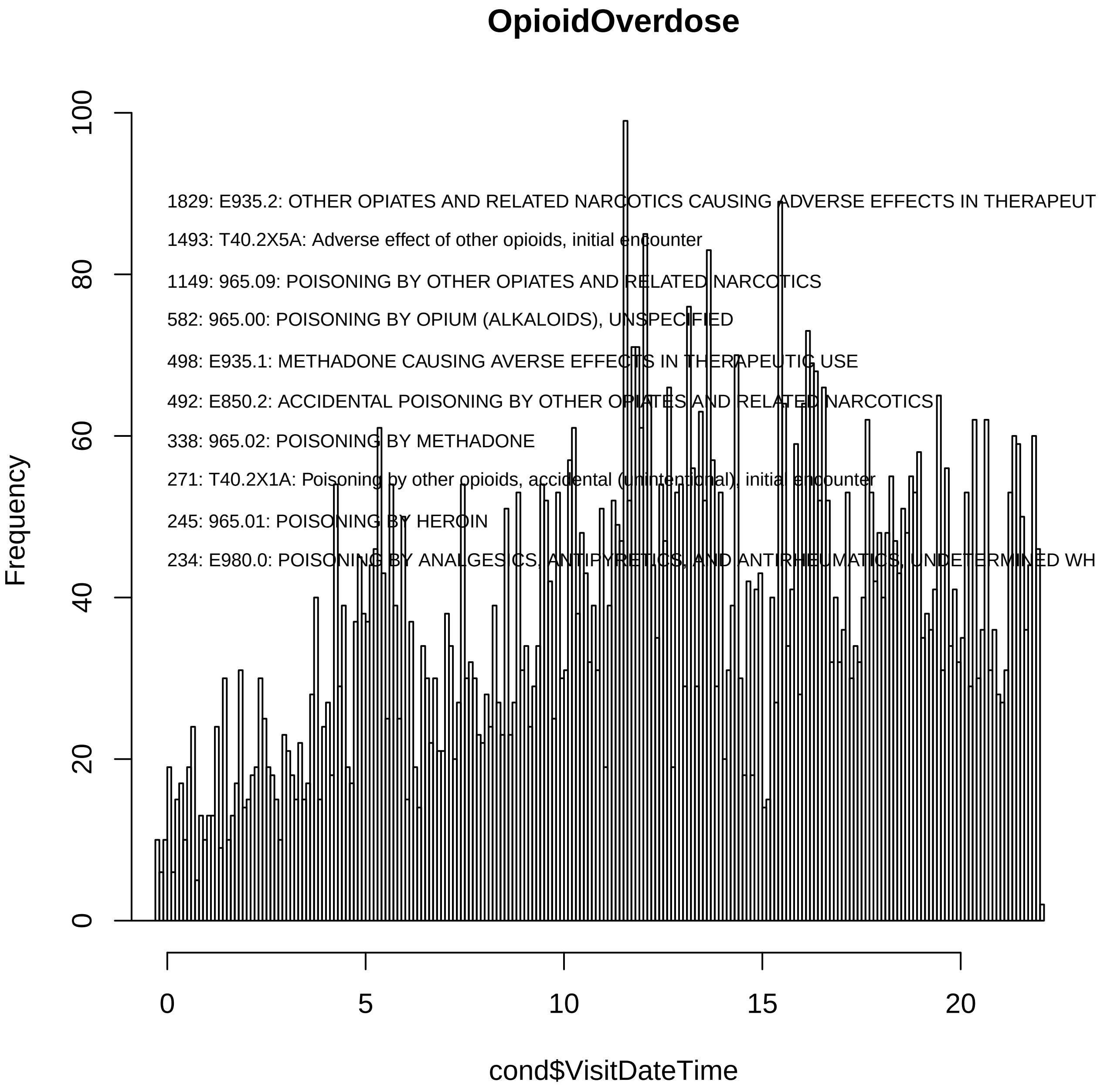
OpioidOverdose
1829: E935.2: OTHER OPIATES AND RELATED NARCOTICS CAUSING ADVERSE EFFECTS IN THERAPEUT
1493: T40.2X5A: Adverse effect of other opioids, initial encounter
1149: 965.09: POISONING BY OTHER OPIATES AND RELATED NARCOTICS
582: 965.00: POISONING BY OPIUM (ALKALOIDS), UNSPECIFIED
498: E935.1: METHADONE CAUSING AVERSE EFFECTS IN THERAPEUTIC USE
492: E850.2: ACCIDENTAL POISONING BY OTHER OPIATES AND RELATED NARCOTICS
338: 965.02: POISONING BY METHADONE
271: T40.2X1A: Poisoning by other opioids, accidental (unintentional), initial encounter
245: 965.01: POISONING BY HEROIN
234: E980.0: POISONING BY ANALGESICS, ANTIPYRETICS, AND ANTIRHEUMATICS, UNDETERMINED WH
Frequency
0
20
40
60
80
100
0
5
10
15
20
cond$VisitDateTime

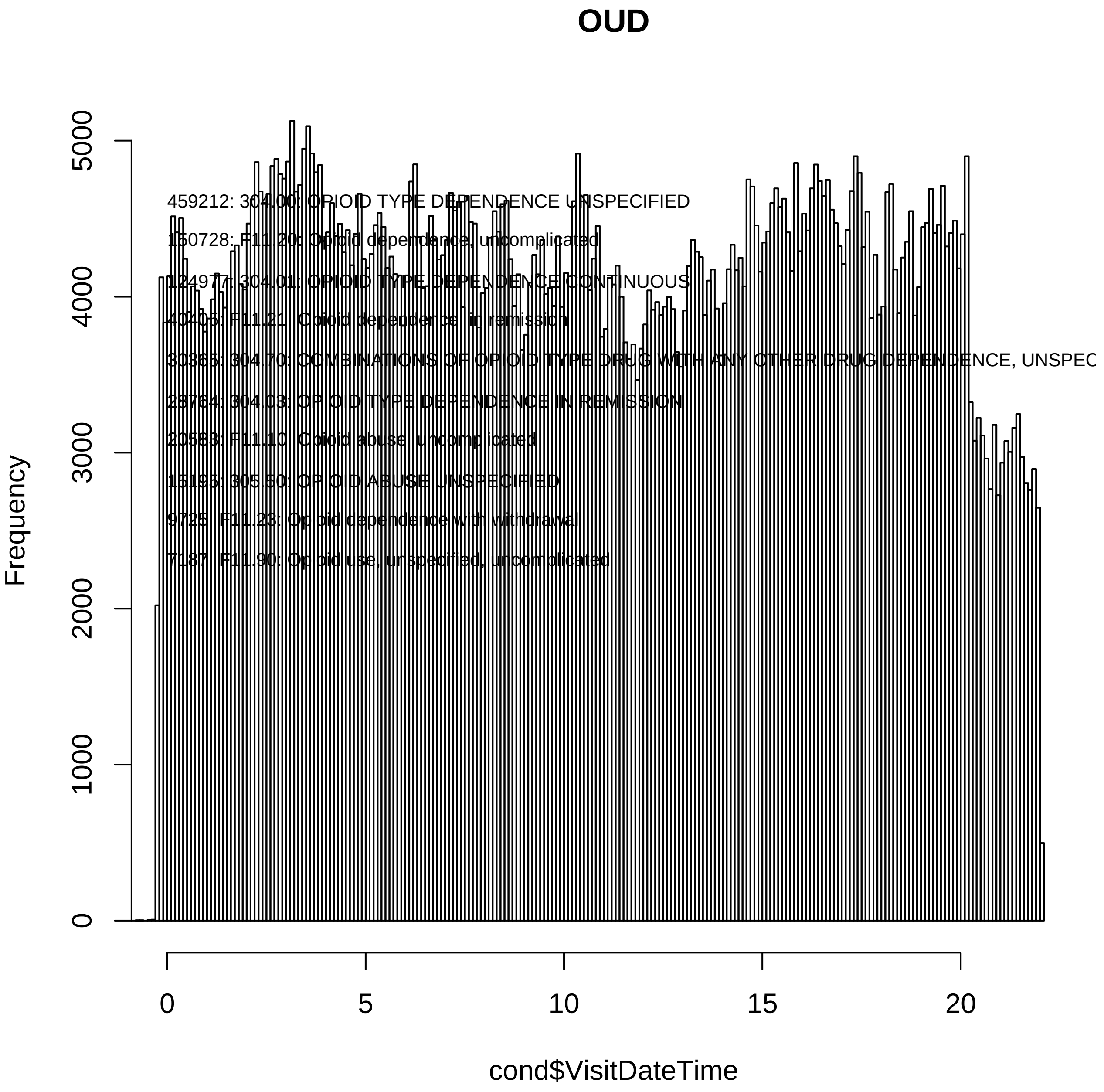
OUD
459212: 304.00: OPIOID TYPE DEPENDENCE UNSPECIFIED
150728: F11.20: Opioid dependence, uncomplicated
124977: 304.01: OPIOID TYPE DEPENDENCE CONTINUOUS
40405: F11.21: Opioid dependence, in remission
30365: 304.70: COMBINATIONS OF OPIOID TYPE DRUG WITH ANY OTHER DRUG DEPENDENCE, UNSPEC
23764: 304.03: OPIOID TYPE DEPENDENCE IN REMISSION
20583: F11.10: Opioid abuse, uncomplicated
15195: 305.50: OPIOID ABUSE UNSPECIFIED
9725: F11.23: Opioid dependence with withdrawal
7187: F11.90: Opioid use, unspecified, uncomplicated
Frequency
0
1000
2000
3000
4000
5000
0
5
10
15
20
cond$VisitDateTime

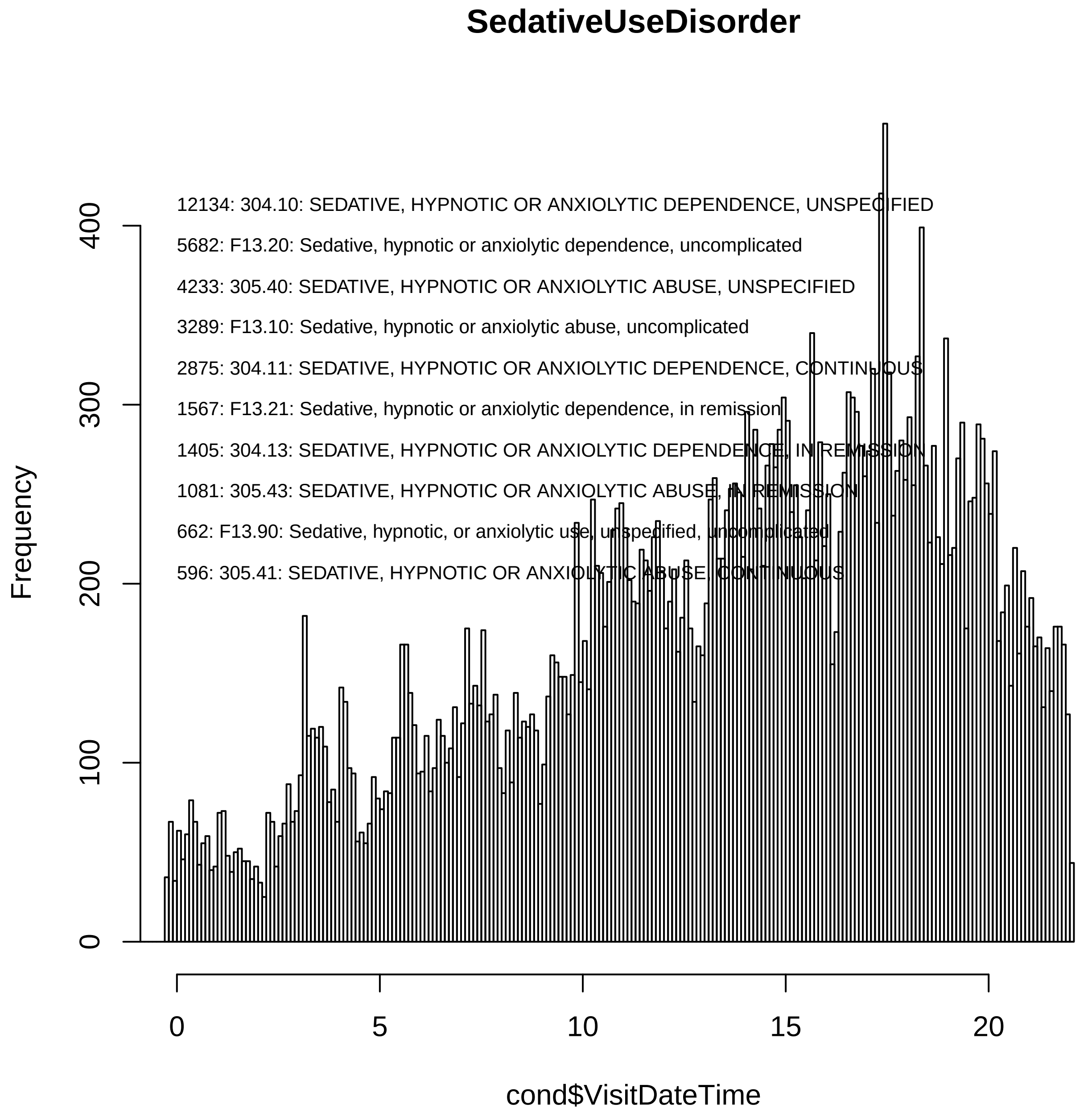
SedativeUseDisorder
12134: 304.10: SEDATIVE, HYPNOTIC OR ANXIOLYTIC DEPENDENCE, UNSPECIFIED
5682: F13.20: Sedative, hypnotic or anxiolytic dependence, uncomplicated
4233: 305.40: SEDATIVE, HYPNOTIC OR ANXIOLYTIC ABUSE, UNSPECIFIED
3289: F13.10: Sedative, hypnotic or anxiolytic abuse, uncomplicated
2875: 304.11: SEDATIVE, HYPNOTIC OR ANXIOLYTIC DEPENDENCE, CONTINUOUS
1567: F13.21: Sedative, hypnotic or anxiolytic dependence, in remission
1405: 304.13: SEDATIVE, HYPNOTIC OR ANXIOLYTIC DEPENDENCE, IN REMISSION
1081: 305.43: SEDATIVE, HYPNOTIC OR ANXIOLYTIC ABUSE, IN REMISSION
662: F13.90: Sedative, hypnotic, or anxiolytic use, unspecified, uncomplicated
596: 305.41: SEDATIVE, HYPNOTIC OR ANXIOLYTIC ABUSE, CONTINUOUS
Frequency
0
100
200
300
400
0
5
10
15
20
cond$VisitDateTime

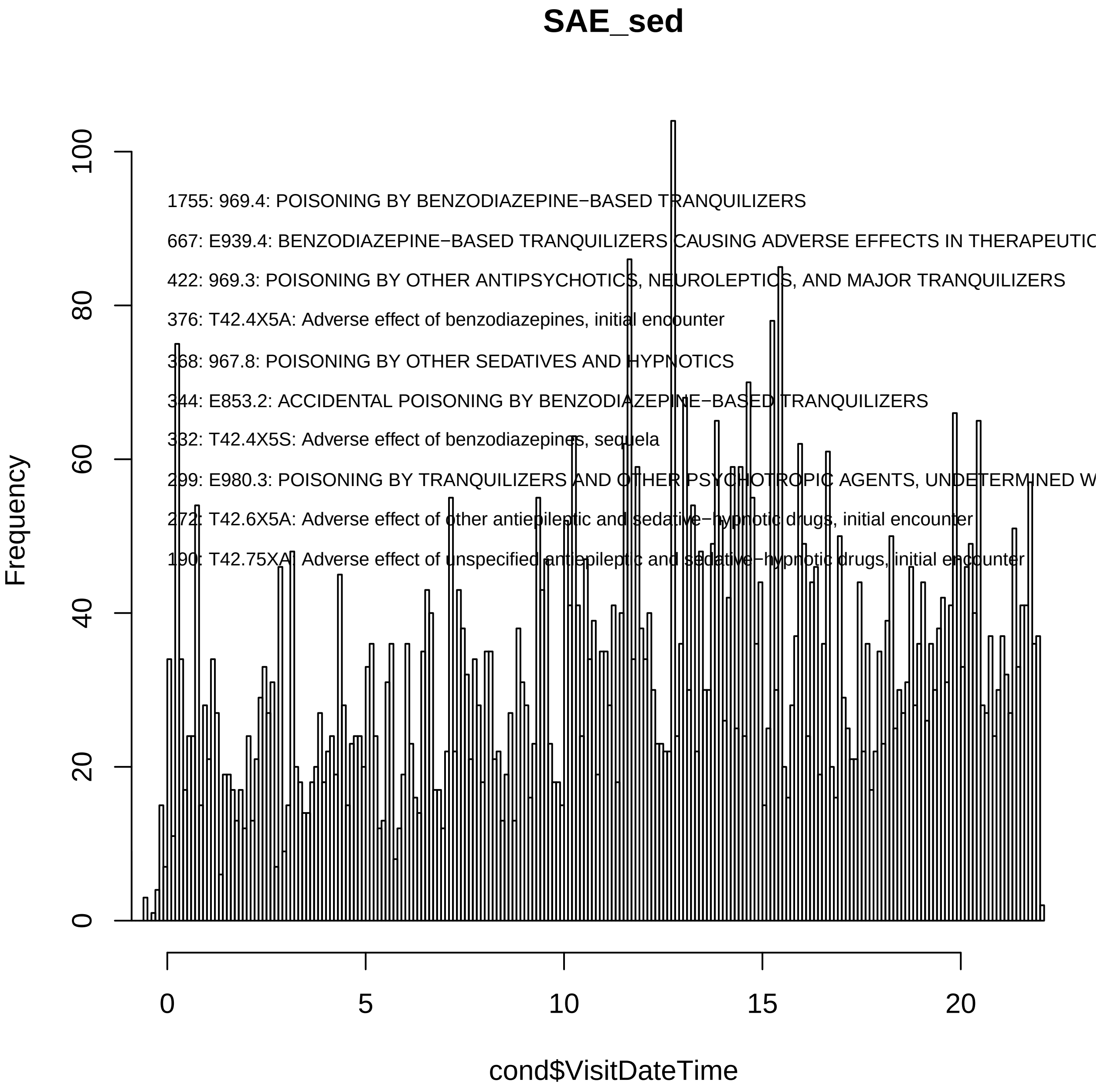
SAE_sed
1755: 969.4: POISONING BY BENZODIAZEPINE−BASED TRANQUILIZERS
667: E939.4: BENZODIAZEPINE−BASED TRANQUILIZERS CAUSING ADVERSE EFFECTS IN THERAPEUTIC
422: 969.3: POISONING BY OTHER ANTIPSYCHOTICS, NEUROLEPTICS, AND MAJOR TRANQUILIZERS
376: T42.4X5A: Adverse effect of benzodiazepines, initial encounter
368: 967.8: POISONING BY OTHER SEDATIVES AND HYPNOTICS
344: E853.2: ACCIDENTAL POISONING BY BENZODIAZEPINE−BASED TRANQUILIZERS
332: T42.4X5S: Adverse effect of benzodiazepines, sequela
299: E980.3: POISONING BY TRANQUILIZERS AND OTHER PSYCHOTROPIC AGENTS, UNDETERMINED W
272: T42.6X5A: Adverse effect of other antiepileptic and sedative−hypnotic drugs, initial encounter
190: T42.75XA: Adverse effect of unspecified antiepileptic and sedative−hypnotic drugs, initial encounter
Frequency
0
20
40
60
80
100
0
5
10
15
20
cond$VisitDateTime

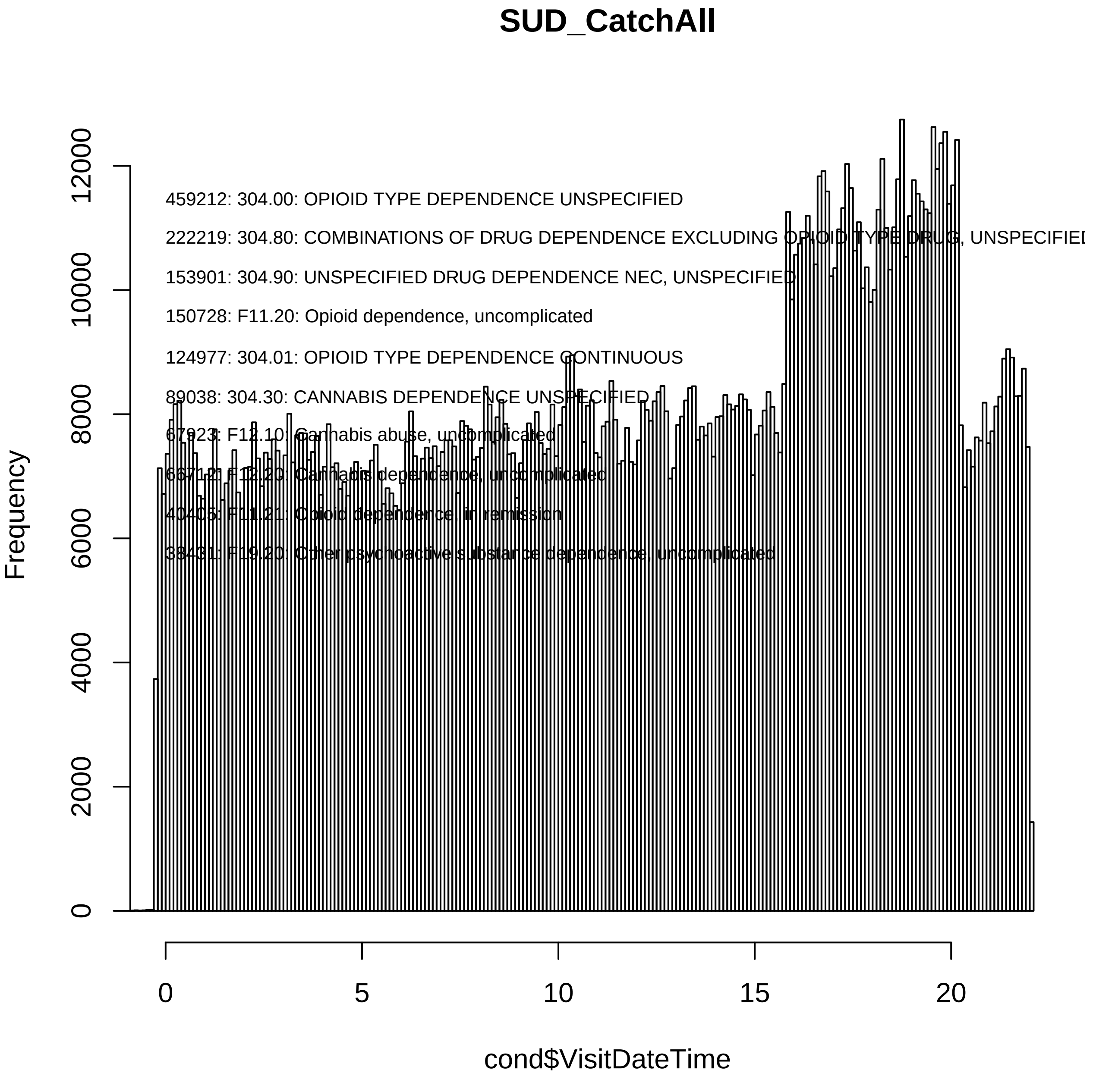
SUD_CatchAll
459212: 304.00: OPIOID TYPE DEPENDENCE UNSPECIFIED
222219: 304.80: COMBINATIONS OF DRUG DEPENDENCE EXCLUDING OPIOID TYPE DRUG, UNSPECIFIED
153901: 304.90: UNSPECIFIED DRUG DEPENDENCE NEC, UNSPECIFIED
150728: F11.20: Opioid dependence, uncomplicated
124977: 304.01: OPIOID TYPE DEPENDENCE CONTINUOUS
89038: 304.30: CANNABIS DEPENDENCE UNSPECIFIED
67923: F12.10: Cannabis abuse, uncomplicated
66712: F12.20: Cannabis dependence, uncomplicated
40405: F11.21: Opioid dependence, in remission
38431: F19.20: Other psychoactive substance dependence, uncomplicated
Frequency
0
2000
4000
6000
8000
10000
12000
0
5
10
15
20
cond$VisitDateTime

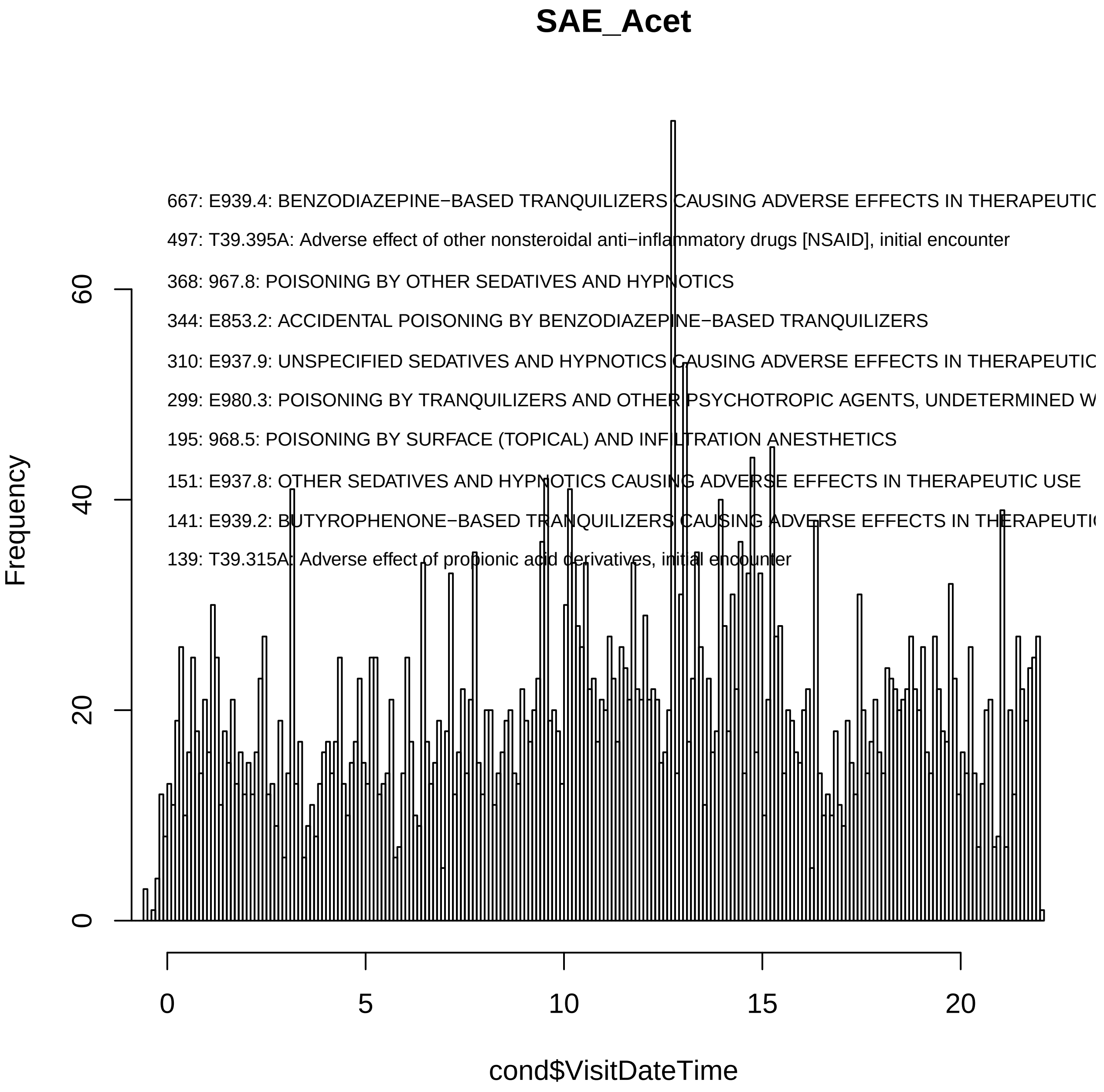

SAE_Acet
667: E939.4: BENZODIAZEPINE−BASED TRANQUILIZERS CAUSING ADVERSE EFFECTS IN THERAPEUTIC
497: T39.395A: Adverse effect of other nonsteroidal anti−inflammatory drugs [NSAID], initial encounter
368: 967.8: POISONING BY OTHER SEDATIVES AND HYPNOTICS
344: E853.2: ACCIDENTAL POISONING BY BENZODIAZEPINE−BASED TRANQUILIZERS
310: E937.9: UNSPECIFIED SEDATIVES AND HYPNOTICS CAUSING ADVERSE EFFECTS IN THERAPEUTIC
299: E980.3: POISONING BY TRANQUILIZERS AND OTHER PSYCHOTROPIC AGENTS, UNDETERMINED W
195: 968.5: POISONING BY SURFACE (TOPICAL) AND INFILTRATION ANESTHETICS
151: E937.8: OTHER SEDATIVES AND HYPNOTICS CAUSING ADVERSE EFFECTS IN THERAPEUTIC USE
141: E939.2: BUTYROPHENONE−BASED TRANQUILIZERS CAUSING ADVERSE EFFECTS IN THERAPEUTIC
139: T39.315A: Adverse effect of propionic acid derivatives, initial encounter
Frequency
0
20
40
60
0
5
10
15
20
cond$VisitDateTime

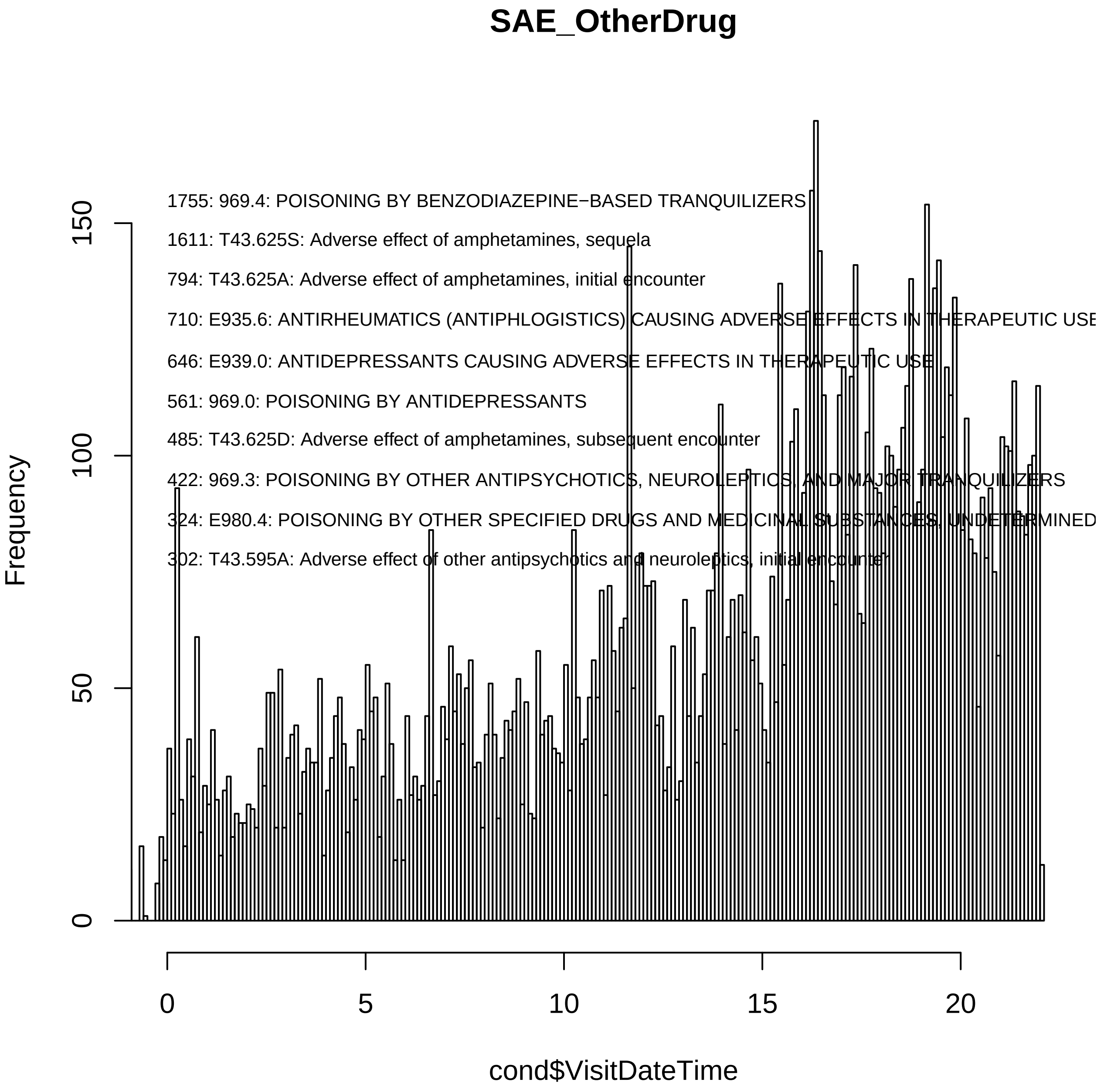
SAE_OtherDrug
1755: 969.4: POISONING BY BENZODIAZEPINE−BASED TRANQUILIZERS
1611: T43.625S: Adverse effect of amphetamines, sequela
794: T43.625A: Adverse effect of amphetamines, initial encounter
710: E935.6: ANTIRHEUMATICS (ANTIPHLOGISTICS) CAUSING ADVERSE EFFECTS IN THERAPEUTIC USE
646: E939.0: ANTIDEPRESSANTS CAUSING ADVERSE EFFECTS IN THERAPEUTIC USE
561: 969.0: POISONING BY ANTIDEPRESSANTS
485: T43.625D: Adverse effect of amphetamines, subsequent encounter
422: 969.3: POISONING BY OTHER ANTIPSYCHOTICS, NEUROLEPTICS, AND MAJOR TRANQUILIZERS
324: E980.4: POISONING BY OTHER SPECIFIED DRUGS AND MEDICINAL SUBSTANCES, UNDETERMINED
302: T43.595A: Adverse effect of other antipsychotics and neuroleptics, initial encounter
Frequency
0
50
100
150
0
5
10
15
20
cond$VisitDateTime

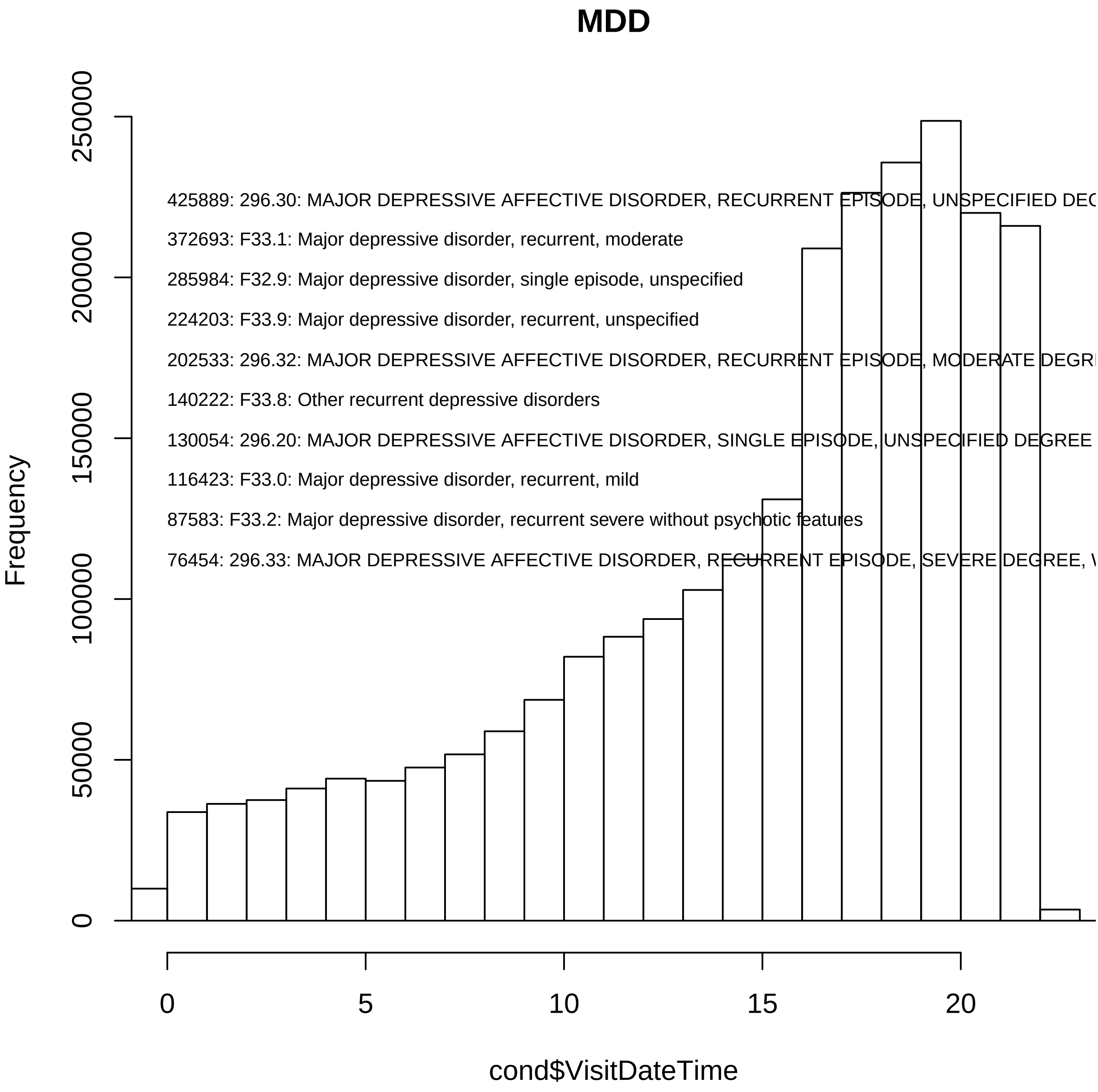

MDD
425889: 296.30: MAJOR DEPRESSIVE AFFECTIVE DISORDER, RECURRENT EPISODE, UNSPECIFIED DEG
372693: F33.1: Major depressive disorder, recurrent, moderate
285984: F32.9: Major depressive disorder, single episode, unspecified
224203: F33.9: Major depressive disorder, recurrent, unspecified
202533: 296.32: MAJOR DEPRESSIVE AFFECTIVE DISORDER, RECURRENT EPISODE, MODERATE DEGR
140222: F33.8: Other recurrent depressive disorders
130054: 296.20: MAJOR DEPRESSIVE AFFECTIVE DISORDER, SINGLE EPISODE, UNSPECIFIED DEGREE
116423: F33.0: Major depressive disorder, recurrent, mild
87583: F33.2: Major depressive disorder, recurrent severe without psychotic features
76454: 296.33: MAJOR DEPRESSIVE AFFECTIVE DISORDER, RECURRENT EPISODE, SEVERE DEGREE, W
Frequency
0
50000
100000
150000
200000
250000
0
5
10
15
20
cond$VisitDateTime

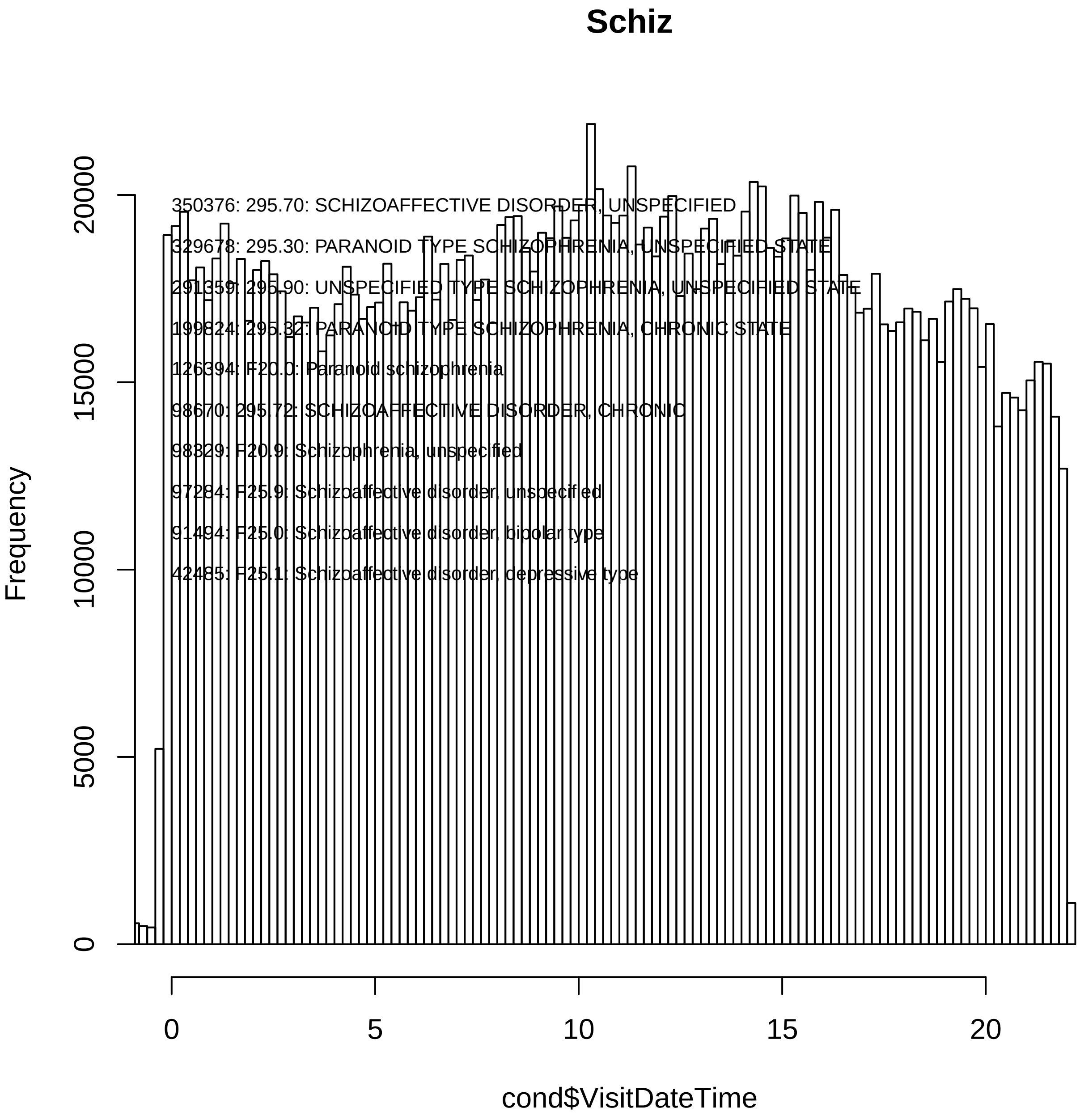
Schiz
350376: 295.70: SCHIZOAFFECTIVE DISORDER, UNSPECIFIED
329678: 295.30: PARANOID TYPE SCHIZOPHRENIA, UNSPECIFIED STATE
291359: 295.90: UNSPECIFIED TYPE SCHIZOPHRENIA, UNSPECIFIED STATE
199824: 295.32: PARANOID TYPE SCHIZOPHRENIA, CHRONIC STATE
126394: F20.0: Paranoid schizophrenia
98670: 295.72: SCHIZOAFFECTIVE DISORDER, CHRONIC
98329: F20.9: Schizophrenia, unspecified
97284: F25.9: Schizoaffective disorder, unspecified
91494: F25.0: Schizoaffective disorder, bipolar type
42485: F25.1: Schizoaffective disorder, depressive type
Frequency
0
5000
10000
15000
20000
0
5
10
15
20
cond$VisitDateTime

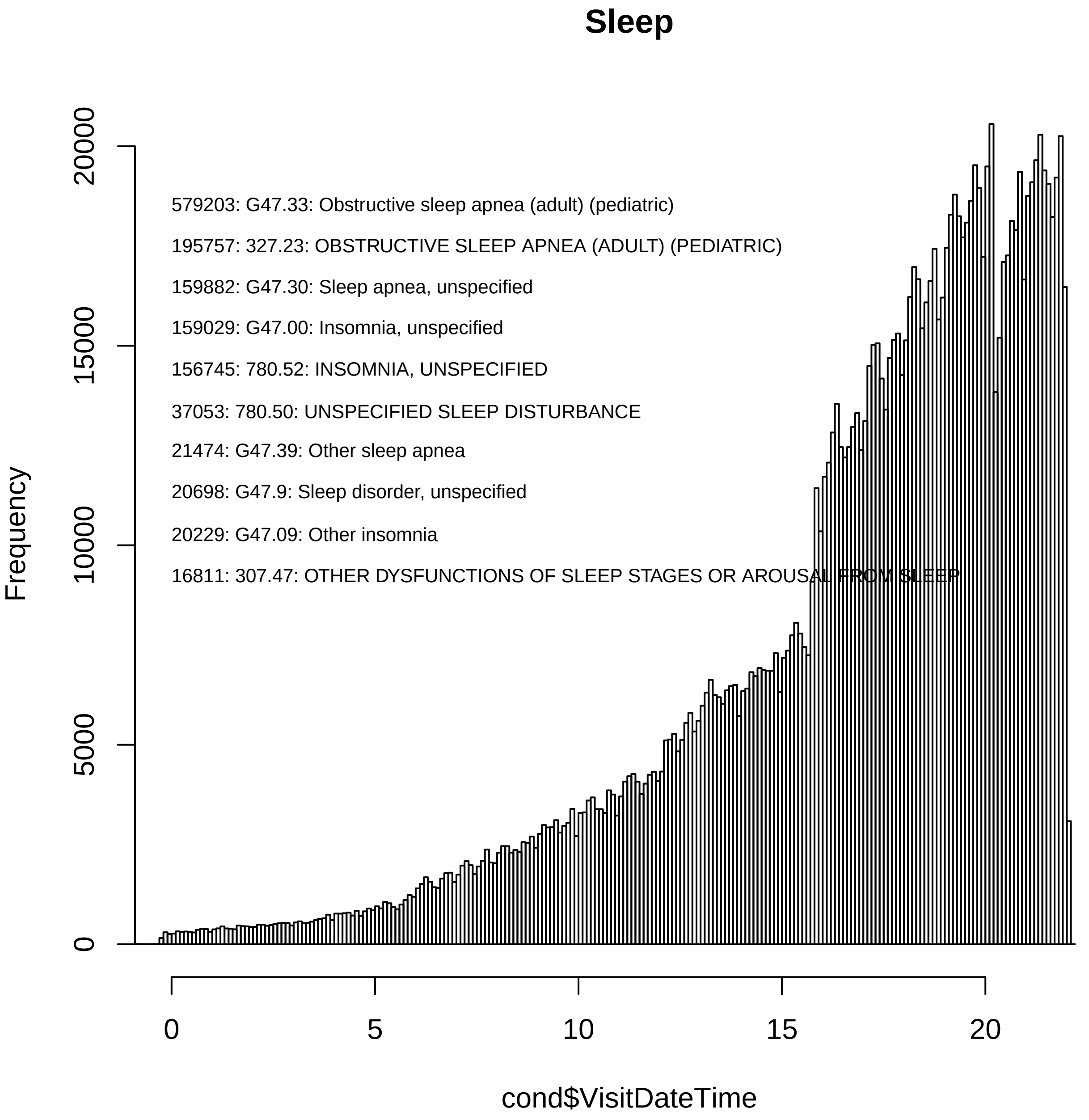
Sleep
579203: G47.33: Obstructive sleep apnea (adult) (pediatric)
195757: 327.23: OBSTRUCTIVE SLEEP APNEA (ADULT) (PEDIATRIC)
159882: G47.30: Sleep apnea, unspecified
159029: G47.00: Insomnia, unspecified
156745: 780.52: INSOMNIA, UNSPECIFIED
37053: 780.50: UNSPECIFIED SLEEP DISTURBANCE
21474: G47.39: Other sleep apnea
20698: G47.9: Sleep disorder, unspecified
20229: G47.09: Other insomnia
16811: 307.47: OTHER DYSFUNCTIONS OF SLEEP STAGES OR AROUSAL FROM SLEEP
Frequency
0
5000
10000
15000
20000
0
5
10
15
20
cond$VisitDateTime

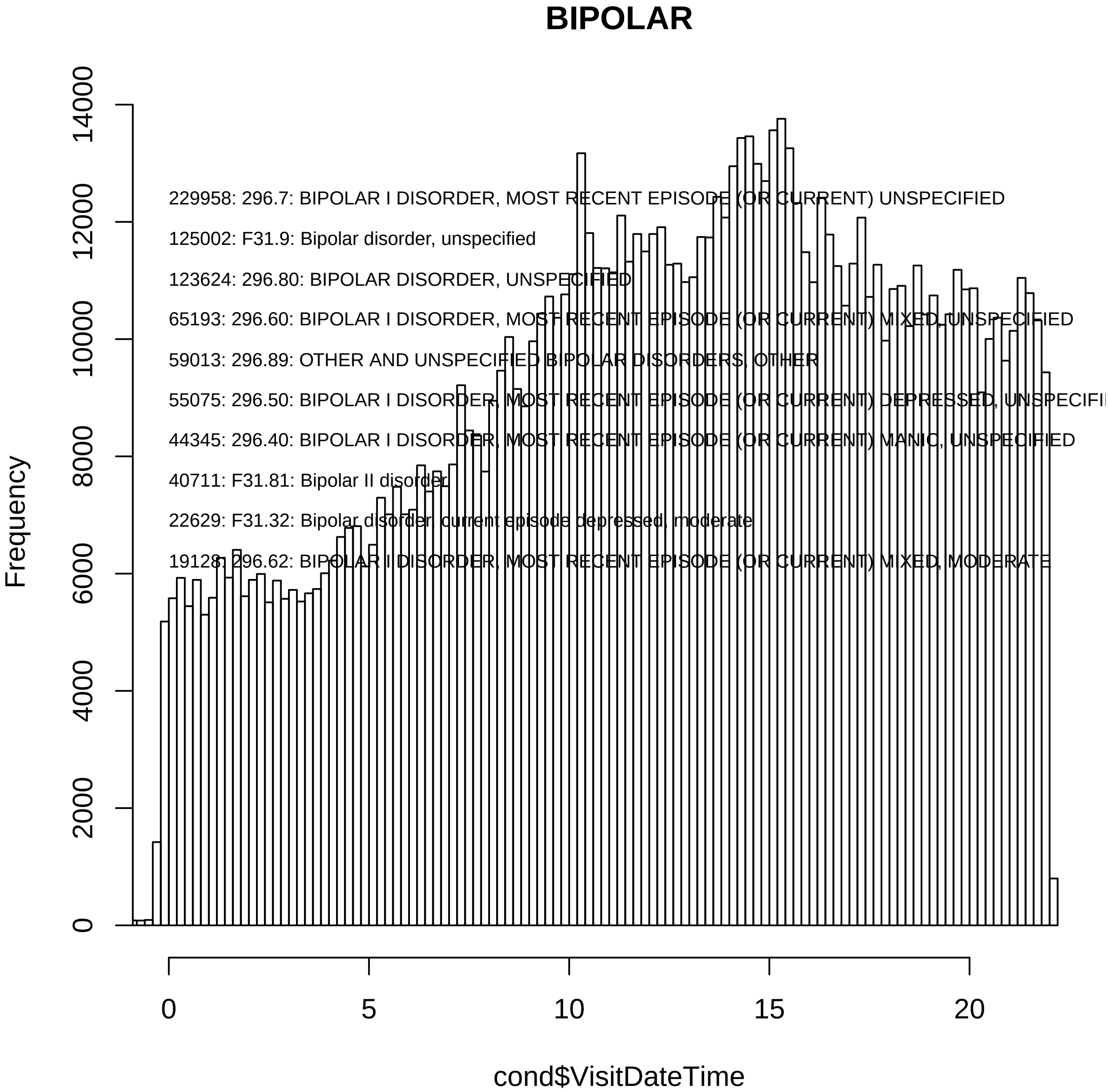
BIPOLAR
229958: 296.7: BIPOLAR I DISORDER, MOST RECENT EPISODE (OR CURRENT) UNSPECIFIED
125002: F31.9: Bipolar disorder, unspecified
123624: 296.80: BIPOLAR DISORDER, UNSPECIFIED
65193: 296.60: BIPOLAR I DISORDER, MOST RECENT EPISODE (OR CURRENT) MIXED, UNSPECIFIED
59013: 296.89: OTHER AND UNSPECIFIED BIPOLAR DISORDERS, OTHER
55075: 296.50: BIPOLAR I DISORDER, MOST RECENT EPISODE (OR CURRENT) DEPRESSED, UNSPECIFI
44345: 296.40: BIPOLAR I DISORDER, MOST RECENT EPISODE (OR CURRENT) MANIC, UNSPECIFIED
40711: F31.81: Bipolar II disorder
22629: F31.32: Bipolar disorder, current episode depressed, moderate
19128: 296.62: BIPOLAR I DISORDER, MOST RECENT EPISODE (OR CURRENT) MIXED, MODERATE
Frequency
0
2000
4000
6000
8000
10000
12000
14000
0
5
10
15
20
cond$VisitDateTime

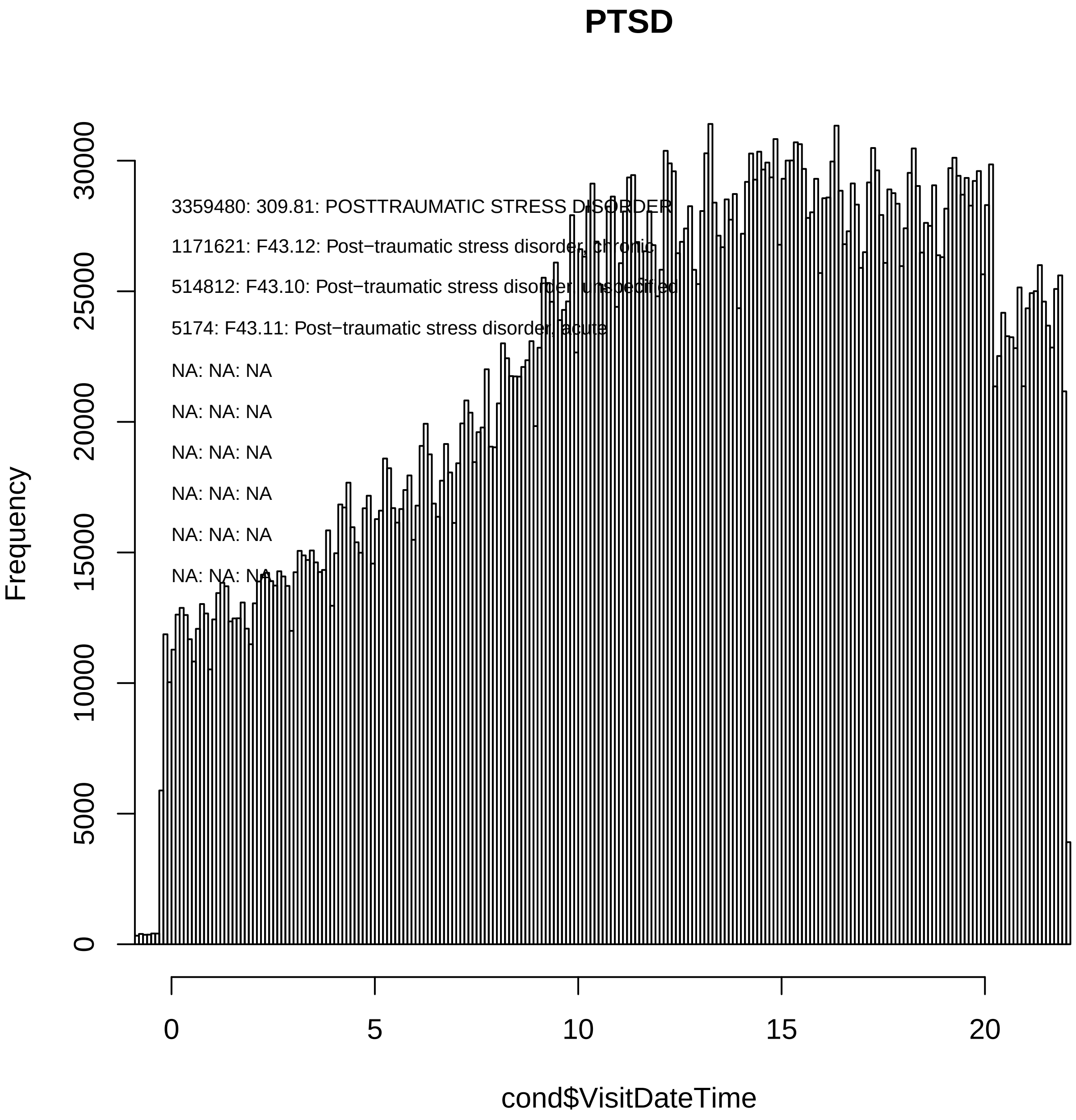
PTSD
3359480: 309.81: POSTTRAUMATIC STRESS DISORDER
1171621: F43.12: Post−traumatic stress disorder, chronic
514812: F43.10: Post−traumatic stress disorder, unspecified
5174: F43.11: Post−traumatic stress disorder, acute
NA: NA: NA
NA: NA: NA
NA: NA: NA
NA: NA: NA
NA: NA: NA
NA: NA: NA
Frequency
0
5000
10000
15000
20000
25000
30000
0
5
10
15
20
cond$VisitDateTime

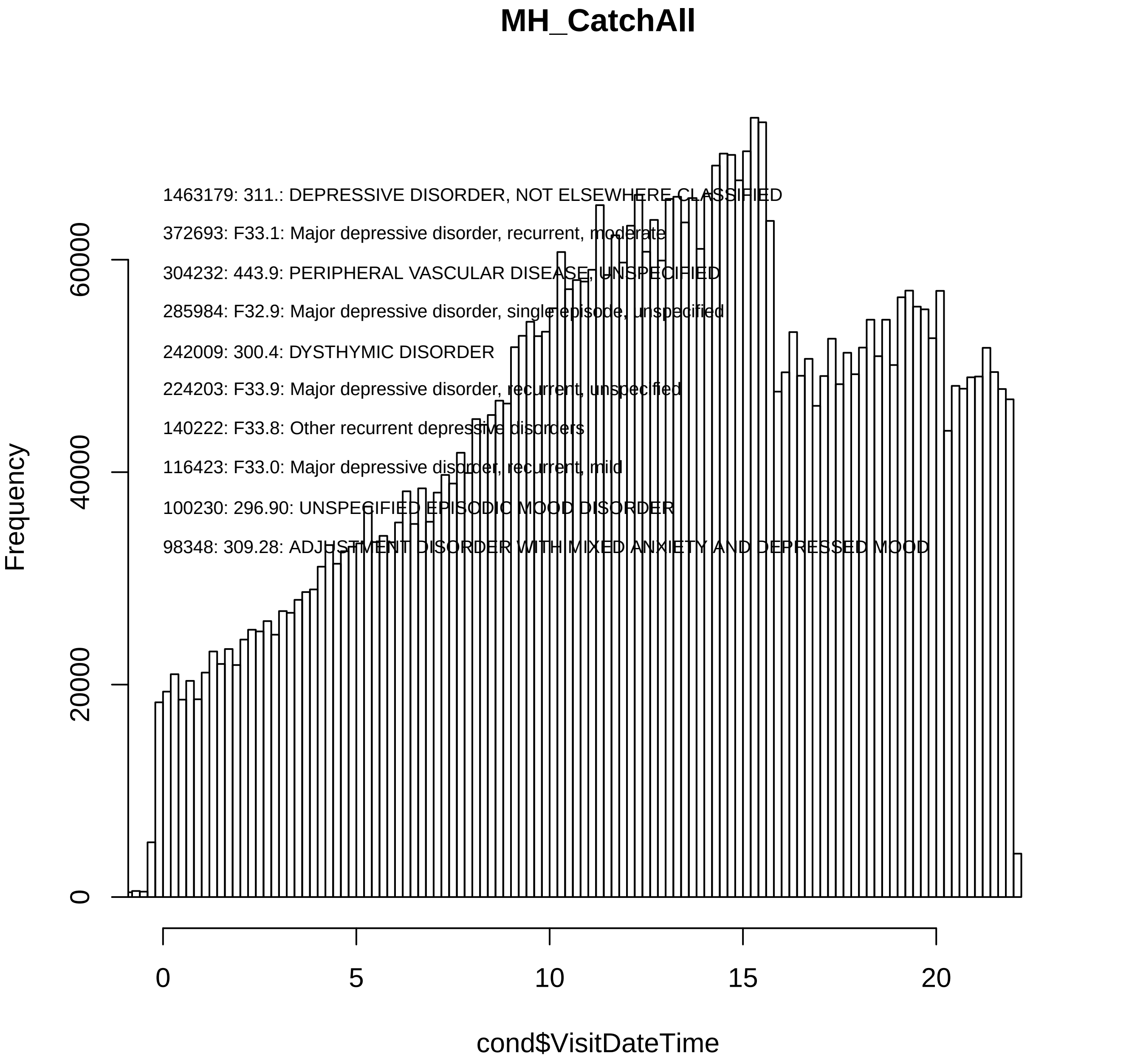
MH_CatchAll
1463179: 311.: DEPRESSIVE DISORDER, NOT ELSEWHERE CLASSIFIED
372693: F33.1: Major depressive disorder, recurrent, moderate
304232: 443.9: PERIPHERAL VASCULAR DISEASE, UNSPECIFIED
285984: F32.9: Major depressive disorder, single episode, unspecified
242009: 300.4: DYSTHYMIC DISORDER
224203: F33.9: Major depressive disorder, recurrent, unspecified
140222: F33.8: Other recurrent depressive disorders
116423: F33.0: Major depressive disorder, recurrent, mild
100230: 296.90: UNSPECIFIED EPISODIC MOOD DISORDER
98348: 309.28: ADJUSTMENT DISORDER WITH MIXED ANXIETY AND DEPRESSED MOOD
Frequency
0
20000
40000
60000
0
5
10
15
20
cond$VisitDateTime

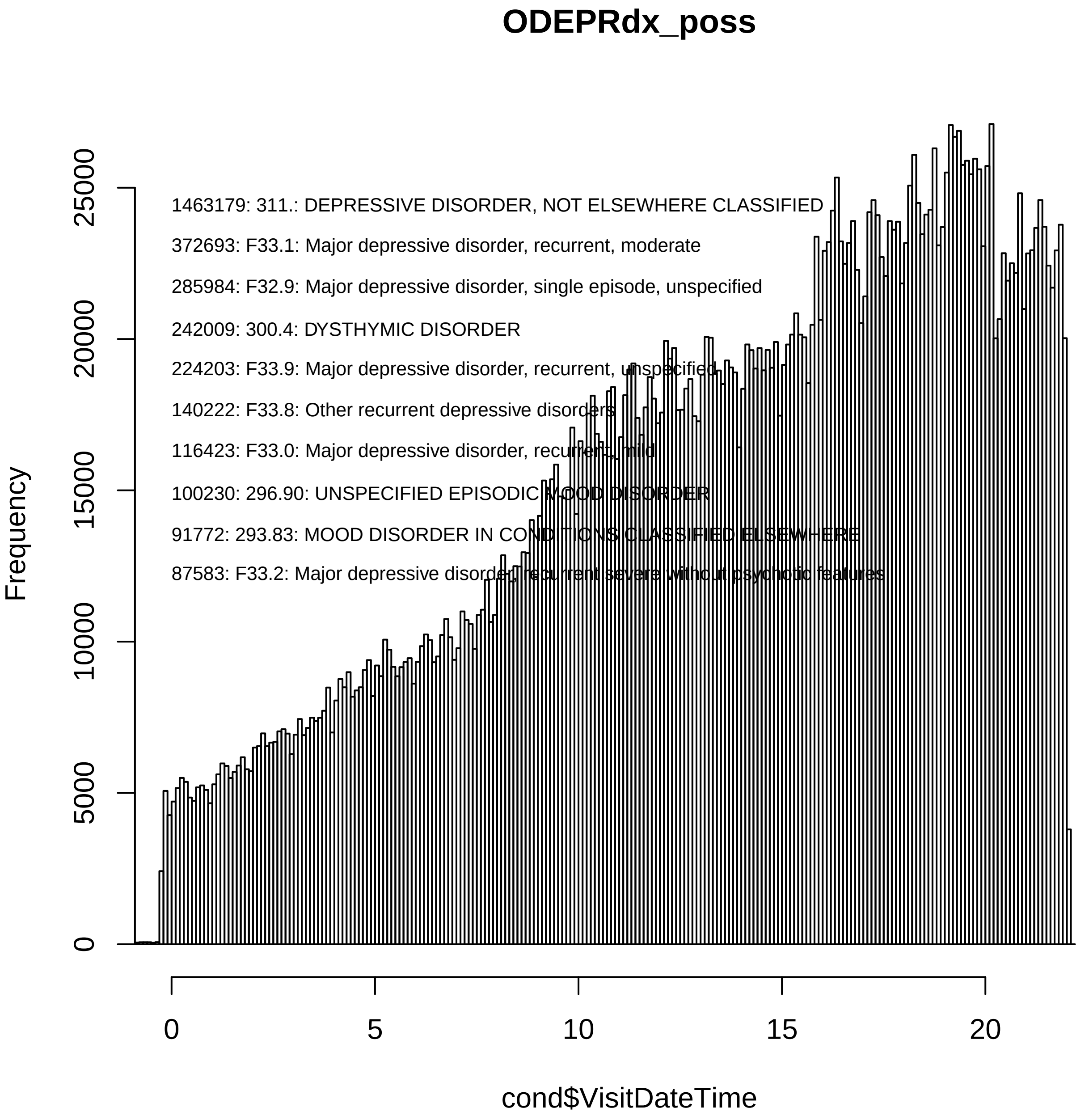
ODEPRdx_poss
1463179: 311.: DEPRESSIVE DISORDER, NOT ELSEWHERE CLASSIFIED
372693: F33.1: Major depressive disorder, recurrent, moderate
285984: F32.9: Major depressive disorder, single episode, unspecified
242009: 300.4: DYSTHYMIC DISORDER
224203: F33.9: Major depressive disorder, recurrent, unspecified
140222: F33.8: Other recurrent depressive disorders
116423: F33.0: Major depressive disorder, recurrent, mild
100230: 296.90: UNSPECIFIED EPISODIC MOOD DISORDER
91772: 293.83: MOOD DISORDER IN CONDITIONS CLASSIFIED ELSEWHERE
87583: F33.2: Major depressive disorder, recurrent severe without psychotic features
Frequency
0
5000
10000
15000
20000
25000
0
5
10
15
20
cond$VisitDateTime

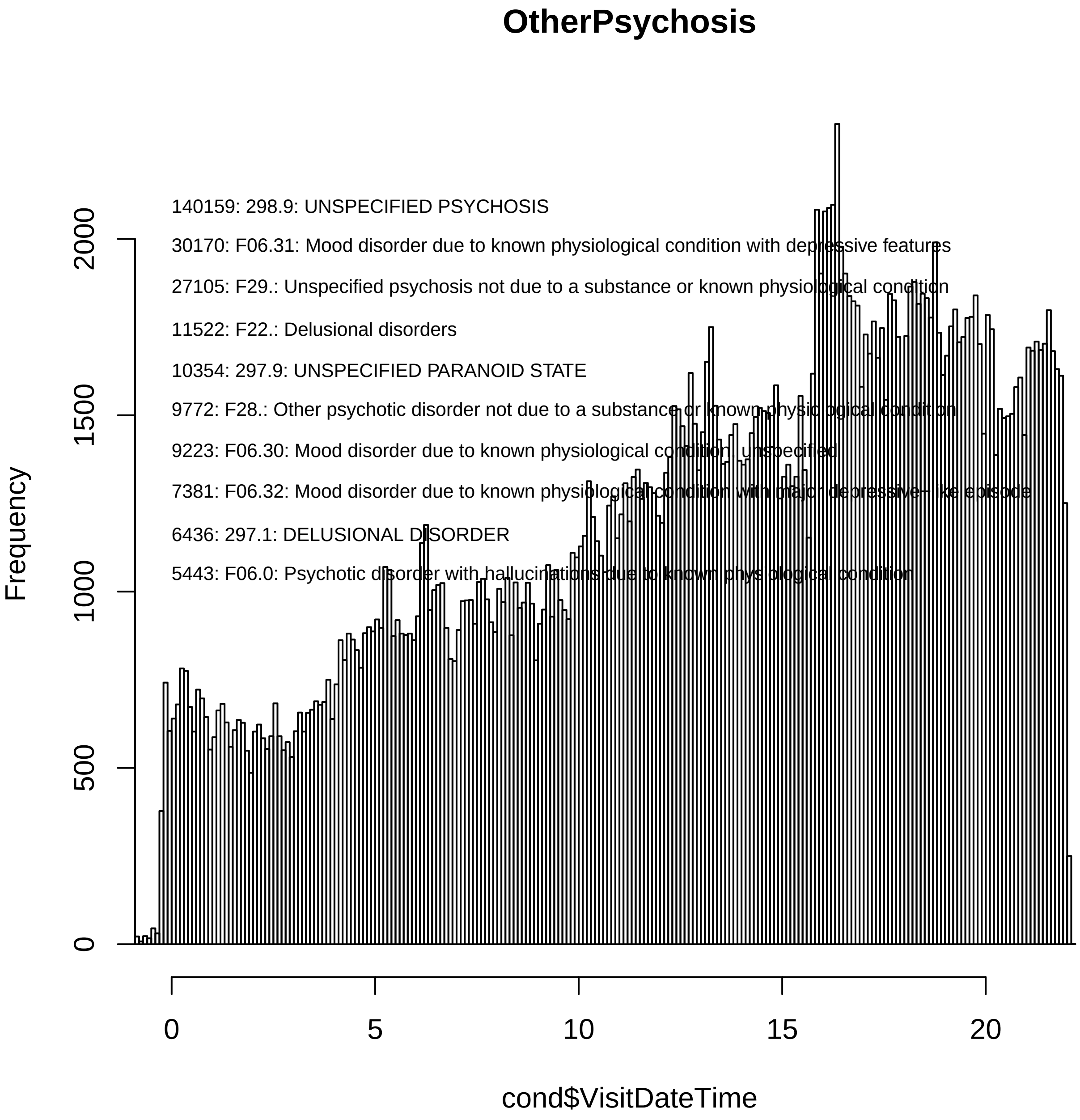

OtherPsychosis
140159: 298.9: UNSPECIFIED PSYCHOSIS
30170: F06.31: Mood disorder due to known physiological condition with depressive features
27105: F29.: Unspecified psychosis not due to a substance or known physiological condition
11522: F22.: Delusional disorders
10354: 297.9: UNSPECIFIED PARANOID STATE
9772: F28.: Other psychotic disorder not due to a substance or known physiological condition
9223: F06.30: Mood disorder due to known physiological condition, unspecified
7381: F06.32: Mood disorder due to known physiological condition with major depressive−like episode
6436: 297.1: DELUSIONAL DISORDER
5443: F06.0: Psychotic disorder with hallucinations due to known physiological condition
Frequency
0
500
1000
1500
2000
0
5
10
15
20
cond$VisitDateTime

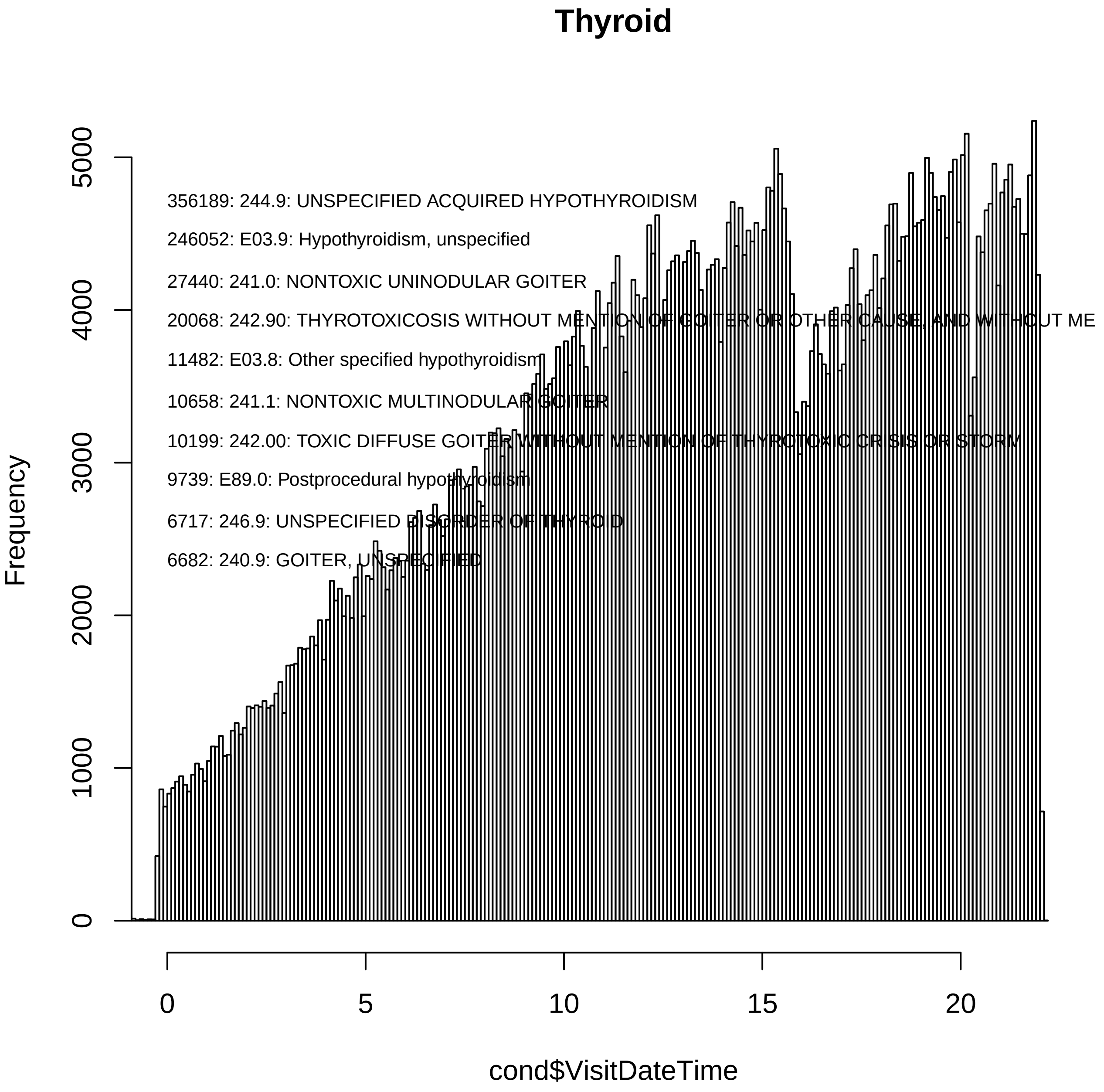
Thyroid
356189: 244.9: UNSPECIFIED ACQUIRED HYPOTHYROIDISM
246052: E03.9: Hypothyroidism, unspecified
27440: 241.0: NONTOXIC UNINODULAR GOITER
20068: 242.90: THYROTOXICOSIS WITHOUT MENTION OF GOITER OR OTHER CAUSE, AND WITHOUT ME
11482: E03.8: Other specified hypothyroidism
10658: 241.1: NONTOXIC MULTINODULAR GOITER
10199: 242.00: TOXIC DIFFUSE GOITER WITHOUT MENTION OF THYROTOXIC CRISIS OR STORM
9739: E89.0: Postprocedural hypothyroidism
6717: 246.9: UNSPECIFIED DISORDER OF THYROID
6682: 240.9: GOITER, UNSPECIFIED
Frequency
0
1000
2000
3000
4000
5000
0
5
10
15
20
cond$VisitDateTime

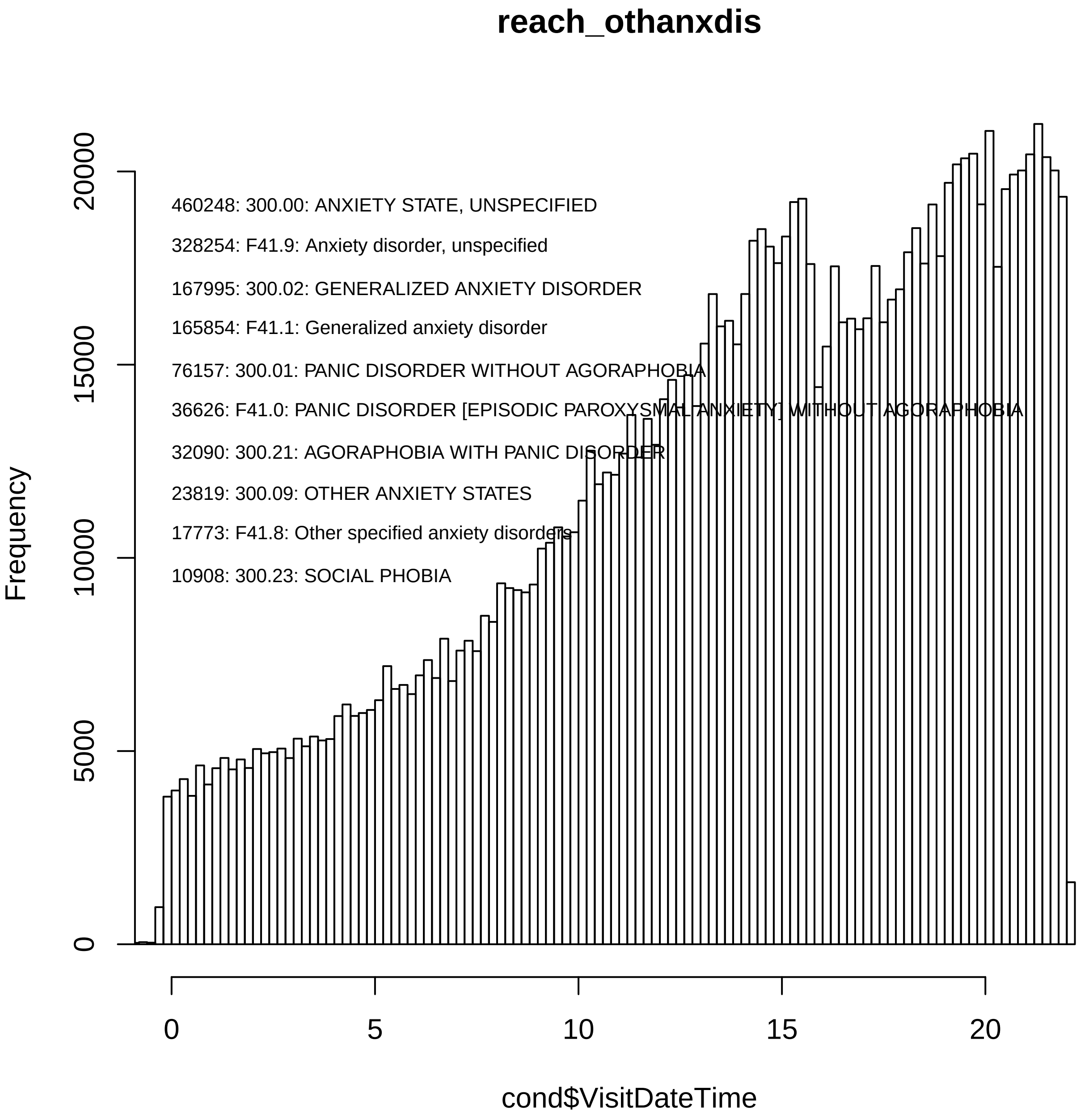
reach_othanxdis
460248: 300.00: ANXIETY STATE, UNSPECIFIED
328254: F41.9: Anxiety disorder, unspecified
167995: 300.02: GENERALIZED ANXIETY DISORDER
165854: F41.1: Generalized anxiety disorder
76157: 300.01: PANIC DISORDER WITHOUT AGORAPHOBIA
36626: F41.0: PANIC DISORDER [EPISODIC PAROXYSMAL ANXIETY] WITHOUT AGORAPHOBIA
32090: 300.21: AGORAPHOBIA WITH PANIC DISORDER
23819: 300.09: OTHER ANXIETY STATES
17773: F41.8: Other specified anxiety disorders
10908: 300.23: SOCIAL PHOBIA
Frequency
0
5000
10000
15000
20000
0
5
10
15
20
cond$VisitDateTime

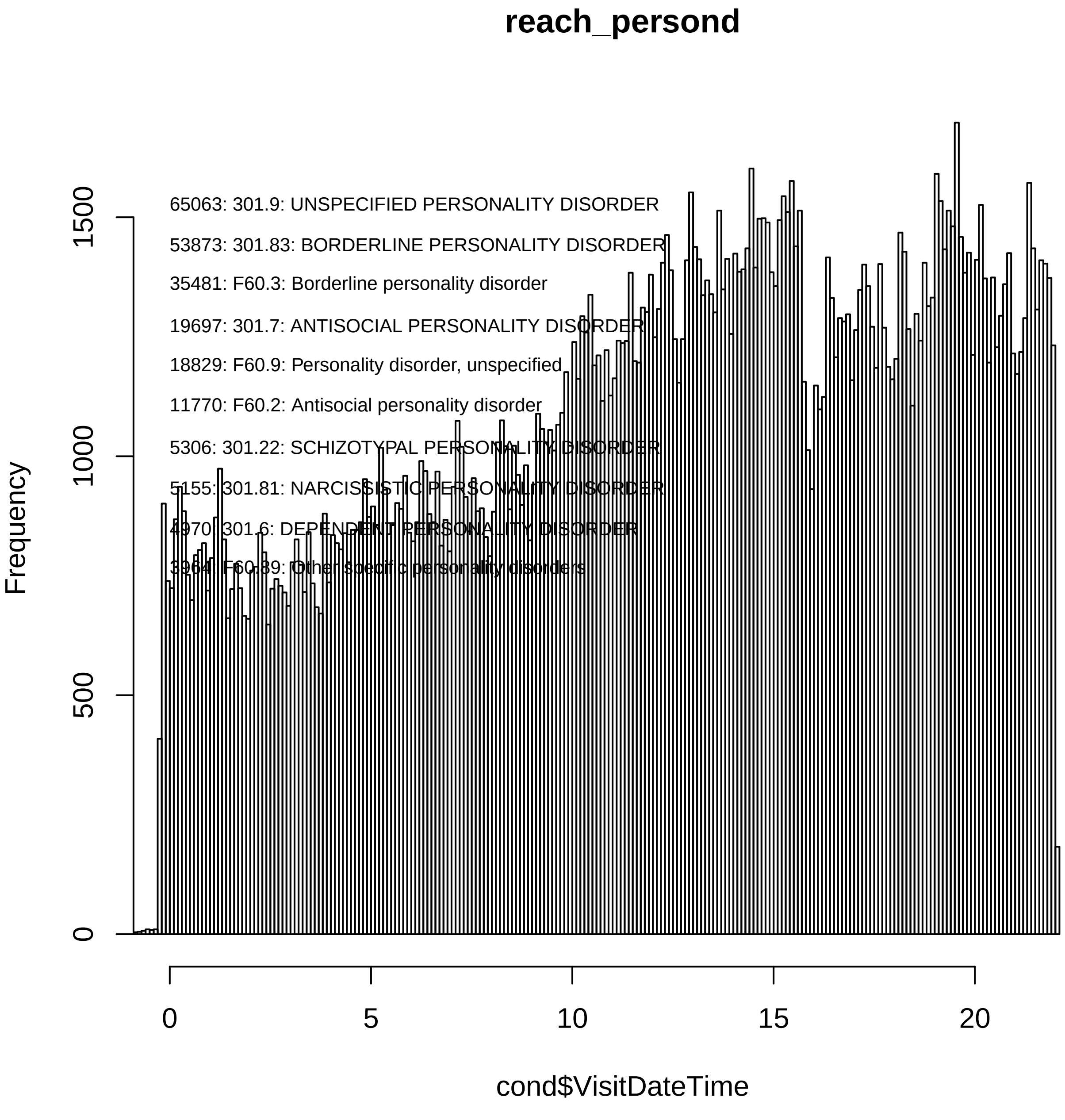
reach_persond
65063: 301.9: UNSPECIFIED PERSONALITY DISORDER
53873: 301.83: BORDERLINE PERSONALITY DISORDER
35481: F60.3: Borderline personality disorder
19697: 301.7: ANTISOCIAL PERSONALITY DISORDER
18829: F60.9: Personality disorder, unspecified
11770: F60.2: Antisocial personality disorder
5306: 301.22: SCHIZOTYPAL PERSONALITY DISORDER
5155: 301.81: NARCISSISTIC PERSONALITY DISORDER
4970: 301.6: DEPENDENT PERSONALITY DISORDER
3964: F60.89: Other specific personality disorders
Frequency
0
500
1000
1500
0
5
10
15
20
cond$VisitDateTime

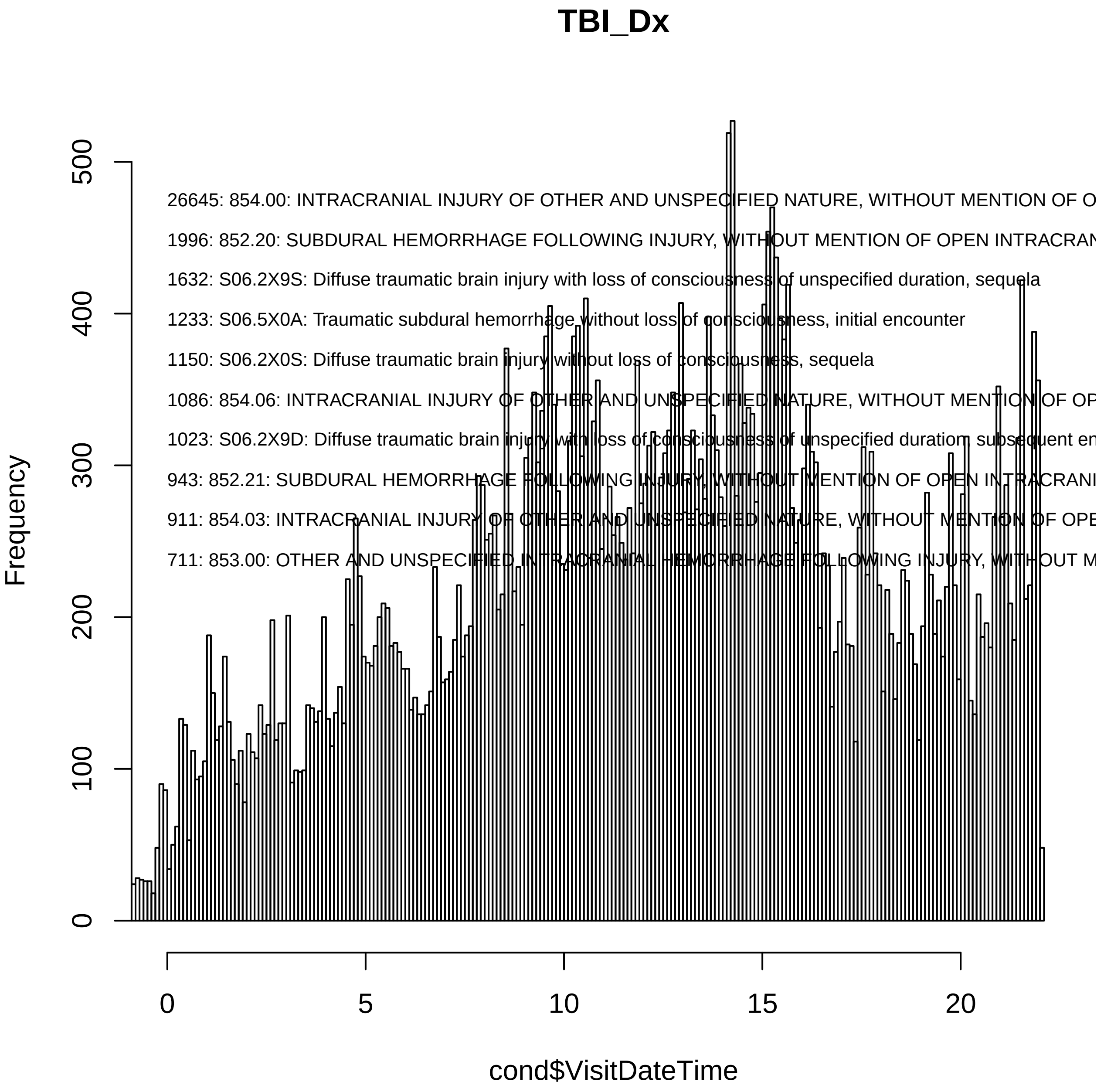

TBI_Dx
26645: 854.00: INTRACRANIAL INJURY OF OTHER AND UNSPECIFIED NATURE, WITHOUT MENTION OF O
1996: 852.20: SUBDURAL HEMORRHAGE FOLLOWING INJURY, WITHOUT MENTION OF OPEN INTRACRAN
1632: S06.2X9S: Diffuse traumatic brain injury with loss of consciousness of unspecified duration, sequela
1233: S06.5X0A: Traumatic subdural hemorrhage without loss of consciousness, initial encounter
1150: S06.2X0S: Diffuse traumatic brain injury without loss of consciousness, sequela
1086: 854.06: INTRACRANIAL INJURY OF OTHER AND UNSPECIFIED NATURE, WITHOUT MENTION OF OF
1023: S06.2X9D: Diffuse traumatic brain injury with loss of consciousness of unspecified duration, subsequent en
943: 852.21: SUBDURAL HEMORRHAGE FOLLOWING INJURY, WITHOUT MENTION OF OPEN INTRACRANI
911: 854.03: INTRACRANIAL INJURY OF OTHER AND UNSPECIFIED NATURE, WITHOUT MENTION OF OPE
711: 853.00: OTHER AND UNSPECIFIED INTRACRANIAL HEMORRHAGE FOLLOWING INJURY, WITHOUT M
Frequency
0
100
200
300
400
500
0
5
10
15
20
cond$VisitDateTime

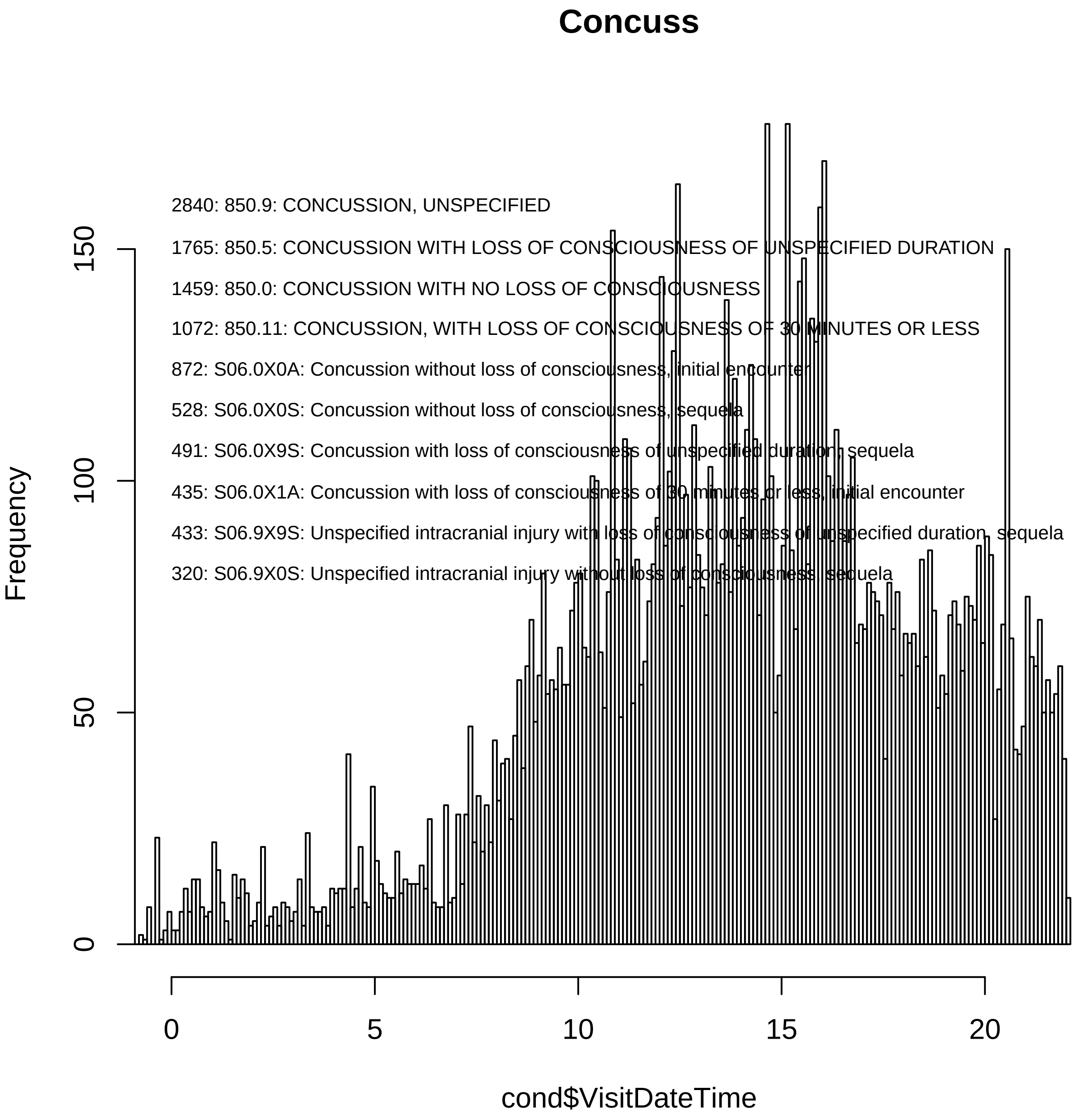
Concuss
2840: 850.9: CONCUSSION, UNSPECIFIED
1765: 850.5: CONCUSSION WITH LOSS OF CONSCIOUSNESS OF UNSPECIFIED DURATION
1459: 850.0: CONCUSSION WITH NO LOSS OF CONSCIOUSNESS
1072: 850.11: CONCUSSION, WITH LOSS OF CONSCIOUSNESS OF 30 MINUTES OR LESS
872: S06.0X0A: Concussion without loss of consciousness, initial encounter
528: S06.0X0S: Concussion without loss of consciousness, sequela
491: S06.0X9S: Concussion with loss of consciousness of unspecified duration, sequela
435: S06.0X1A: Concussion with loss of consciousness of 30 minutes or less, initial encounter
433: S06.9X9S: Unspecified intracranial injury with loss of consciousness of unspecified duration, sequela
320: S06.9X0S: Unspecified intracranial injury without loss of consciousness, sequela
Frequency
0
50
100
150
0
5
10
15
20
cond$VisitDateTime

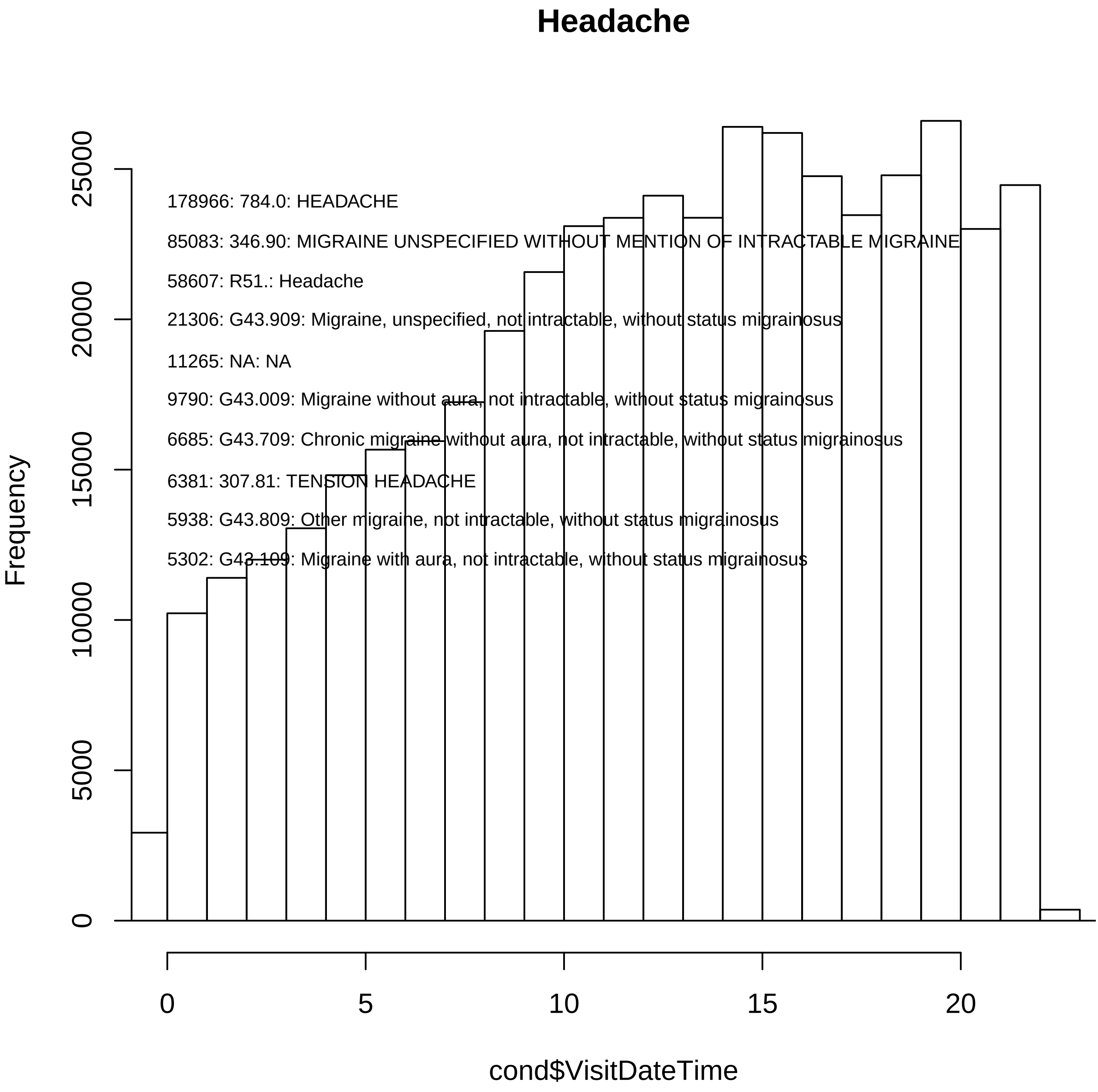
Headache
178966: 784.0: HEADACHE
85083: 346.90: MIGRAINE UNSPECIFIED WITHOUT MENTION OF INTRACTABLE MIGRAINE
58607: R51.: Headache
21306: G43.909: Migraine, unspecified, not intractable, without status migrainosus
11265: NA: NA
9790: G43.009: Migraine without aura, not intractable, without status migrainosus
6685: G43.709: Chronic migraine without aura, not intractable, without status migrainosus
6381: 307.81: TENSION HEADACHE
5938: G43.809: Other migraine, not intractable, without status migrainosus
5302: G43.109: Migraine with aura, not intractable, without status migrainosus
Frequency
0
5000
10000
15000
20000
25000
0
5
10
15
20
cond$VisitDateTime

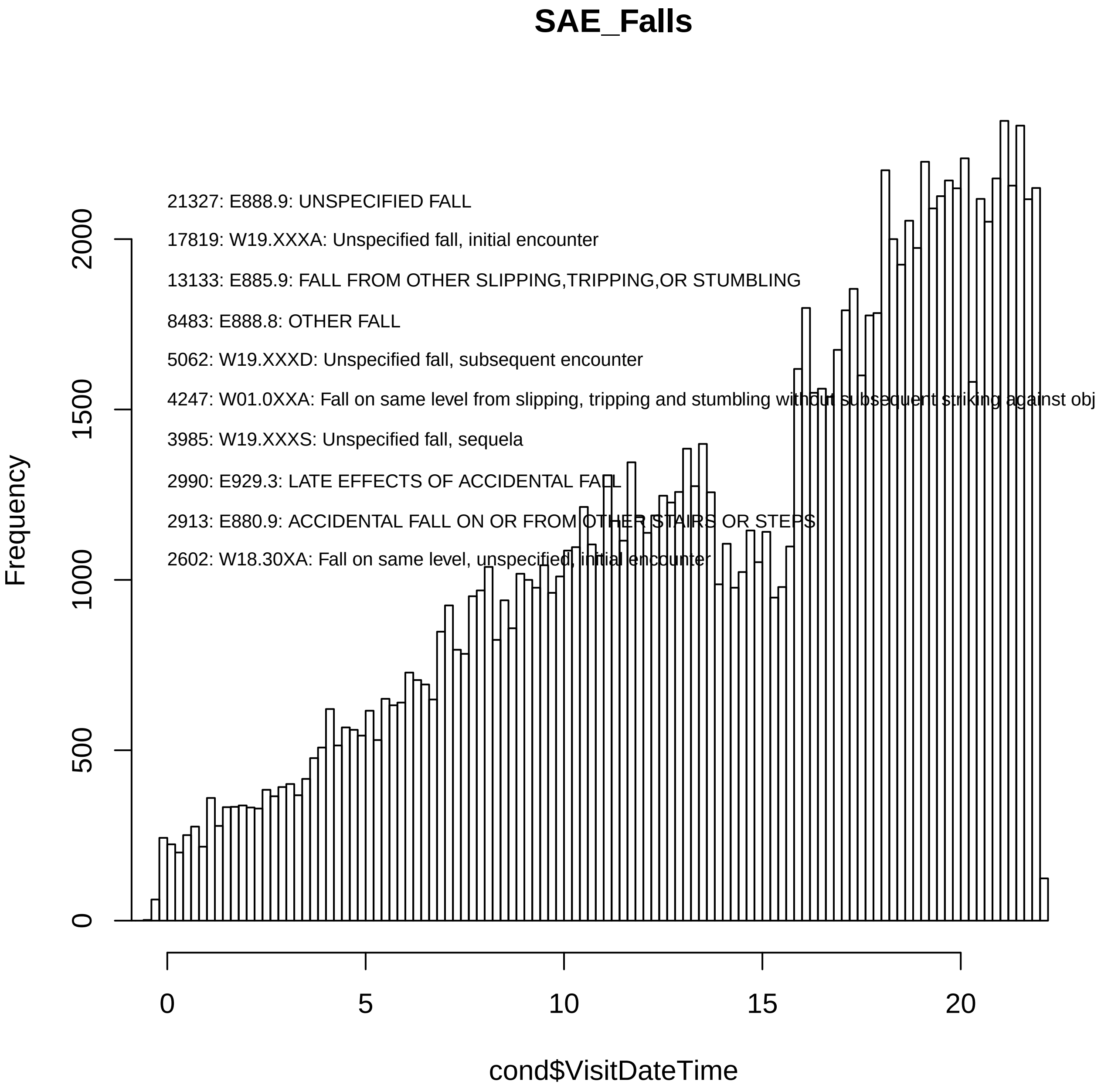
SAE_Falls
21327: E888.9: UNSPECIFIED FALL
17819: W19.XXXA: Unspecified fall, initial encounter
13133: E885.9: FALL FROM OTHER SLIPPING,TRIPPING,OR STUMBLING
8483: E888.8: OTHER FALL
5062: W19.XXXD: Unspecified fall, subsequent encounter
4247: W01.0XXA: Fall on same level from slipping, tripping and stumbling without subsequent striking against obj
3985: W19.XXXS: Unspecified fall, sequela
2990: E929.3: LATE EFFECTS OF ACCIDENTAL FALL
2913: E880.9: ACCIDENTAL FALL ON OR FROM OTHER STAIRS OR STEPS
2602: W18.30XA: Fall on same level, unspecified, initial encounter
Frequency
0
500
1000
1500
2000
0
5
10
15
20
cond$VisitDateTime

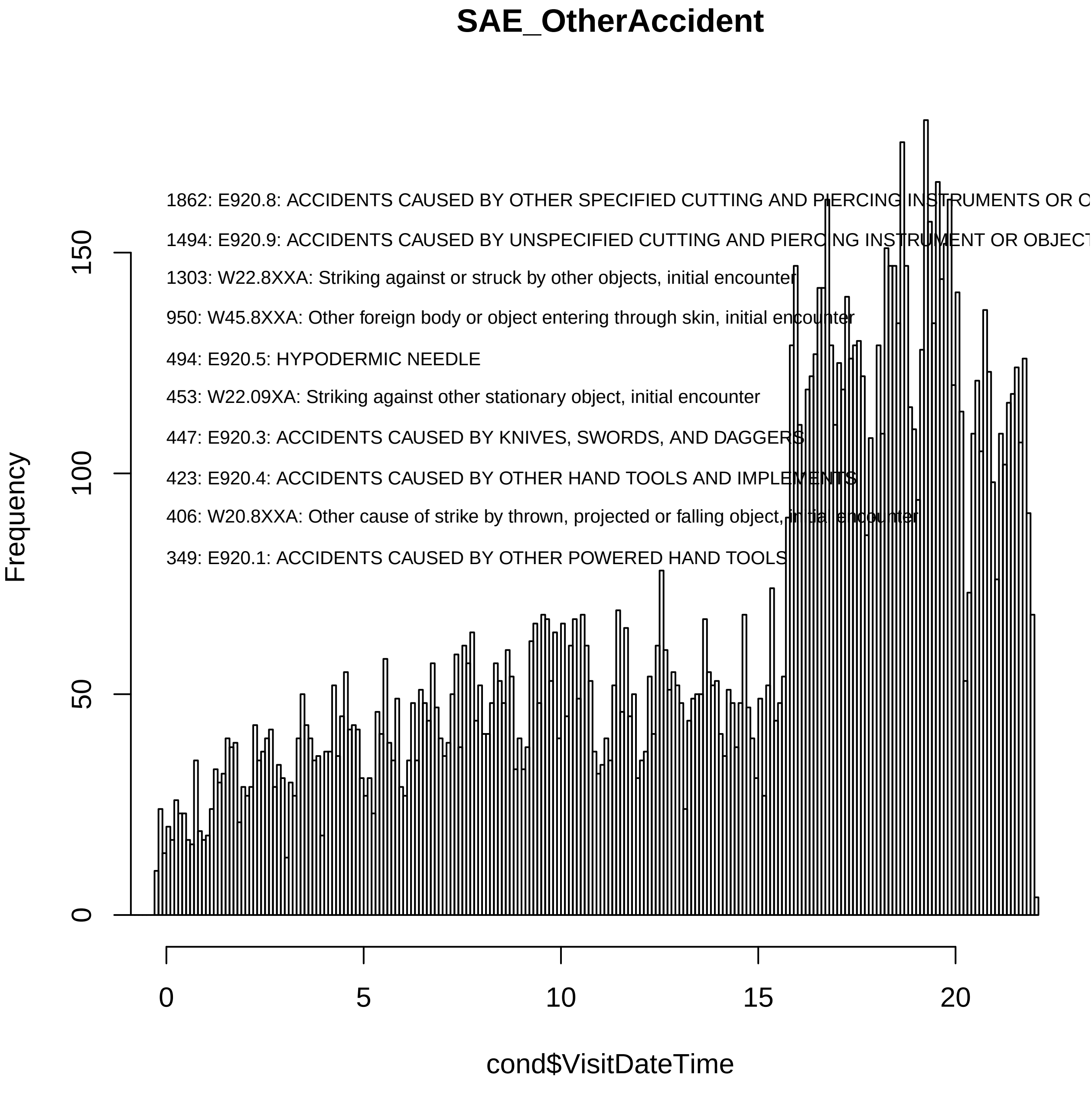
SAE_OtherAccident
1862: E920.8: ACCIDENTS CAUSED BY OTHER SPECIFIED CUTTING AND PIERCING INSTRUMENTS OR C
1494: E920.9: ACCIDENTS CAUSED BY UNSPECIFIED CUTTING AND PIERCING INSTRUMENT OR OBJECT
1303: W22.8XXA: Striking against or struck by other objects, initial encounter
950: W45.8XXA: Other foreign body or object entering through skin, initial encounter
494: E920.5: HYPODERMIC NEEDLE
453: W22.09XA: Striking against other stationary object, initial encounter
447: E920.3: ACCIDENTS CAUSED BY KNIVES, SWORDS, AND DAGGERS
423: E920.4: ACCIDENTS CAUSED BY OTHER HAND TOOLS AND IMPLEMENTS
406: W20.8XXA: Other cause of strike by thrown, projected or falling object, initial encounter
349: E920.1: ACCIDENTS CAUSED BY OTHER POWERED HAND TOOLS
Frequency
0
50
100
150
0
5
10
15
20
cond$VisitDateTime

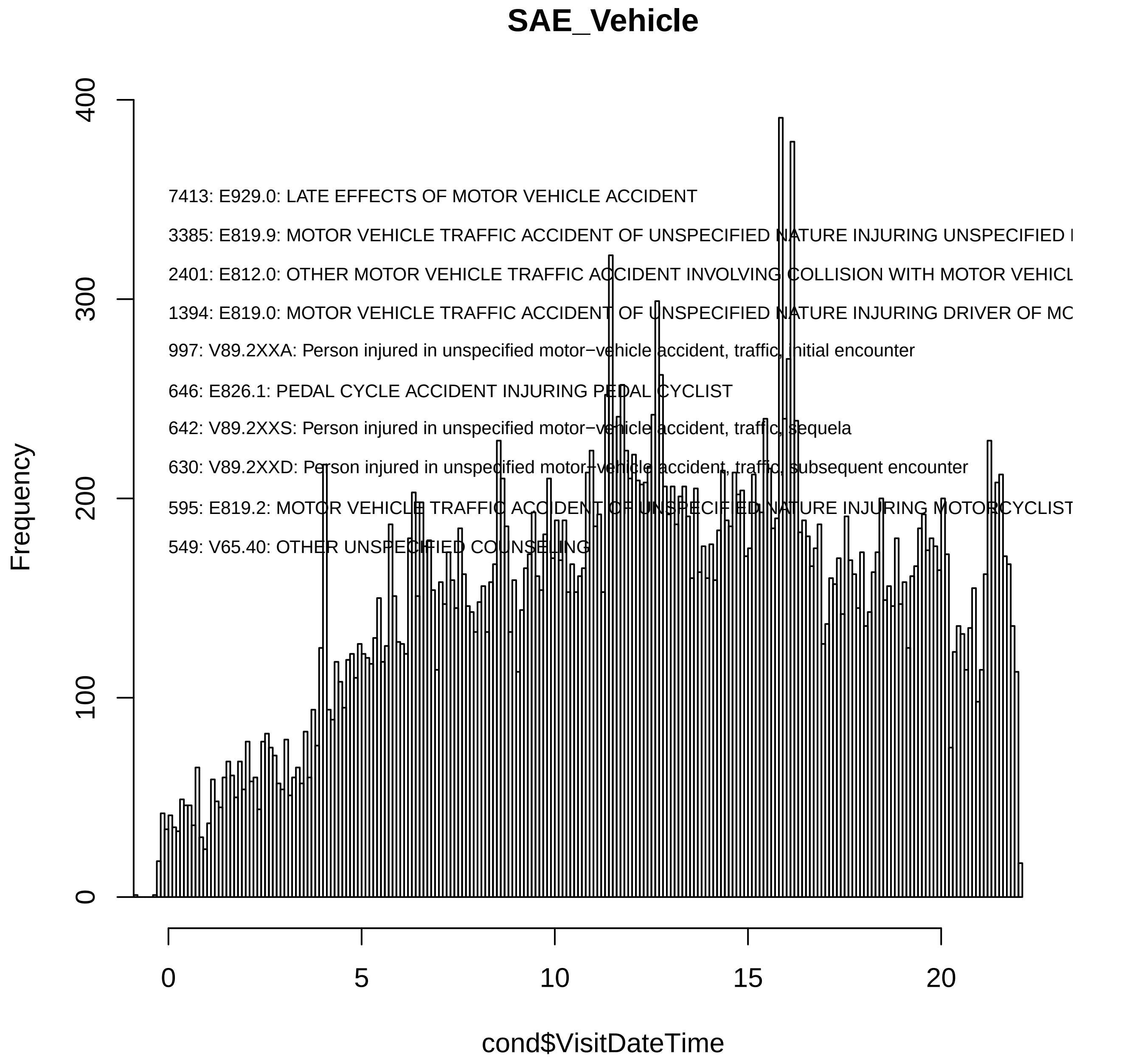
SAE_Vehicle
Frequency
cond$VisitDateTime
0
100
200
300
400
0
5
10
15
20
7413: E929.0: LATE EFFECTS OF MOTOR VEHICLE ACCIDENT
3385: E819.9: MOTOR VEHICLE TRAFFIC ACCIDENT OF UNSPECIFIED NATURE INJURING UNSPECIFIED
2401: E812.0: OTHER MOTOR VEHICLE TRAFFIC ACCIDENT INVOLVING COLLISION WITH MOTOR VEHICL
1394: E819.0: MOTOR VEHICLE TRAFFIC ACCIDENT OF UNSPECIFIED NATURE INJURING DRIVER OF MC
997: V89.2XXA: Person injured in unspecified motor−vehicle accident, traffic, initial encounter
646: E826.1: PEDAL CYCLE ACCIDENT INJURING PEDAL CYCLIST
642: V89.2XXS: Person injured in unspecified motor−vehicle accident, traffic, sequela
630: V89.2XXD: Person injured in unspecified motor−vehicle accident, traffic, subsequent encounter
595: E819.2: MOTOR VEHICLE TRAFFIC ACCIDENT OF UNSPECIFIED NATURE INJURING MOTORCYCLIST
549: V65.40: OTHER UNSPECIFIED COUNSELING

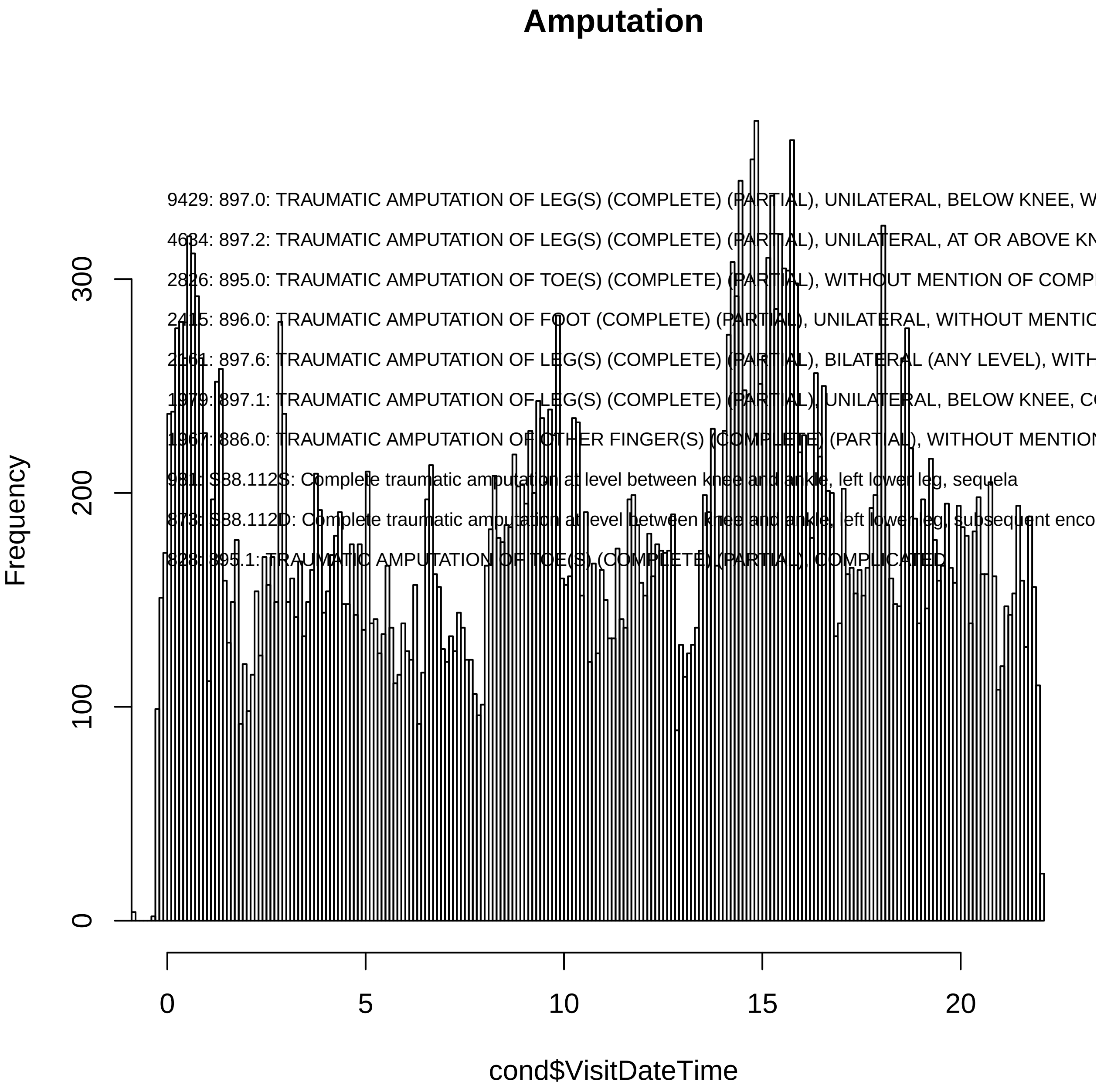
Amputation
9429: 897.0: TRAUMATIC AMPUTATION OF LEG(S) (COMPLETE) (PARTIAL), UNILATERAL, BELOW KNEE, W
4634: 897.2: TRAUMATIC AMPUTATION OF LEG(S) (COMPLETE) (PARTIAL), UNILATERAL, AT OR ABOVE KN
2826: 895.0: TRAUMATIC AMPUTATION OF TOE(S) (COMPLETE) (PARTIAL), WITHOUT MENTION OF COMPL
2415: 896.0: TRAUMATIC AMPUTATION OF FOOT (COMPLETE) (PARTIAL), UNILATERAL, WITHOUT MENTIO
2161: 897.6: TRAUMATIC AMPUTATION OF LEG(S) (COMPLETE) (PARTIAL), BILATERAL (ANY LEVEL), WITH
1979: 897.1: TRAUMATIC AMPUTATION OF LEG(S) (COMPLETE) (PARTIAL), UNILATERAL, BELOW KNEE, CO
1967: 886.0: TRAUMATIC AMPUTATION OF OTHER FINGER(S) (COMPLETE) (PARTIAL), WITHOUT MENTION
981: S88.112S: Complete traumatic amputation at level between knee and ankle, left lower leg, sequela
873: S88.112D: Complete traumatic amputation at level between knee and ankle, left lower leg, subsequent enco
828: 895.1: TRAUMATIC AMPUTATION OF TOE(S) (COMPLETE) (PARTIAL), COMPLICATED
Frequency
0
100
200
300
0
5
10
15
20
cond$VisitDateTime

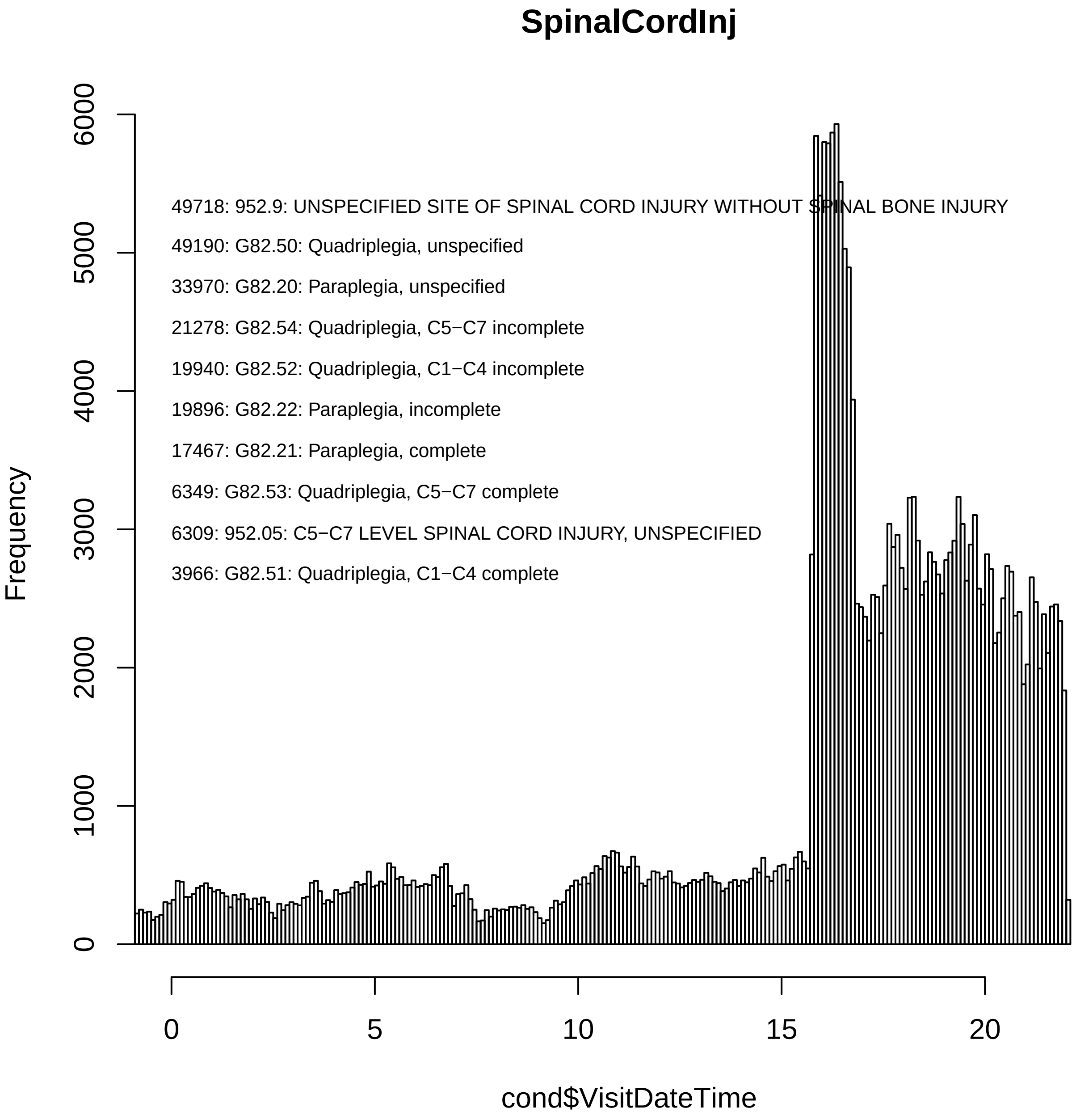
SpinalCordInj
49718: 952.9: UNSPECIFIED SITE OF SPINAL CORD INJURY WITHOUT SPINAL BONE INJURY
49190: G82.50: Quadriplegia, unspecified
33970: G82.20: Paraplegia, unspecified
21278: G82.54: Quadriplegia, C5−C7 incomplete
19940: G82.52: Quadriplegia, C1−C4 incomplete
19896: G82.22: Paraplegia, incomplete
17467: G82.21: Paraplegia, complete
6349: G82.53: Quadriplegia, C5−C7 complete
6309: 952.05: C5−C7 LEVEL SPINAL CORD INJURY, UNSPECIFIED
3966: G82.51: Quadriplegia, C1−C4 complete
Frequency
0
1000
2000
3000
4000
5000
6000
0
5
10
15
20
cond$VisitDateTime

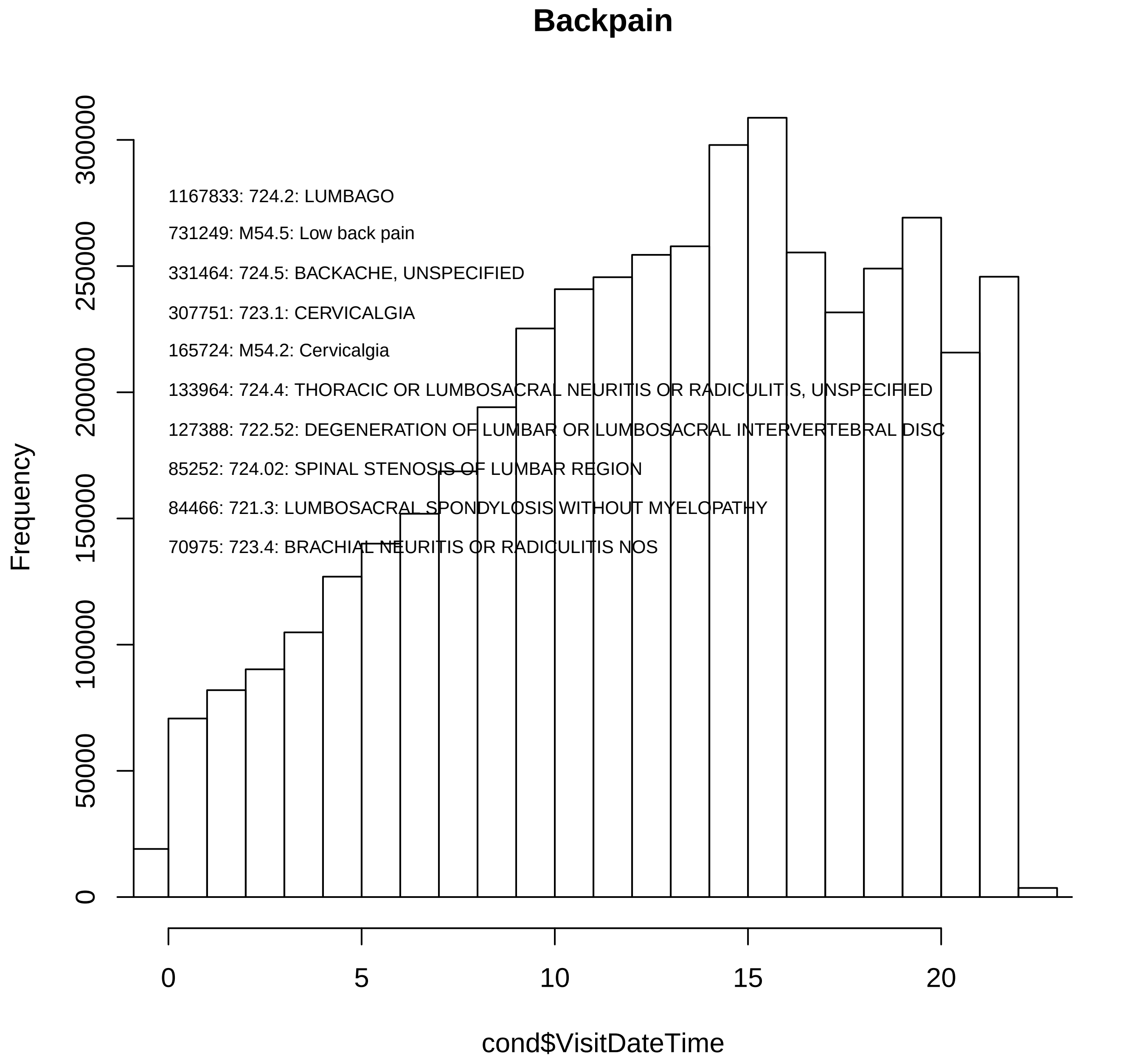
Backpain
1167833: 724.2: LUMBAGO
731249: M54.5: Low back pain
331464: 724.5: BACKACHE, UNSPECIFIED
307751: 723.1: CERVICALGIA
165724: M54.2: Cervicalgia
133964: 724.4: THORACIC OR LUMBOSACRAL NEURITIS OR RADICULITIS, UNSPECIFIED
127388: 722.52: DEGENERATION OF LUMBAR OR LUMBOSACRAL INTERVERTEBRAL DISC
85252: 724.02: SPINAL STENOSIS OF LUMBAR REGION
84466: 721.3: LUMBOSACRAL SPONDYLOSIS WITHOUT MYELOPATHY
70975: 723.4: BRACHIAL NEURITIS OR RADICULITIS NOS
Frequency
0
50000
100000
150000
200000
250000
300000
0
5
10
15
20
cond$VisitDateTime

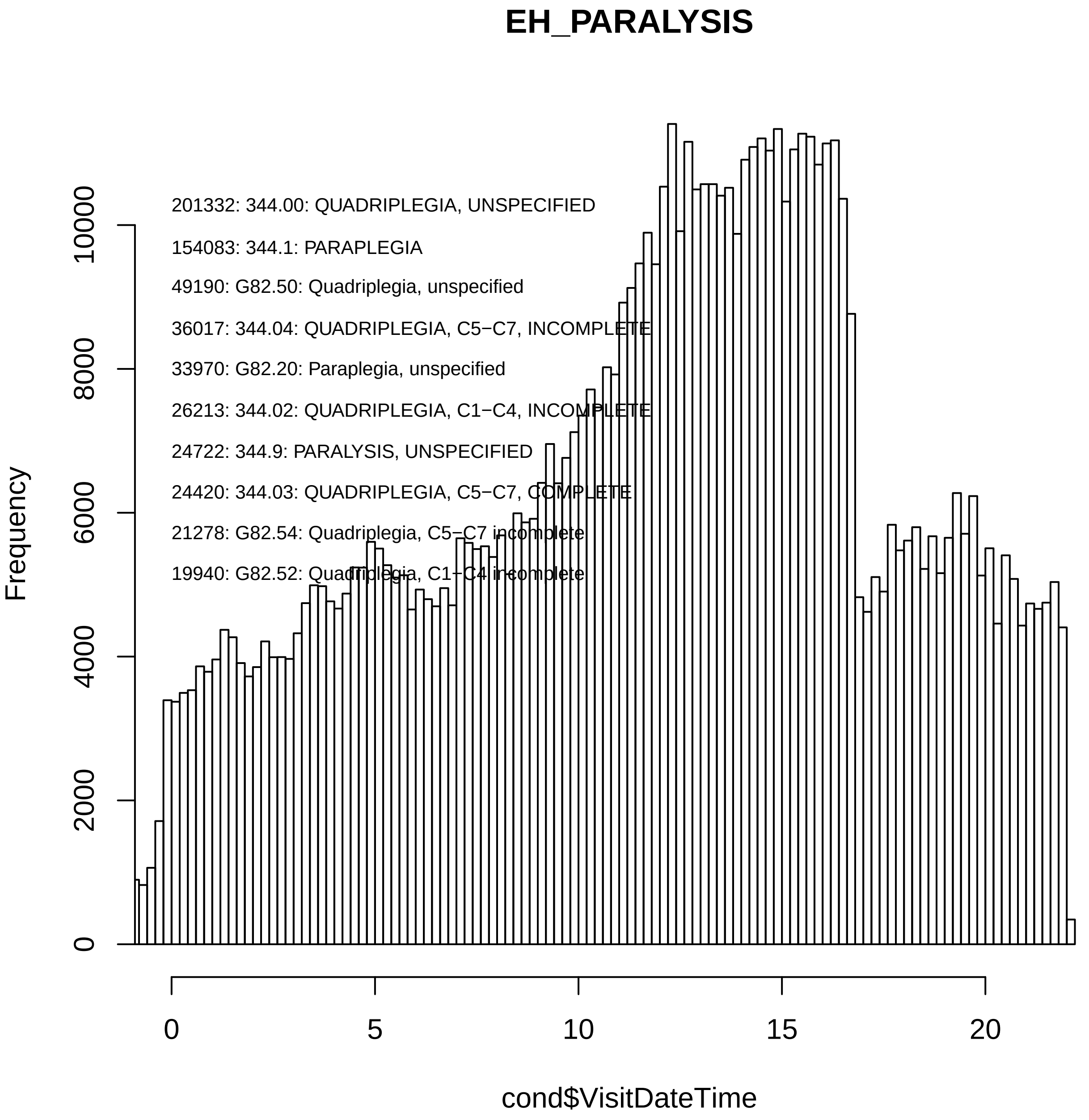
EH_PARALYSIS
201332: 344.00: QUADRIPLEGIA, UNSPECIFIED
154083: 344.1: PARAPLEGIA
49190: G82.50: Quadriplegia, unspecified
36017: 344.04: QUADRIPLEGIA, C5−C7, INCOMPLETE
33970: G82.20: Paraplegia, unspecified
26213: 344.02: QUADRIPLEGIA, C1−C4, INCOMPLETE
24722: 344.9: PARALYSIS, UNSPECIFIED
24420: 344.03: QUADRIPLEGIA, C5−C7, COMPLETE
21278: G82.54: Quadriplegia, C5−C7 incomplete
19940: G82.52: Quadriplegia, C1−C4 incomplete
Frequency
0
2000
4000
6000
8000
10000
0
5
10
15
20
cond$VisitDateTime

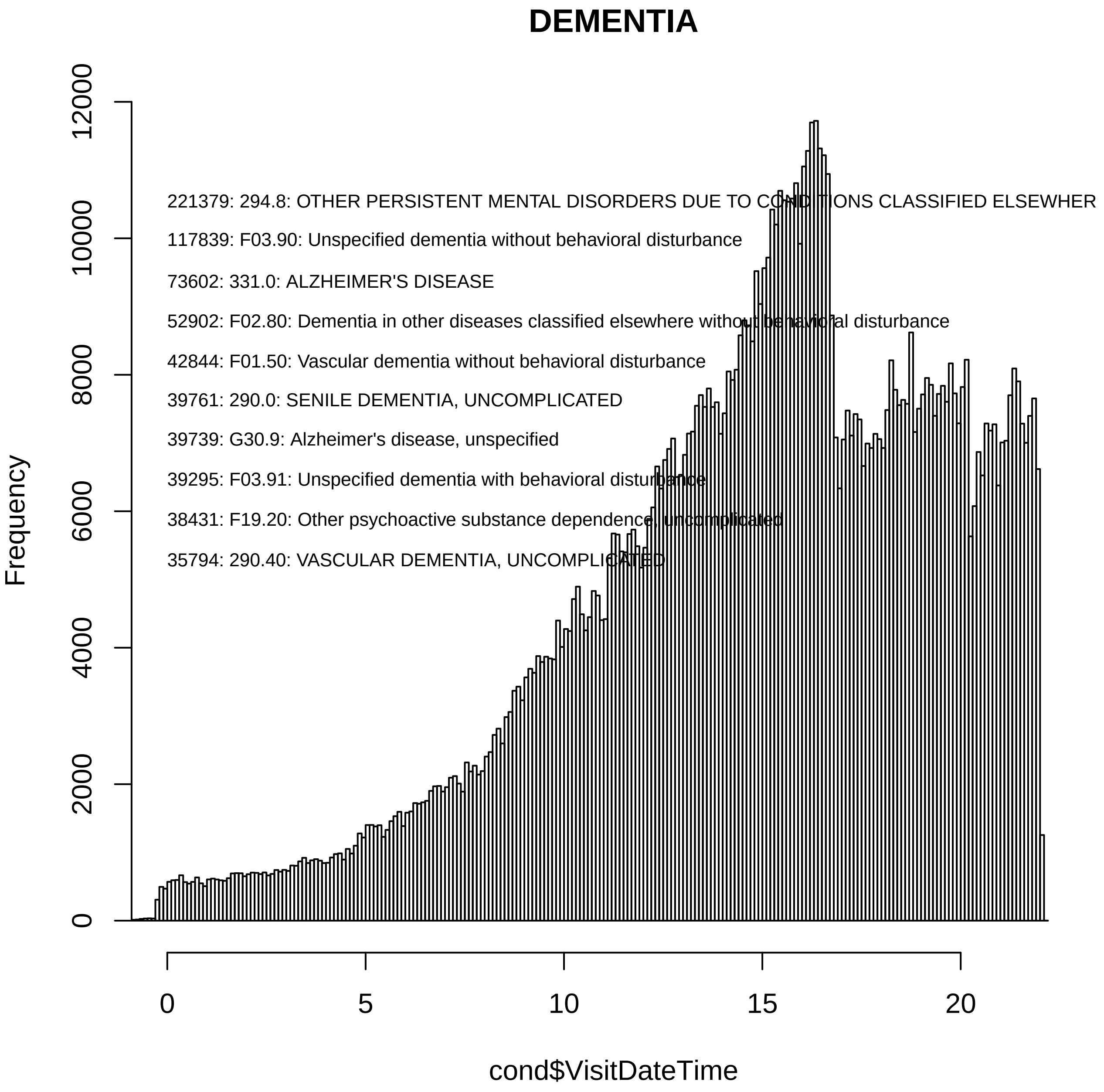

DEMENTIA
221379: 294.8: OTHER PERSISTENT MENTAL DISORDERS DUE TO CONDITIONS CLASSIFIED ELSEWHER
117839: F03.90: Unspecified dementia without behavioral disturbance
73602: 331.0: ALZHEIMER'S DISEASE
52902: F02.80: Dementia in other diseases classified elsewhere without behavioral disturbance
42844: F01.50: Vascular dementia without behavioral disturbance
39761: 290.0: SENILE DEMENTIA, UNCOMPLICATED
39739: G30.9: Alzheimer's disease, unspecified
39295: F03.91: Unspecified dementia with behavioral disturbance
38431: F19.20: Other psychoactive substance dependence, uncomplicated
35794: 290.40: VASCULAR DEMENTIA, UNCOMPLICATED
Frequency
0
2000
4000
6000
8000
10000
12000
0
5
10
15
20
cond$VisitDateTime

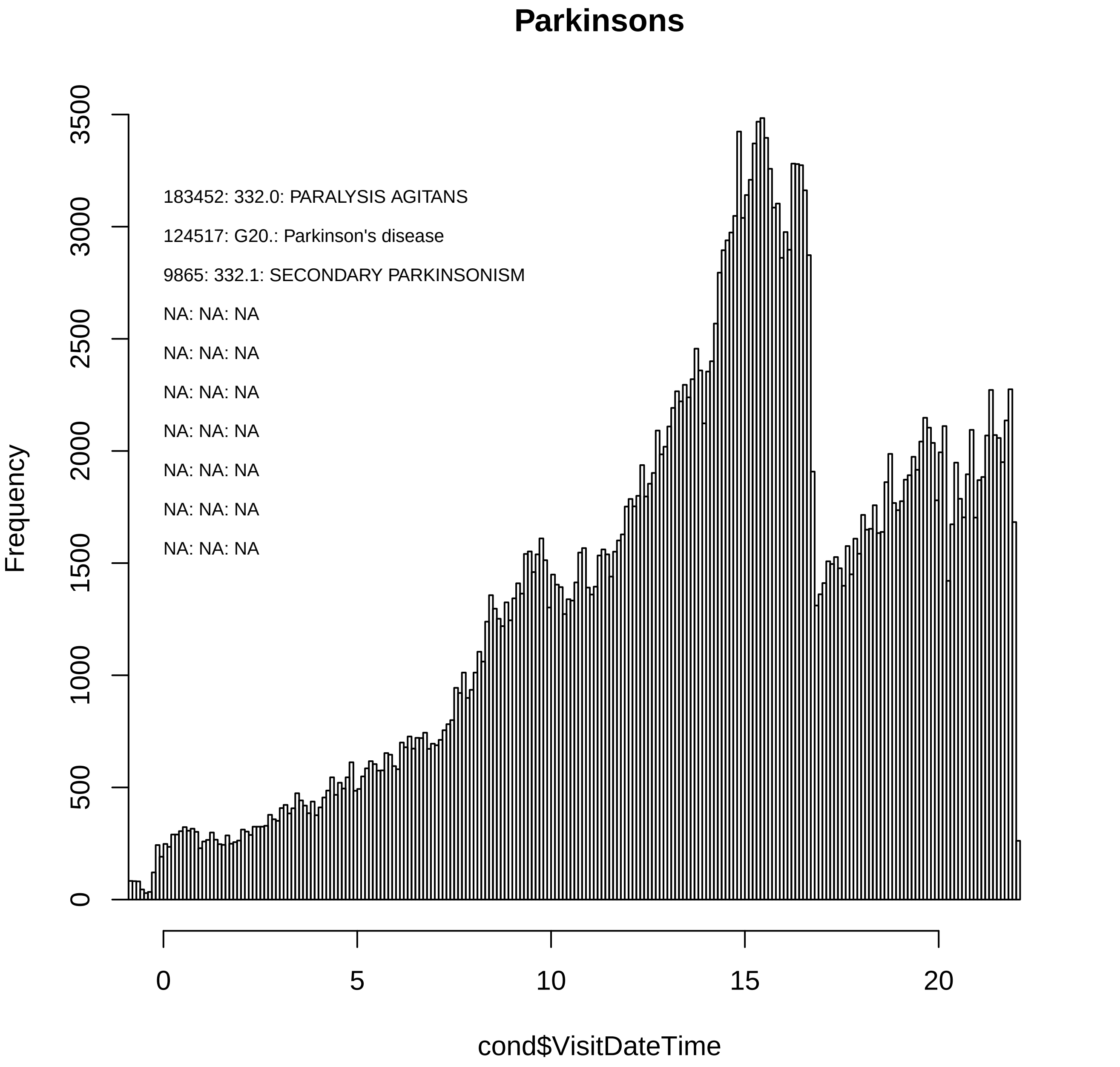
Parkinsons
Frequency
cond$VisitDateTime
0
500
1000
1500
2000
2500
3000
3500
0
5
10
15
20
183452: 332.0: PARALYSIS AGITANS
124517: G20.: Parkinson's disease
9865: 332.1: SECONDARY PARKINSONISM
NA: NA: NA
NA: NA: NA
NA: NA: NA
NA: NA: NA
NA: NA: NA
NA: NA: NA
NA: NA: NA

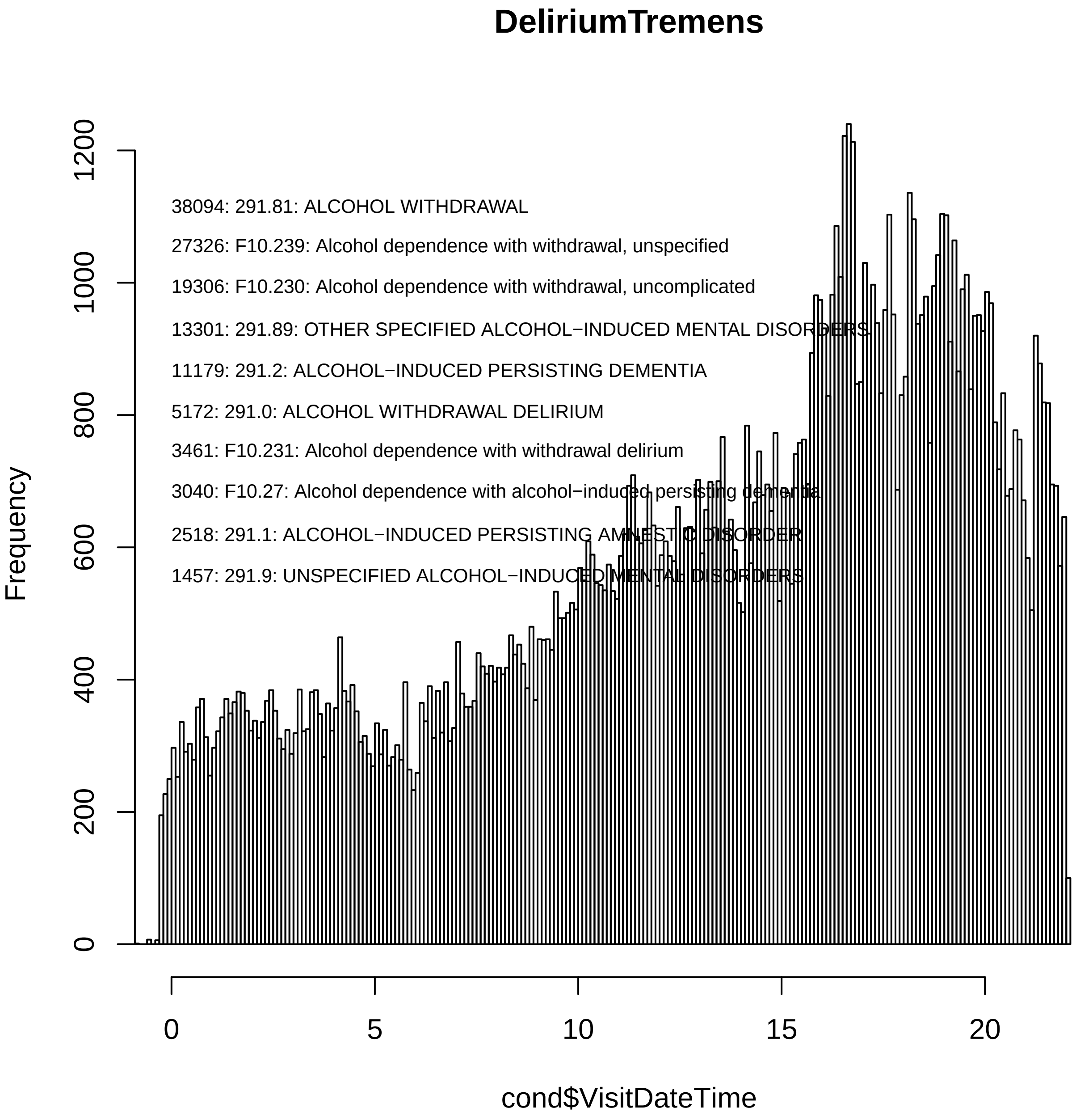
DeliriumTremens
38094: 291.81: ALCOHOL WITHDRAWAL
27326: F10.239: Alcohol dependence with withdrawal, unspecified
19306: F10.230: Alcohol dependence with withdrawal, uncomplicated
13301: 291.89: OTHER SPECIFIED ALCOHOL−INDUCED MENTAL DISORDERS
11179: 291.2: ALCOHOL−INDUCED PERSISTING DEMENTIA
5172: 291.0: ALCOHOL WITHDRAWAL DELIRIUM
3461: F10.231: Alcohol dependence with withdrawal delirium
3040: F10.27: Alcohol dependence with alcohol−induced persisting dementia
2518: 291.1: ALCOHOL−INDUCED PERSISTING AMNESTIC DISORDER
1457: 291.9: UNSPECIFIED ALCOHOL−INDUCED MENTAL DISORDERS
Frequency
0
200
400
600
800
1000
1200
0
5
10
15
20
cond$VisitDateTime

Huntington

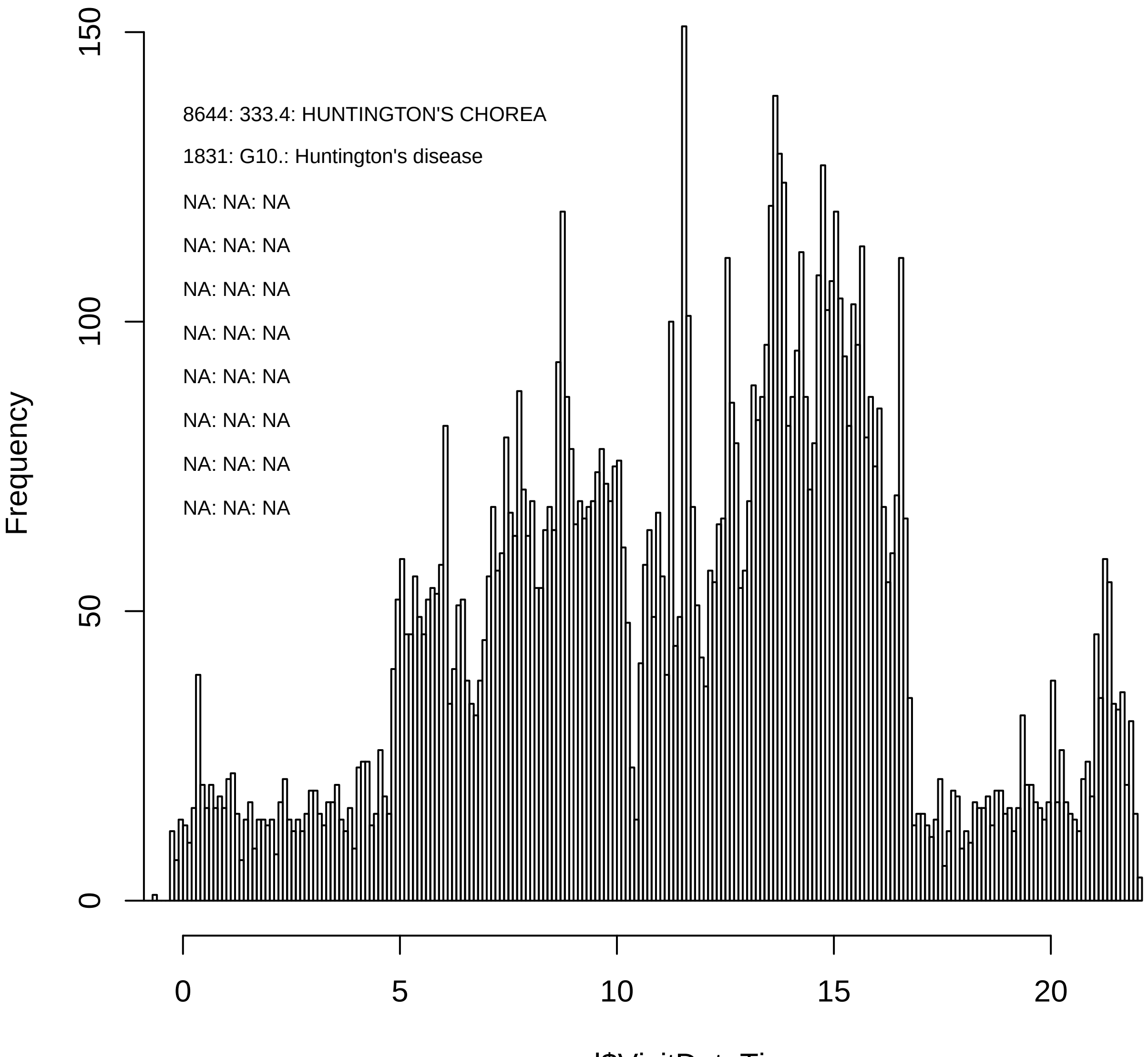

8644: 333.4: HUNTINGTON'S CHOREA
1831: G10.: Huntington's disease
NA: NA: NA
NA: NA: NA
NA: NA: NA
NA: NA: NA
NA: NA: NA
NA: NA: NA
NA: NA: NA
NA: NA: NA
Frequency
150
100
50
0
0
5
10
15
20
cond$VisitDateTime

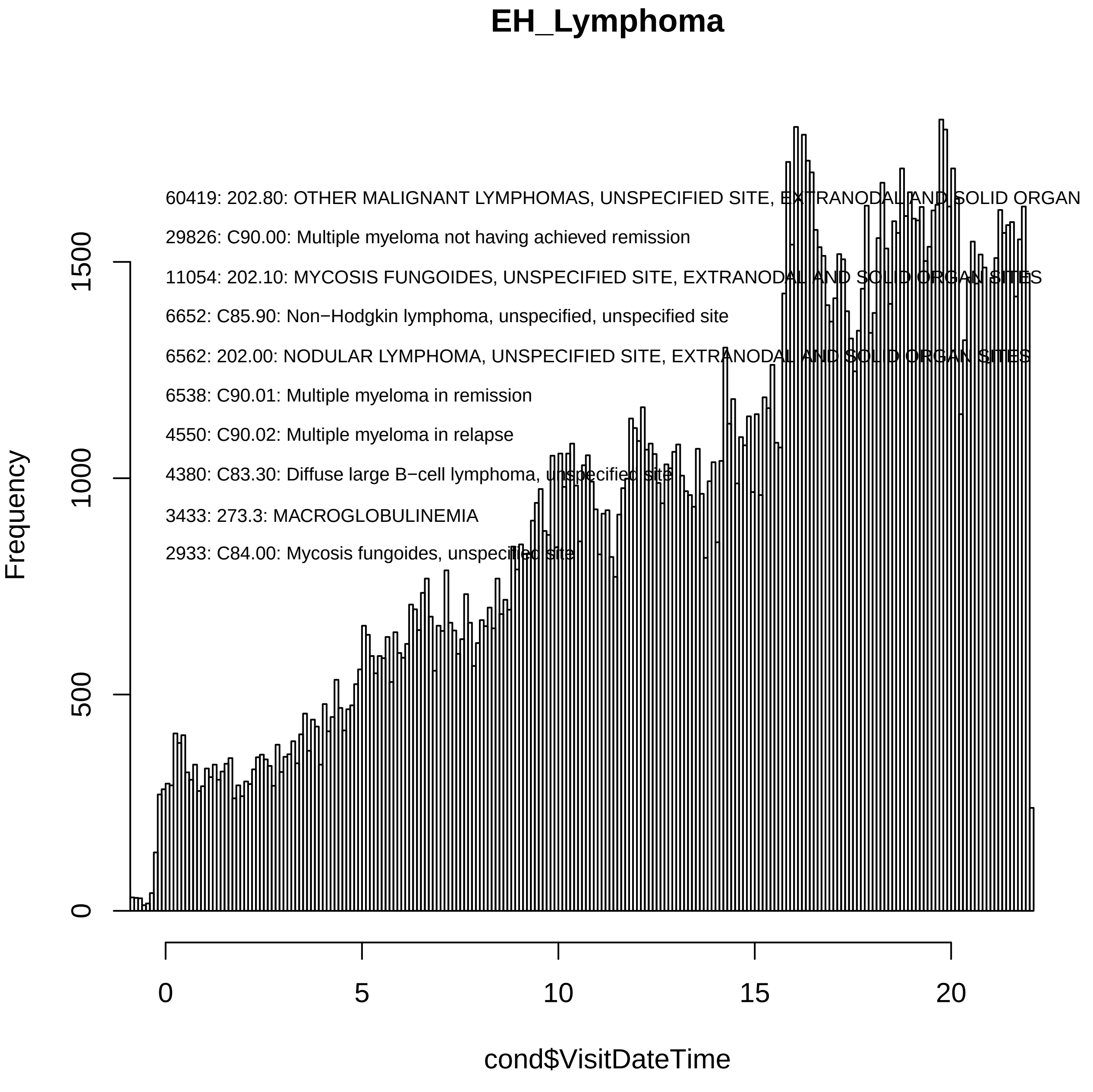
EH_Lymphoma
60419: 202.80: OTHER MALIGNANT LYMPHOMAS, UNSPECIFIED SITE, EXTRANODAL AND SOLID ORGAN
29826: C90.00: Multiple myeloma not having achieved remission
11054: 202.10: MYCOSIS FUNGOIDES, UNSPECIFIED SITE, EXTRANODAL AND SOLID ORGAN SITES
6652: C85.90: Non−Hodgkin lymphoma, unspecified, unspecified site
6562: 202.00: NODULAR LYMPHOMA, UNSPECIFIED SITE, EXTRANODAL AND SOLID ORGAN SITES
6538: C90.01: Multiple myeloma in remission
4550: C90.02: Multiple myeloma in relapse
4380: C83.30: Diffuse large B−cell lymphoma, unspecified site
3433: 273.3: MACROGLOBULINEMIA
2933: C84.00: Mycosis fungoides, unspecified site
Frequency
0
500
1000
1500
0
5
10
15
20
cond$VisitDateTime

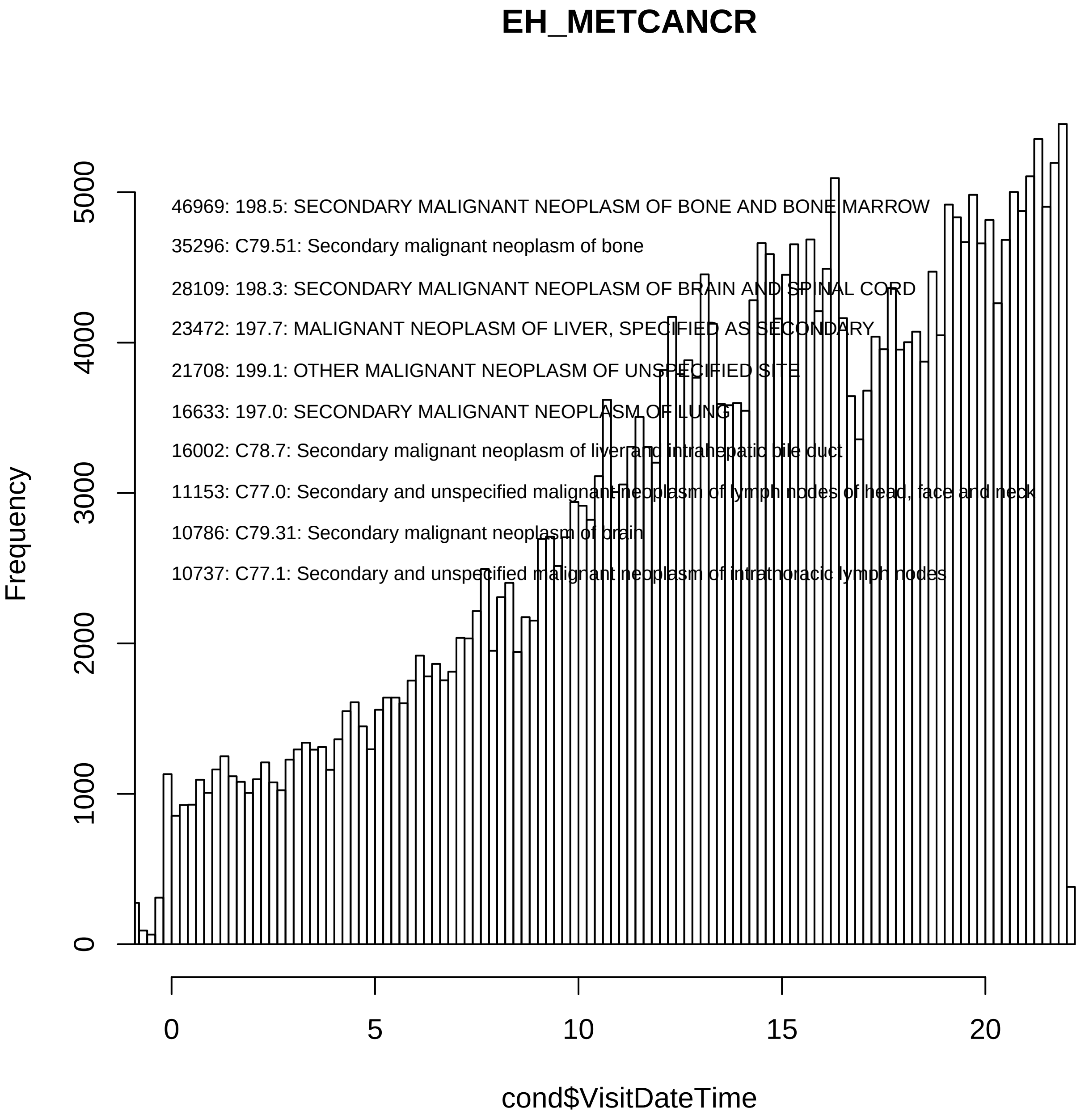
EH_METCANCR
46969: 198.5: SECONDARY MALIGNANT NEOPLASM OF BONE AND BONE MARROW
35296: C79.51: Secondary malignant neoplasm of bone
28109: 198.3: SECONDARY MALIGNANT NEOPLASM OF BRAIN AND SPINAL CORD
23472: 197.7: MALIGNANT NEOPLASM OF LIVER, SPECIFIED AS SECONDARY
21708: 199.1: OTHER MALIGNANT NEOPLASM OF UNSPECIFIED SITE
16633: 197.0: SECONDARY MALIGNANT NEOPLASM OF LUNG
16002: C78.7: Secondary malignant neoplasm of liver and intrahepatic bile duct
11153: C77.0: Secondary and unspecified malignant neoplasm of lymph nodes of head, face and neck
10786: C79.31: Secondary malignant neoplasm of brain
10737: C77.1: Secondary and unspecified malignant neoplasm of intrathoracic lymph nodes
Frequency
0
1000
2000
3000
4000
5000
0
5
10
15
20
cond$VisitDateTime

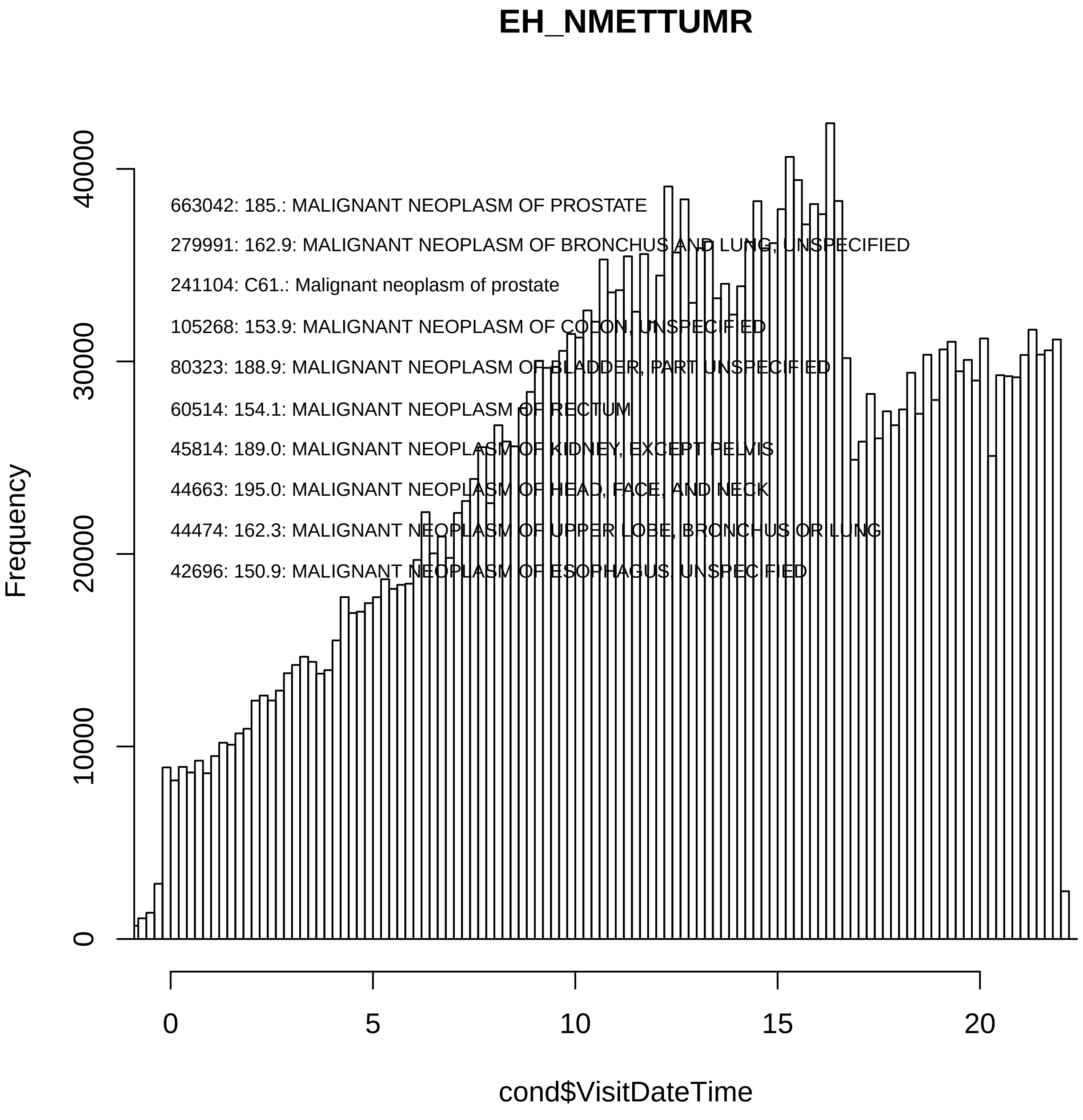
EH_NMETTUMR
663042: 185.: MALIGNANT NEOPLASM OF PROSTATE
279991: 162.9: MALIGNANT NEOPLASM OF BRONCHUS AND LUNG, UNSPECIFIED
241104: C61.: Malignant neoplasm of prostate
105268: 153.9: MALIGNANT NEOPLASM OF COLON, UNSPECIFIED
80323: 188.9: MALIGNANT NEOPLASM OF BLADDER, PART UNSPECIFIED
60514: 154.1: MALIGNANT NEOPLASM OF RECTUM
45814: 189.0: MALIGNANT NEOPLASM OF KIDNEY, EXCEPT PELVIS
44663: 195.0: MALIGNANT NEOPLASM OF HEAD, FACE, AND NECK
44474: 162.3: MALIGNANT NEOPLASM OF UPPER LOBE, BRONCHUS OR LUNG
42696: 150.9: MALIGNANT NEOPLASM OF ESOPHAGUS, UNSPECIFIED
Frequency
0
10000
20000
30000
40000
0
5
10
15
20
cond$VisitDateTime

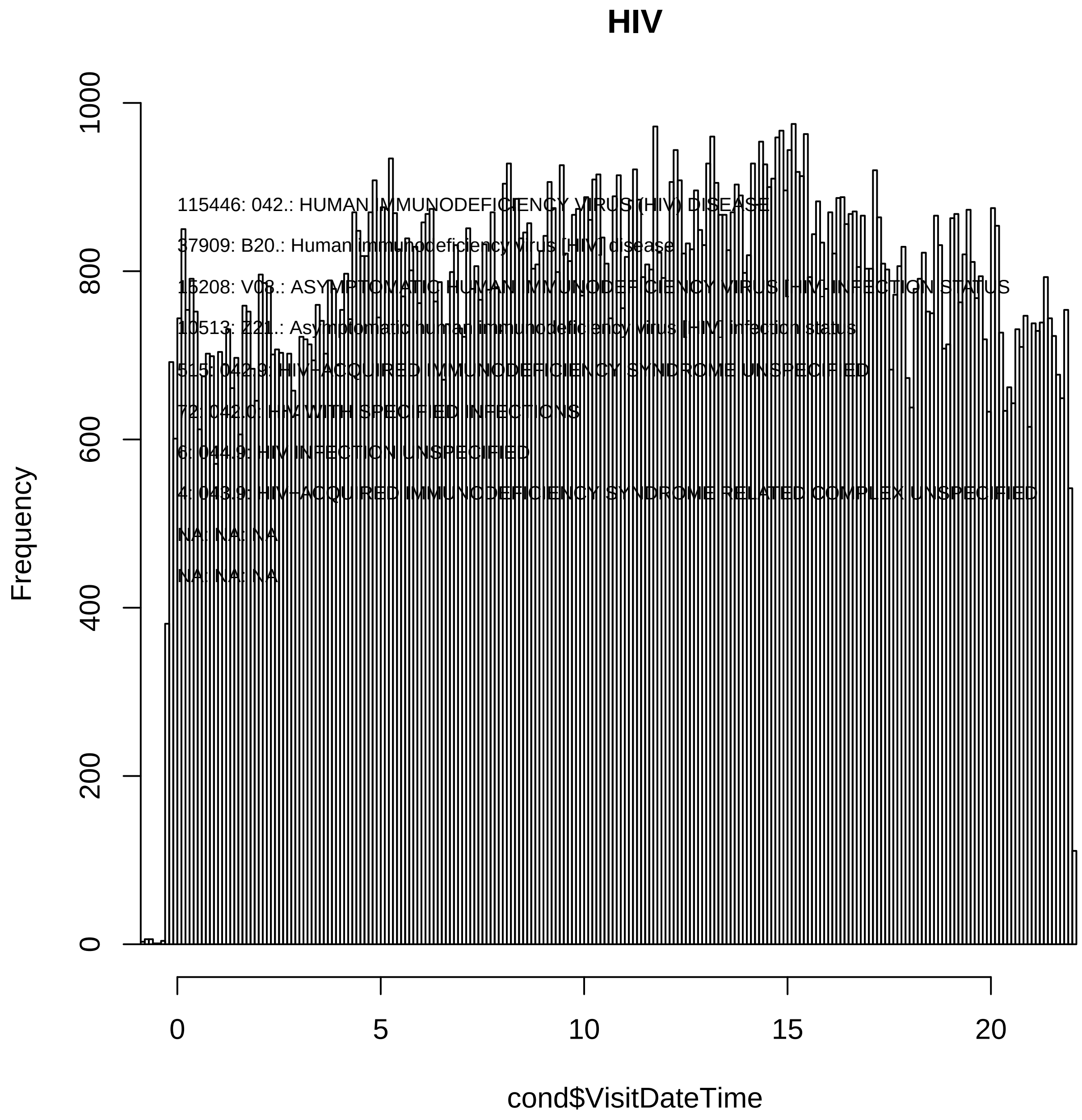
HIV
Frequency
cond$VisitDateTime
0
200
400
600
800
1000
0
5
10
15
20
115446: 042.: HUMAN IMMUNODEFICIENCY VIRUS (HIV) DISEASE
37909: B20.: Human immunodeficiency virus [HIV] disease
15208: V08.: ASYMPTOMATIC HUMAN IMMUNODEFICIENCY VIRUS [HIV] INFECTION STATUS
10513: Z21.: Asymptomatic human immunodeficiency virus [HIV] infection status
515: 042.9: HIV-ACQUIRED IMMUNODEFICIENCY SYNDROME UNSPECIFIED
72: 042.0: HIV WITH SPECIFIED INFECTIONS
6: 044.9: HIV INFECTION UNSPECIFIED
4: 043.9: HIV-ACQUIRED IMMUNODEFICIENCY SYNDROME RELATED COMPLEX UNSPECIFIED
NA: NA: NA
NA: NA: NA

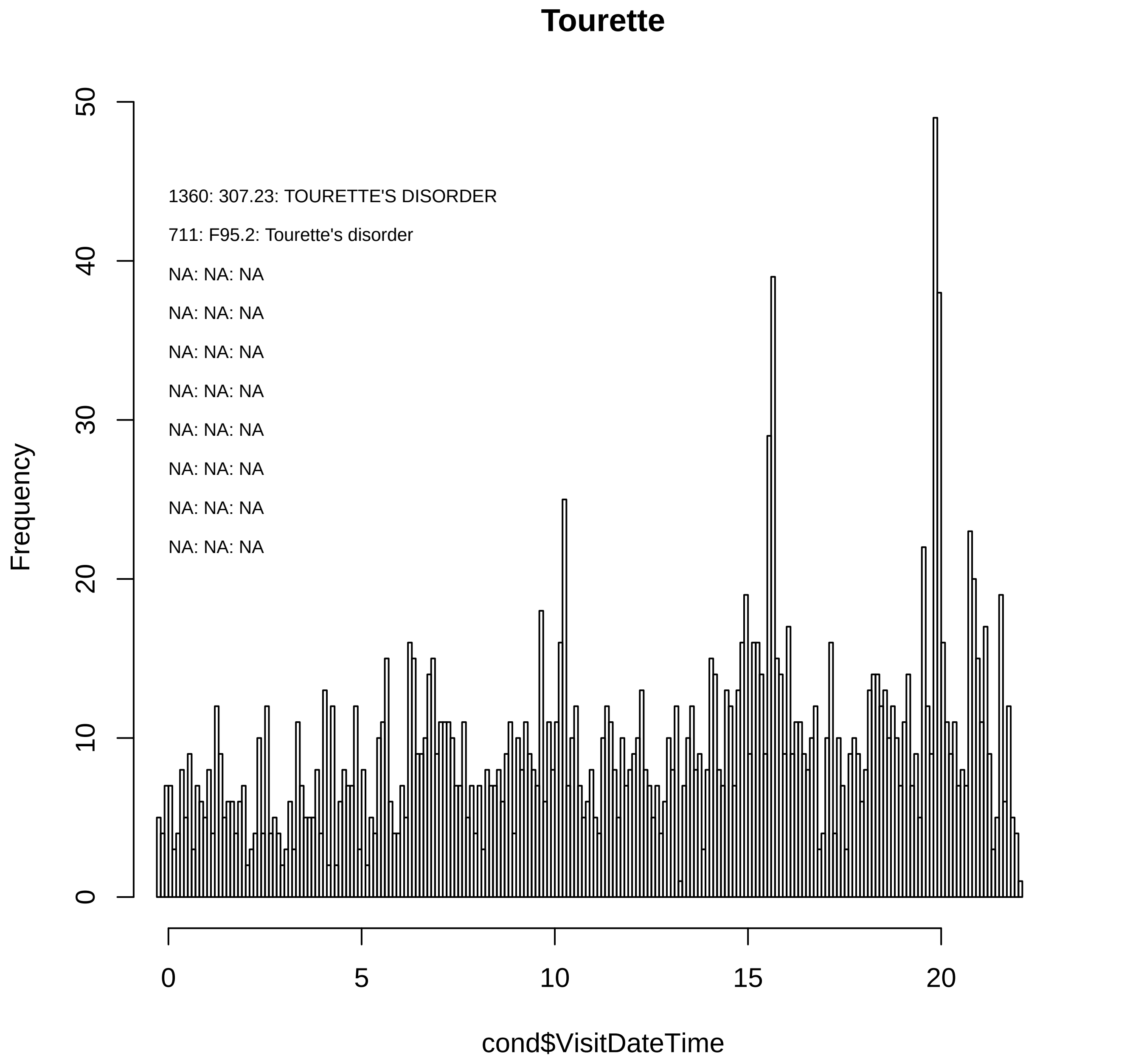
Tourette
1360: 307.23: TOURETTE'S DISORDER
711: F95.2: Tourette's disorder
NA: NA: NA
NA: NA: NA
NA: NA: NA
NA: NA: NA
NA: NA: NA
NA: NA: NA
NA: NA: NA
NA: NA: NA
Frequency
0
10
20
30
40
50
0
5
10
15
20
cond$VisitDateTime

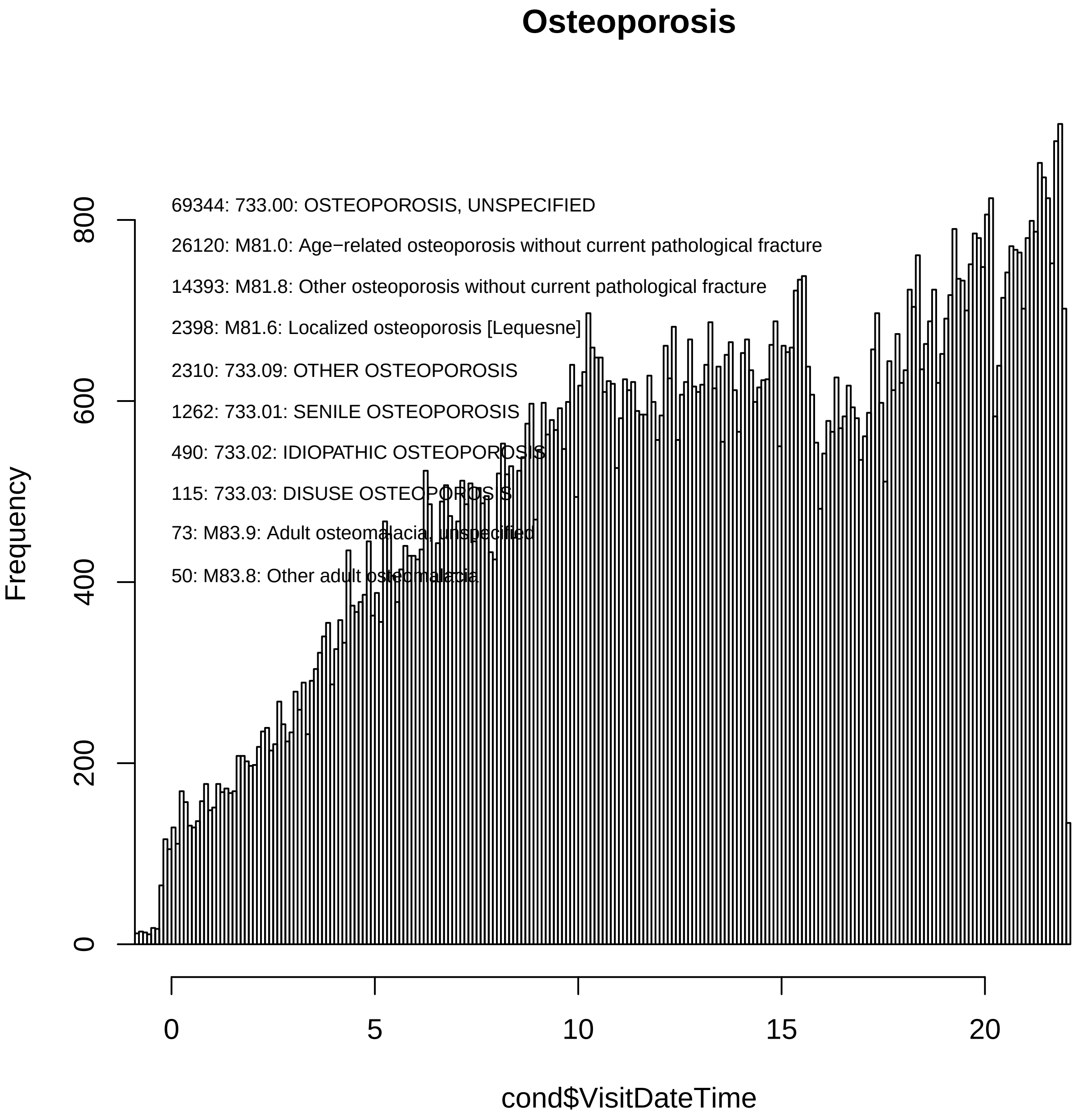

Osteoporosis
69344: 733.00: OSTEOPOROSIS, UNSPECIFIED
26120: M81.0: Age−related osteoporosis without current pathological fracture
14393: M81.8: Other osteoporosis without current pathological fracture
2398: M81.6: Localized osteoporosis [Lequesne]
2310: 733.09: OTHER OSTEOPOROSIS
1262: 733.01: SENILE OSTEOPOROSIS
490: 733.02: IDIOPATHIC OSTEOPOROSIS
115: 733.03: DISUSE OSTEOPOROSIS
73: M83.9: Adult osteomalacia, unspecified
50: M83.8: Other adult osteomalacia
Frequency
0
200
400
600
800
0
5
10
15
20
cond$VisitDateTime

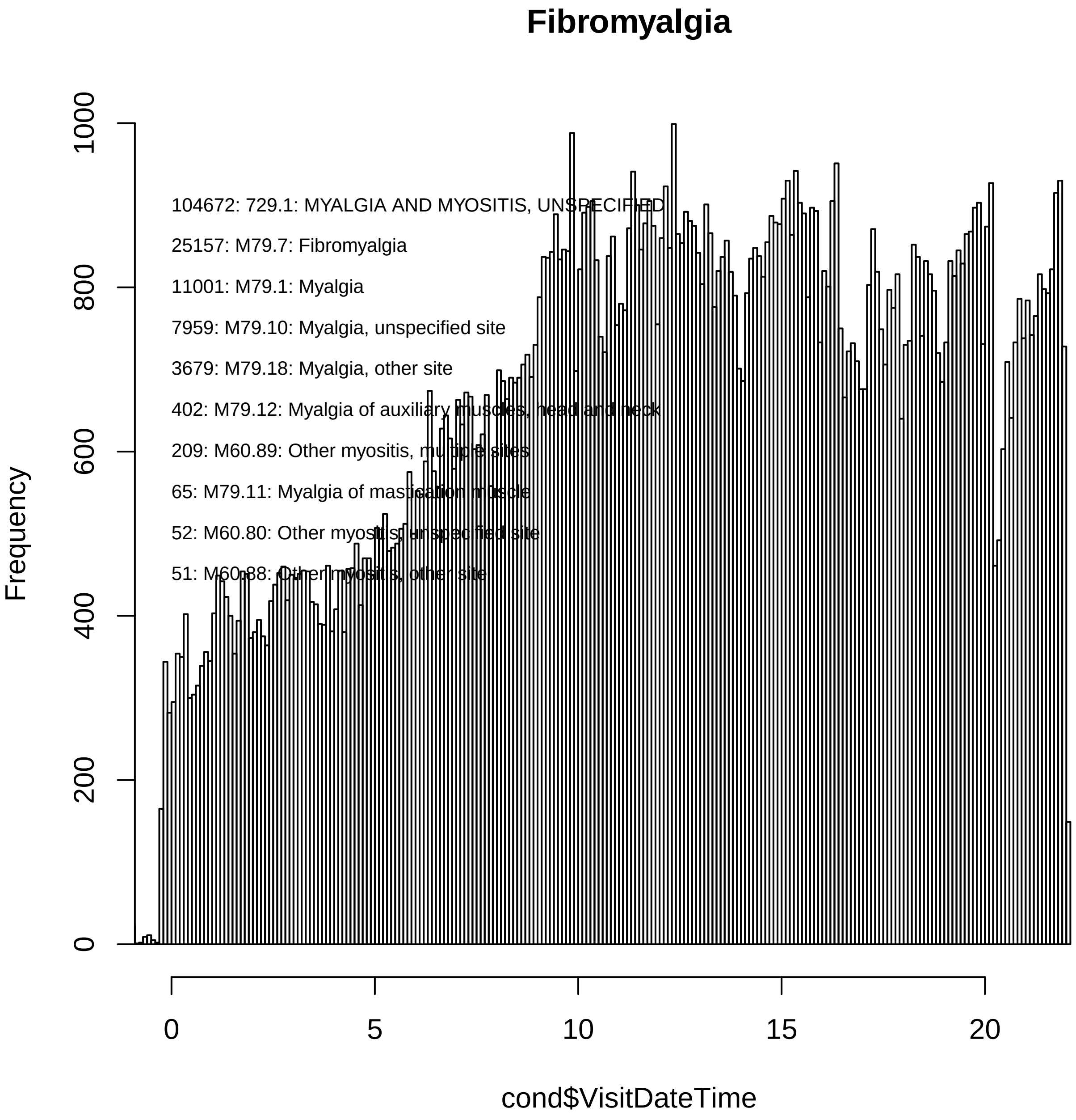
Fibromyalgia
Frequency
cond$VisitDateTime
0
200
400
600
800
1000
0
5
10
15
20
104672: 729.1: MYALGIA AND MYOSITIS, UNSPECIFIED
25157: M79.7: Fibromyalgia
11001: M79.1: Myalgia
7959: M79.10: Myalgia, unspecified site
3679: M79.18: Myalgia, other site
402: M79.12: Myalgia of auxiliary muscles, head and neck
209: M60.89: Other myositis, multiple sites
65: M79.11: Myalgia of mastication muscle
52: M60.80: Other myositis, unspecified site
51: M60.88: Other myositis, other site

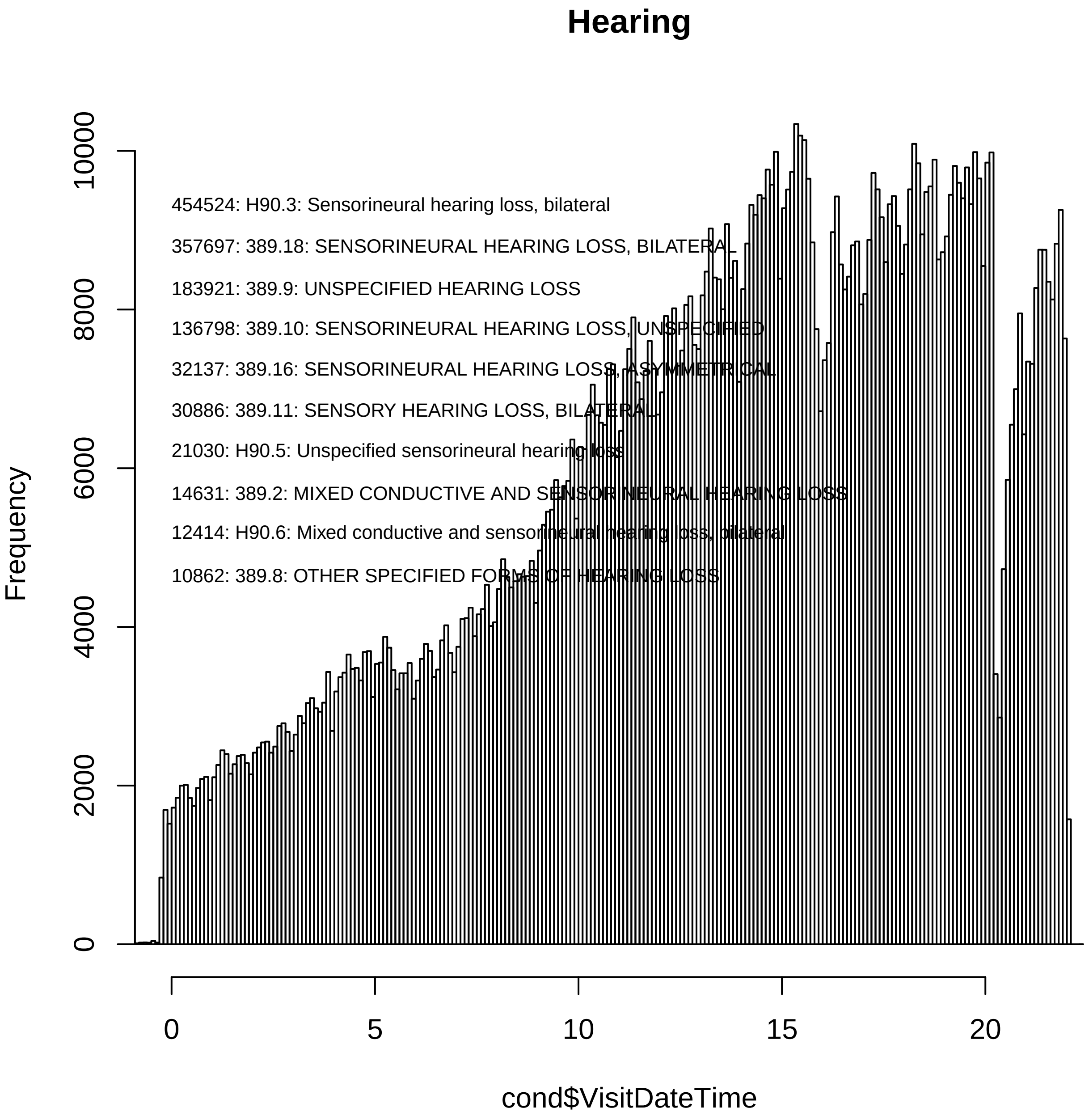
Hearing
454524: H90.3: Sensorineural hearing loss, bilateral
357697: 389.18: SENSORINEURAL HEARING LOSS, BILATERAL
183921: 389.9: UNSPECIFIED HEARING LOSS
136798: 389.10: SENSORINEURAL HEARING LOSS, UNSPECIFIED
32137: 389.16: SENSORINEURAL HEARING LOSS, ASYMMETRICAL
30886: 389.11: SENSORY HEARING LOSS, BILATERAL
21030: H90.5: Unspecified sensorineural hearing loss
14631: 389.2: MIXED CONDUCTIVE AND SENSORINEURAL HEARING LOSS
12414: H90.6: Mixed conductive and sensorineural hearing loss, bilateral
10862: 389.8: OTHER SPECIFIED FORMS OF HEARING LOSS
Frequency
0
2000
4000
6000
8000
10000
0
5
10
15
20
cond$VisitDateTime

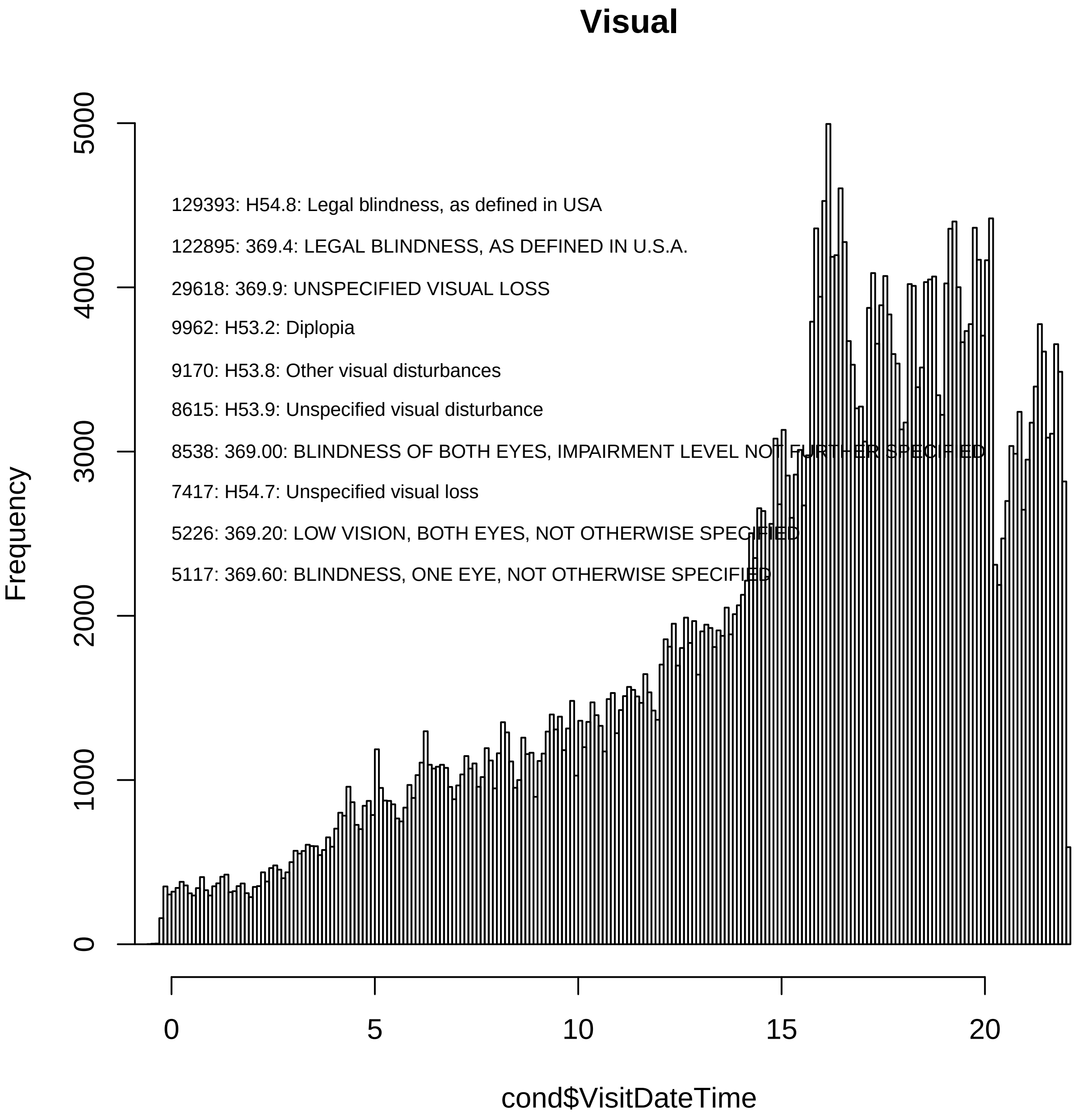
Visual
Frequency
cond$VisitDateTime
0
1000
2000
3000
4000
5000
0
5
10
15
20
129393: H54.8: Legal blindness, as defined in USA
122895: 369.4: LEGAL BLINDNESS, AS DEFINED IN U.S.A.
29618: 369.9: UNSPECIFIED VISUAL LOSS
9962: H53.2: Diplopia
9170: H53.8: Other visual disturbances
8615: H53.9: Unspecified visual disturbance
8538: 369.00: BLINDNESS OF BOTH EYES, IMPAIRMENT LEVEL NOT FURTHER SPECIFIED
7417: H54.7: Unspecified visual loss
5226: 369.20: LOW VISION, BOTH EYES, NOT OTHERWISE SPECIFIED
5117: 369.60: BLINDNESS, ONE EYE, NOT OTHERWISE SPECIFIED

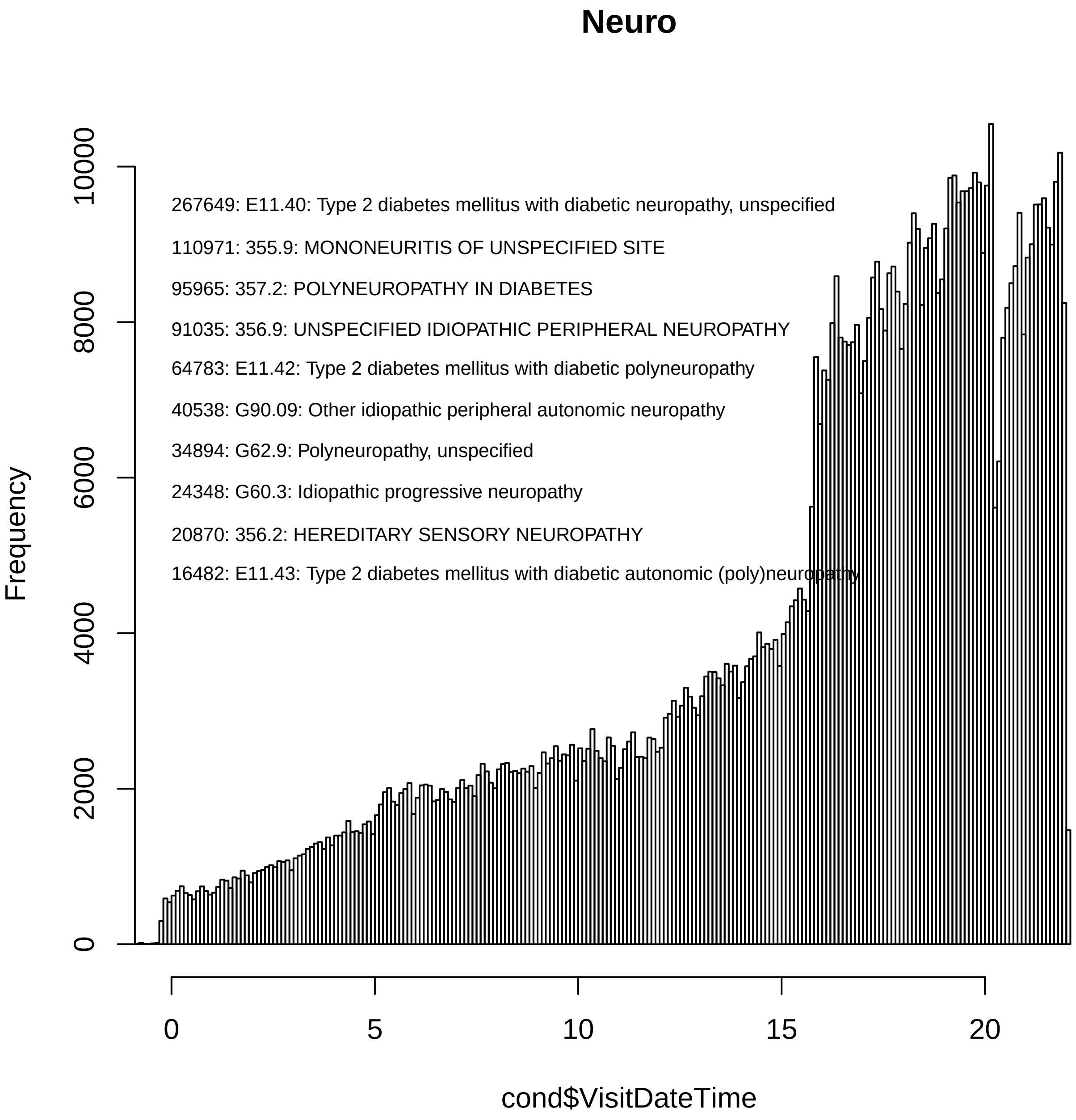
Neuro
267649: E11.40: Type 2 diabetes mellitus with diabetic neuropathy, unspecified
110971: 355.9: MONONEURITIS OF UNSPECIFIED SITE
95965: 357.2: POLYNEUROPATHY IN DIABETES
91035: 356.9: UNSPECIFIED IDIOPATHIC PERIPHERAL NEUROPATHY
64783: E11.42: Type 2 diabetes mellitus with diabetic polyneuropathy
40538: G90.09: Other idiopathic peripheral autonomic neuropathy
34894: G62.9: Polyneuropathy, unspecified
24348: G60.3: Idiopathic progressive neuropathy
20870: 356.2: HEREDITARY SENSORY NEUROPATHY
16482: E11.43: Type 2 diabetes mellitus with diabetic autonomic (poly)neuropathy
Frequency
0
2000
4000
6000
8000
10000
0
5
10
15
20
cond$VisitDateTime

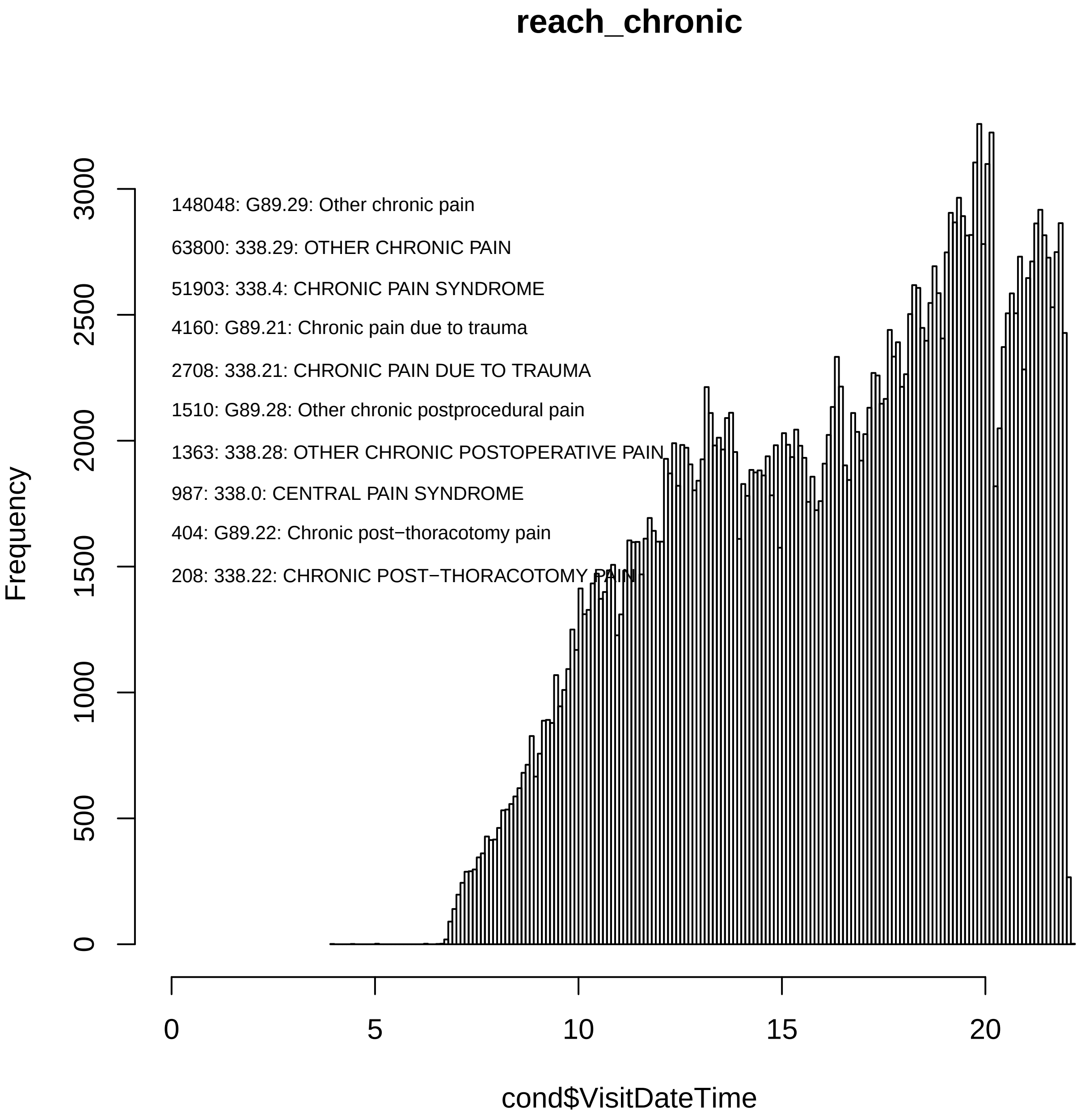

reach_chronic
148048: G89.29: Other chronic pain
63800: 338.29: OTHER CHRONIC PAIN
51903: 338.4: CHRONIC PAIN SYNDROME
4160: G89.21: Chronic pain due to trauma
2708: 338.21: CHRONIC PAIN DUE TO TRAUMA
1510: G89.28: Other chronic postprocedural pain
1363: 338.28: OTHER CHRONIC POSTOPERATIVE PAIN
987: 338.0: CENTRAL PAIN SYNDROME
404: G89.22: Chronic post−thoracotomy pain
208: 338.22: CHRONIC POST−THORACOTOMY PAIN
Frequency
0
500
1000
1500
2000
2500
3000
0
5
10
15
20
cond$VisitDateTime

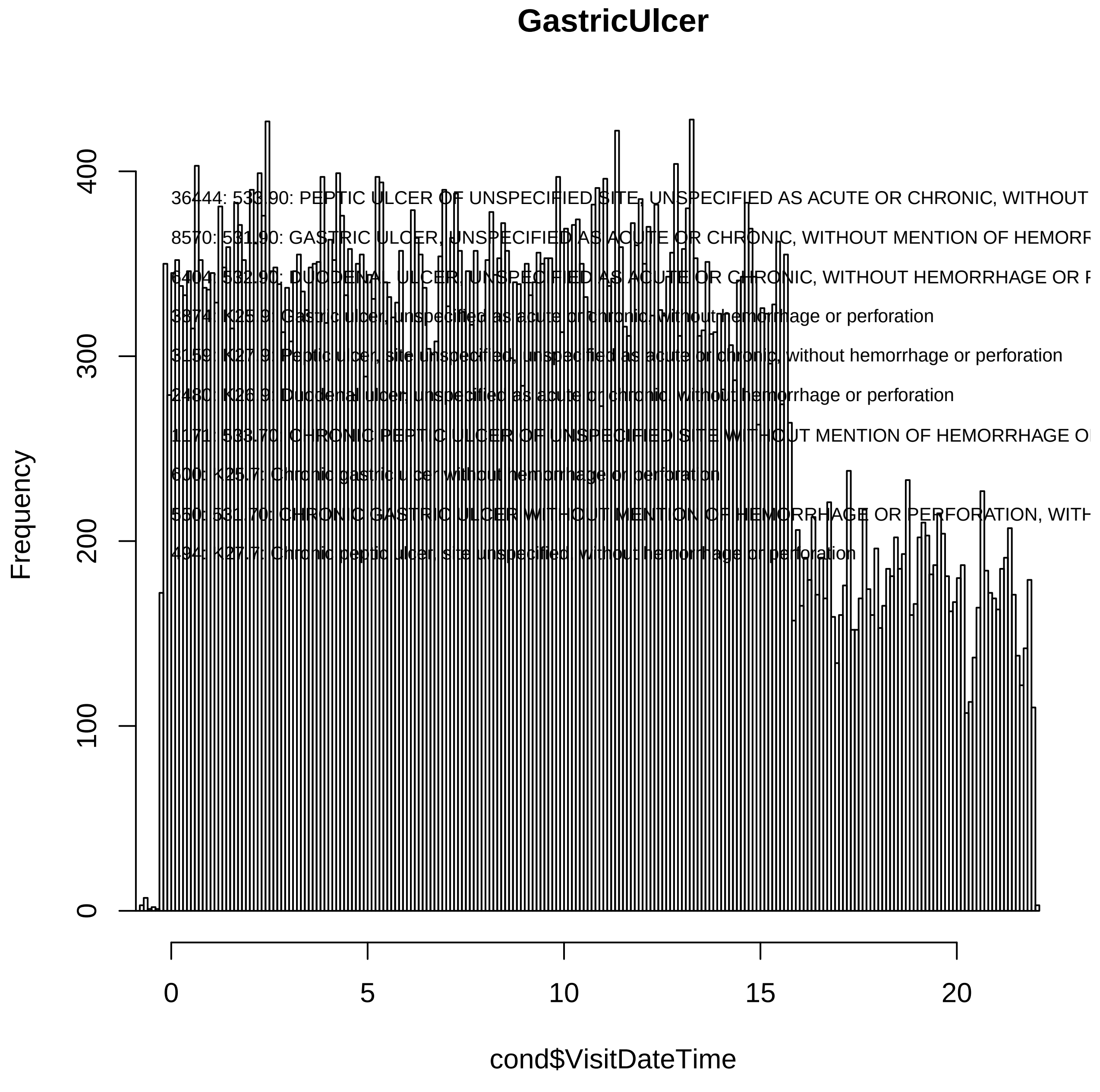
GastricUlcer
Frequency
cond$VisitDateTime
0
100
200
300
400
0
5
10
15
20
36444: 533.90: PEPTIC ULCER OF UNSPECIFIED SITE, UNSPECIFIED AS ACUTE OR CHRONIC, WITHOUT
8570: 531.90: GASTRIC ULCER, UNSPECIFIED AS ACUTE OR CHRONIC, WITHOUT MENTION OF HEMORR
6404: 532.90: DUODENAL ULCER, UNSPECIFIED AS ACUTE OR CHRONIC, WITHOUT HEMORRHAGE OR P
3874: K25.9: Gastric ulcer, unspecified as acute or chronic, without hemorrhage or perforation
3159: K27.9: Peptic ulcer, site unspecified, unspecified as acute or chronic, without hemorrhage or perforation
2480: K26.9: Duodenal ulcer, unspecified as acute or chronic, without hemorrhage or perforation
1171: 533.70: CHRONIC PEPTIC ULCER OF UNSPECIFIED SITE WITHOUT MENTION OF HEMORRHAGE O
600: K25.7: Chronic gastric ulcer without hemorrhage or perforation
550: 531.70: CHRONIC GASTRIC ULCER WITHOUT MENTION OF HEMORRHAGE OR PERFORATION, WITH
494: K27.7: Chronic peptic ulcer, site unspecified, without hemorrhage or perforation

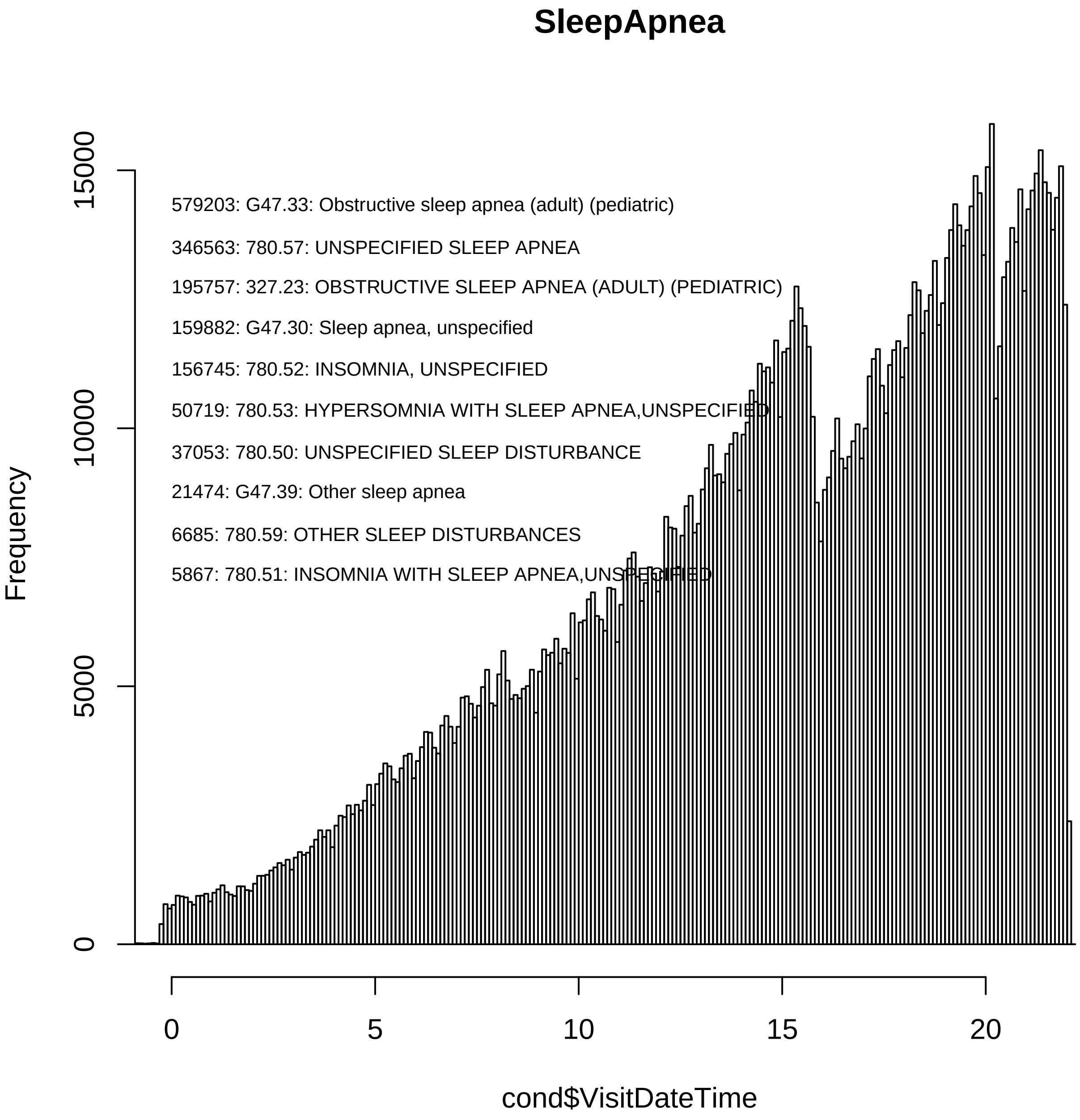
SleepApnea
579203: G47.33: Obstructive sleep apnea (adult) (pediatric)
346563: 780.57: UNSPECIFIED SLEEP APNEA
195757: 327.23: OBSTRUCTIVE SLEEP APNEA (ADULT) (PEDIATRIC)
159882: G47.30: Sleep apnea, unspecified
156745: 780.52: INSOMNIA, UNSPECIFIED
50719: 780.53: HYPERSOMNIA WITH SLEEP APNEA,UNSPECIFIED
37053: 780.50: UNSPECIFIED SLEEP DISTURBANCE
21474: G47.39: Other sleep apnea
6685: 780.59: OTHER SLEEP DISTURBANCES
5867: 780.51: INSOMNIA WITH SLEEP APNEA,UNSPECIFIED
Frequency
0
5000
10000
15000
0
5
10
15
20
cond$VisitDateTime

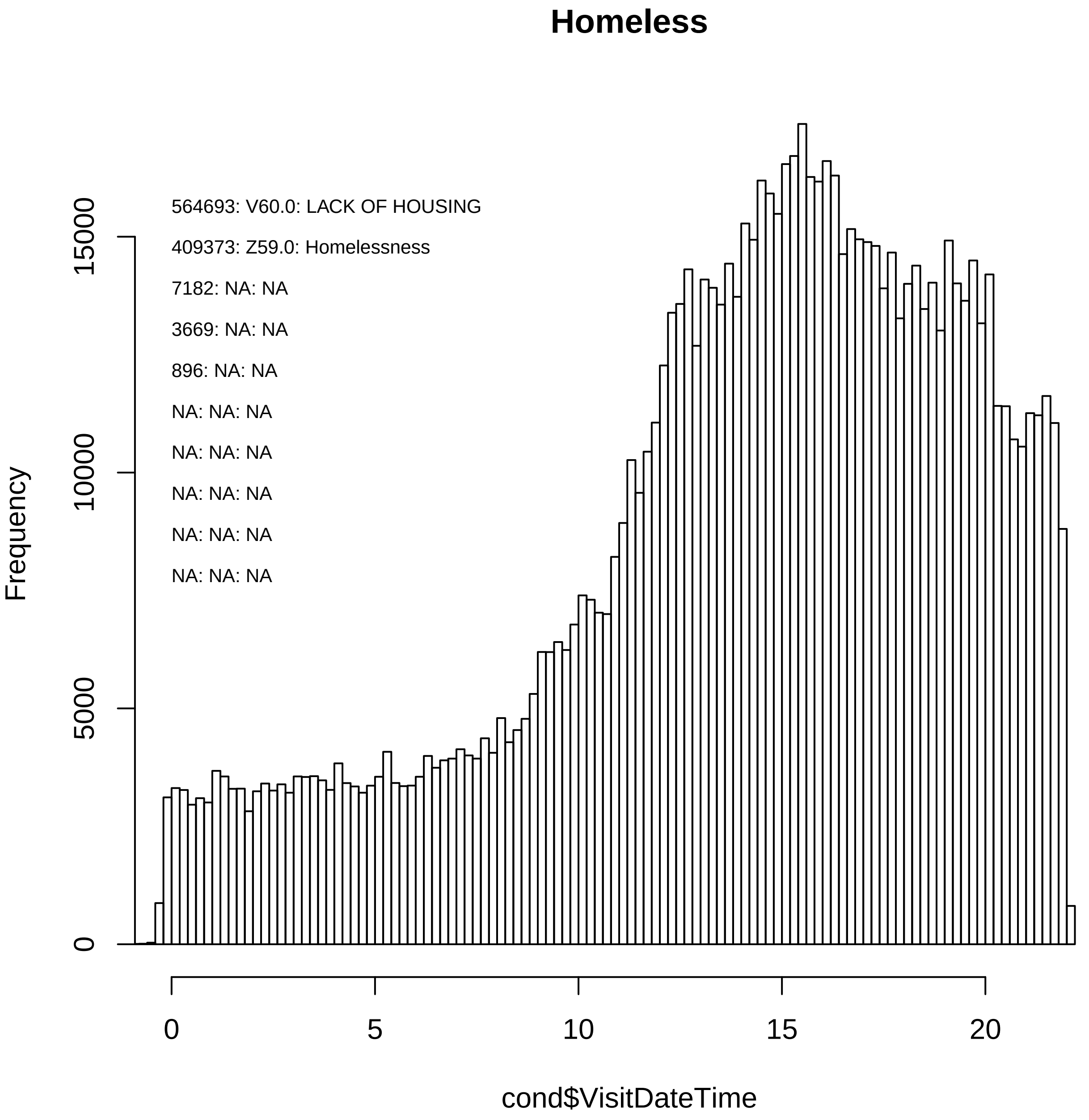
Homeless
564693: V60.0: LACK OF HOUSING
409373: Z59.0: Homelessness
7182: NA: NA
3669: NA: NA
896: NA: NA
NA: NA: NA
NA: NA: NA
NA: NA: NA
NA: NA: NA
NA: NA: NA
Frequency
0
5000
10000
15000
0
5
10
15
20
cond$VisitDateTime

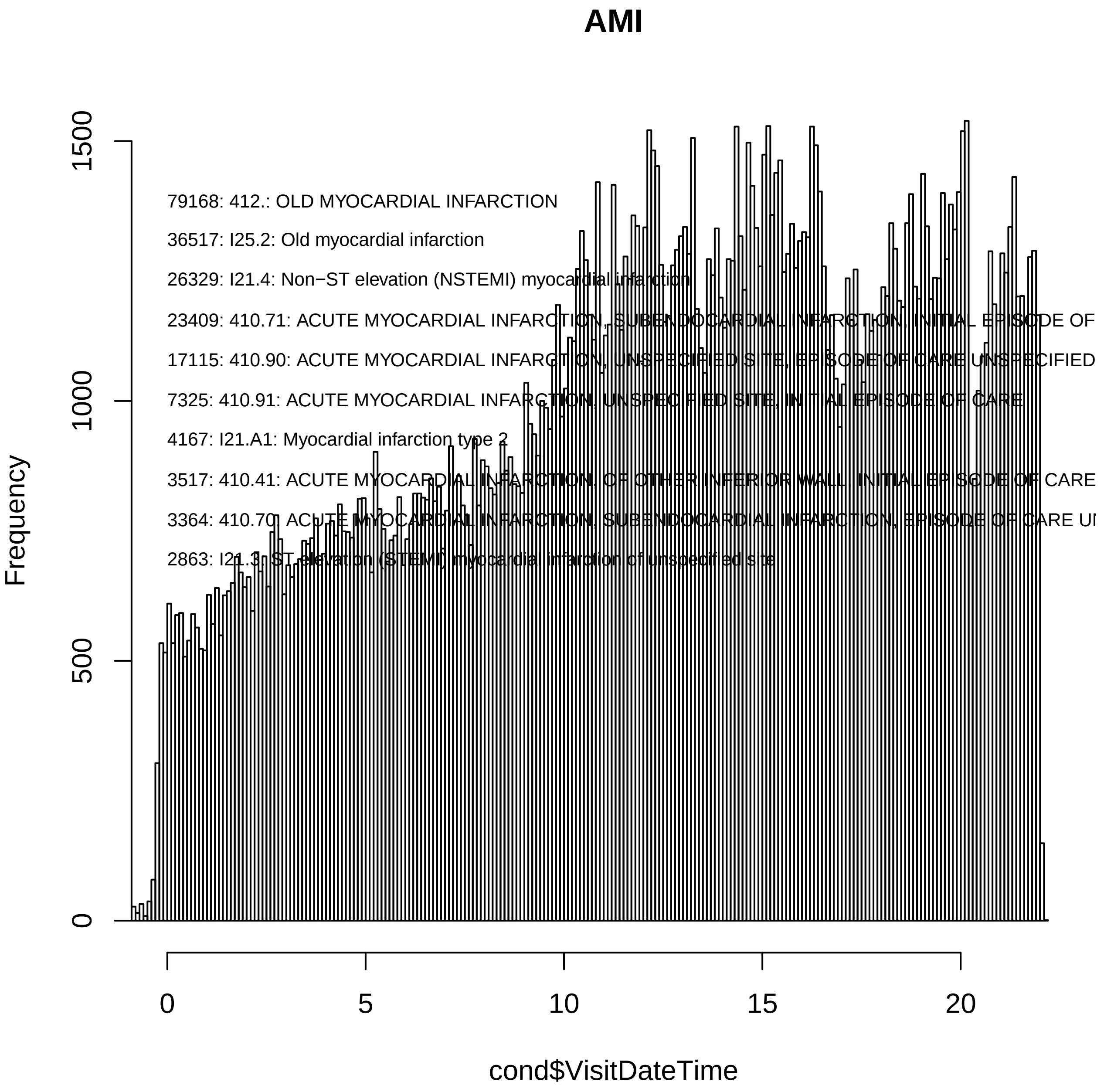
AMI
Frequency
0
500
1000
1500
cond$VisitDateTime
0
5
10
15
20
79168: 412.: OLD MYOCARDIAL INFARCTION
36517: I25.2: Old myocardial infarction
26329: I21.4: Non−ST elevation (NSTEMI) myocardial infarction
23409: 410.71: ACUTE MYOCARDIAL INFARCTION, SUBENDOCARDIAL INFARCTION, INITIAL EPISODE OF
17115: 410.90: ACUTE MYOCARDIAL INFARCTION, UNSPECIFIED SITE, EPISODE OF CARE UNSPECIFIED
7325: 410.91: ACUTE MYOCARDIAL INFARCTION, UNSPECIFIED SITE, INITIAL EPISODE OF CARE
4167: I21.A1: Myocardial infarction type 2
3517: 410.41: ACUTE MYOCARDIAL INFARCTION, OF OTHER INFERIOR WALL, INITIAL EPISODE OF CARE
3364: 410.70: ACUTE MYOCARDIAL INFARCTION, SUBENDOCARDIAL INFARCTION, EPISODE OF CARE UI
2863: I21.3: ST elevation (STEMI) myocardial infarction of unspecified site

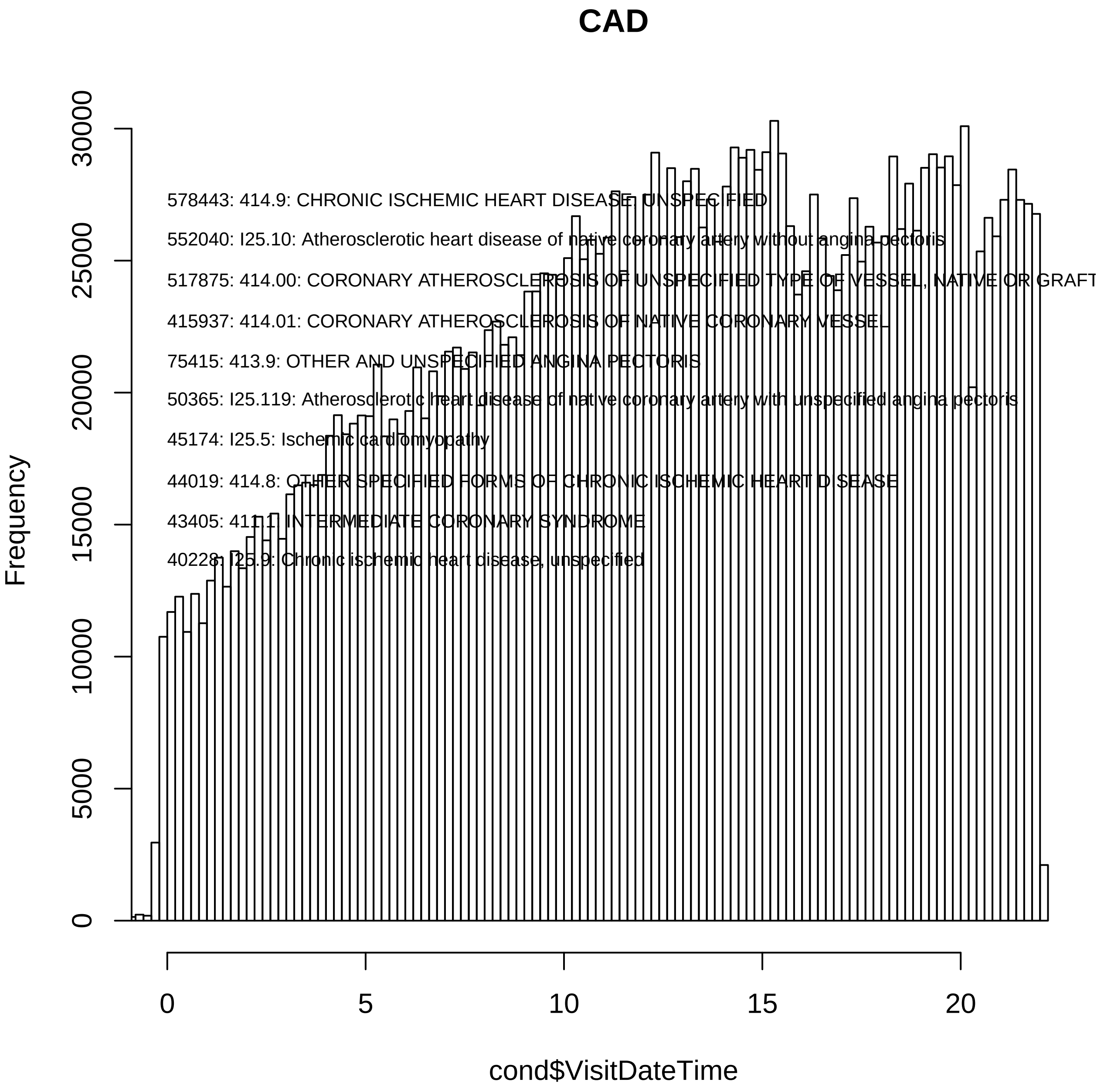
CAD
578443: 414.9: CHRONIC ISCHEMIC HEART DISEASE, UNSPECIFIED
552040: I25.10: Atherosclerotic heart disease of native coronary artery without angina pectoris
517875: 414.00: CORONARY ATHEROSCLEROSIS OF UNSPECIFIED TYPE OF VESSEL, NATIVE OR GRAFT
415937: 414.01: CORONARY ATHEROSCLEROSIS OF NATIVE CORONARY VESSEL
75415: 413.9: OTHER AND UNSPECIFIED ANGINA PECTORIS
50365: I25.119: Atherosclerotic heart disease of native coronary artery with unspecified angina pectoris
45174: I25.5: Ischemic cardiomyopathy
44019: 414.8: OTHER SPECIFIED FORMS OF CHRONIC ISCHEMIC HEART DISEASE
43405: 411.1: INTERMEDIATE CORONARY SYNDROME
40228: I25.9: Chronic ischemic heart disease, unspecified
Frequency
0
5000
10000
15000
20000
25000
30000
0
5
10
15
20
cond$VisitDateTime

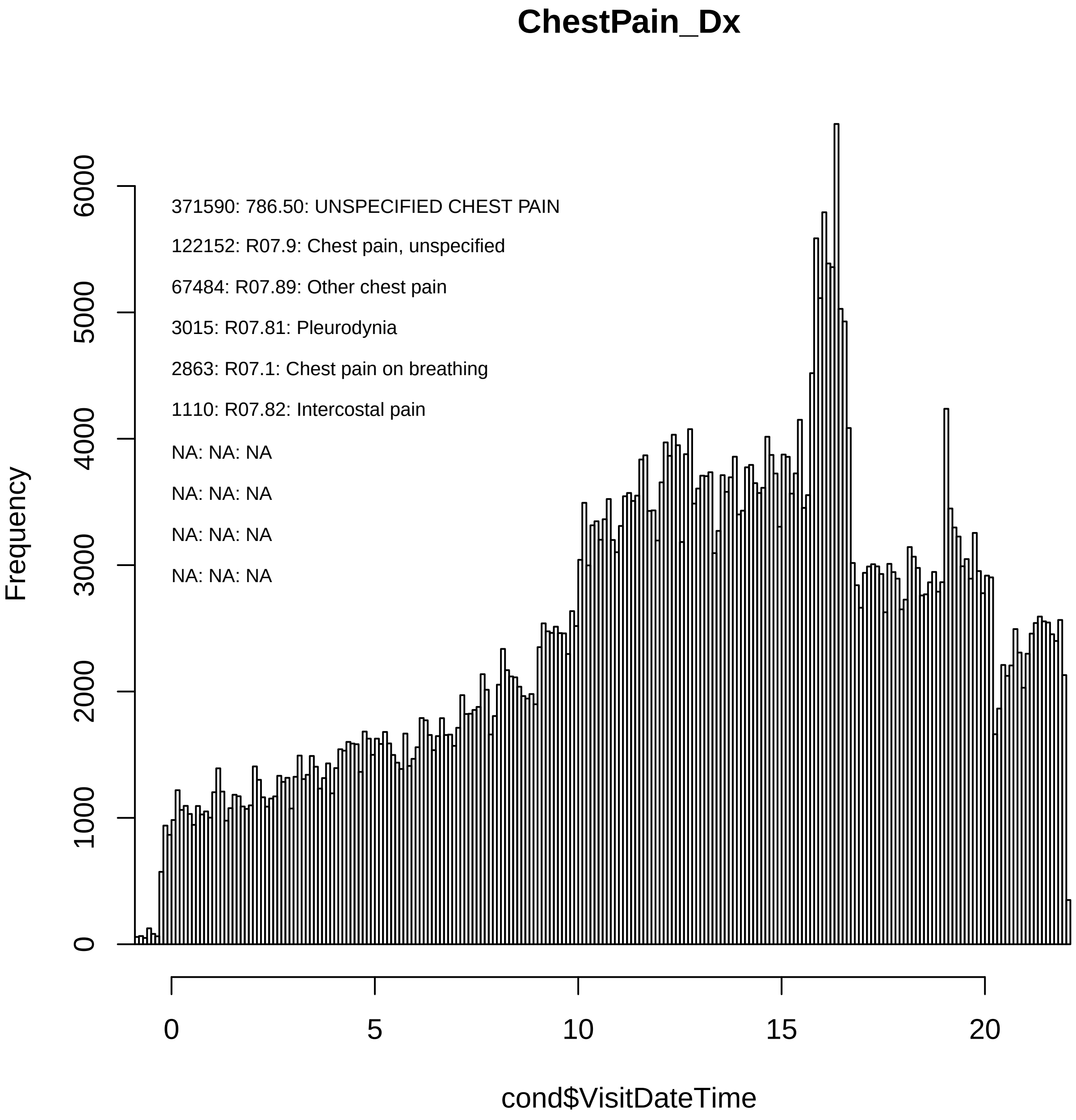
ChestPain_Dx
371590: 786.50: UNSPECIFIED CHEST PAIN
122152: R07.9: Chest pain, unspecified
67484: R07.89: Other chest pain
3015: R07.81: Pleurodynia
2863: R07.1: Chest pain on breathing
1110: R07.82: Intercostal pain
NA: NA: NA
NA: NA: NA
NA: NA: NA
NA: NA: NA
Frequency
0
1000
2000
3000
4000
5000
6000
0
5
10
15
20
cond$VisitDateTime

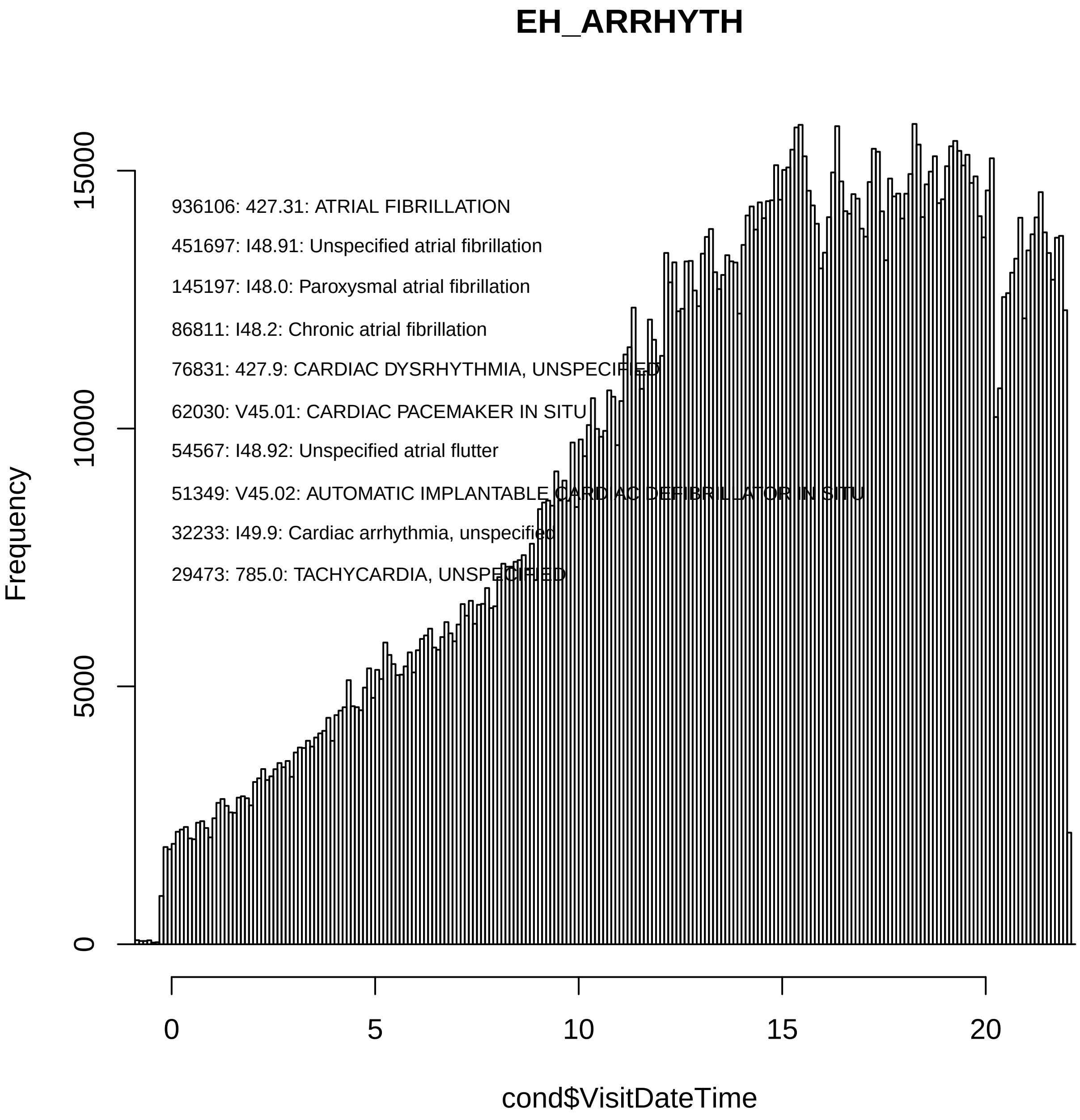
EH_ARRHYTH
936106: 427.31: ATRIAL FIBRILLATION
451697: I48.91: Unspecified atrial fibrillation
145197: I48.0: Paroxysmal atrial fibrillation
86811: I48.2: Chronic atrial fibrillation
76831: 427.9: CARDIAC DYSRHYTHMIA, UNSPECIFIED
62030: V45.01: CARDIAC PACEMAKER IN SITU
54567: I48.92: Unspecified atrial flutter
51349: V45.02: AUTOMATIC IMPLANTABLE CARDIAC DEFIBRILLATOR IN SITU
32233: I49.9: Cardiac arrhythmia, unspecified
29473: 785.0: TACHYCARDIA, UNSPECIFIED
Frequency
0
5000
10000
15000
0
5
10
15
20
cond$VisitDateTime

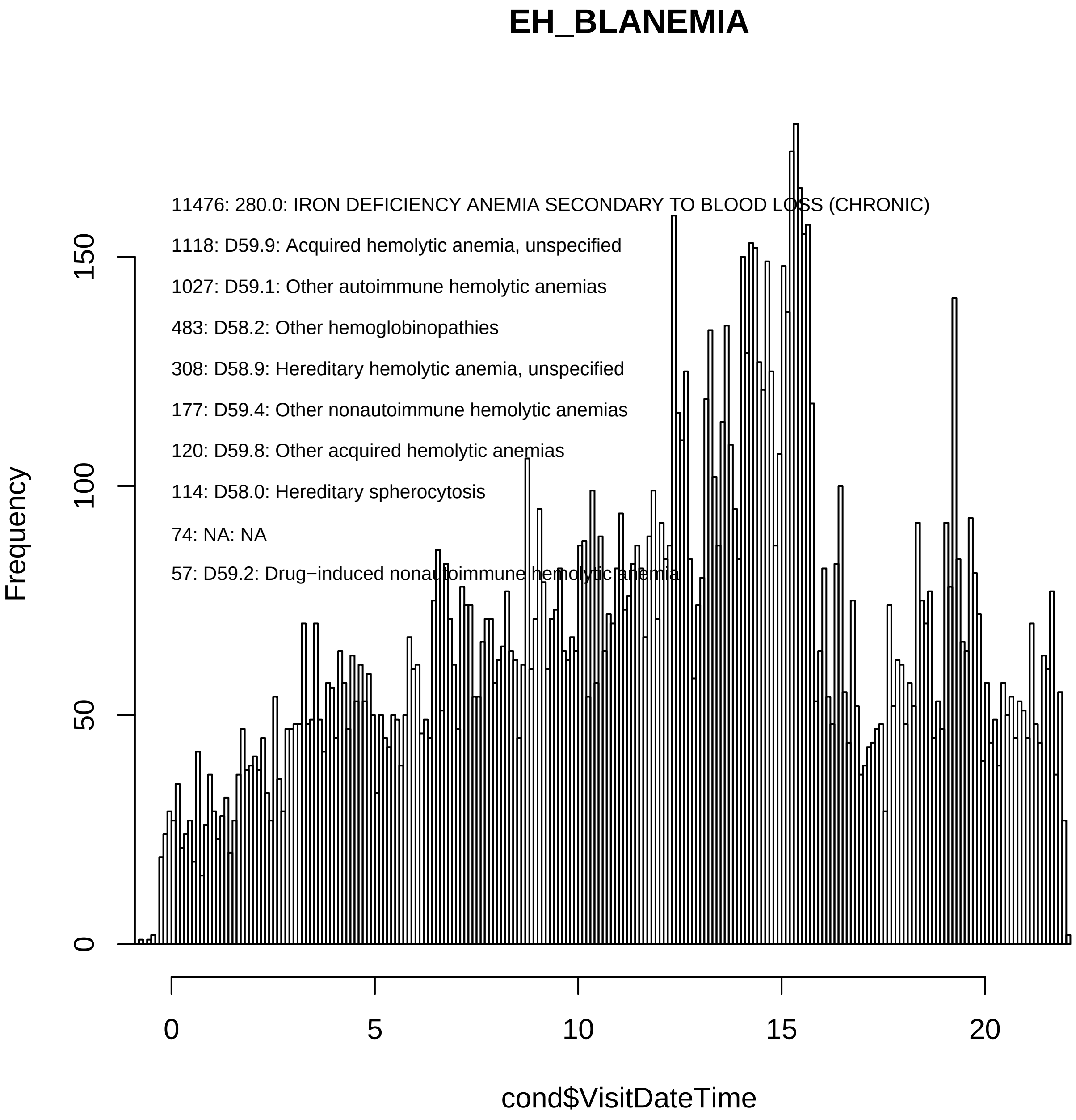
EH_BLANEMIA
11476: 280.0: IRON DEFICIENCY ANEMIA SECONDARY TO BLOOD LOSS (CHRONIC)
1118: D59.9: Acquired hemolytic anemia, unspecified
1027: D59.1: Other autoimmune hemolytic anemias
483: D58.2: Other hemoglobinopathies
308: D58.9: Hereditary hemolytic anemia, unspecified
177: D59.4: Other nonautoimmune hemolytic anemias
120: D59.8: Other acquired hemolytic anemias
114: D58.0: Hereditary spherocytosis
74: NA: NA
57: D59.2: Drug−induced nonautoimmune hemolytic anemia
Frequency
0
50
100
150
0
5
10
15
20
cond$VisitDateTime

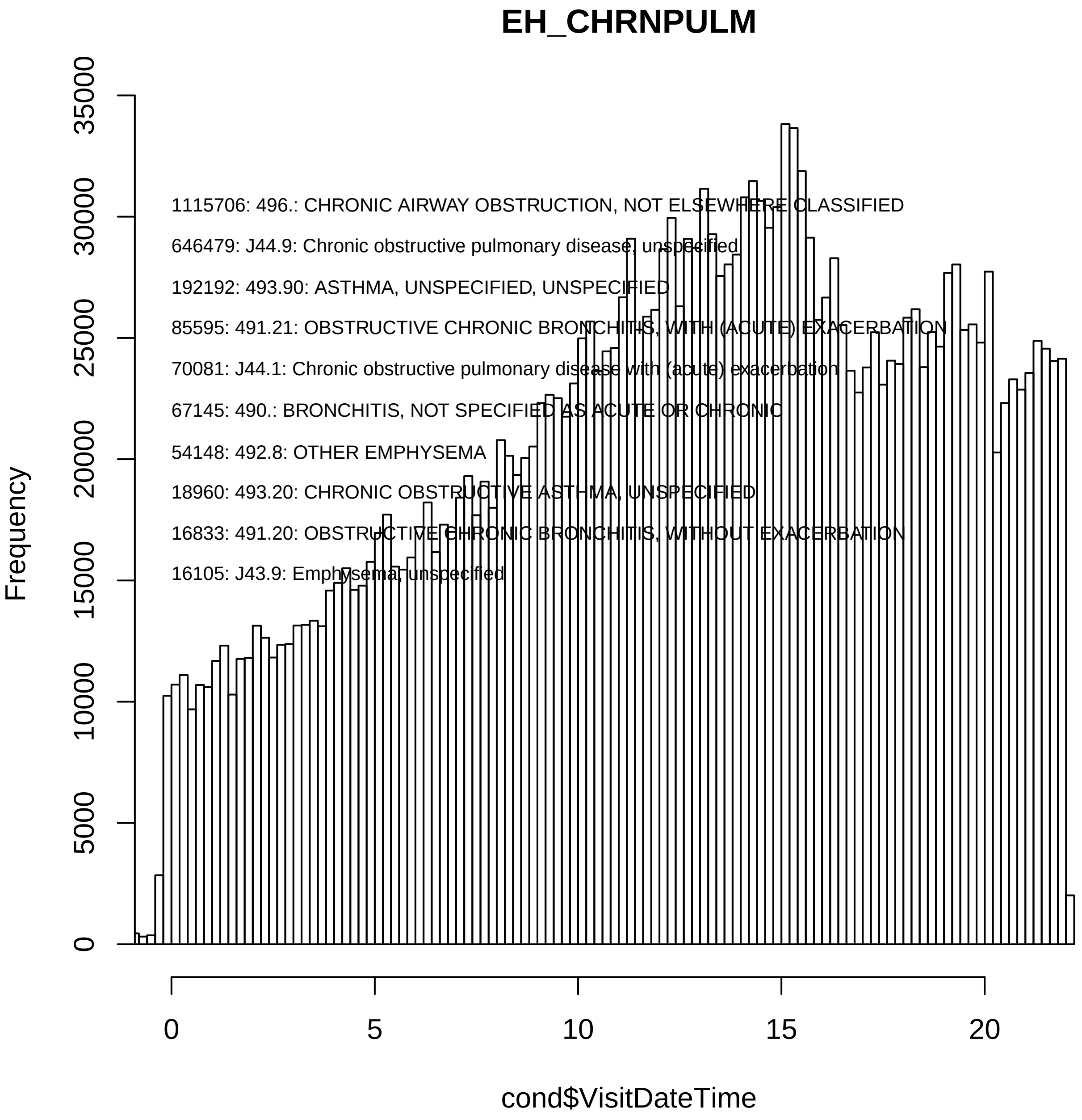
EH_CHRNPULM
Frequency
0
5000
10000
15000
20000
25000
30000
35000
1115706: 496.: CHRONIC AIRWAY OBSTRUCTION, NOT ELSEWHERE CLASSIFIED
646479: J44.9: Chronic obstructive pulmonary disease, unspecified
192192: 493.90: ASTHMA, UNSPECIFIED, UNSPECIFIED
85595: 491.21: OBSTRUCTIVE CHRONIC BRONCHITIS, WITH (ACUTE) EXACERBATION
70081: J44.1: Chronic obstructive pulmonary disease with (acute) exacerbation
67145: 490.: BRONCHITIS, NOT SPECIFIED AS ACUTE OR CHRONIC
54148: 492.8: OTHER EMPHYSEMA
18960: 493.20: CHRONIC OBSTRUCTIVE ASTHMA, UNSPECIFIED
16833: 491.20: OBSTRUCTIVE CHRONIC BRONCHITIS, WITHOUT EXACERBATION
16105: J43.9: Emphysema, unspecified
0
5
10
15
20
cond$VisitDateTime

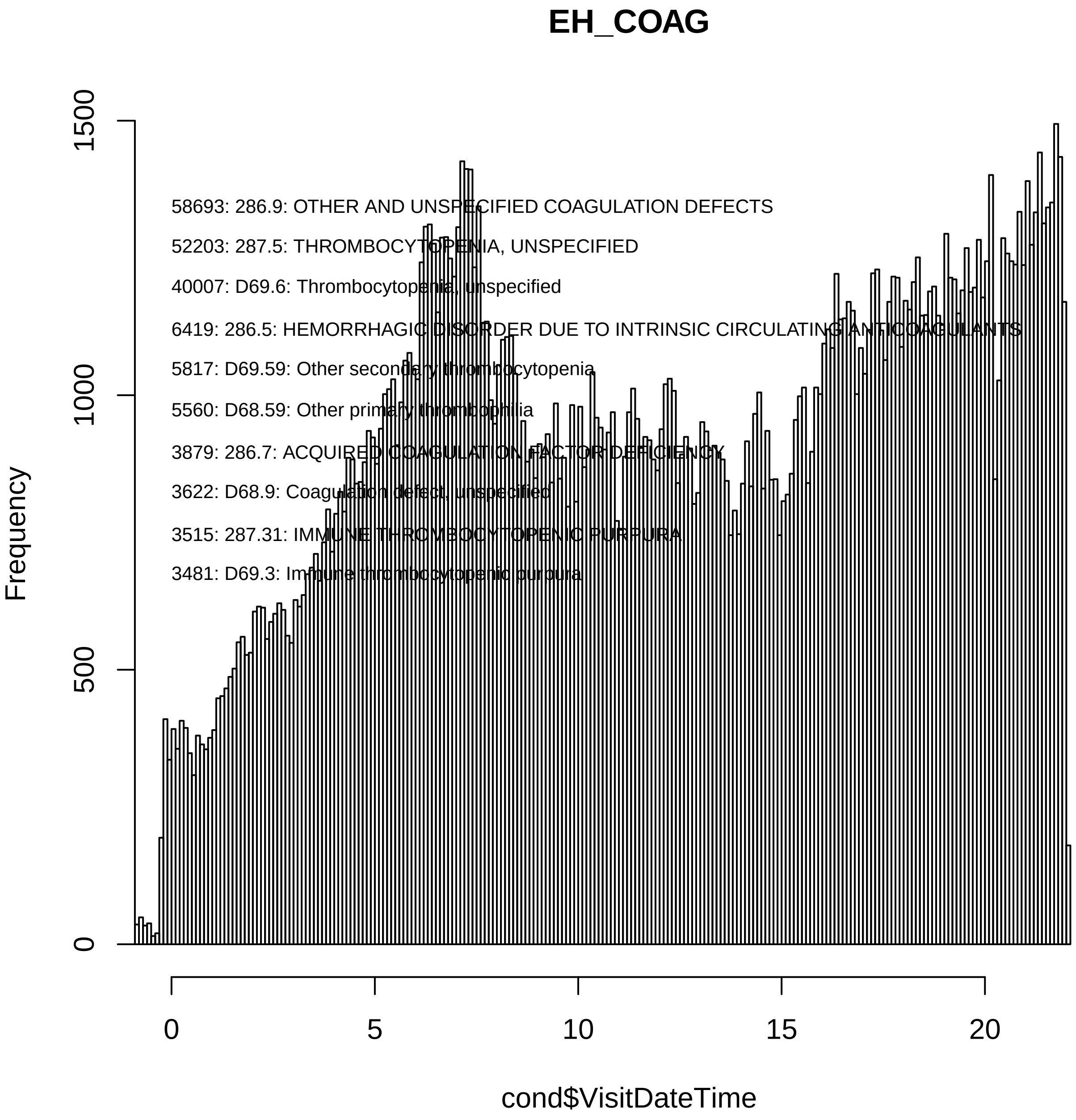

EH_COAG
58693: 286.9: OTHER AND UNSPECIFIED COAGULATION DEFECTS
52203: 287.5: THROMBOCYTOPENIA, UNSPECIFIED
40007: D69.6: Thrombocytopenia, unspecified
6419: 286.5: HEMORRHAGIC DISORDER DUE TO INTRINSIC CIRCULATING ANTICOAGULANTS
5817: D69.59: Other secondary thrombocytopenia
5560: D68.59: Other primary thrombophilia
3879: 286.7: ACQUIRED COAGULATION FACTOR DEFICIENCY
3622: D68.9: Coagulation defect, unspecified
3515: 287.31: IMMUNE THROMBOCYTOPENIC PURPURA
3481: D69.3: Immune thrombocytopenic purpura
Frequency
0
500
1000
1500
0
5
10
15
20
cond$VisitDateTime

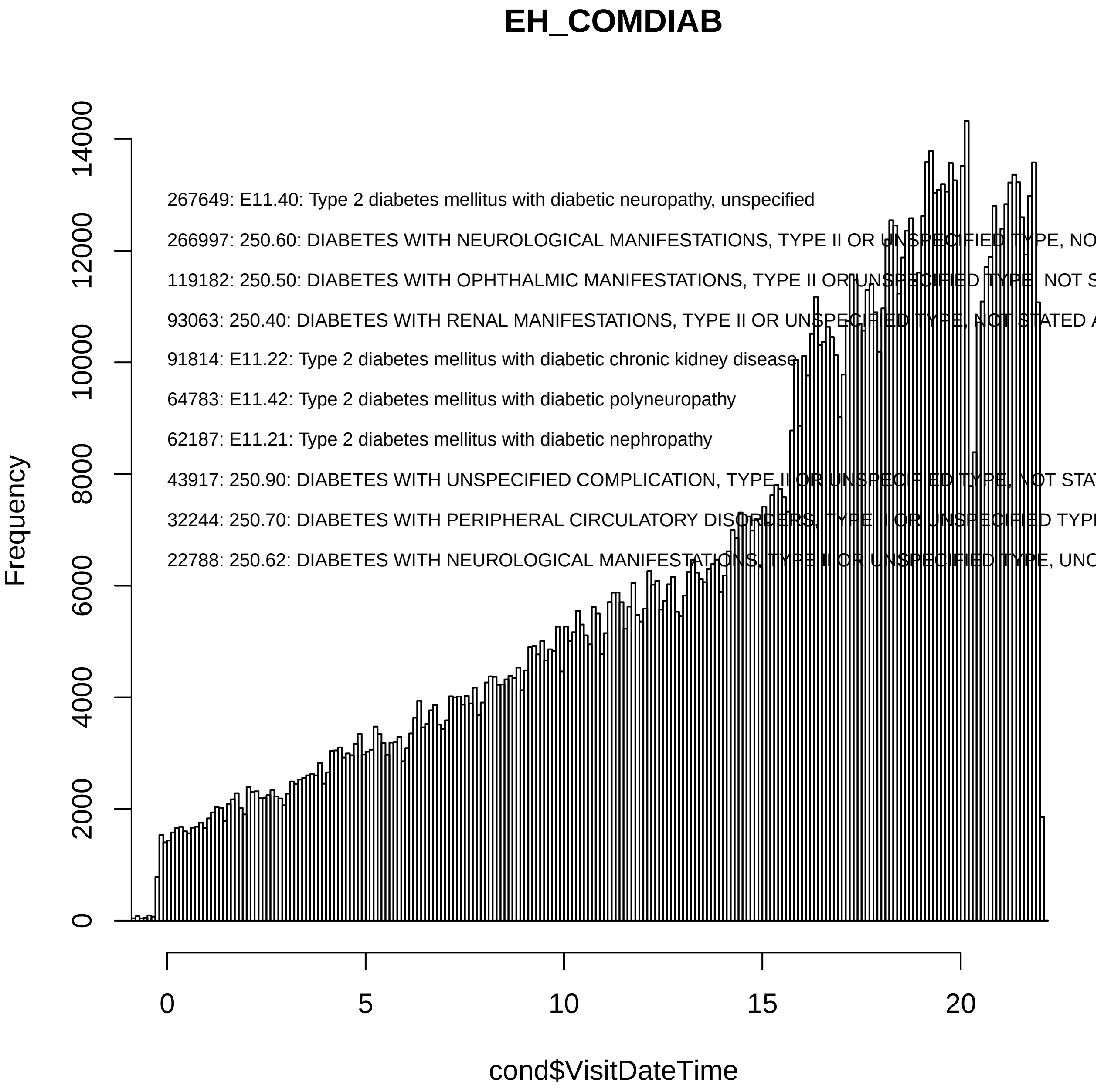
EH_COMDIAB
267649: E11.40: Type 2 diabetes mellitus with diabetic neuropathy, unspecified
266997: 250.60: DIABETES WITH NEUROLOGICAL MANIFESTATIONS, TYPE II OR UNSPECIFIED TYPE, NO
119182: 250.50: DIABETES WITH OPHTHALMIC MANIFESTATIONS, TYPE II OR UNSPECIFIED TYPE, NOT S
93063: 250.40: DIABETES WITH RENAL MANIFESTATIONS, TYPE II OR UNSPECIFIED TYPE, NOT STATED A
91814: E11.22: Type 2 diabetes mellitus with diabetic chronic kidney disease
64783: E11.42: Type 2 diabetes mellitus with diabetic polyneuropathy
62187: E11.21: Type 2 diabetes mellitus with diabetic nephropathy
43917: 250.90: DIABETES WITH UNSPECIFIED COMPLICATION, TYPE II OR UNSPECIFIED TYPE, NOT STAT
32244: 250.70: DIABETES WITH PERIPHERAL CIRCULATORY DISORDERS, TYPE II OR UNSPECIFIED TYPE
22788: 250.62: DIABETES WITH NEUROLOGICAL MANIFESTATIONS, TYPE II OR UNSPECIFIED TYPE, UNC
Frequency
0
2000
4000
6000
8000
10000
12000
14000
0
5
10
15
20
cond$VisitDateTime

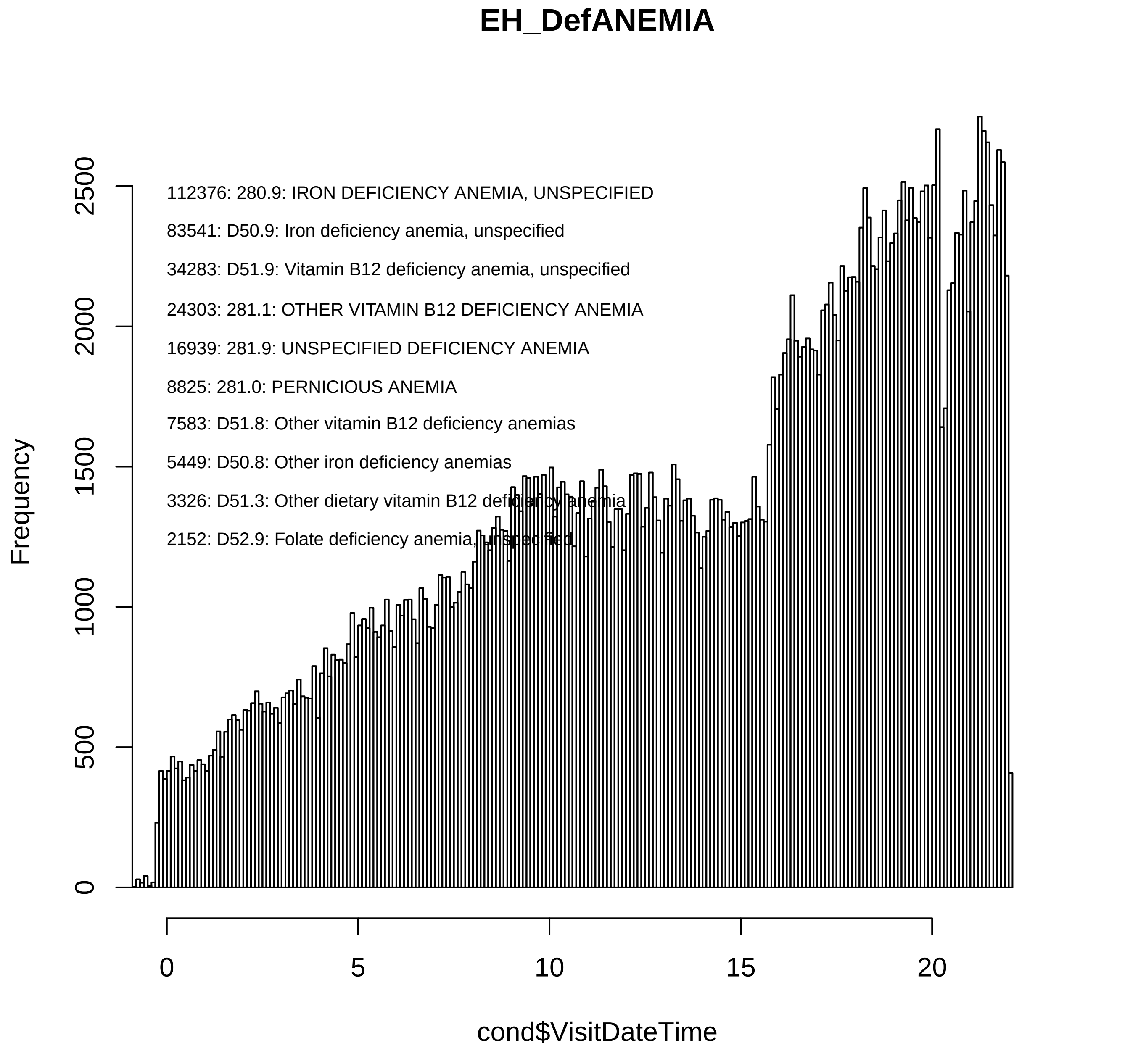
EH_DefANEMIA
112376: 280.9: IRON DEFICIENCY ANEMIA, UNSPECIFIED
83541: D50.9: Iron deficiency anemia, unspecified
34283: D51.9: Vitamin B12 deficiency anemia, unspecified
24303: 281.1: OTHER VITAMIN B12 DEFICIENCY ANEMIA
16939: 281.9: UNSPECIFIED DEFICIENCY ANEMIA
8825: 281.0: PERNICIOUS ANEMIA
7583: D51.8: Other vitamin B12 deficiency anemias
5449: D50.8: Other iron deficiency anemias
3326: D51.3: Other dietary vitamin B12 deficiency anemia
2152: D52.9: Folate deficiency anemia, unspecified
Frequency
0
500
1000
1500
2000
2500
0
5
10
15
20
cond$VisitDateTime

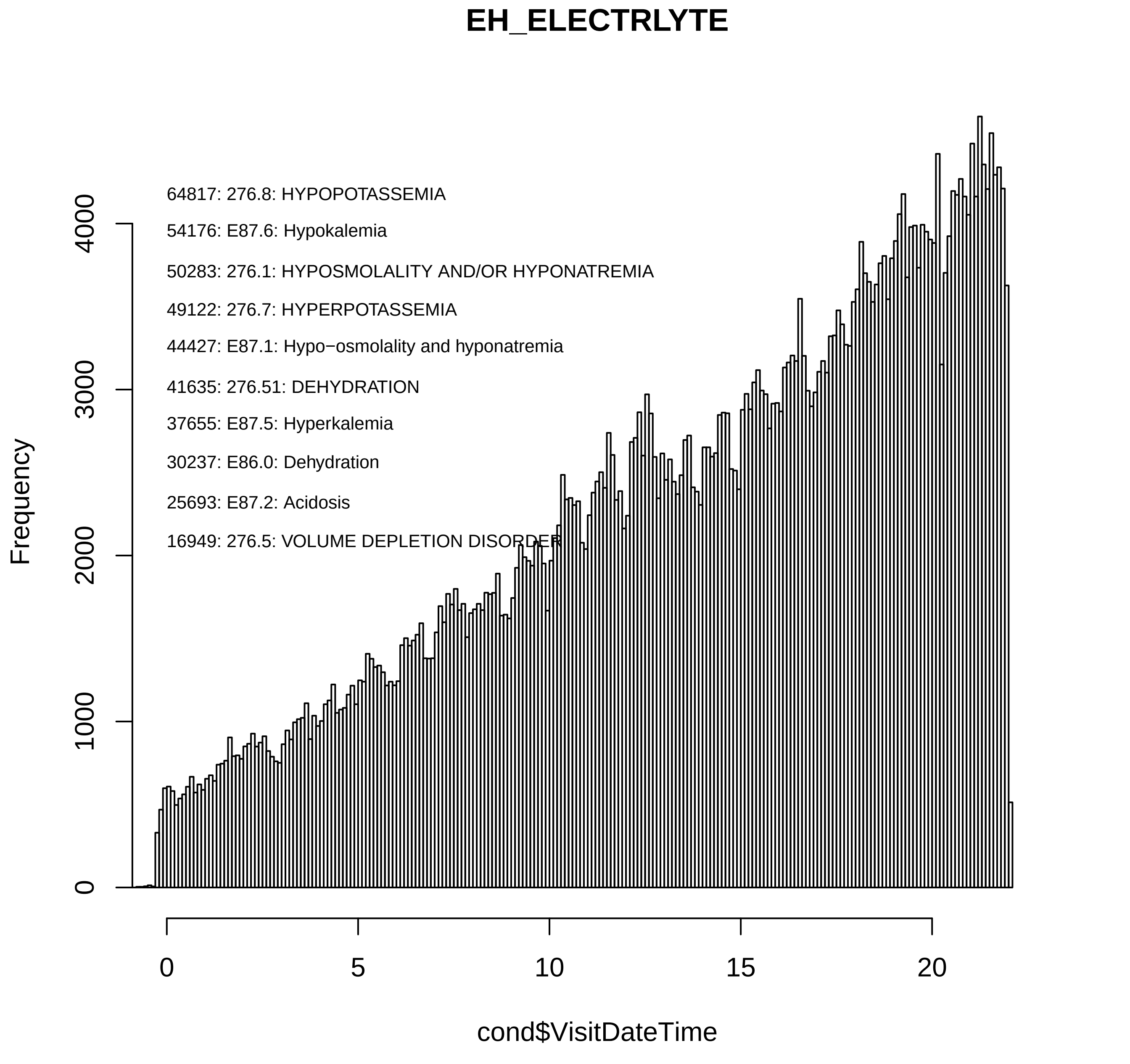
EH_ELECTRLYTE
64817: 276.8: HYPOPOTASSEMIA
54176: E87.6: Hypokalemia
50283: 276.1: HYPOSMOLALITY AND/OR HYPONATREMIA
49122: 276.7: HYPERPOTASSEMIA
44427: E87.1: Hypo−osmolality and hyponatremia
41635: 276.51: DEHYDRATION
37655: E87.5: Hyperkalemia
30237: E86.0: Dehydration
25693: E87.2: Acidosis
16949: 276.5: VOLUME DEPLETION DISORDER
Frequency
0
1000
2000
3000
4000
0
5
10
15
20
cond$VisitDateTime

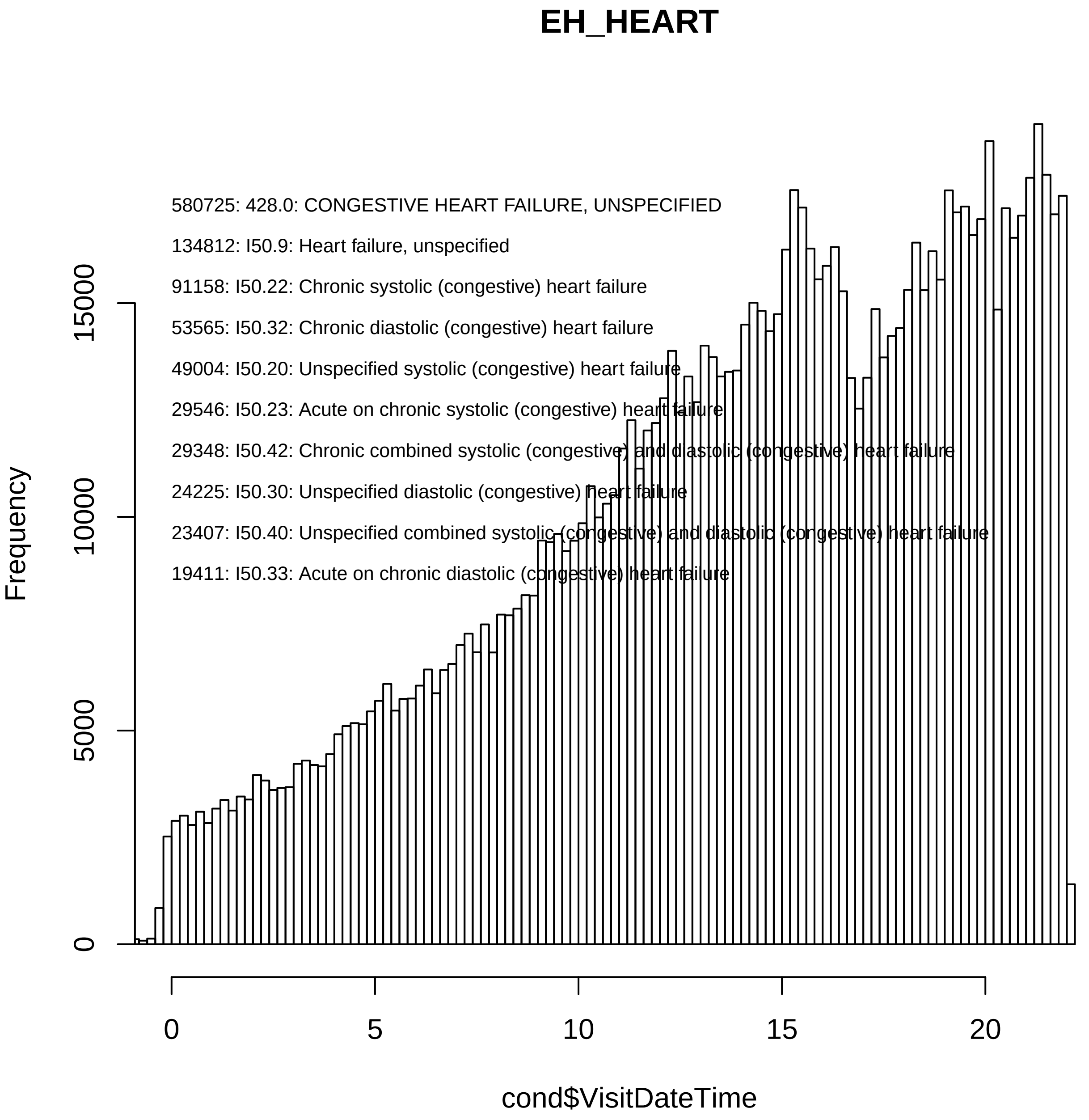
EH_HEART
580725: 428.0: CONGESTIVE HEART FAILURE, UNSPECIFIED
134812: I50.9: Heart failure, unspecified
91158: I50.22: Chronic systolic (congestive) heart failure
53565: I50.32: Chronic diastolic (congestive) heart failure
49004: I50.20: Unspecified systolic (congestive) heart failure
29546: I50.23: Acute on chronic systolic (congestive) heart failure
29348: I50.42: Chronic combined systolic (congestive) and diastolic (congestive) heart failure
24225: I50.30: Unspecified diastolic (congestive) heart failure
23407: I50.40: Unspecified combined systolic (congestive) and diastolic (congestive) heart failure
19411: I50.33: Acute on chronic diastolic (congestive) heart failure
Frequency
0
5000
10000
15000
0
5
10
15
20
cond$VisitDateTime

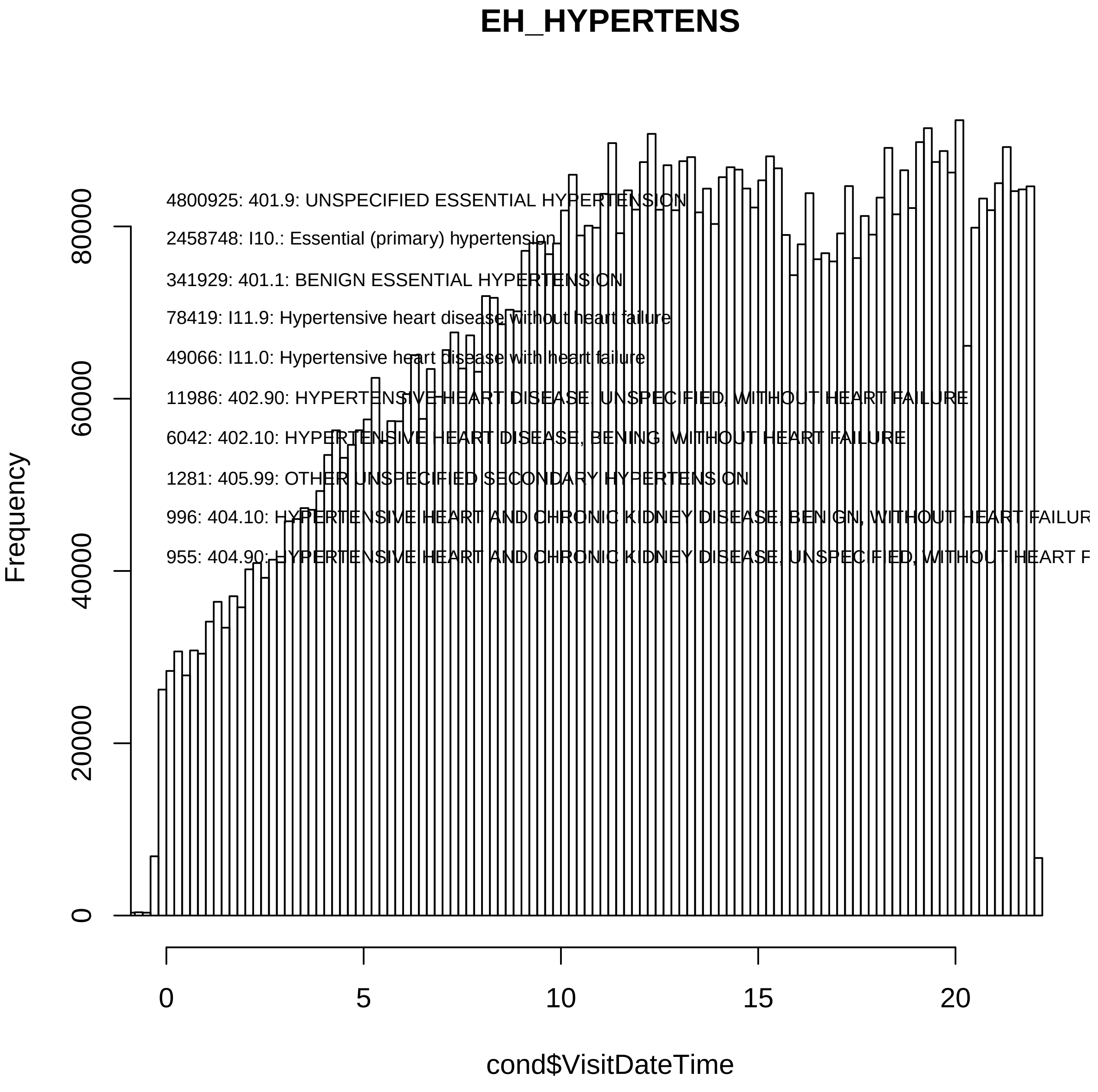
EH_HYPERTENS
4800925: 401.9: UNSPECIFIED ESSENTIAL HYPERTENSION
2458748: I10.: Essential (primary) hypertension
341929: 401.1: BENIGN ESSENTIAL HYPERTENSION
78419: I11.9: Hypertensive heart disease without heart failure
49066: I11.0: Hypertensive heart disease with heart failure
11986: 402.90: HYPERTENSIVE HEART DISEASE, UNSPECIFIED, WITHOUT HEART FAILURE
6042: 402.10: HYPERTENSIVE HEART DISEASE, BENING, WITHOUT HEART FAILURE
1281: 405.99: OTHER UNSPECIFIED SECONDARY HYPERTENSION
996: 404.10: HYPERTENSIVE HEART AND CHRONIC KIDNEY DISEASE, BENIGN, WITHOUT HEART FAILUR
955: 404.90: HYPERTENSIVE HEART AND CHRONIC KIDNEY DISEASE, UNSPECIFIED, WITHOUT HEART F
Frequency
0
20000
40000
60000
80000
0
5
10
15
20
cond$VisitDateTime

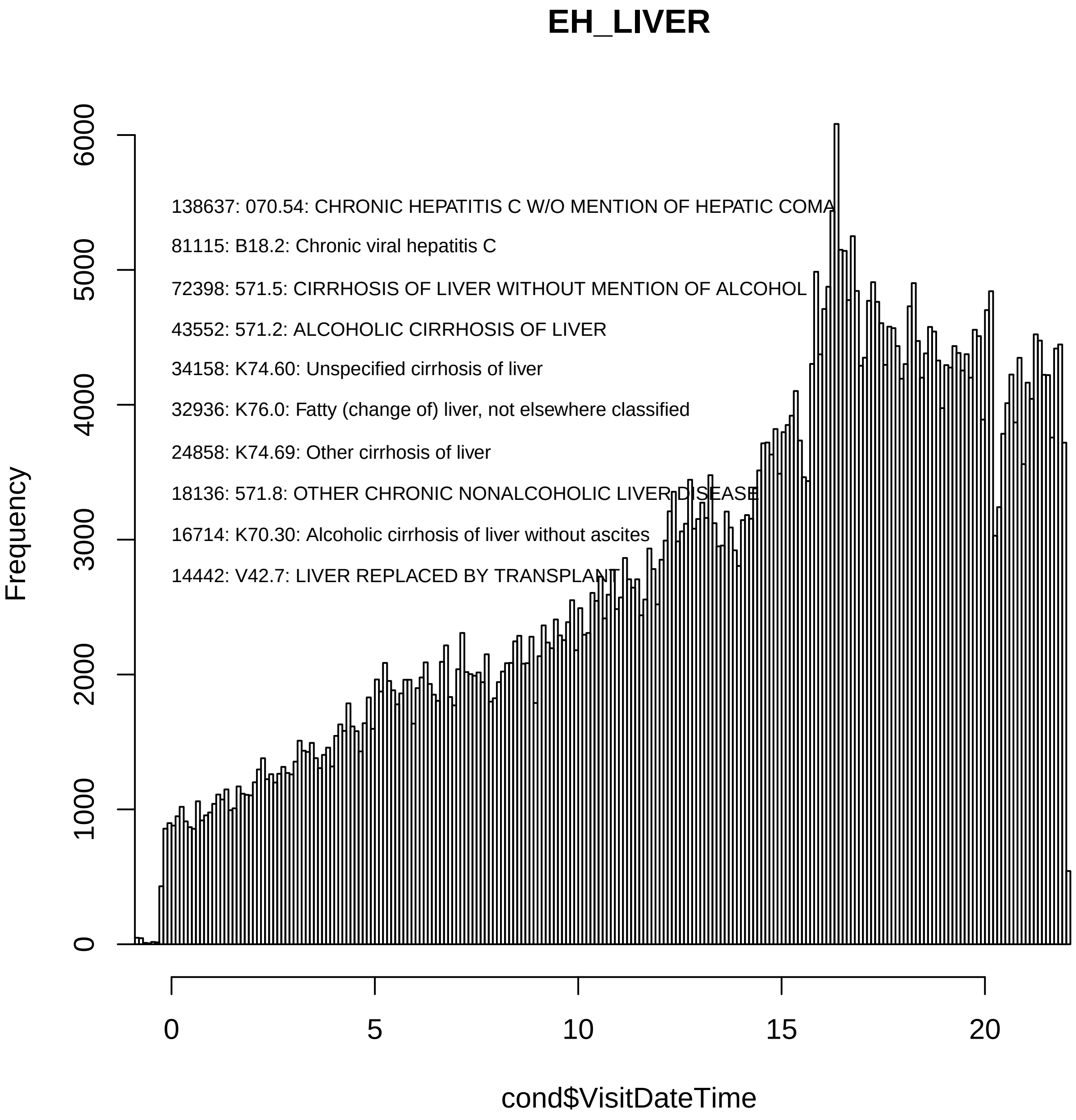

EH_LIVER
138637: 070.54: CHRONIC HEPATITIS C W/O MENTION OF HEPATIC COMA
81115: B18.2: Chronic viral hepatitis C
72398: 571.5: CIRRHOSIS OF LIVER WITHOUT MENTION OF ALCOHOL
43552: 571.2: ALCOHOLIC CIRRHOSIS OF LIVER
34158: K74.60: Unspecified cirrhosis of liver
32936: K76.0: Fatty (change of) liver, not elsewhere classified
24858: K74.69: Other cirrhosis of liver
18136: 571.8: OTHER CHRONIC NONALCOHOLIC LIVER DISEASE
16714: K70.30: Alcoholic cirrhosis of liver without ascites
14442: V42.7: LIVER REPLACED BY TRANSPLANT
Frequency
0
1000
2000
3000
4000
5000
6000
0
5
10
15
20
cond$VisitDateTime

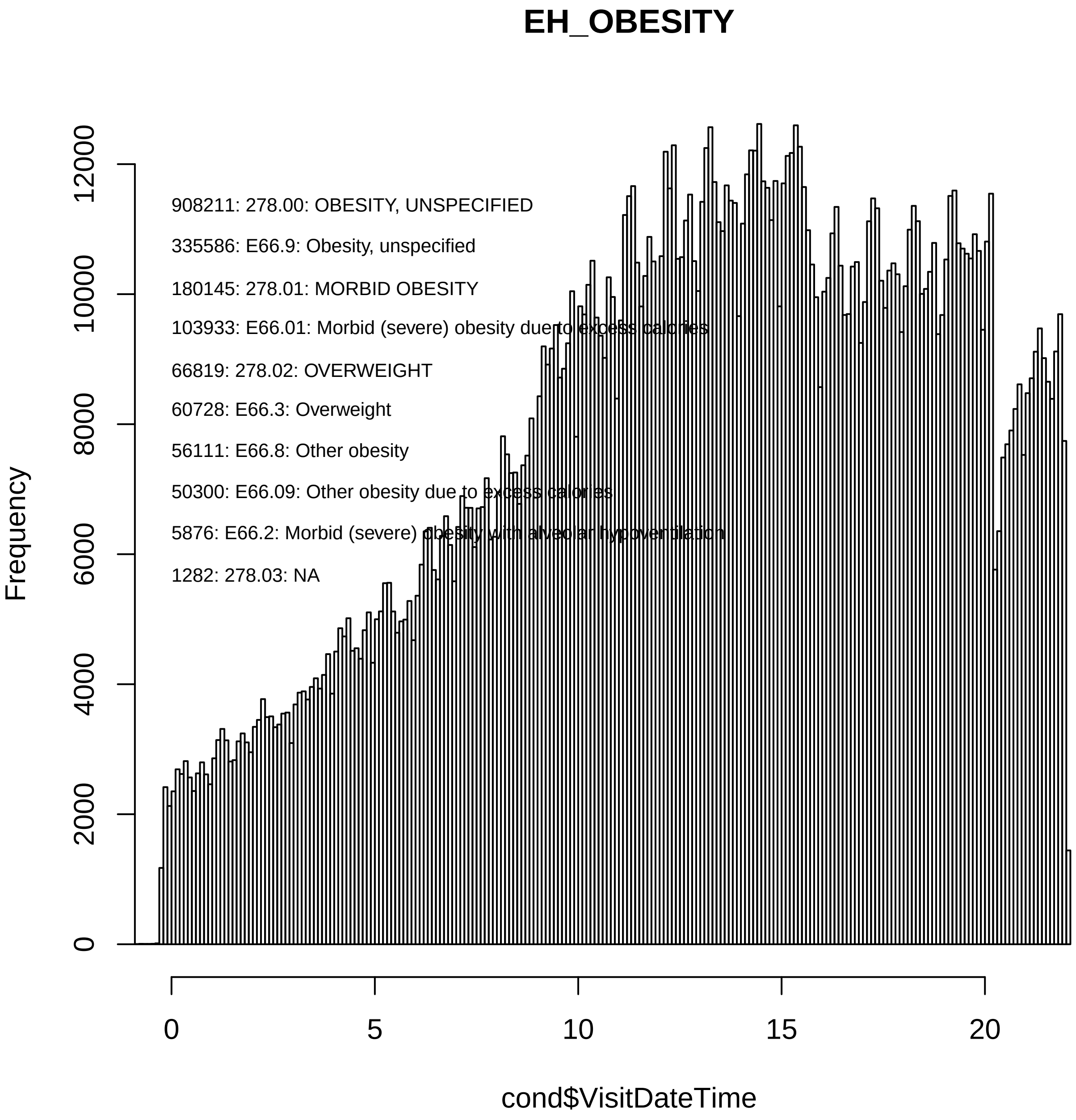
EH_OBESITY
908211: 278.00: OBESITY, UNSPECIFIED
335586: E66.9: Obesity, unspecified
180145: 278.01: MORBID OBESITY
103933: E66.01: Morbid (severe) obesity due to excess calories
66819: 278.02: OVERWEIGHT
60728: E66.3: Overweight
56111: E66.8: Other obesity
50300: E66.09: Other obesity due to excess calories
5876: E66.2: Morbid (severe) obesity with alveolar hypoventilation
1282: 278.03: NA
Frequency
0
2000
4000
6000
8000
10000
12000
0
5
10
15
20
cond$VisitDateTime

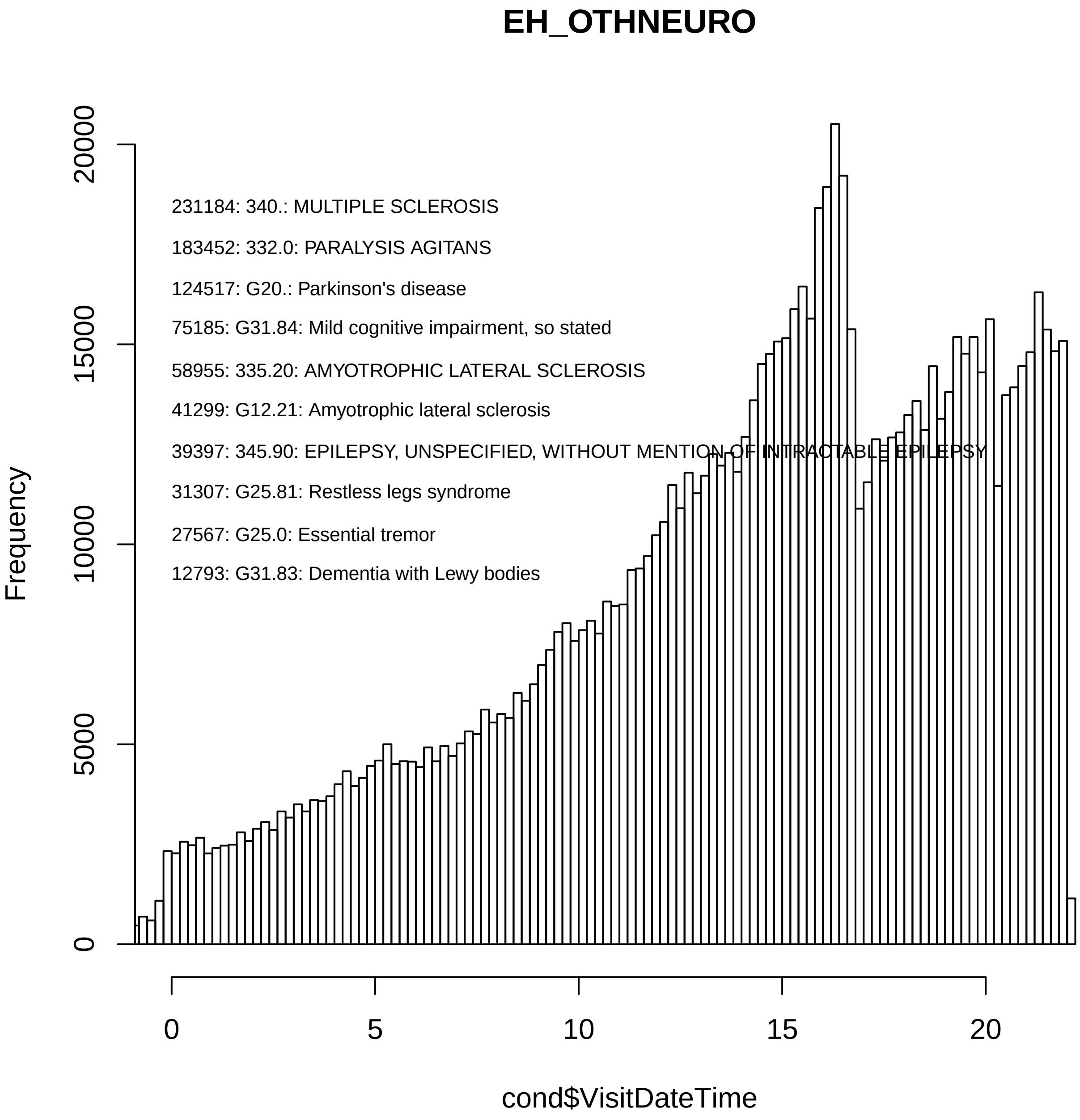

EH_OTHNEURO
231184: 340.: MULTIPLE SCLEROSIS
183452: 332.0: PARALYSIS AGITANS
124517: G20.: Parkinson's disease
75185: G31.84: Mild cognitive impairment, so stated
58955: 335.20: AMYOTROPHIC LATERAL SCLEROSIS
41299: G12.21: Amyotrophic lateral sclerosis
39397: 345.90: EPILEPSY, UNSPECIFIED, WITHOUT MENTION OF INTRACTABLE EPILEPSY
31307: G25.81: Restless legs syndrome
27567: G25.0: Essential tremor
12793: G31.83: Dementia with Lewy bodies
Frequency
0
5000
10000
15000
20000
0
5
10
15
20
cond$VisitDateTime

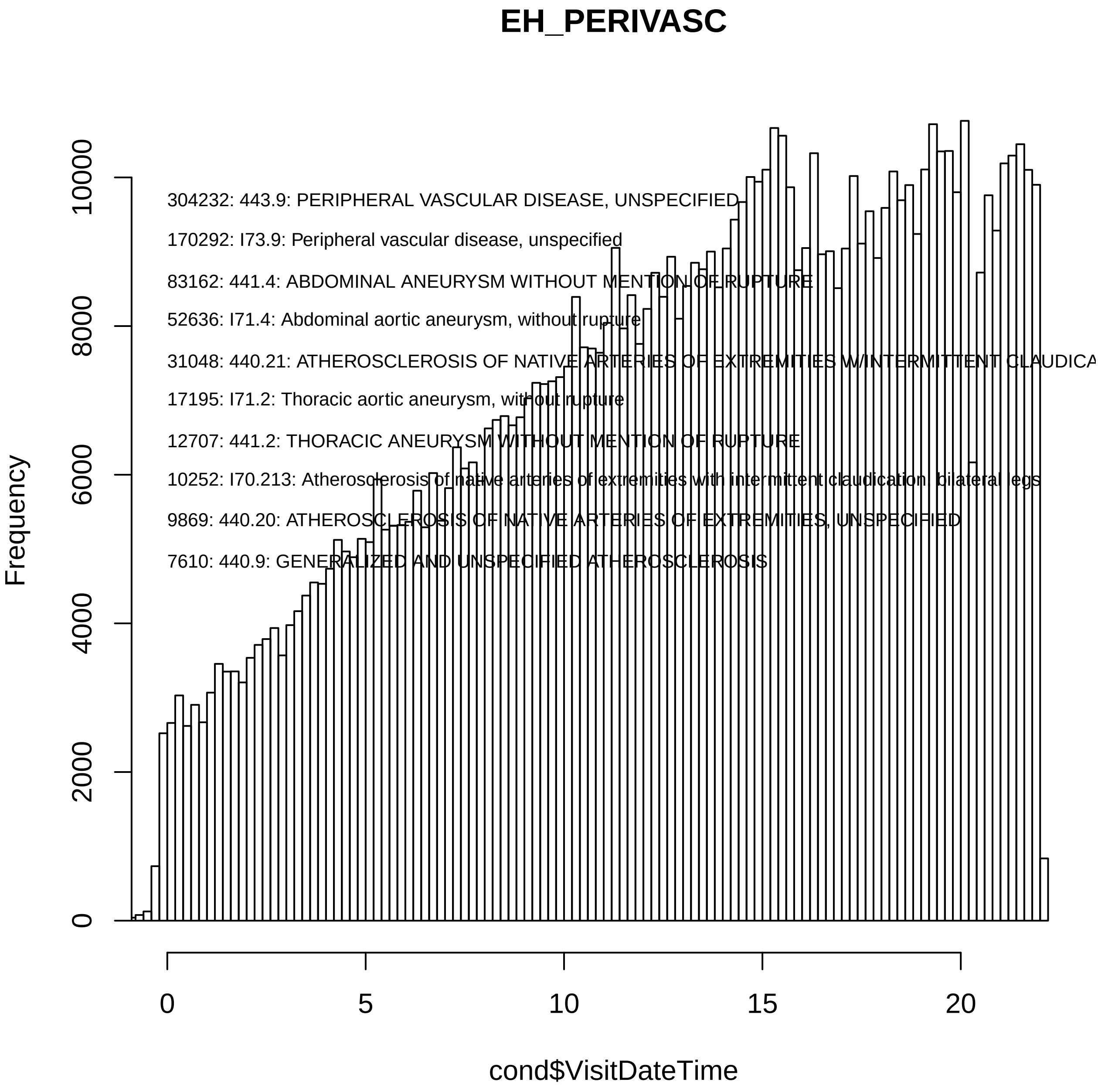
EH_PERIVASC
304232: 443.9: PERIPHERAL VASCULAR DISEASE, UNSPECIFIED
170292: I73.9: Peripheral vascular disease, unspecified
83162: 441.4: ABDOMINAL ANEURYSM WITHOUT MENTION OF RUPTURE
52636: I71.4: Abdominal aortic aneurysm, without rupture
31048: 440.21: ATHEROSCLEROSIS OF NATIVE ARTERIES OF EXTREMITIES W/INTERMITTENT CLAUDICA
17195: I71.2: Thoracic aortic aneurysm, without rupture
12707: 441.2: THORACIC ANEURYSM WITHOUT MENTION OF RUPTURE
10252: I70.213: Atherosclerosis of native arteries of extremities with intermittent claudication, bilateral legs
9869: 440.20: ATHEROSCLEROSIS OF NATIVE ARTERIES OF EXTREMITIES, UNSPECIFIED
7610: 440.9: GENERALIZED AND UNSPECIFIED ATHEROSCLEROSIS
Frequency
0
2000
4000
6000
8000
10000
0
5
10
15
20
cond$VisitDateTime

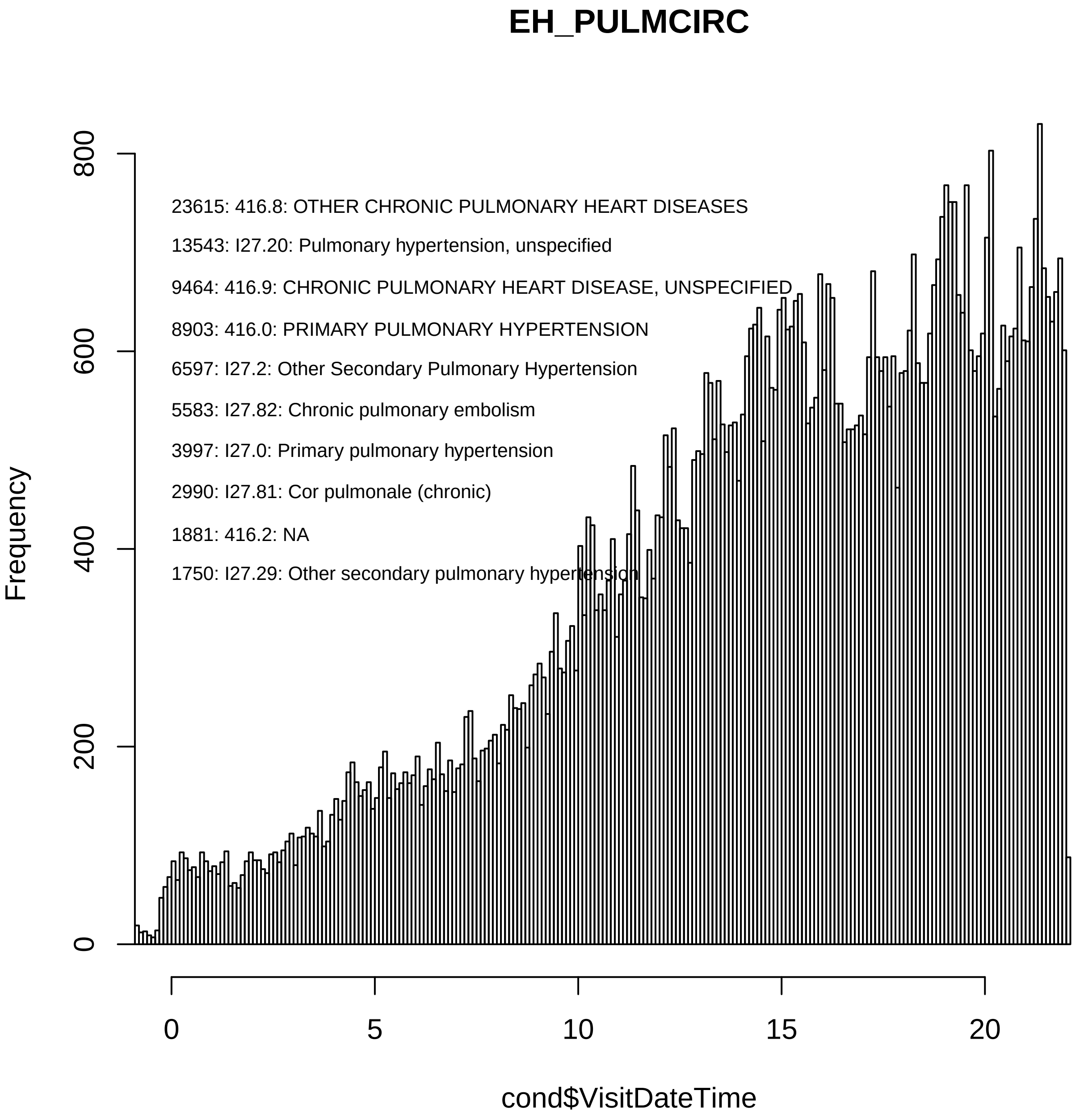
EH_PULMCIRC
23615: 416.8: OTHER CHRONIC PULMONARY HEART DISEASES
13543: I27.20: Pulmonary hypertension, unspecified
9464: 416.9: CHRONIC PULMONARY HEART DISEASE, UNSPECIFIED
8903: 416.0: PRIMARY PULMONARY HYPERTENSION
6597: I27.2: Other Secondary Pulmonary Hypertension
5583: I27.82: Chronic pulmonary embolism
3997: I27.0: Primary pulmonary hypertension
2990: I27.81: Cor pulmonale (chronic)
1881: 416.2: NA
1750: I27.29: Other secondary pulmonary hypertension
Frequency
0
200
400
600
800
0
5
10
15
20
cond$VisitDateTime

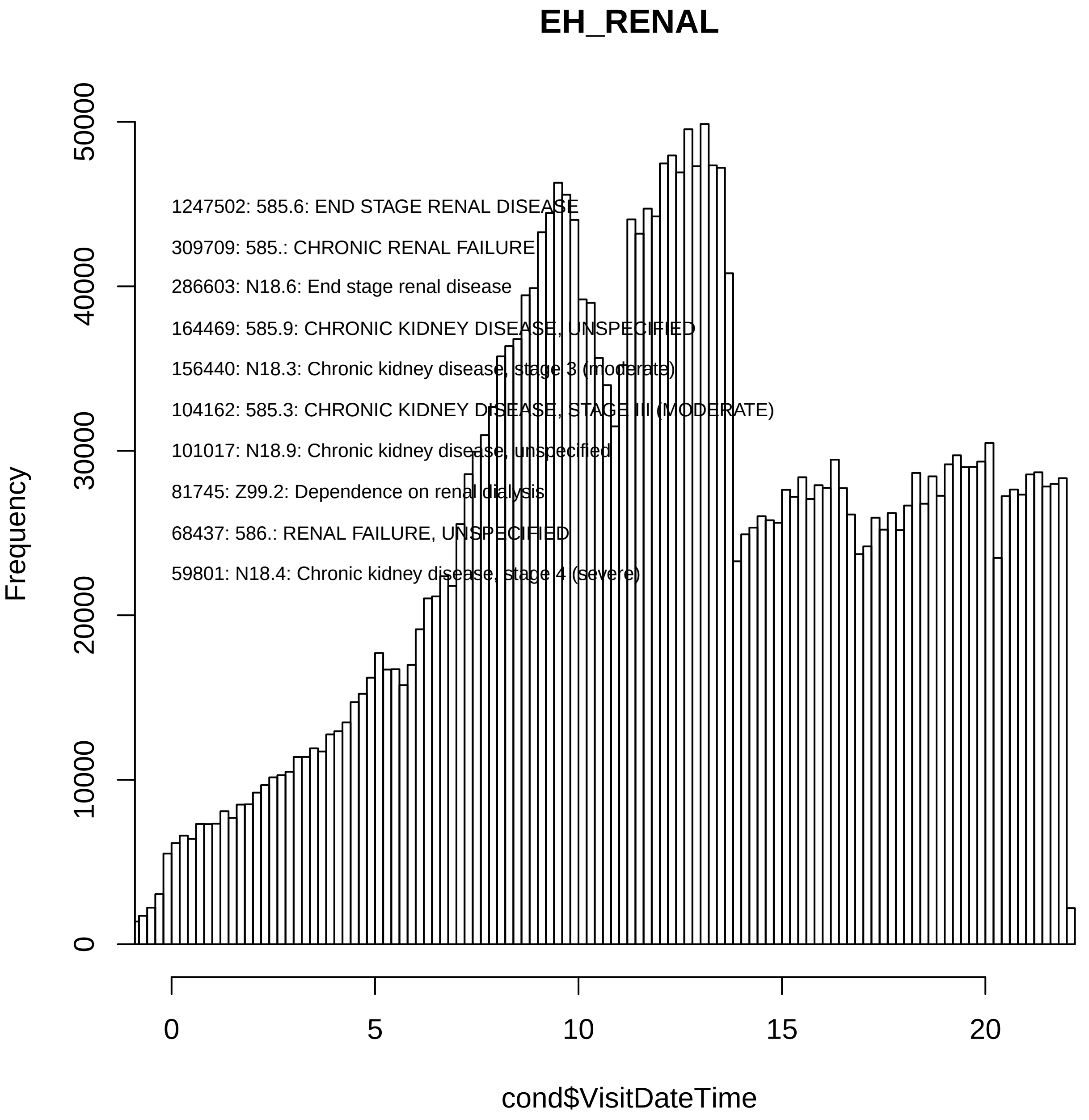
EH_RENAL
1247502: 585.6: END STAGE RENAL DISEASE
309709: 585.: CHRONIC RENAL FAILURE
286603: N18.6: End stage renal disease
164469: 585.9: CHRONIC KIDNEY DISEASE, UNSPECIFIED
156440: N18.3: Chronic kidney disease, stage 3 (moderate)
104162: 585.3: CHRONIC KIDNEY DISEASE, STAGE III (MODERATE)
101017: N18.9: Chronic kidney disease, unspecified
81745: Z99.2: Dependence on renal dialysis
68437: 586.: RENAL FAILURE, UNSPECIFIED
59801: N18.4: Chronic kidney disease, stage 4 (severe)
Frequency
0
10000
20000
30000
40000
50000
0
5
10
15
20
cond$VisitDateTime

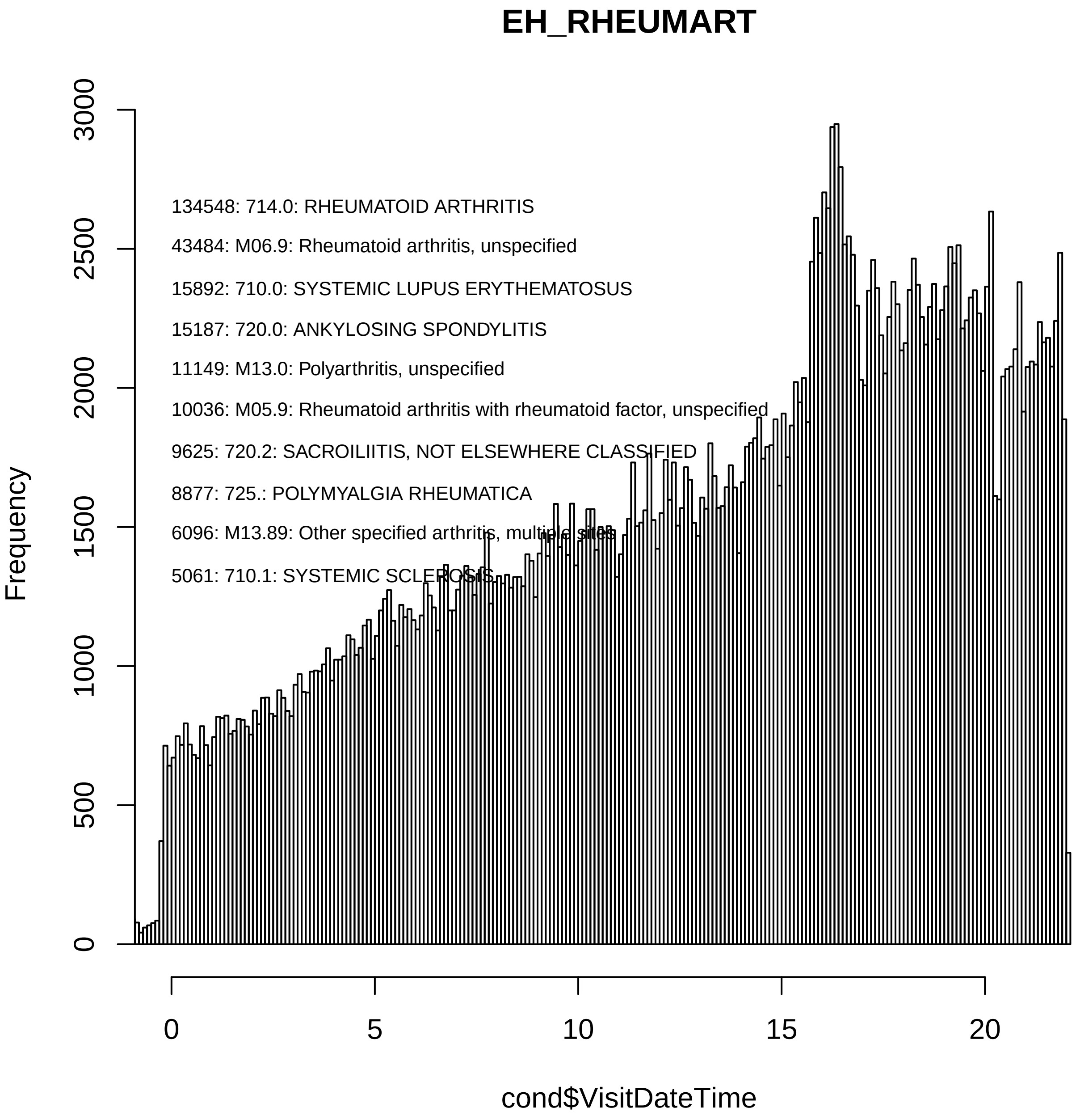
EH_RHEUMART
134548: 714.0: RHEUMATOID ARTHRITIS
43484: M06.9: Rheumatoid arthritis, unspecified
15892: 710.0: SYSTEMIC LUPUS ERYTHEMATOSUS
15187: 720.0: ANKYLOSING SPONDYLITIS
11149: M13.0: Polyarthritis, unspecified
10036: M05.9: Rheumatoid arthritis with rheumatoid factor, unspecified
9625: 720.2: SACROILIITIS, NOT ELSEWHERE CLASSIFIED
8877: 725.: POLYMYALGIA RHEUMATICA
6096: M13.89: Other specified arthritis, multiple sites
5061: 710.1: SYSTEMIC SCLEROSIS
Frequency
0
500
1000
1500
2000
2500
3000
0
5
10
15
20
cond$VisitDateTime

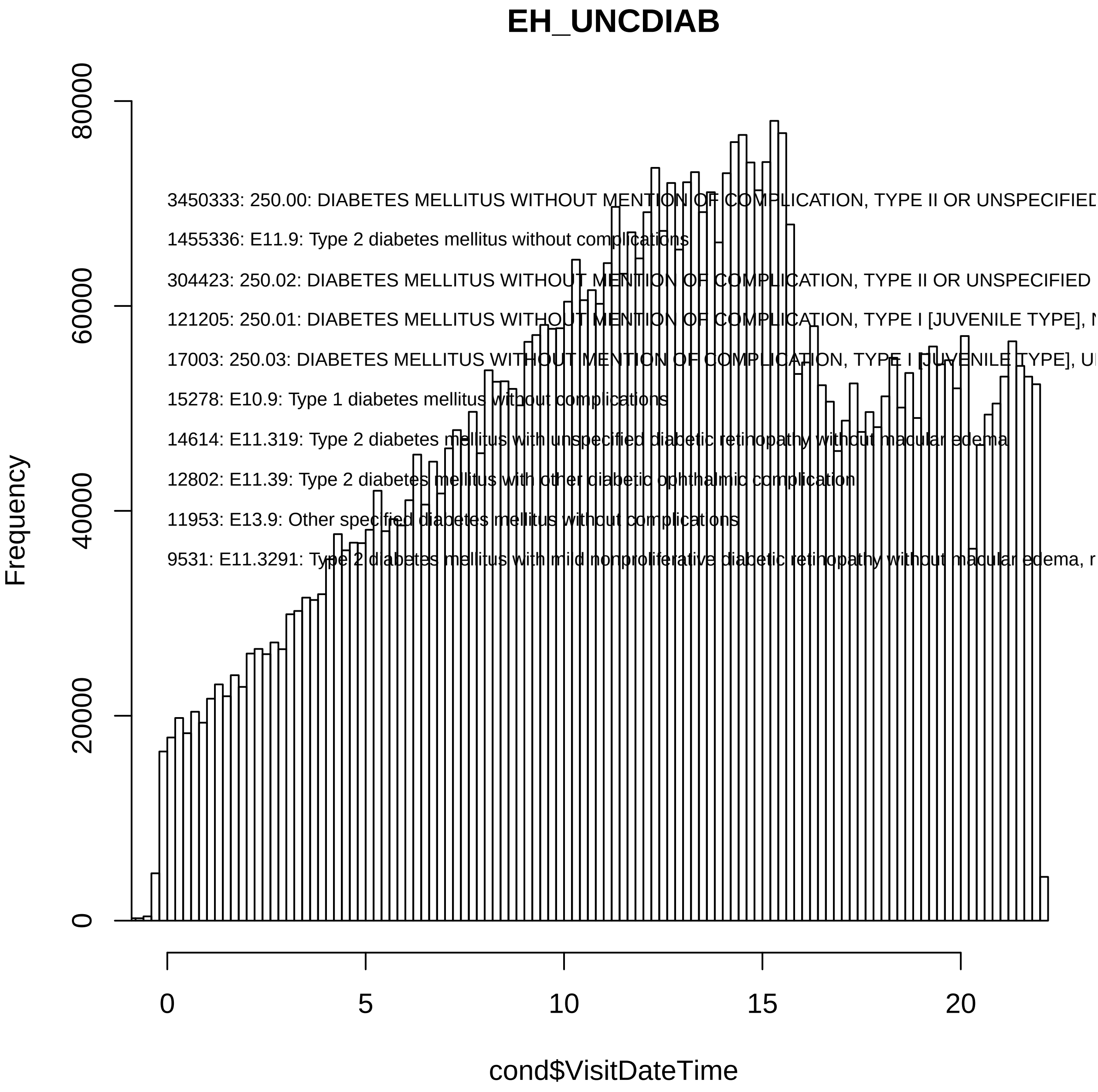
EH_UNCDIAB
3450333: 250.00: DIABETES MELLITUS WITHOUT MENTION OF COMPLICATION, TYPE II OR UNSPECIFIED
1455336: E11.9: Type 2 diabetes mellitus without complications
304423: 250.02: DIABETES MELLITUS WITHOUT MENTION OF COMPLICATION, TYPE II OR UNSPECIFIED
121205: 250.01: DIABETES MELLITUS WITHOUT MENTION OF COMPLICATION, TYPE I [JUVENILE TYPE], N
17003: 250.03: DIABETES MELLITUS WITHOUT MENTION OF COMPLICATION, TYPE I [JUVENILE TYPE], U
15278: E10.9: Type 1 diabetes mellitus without complications
14614: E11.319: Type 2 diabetes mellitus with unspecified diabetic retinopathy without macular edema
12802: E11.39: Type 2 diabetes mellitus with other diabetic ophthalmic complication
11953: E13.9: Other specified diabetes mellitus without complications
9531: E11.3291: Type 2 diabetes mellitus with mild nonproliferative diabetic retinopathy without macular edema, r
Frequency
0
20000
40000
60000
80000
0
5
10
15
20
cond$VisitDateTime

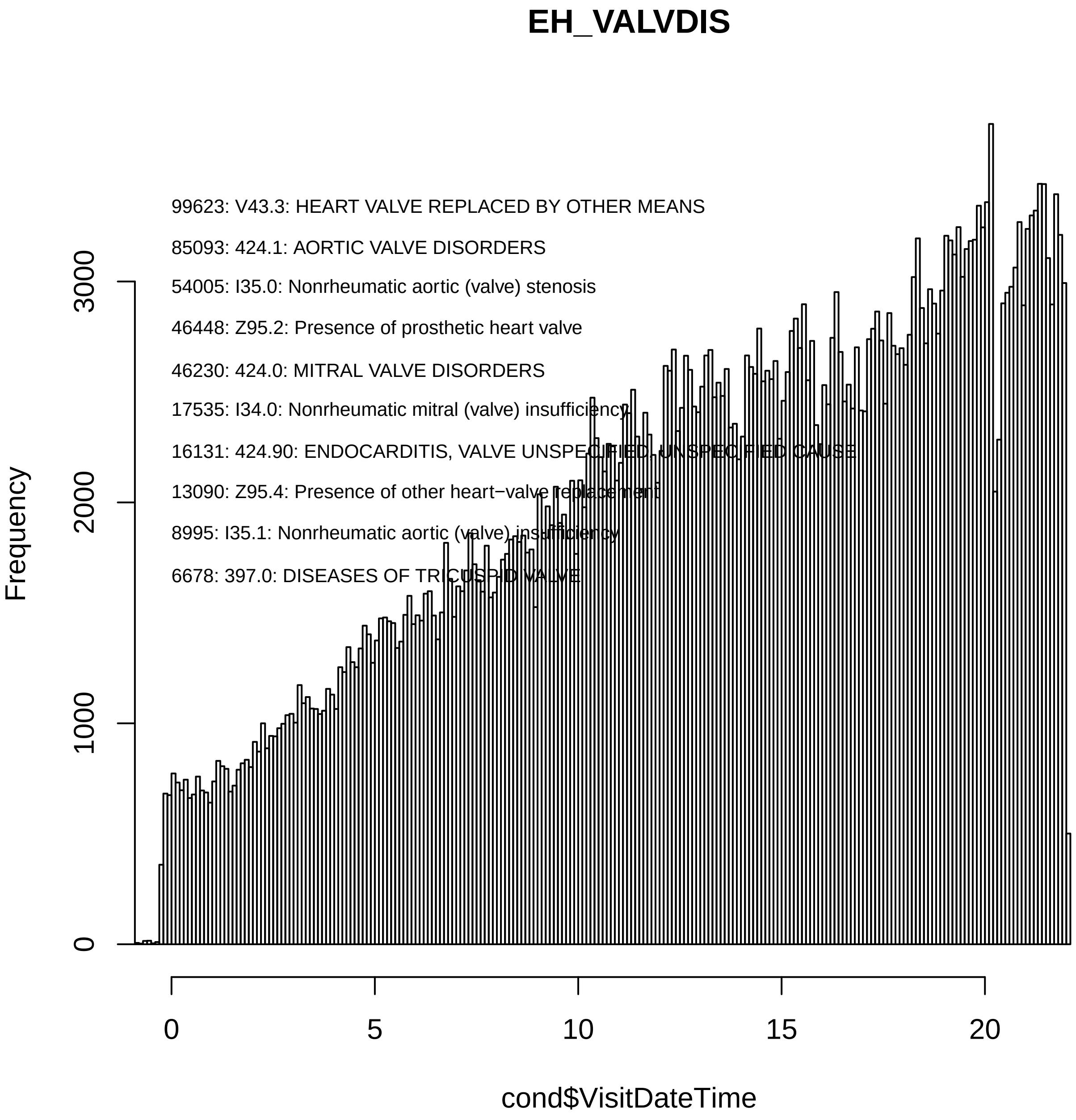
EH_VALVDIS
99623: V43.3: HEART VALVE REPLACED BY OTHER MEANS
85093: 424.1: AORTIC VALVE DISORDERS
54005: I35.0: Nonrheumatic aortic (valve) stenosis
46448: Z95.2: Presence of prosthetic heart valve
46230: 424.0: MITRAL VALVE DISORDERS
17535: I34.0: Nonrheumatic mitral (valve) insufficiency
16131: 424.90: ENDOCARDITIS, VALVE UNSPECIFIED, UNSPECIFIED CAUSE
13090: Z95.4: Presence of other heart−valve replacement
8995: I35.1: Nonrheumatic aortic (valve) insufficiency
6678: 397.0: DISEASES OF TRICUSPID VALVE
Frequency
0
1000
2000
3000
0
5
10
15
20
cond$VisitDateTime

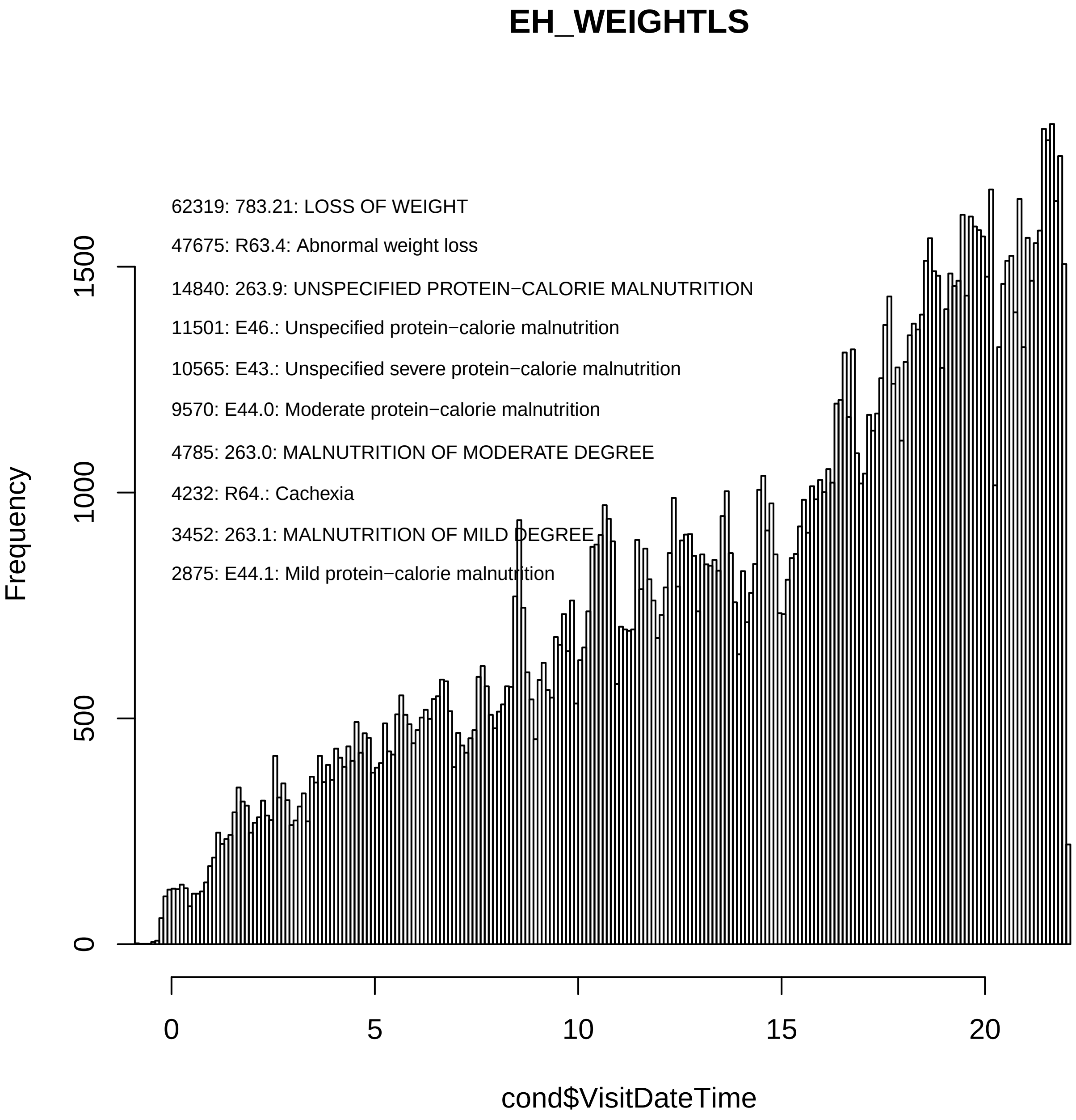
EH_WEIGHTLS
62319: 783.21: LOSS OF WEIGHT
47675: R63.4: Abnormal weight loss
14840: 263.9: UNSPECIFIED PROTEIN−CALORIE MALNUTRITION
11501: E46.: Unspecified protein−calorie malnutrition
10565: E43.: Unspecified severe protein−calorie malnutrition
9570: E44.0: Moderate protein−calorie malnutrition
4785: 263.0: MALNUTRITION OF MODERATE DEGREE
4232: R64.: Cachexia
3452: 263.1: MALNUTRITION OF MILD DEGREE
2875: E44.1: Mild protein−calorie malnutrition
Frequency
0
500
1000
1500
0
5
10
15
20
cond$VisitDateTime
