# Supplementary material for: High dimensional predictions of suicide risk in 4.2 million US Veterans using ensemble transfer learning
Source: Sci Rep. 2024 Jan 20;14:1793. doi: 10.1038/s41598-024-51762-9 (PMC10799879; doi:10.1038/s41598-024-51762-9)
Supplement: Supplementary file 4 — Supplementary Information 4. [file 41598_2024_51762_MOESM4_ESM.pdf]

Additional Information

Supplementary Information SI-1: R code, *pulldata.22.R*, together with wrapped SQL code, used to pull patient data from the VA EMR database.
Supplementary Information SI-2: Pdf, *conditions10.pdf* file with the histogram of monthly incidence of each of the 76 Dx-code-based variables together with the ten most-prevalent of the component ICD9 and ICD10 codes comprising them.
Supplementary Information SI-3: R code, *rv10.R*, used to construct study cohorts, define variables, assign data to time bins, encode data, construct input files for predictive models, and create coefficient plots, calibration curves, and histograms presented in this work. This code utilizes files created by the code provided in SI-1.
Supplementary Information SI-4: Python code, *ml_classifier.py*, used to build, train, and evaluate the machine learning based predictive models. This code also utilizes files created by the code provided in SI-1.
Supplementary Information SI-5: Excel spreadsheet, *Model.Stats*, with LASSO-selected model coefficients and statistics for each of the study designs, Rcc, C17, Cox-Visit, and Cox-MH, all trained on the combined outcome. Additionally, we provide the distribution of LOINC codes (with descriptions) across a 3% sample (date of birth cohorts 25, 50, and 75) of laboratory results of all VA patients, not tied to any specific cohort. We provide the most prevalent LOINC code used for each laboratory result, and all LOINC codes used more than 1% as often as the most prevalent, including a 'Missing' category. Note that LOINC codes were not directly utilized in defining the categories of laboratory results, as the VA utilizes its own nomenclature.

Supplementary Discussion

Several indicators of suicide, including diagnosis of suicide attempts and suicidal ideation, are among the strongest predictors of suicide and our combined outcome. PHQ2 and PHQ9 scores[1] displayed clear associations, with increasing risk from higher scores. A similar finding was noted by Simon *et al.*[2]. While use of overlapping coefficients provides more information to the predictive model, it complicates interpretation and, as evidenced by the subgroup analysis in Supplementary Figure S10, requires non-linear models to accurately account for the overlaps among categories. It is also important in this respect that all of our variables were encoded as 0/1 variables, with NA included as a separate category and normal ranges used as reference. Univariate analyses, such as used in Supplementary Table S3 and the calibration plots in the risk histograms at the bottom of Supplementary Figures S9 - S12 are also valuable to understand overlap of categories.

Mental health diagnoses showed plausible trends, with bipolar and personality disorders most strongly associated with our combined outcome. Behavioral health treatments are the strongest predictors for suicide, but there is likely a selection bias as they are potentially offered to patients at the most significant risk of negative outcomes. The difficulties in controlling for this bias is similar to the problem of comparative effectiveness analysis discussed at length in, for example, reference[3].

Among behavioral health medications, barbiturates, antidepressants, anti-anxiety medications, and opioids for pain are associated with our combined outcome, while stimulants and opioid antagonists such as naloxone were protective. A study conducted in the Netherlands found an increase in suicides resulting from barbiturate overdose[4]. In 2004, the FDA issued a black box warning on SSRI antidepressants as they increase the risk of suicide. It has been suggested that certain depressed patients might already be harboring suicidal thoughts, but would not attempt suicide until their energy is increased as a result of the antidepressant treatment[5]. Medication classes like SSRI antidepressants and benzodiazepines serve as anti-anxiety medications. Benzodiazepines may increase suicide risk in patients by increasing impulsivity or aggression, causing rebound or withdrawal symptoms, and toxicity in overdose[6]. A cohort study of patients with ADHD found a statistically significant protective effect of stimulants such as methylphenidate on suicidal behavior[7], consistant with the protective effect indicated for stimulants in Figure 4. It is proposed that their protective effect may be mediated by the improvement of ADHD symptoms, especially impulsivity[7].

Opioid users have been reported to be fourteen times more likely to die by suicide than the general population[8]. Chronic opioid use alters the limbic and striatal brain circuitry, escalating negative affect and contributing to the mental pain that accompanies suicidal behavior[9]. Our results show opioid overdose and opioid use disorder (OUD) are positively associated with our combined outcomes, while Figure 6b shows the association of opioid related variables increases suicide risk more than suicide attempts. A study conducted in the United States reported that drug overdoses from opioids accounted for roughly 40% of fatal suicide poisonings[10]. Patients with OUD have increased amygdala activation and this activation enhances negative emotional reactivity in healthy adults[11] and suicidal ideation in depressed patients. Opioid overdoses among metastatic cancer patients were examined in[12], who found no elevation in OUD but did see that overdoses more frequently occurred in advanced cancers, among patients with pre-existing mental health diagnoses, and those without previous difficulties with opioid use. Naloxone is an opioid antagonist medication that is used to reverse an opioid overdose. Figure 6a shows a striking shift in the ratio of suicide attempts and overdose, depending on whether the patient's age is above or below sixty years of age.

A 2018 study of 7,418,391 individuals living in Denmark reported that patients with severe traumatic brain injury (TBI) had a 2.38 adjusted increased rate ratio (IRR) of suicide compared to the general population, with a 1.81 IRR for mild TBI[13].

Figure 4 shows the largest risk of suicide with Dx codes for TBI, with concussion showing only a small model coefficient. When comparing to literature, it is important to realize that our model coefficients are adjusted for indicators of suicide, while literature values are typically not. The authors of the Danish study proposed the association between TBI and suicide was at least partially mediated by post-TBI psychiatric symptoms[13].

Elevated pulse rate and pulse pressure are associated with our combined outcome, consistent with the prospective study by Lemogne[14], as are decreased blood oxygenation and anemia. Our work confirms prior literature suggesting has that markers of chronic hypoxia such as smoking, living at high altitude and diagnosis of chronic obstructive pulmonary disease (COPD) or asthma may lead to reduced brain serotonin synthesis, depressing mood and contributing to suicide[15]. A study of the association of elevation to suicide risk identified a hypoxia link, although other variables such as gun ownership and reduced access to medical care must be disentangled from this association[16].

Coronary artery disease (CAD), electrolyte imbalances, and cardiac arrhythmias are predictive of our combined outcome, but heart attacks and diabetes were not. Evidence suggests multiple mechanisms by which anger or hostility may be linked to CAD[17]. It has been hypothesized that this association can also be explained by shared common genetic factors associated with vascular vulnerability which appears to be modulated by high risk environments[18]. Cardiac arrhythmia is most commonly caused by electrolyte imbalance. Low intracellular concentration of electrolytes such as potassium has been observed among depressed patients and also found in the cerebral tissue of patients who died by suicide[19].

We included a dozen variables from the American Community Survey[20] in our analysis, including variables characterizing poverty, disability, insurance, and other demographics. Only two of these variables showed effects above the 20% level: zip codes with relatively high fractions with Native American race and zip codes with a relatively high fraction of Spanish-speaking population. The decreased risk associated with Native Americans is perhaps surprising, given the relatively high suicide rates associated with such communities[21], suggesting that a less-sensitive reporting of events may be the relevant attribute.

Marital status change, including divorce, death of spouse, and new marriages are predictive of the combined outcomes. Being married in contrast, is protective in comparison with not being married. A Norwegian study concluded that being divorced or separated is associated with higher suicide risk than never being married, which they attributed to weakened social ties[22].

Our results, represented in Figure 4 shows obesity is protective against the combined outcomes, consistent with reduced levels of suicide ideation reported in studies in 2009[23], of 33,249 individuals drawn from the South Austrailian population and 2022[24], of 13,943 young US men and women chosen to be representative of the US population.

Supplementary Figures and Tables

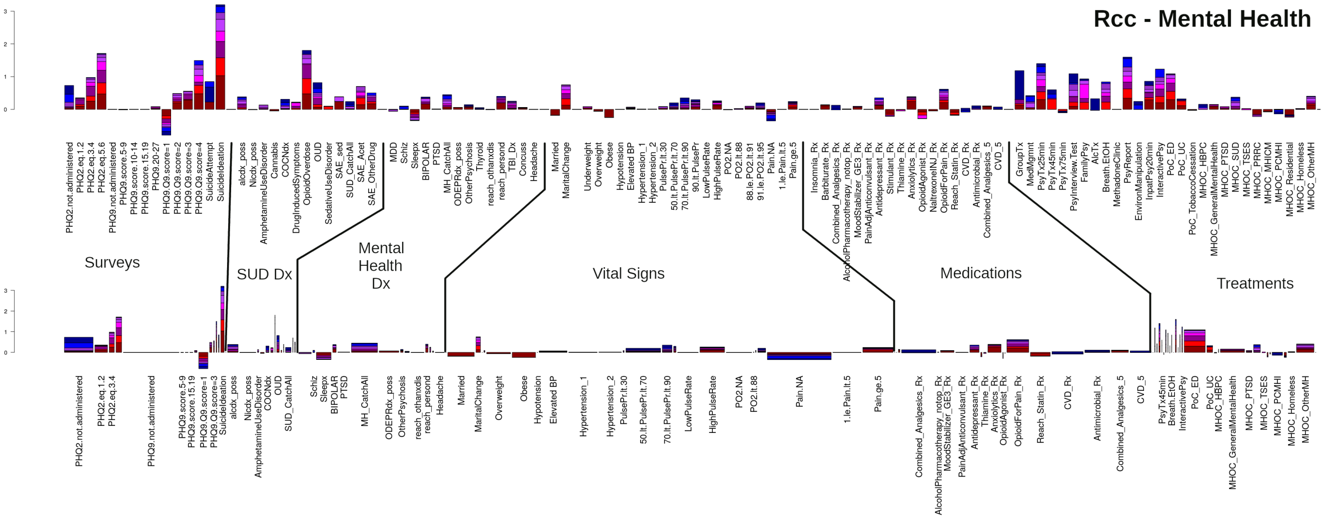

Figure S1. Model coefficients for Rcc cohort related to mental health predictors.

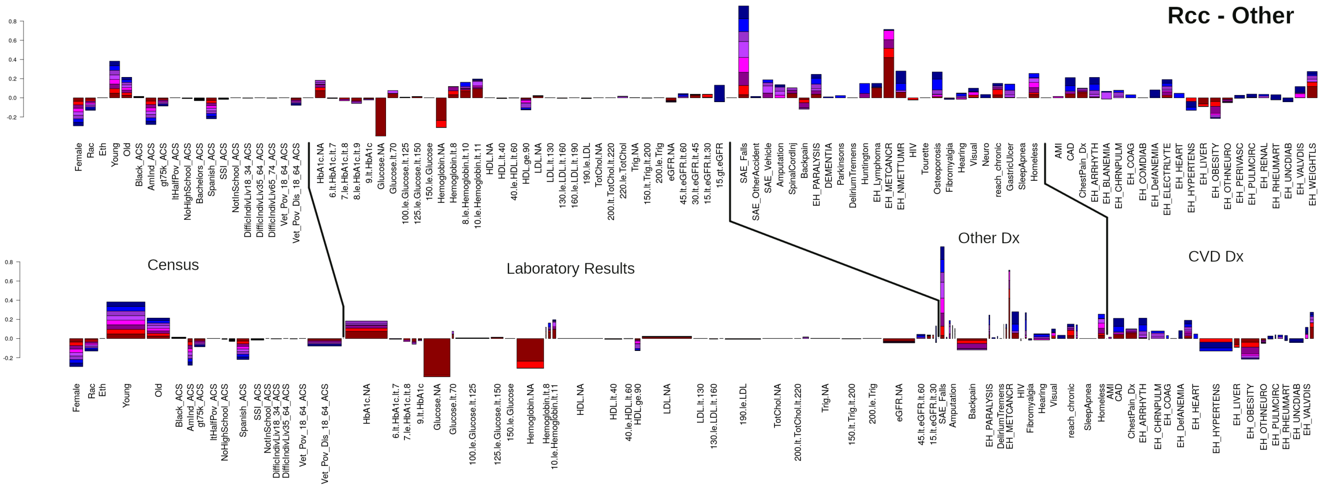

Figure S2. Model coefficients for Rcc cohort related to non mental health related predictors

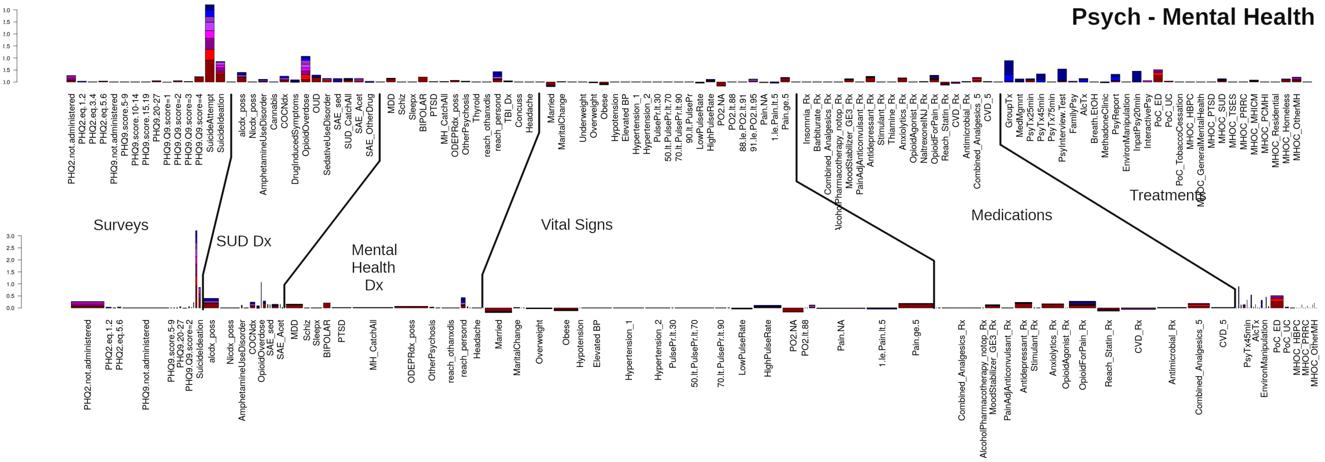

Figure S3. Model coefficients for Cox-MH cohort related to mental health predictors.

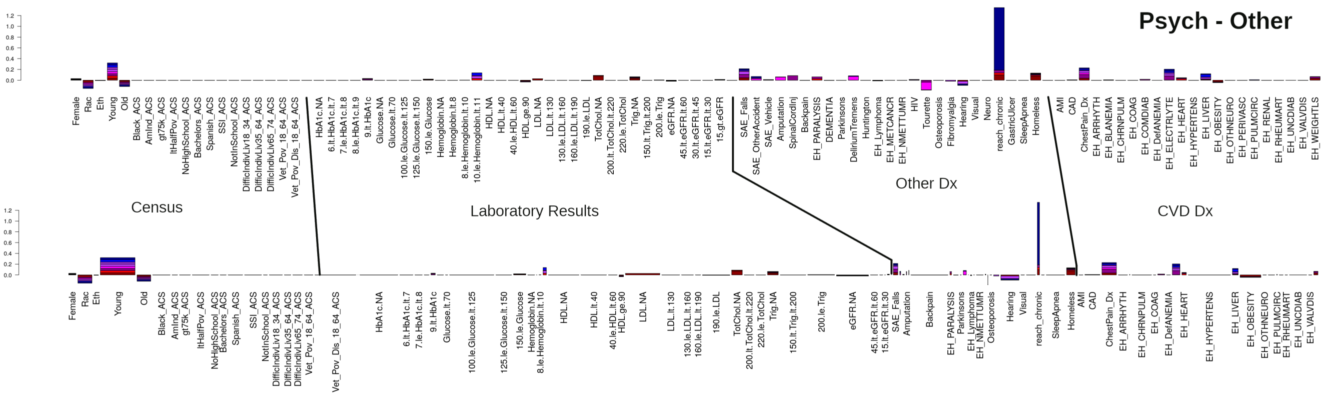

Figure S4. Model coefficients for Cox-MH cohort related to non mental health related predictors

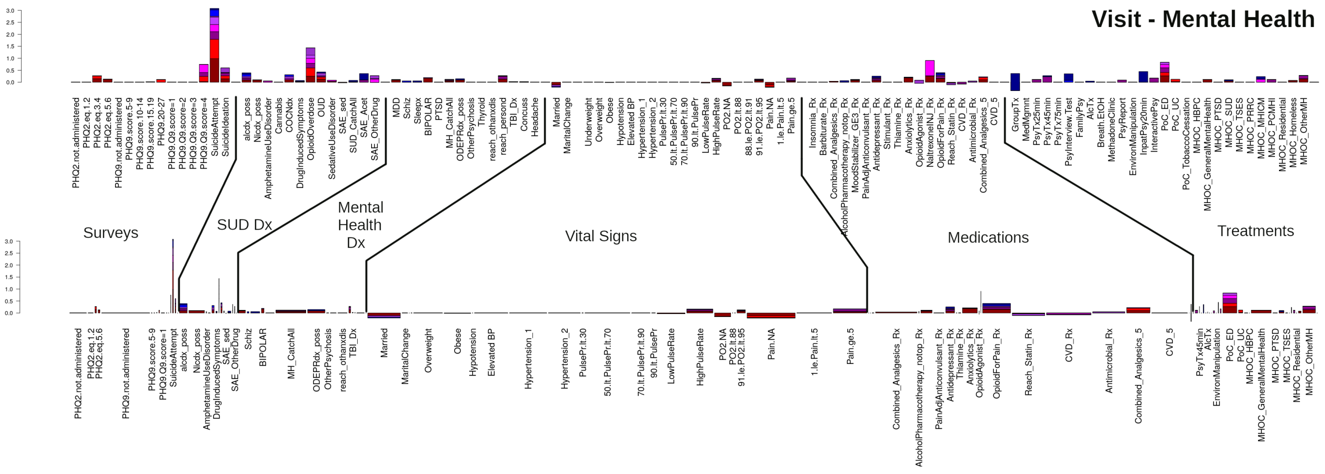

Figure S5. Model coefficients for Cox-Visit cohort related to mental health predictors.

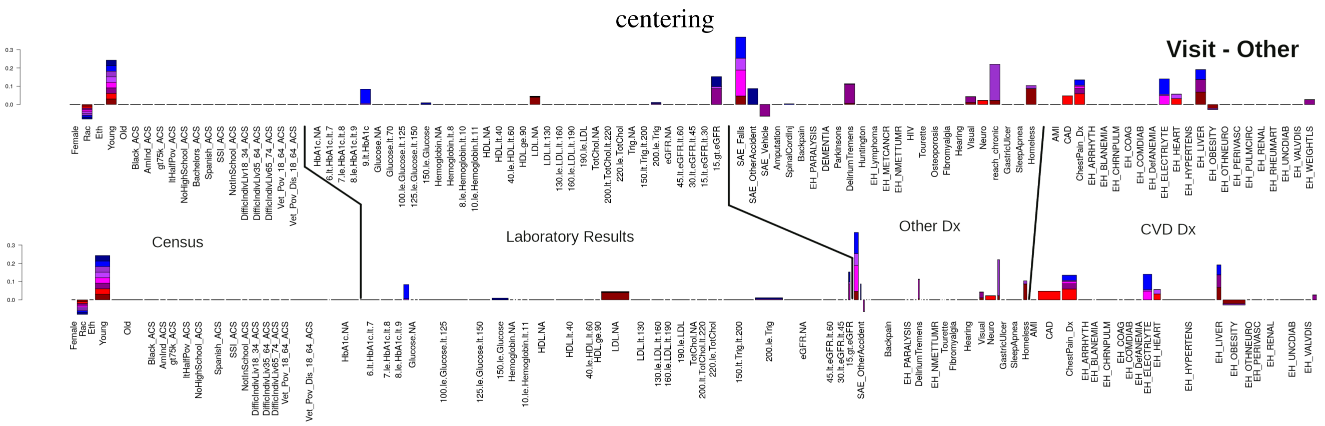

Figure S6. Model coefficients for Cox-Visit cohort related to non mental health related predictors

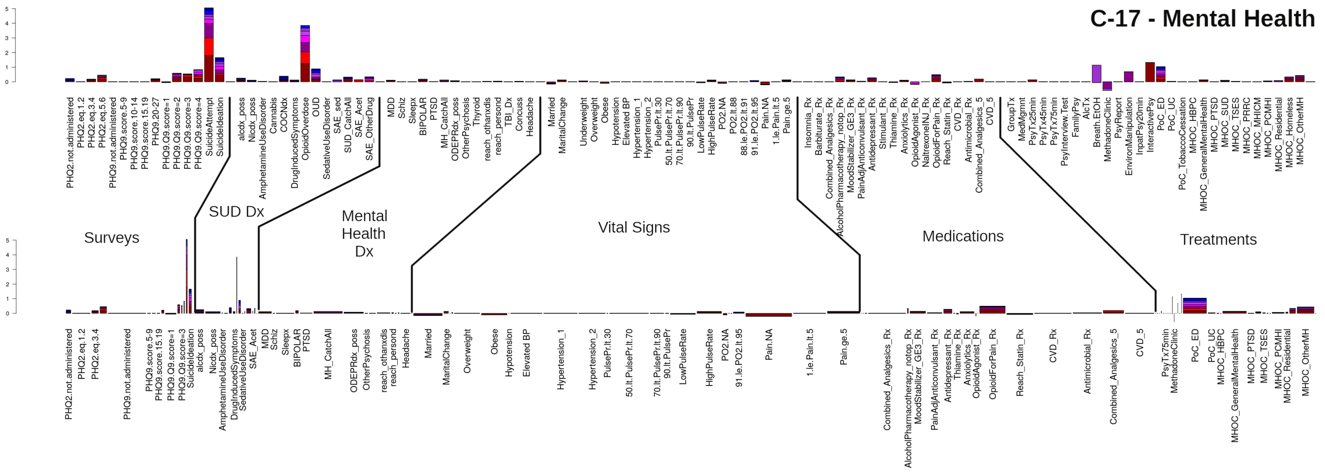

Figure S7. Model coefficients for C17 cohort related to mental health predictors.

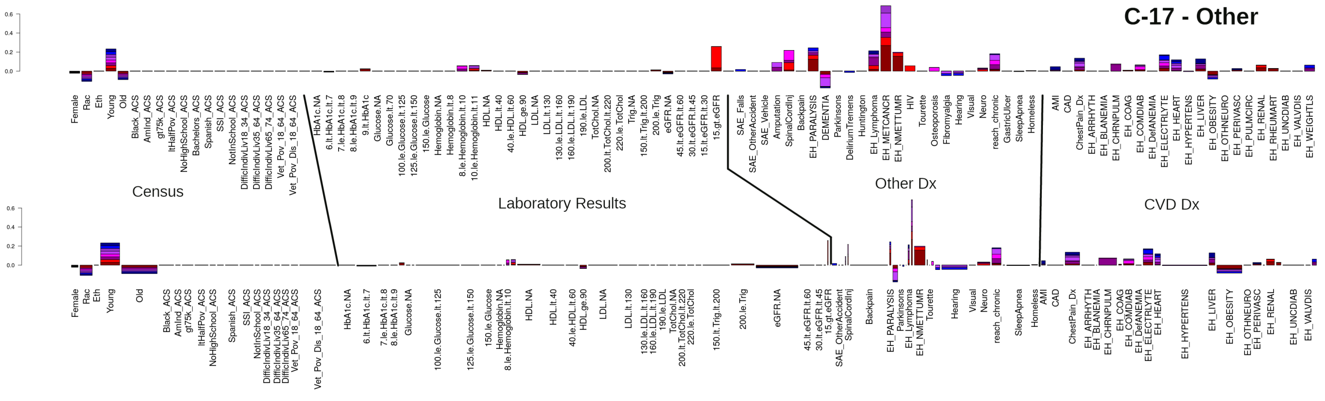

Figure S8. Model coefficients for C17 cohort related to non mental health related predictors

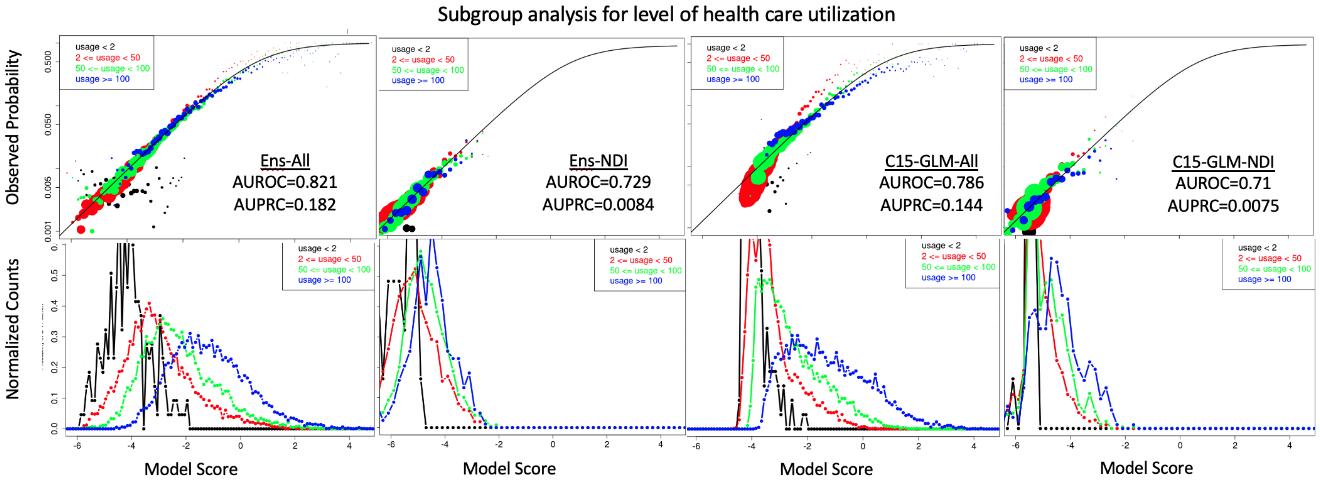

Figure S9. Calibration curves (top) and histograms of scores (bottom) for four subgroups of patients defined by the level of healthcare utilization. Healthcare utilization is quantified as the sum of all 76 Dx-coded variables present across all eight time bins (for a maximum possible value of 608), and color-coded as described in the figure legends. The two left sets of panels show curves for our ensemble models for combined outcome and suicide outcome. The two right sets of panels show curves for our C15 logistic regression model, fine-tuned and evaluated against the C17 cohort.

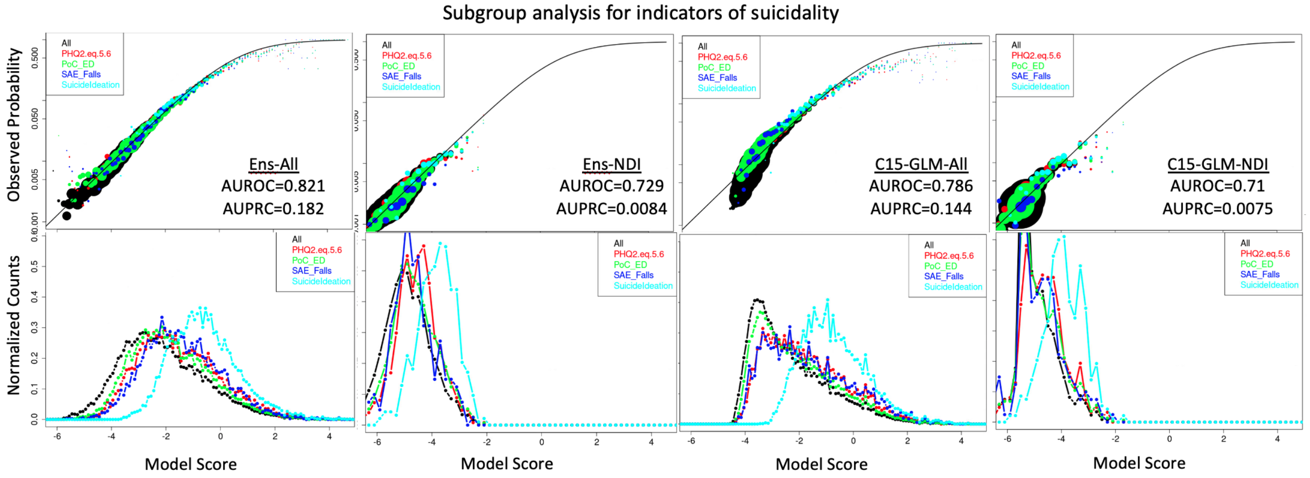

Figure S10. Calibration curves (top) and histograms of scores (bottom) for all patients (black) and four subgroups of patients are defined by the presence of selected variables observed to be strongly predictive of our combined outcome. Curves for patients with high PHQ2 scores are in red, patients visiting the Emergency Department are in green, patients receiving a diagnosis code indicating injury from a fall are in blue, and patients receiving a diagnosis code of suicidal ideation are in cyan. The two left sets of panels show curves for our ensemble models for combined outcome and suicide outcome. The two right sets of panels show curves for our C15 logistic regression model, fine-tuned and evaluated against the C17 cohort.

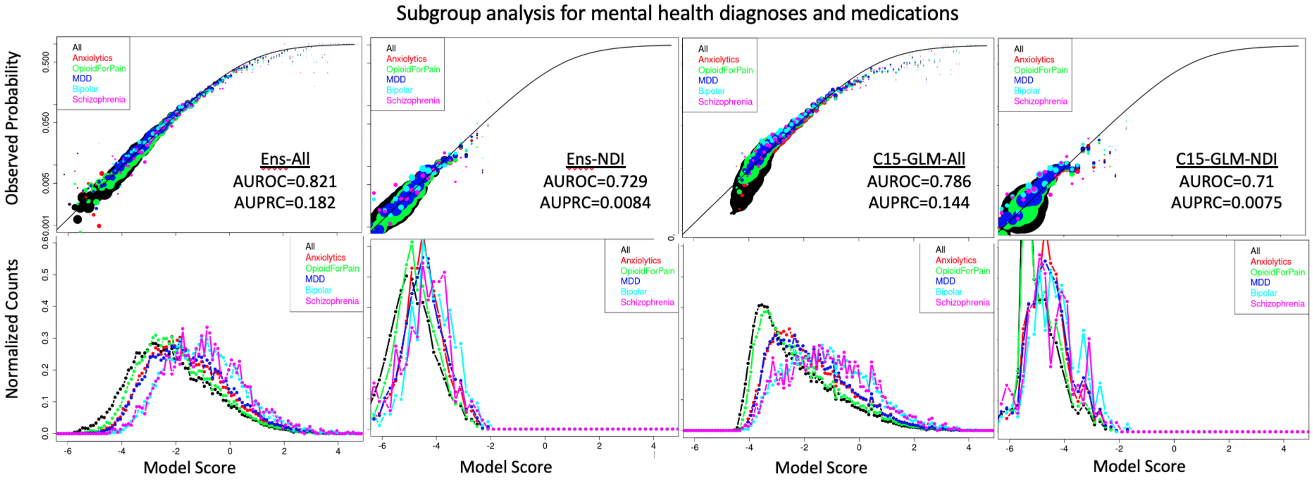

Figure S11. Calibration curves (top) and histograms of scores (bottom) for all patients (black) and five subgroups of patients, defined by the presence of mental health diagnoses and treatments. Curves for patients taking anxiolytic medications are in red and opioids in green. Patients diagnosed with major depressive disorder are in blue, bipolar disorder in cyan, and schizophrenia in magenta. The two left sets of panels show curves for our ensemble models for combined outcome and suicide outcome. The two right sets of panels show curves for our C15 logistic regression model, fine-tuned and evaluated against the C17 cohort.

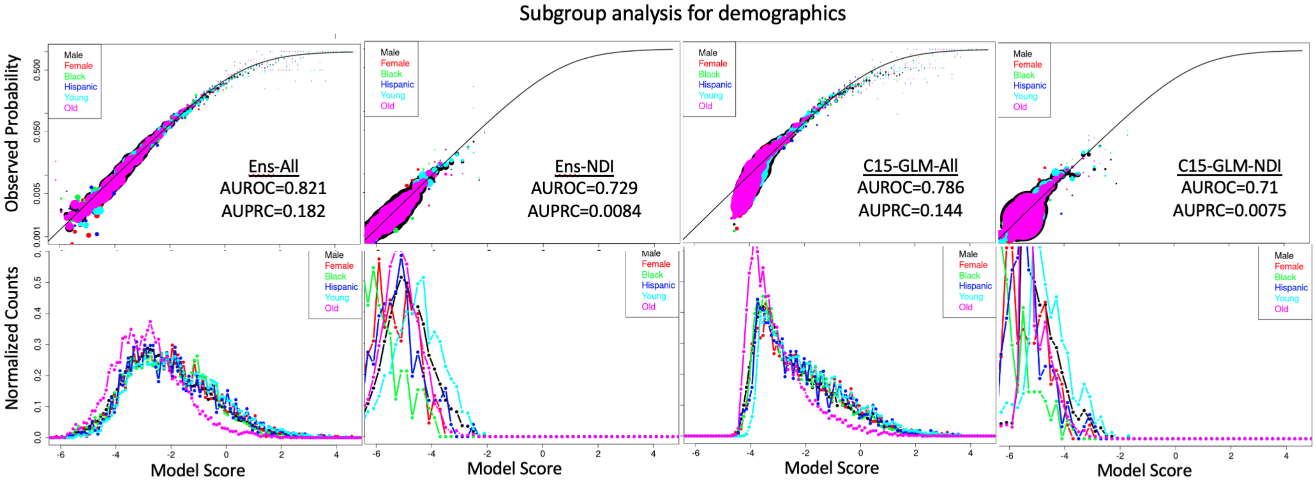

Figure S12. Calibration curves (top) and histograms of scores (bottom) for all male patients (black) and five subgroups of patients, defined by the gender (female) in red, race (African American) in green, ethnicity (Hispanic) in blue, age less than 50 years in cyan and greater than 65 in magenta. The two left sets of panels show curves for our ensemble models for combined outcome and suicide outcome. The two right sets of panels show curves for our C15 logistic regression model, fine-tuned and evaluated against the C17 cohort.

Training \ Evaluation	DoF	Rcc		C17	
		comb	NDI	comb	NDI
Percent cases		20.5%	2.9%	2.2%*	0.24%*
**C17 - NDI (LR)**	51				0.712
**Rcc - comb. (LR)**	607	0.816	0.659	0.798	0.669
**fine-tuned**		0.816	0.743	0.810	0.712
**Rcc - comb. (Neural Net)**		0.821	0.678	0.772	0.671 / 0.666
**fine-tuned**		0.823	0.760	0.795	0.714 / 0.704
**Rcc - comb. (RF)**		0.808	0.639	0.787	0.651 / 0.652
**fine-tuned**		0.814	0.756	0.803	0.716 /0.699
**Rcc - comb. (TabNet)**		0.827	0.681	0.791	0.678 / 0.671
**fine-tuned**		0.828	0.751	0.804	0.718 / 0.709
**Cox-MH - comb. (Cox)**	279	0.814	0.493	0.781	0.640
**fine-tuned**		0.814	0.706	0.784	0.685
**Cox-Visit - comb. (Cox)**	439	0.778	0.541	0.807	0.646
**fine-tuned**		0.791	0.711	0.810	0.702
**C17 - comb (LR)**	308	0.780	0.545	0.815	0.654
**fine-tuned**		0.793	0.712	0.815	0.702
**C17 - comb. (NNet)**		0.741	0.543 / 0.541	0.789	0.752 / 0.658
**fine-tuned**		0.779	0.711 / 0.710	0.792	0.784 / 0.695
**C17 - comb. (RF)**		0.788	0.556 / 0.552	0.807	0.733 / 0.657
**fine-tuned**		0.798	0.718 / 0.716	0.81	0.801 / 0.708
**C17 - comb. (TabNet)**		0.764	0.556 / 0.553	0.806	0.706 / 0.668
**fine-tuned**		0.785	0.714 / 0.712	0.808	0.748 / 0.709

Table S1. C-statistics for outcome prediction from models trained on the seven methodologies (rows) applied to predict outcomes on the Rcc and C17 cohorts. For each combination, the top score is AUROC for the model used directly on the evaluation set. The bottom number (fine-tuned) is obtained by re-fitting a logistic regression model with the score from the previous model along with demographics gender, race, ethnicity, and age, and all eight time bins for four variables: Pain ¿ 0, suicidal ideation, suicide attempt, and overdose in a logistic regression, together with original model scores. For the nonlinear models, log(score/(1-score)) was included in the fine-tuning rather than the model score directly. For models where differences between test and train accuracies, both are provided in the order train/test cohorts. Additionally, for the logistic regression and Cox models, the 'DoF' column provides the number of degrees of freedom that survived LASSO model selection. * For the C17 cohort, controls were randomly down-selected by a factor of four in each date-of-birth cohort. Numbers in this table reflect down selected groups, while numbers in Figure 1 reflect quantities before down selection.

	Rcc			Cox-MH		Cox-Visit		C17 (2-yr)		
	Ctrls	Cases (%)	NDI (%)	Ctrls	Cases (%)	Ctrls	Cases (%)	Ctrls	Cases (%)	NDI (%)
**Total (%)**	1,297,062	333,712 (25.7)	46,944(2.88)	1,137,724	80,771(7.10)	5,440,660	148,745(2.7)	4,193,943	24,446(0.5)	2,812(0.067)
**SubCohort**						1,306,165	148,745(10.9)	1,048,486	24,446(2.3)	2,812(0.27)
**Female (%)**	226,627	37,701 (16.6)	1,503(0.57)	105,350	9,941(9.44)	310,540	13,367 (4.3)	402,668	5,193(1.3)	114(0.028)
**Black (%)**	199,639	60,211 (30.15)	2,133 (0.82)	209,437	16,863(8.05)	790,524	29,109(3.7)	740,652	9,851(1.3)	166(0.022)
**Hispanic (%)**	75,814	20,862 (27.5)	1,539(1.59)	65,286	4,483(6.87)	257,632	8,102(3.1)	261,580	3,384(1.3)	129(0.049)
**65 and older**	360,272	91,353 (25.4)	21,140(4.68)	214,501	5,699(2.65)	2,263,748	26,565(1.2)	2,209,820	15,774(0.71)	1,336(0.60)
**under 55**	606,288	158,574 (26 .2)	16,205(2.12)	506,529	51,368(10.1)	1,481,912	76,422(4.9)	1,146,620	18,889(1.6)	998(0.087)

Table S2. Characteristics of the study population. Study size and number of cases are provided for retrospective case-control (Rcc), time-to-event (Cox) models for Cox-MH and Cox-Visit study designs, and interval predictions for C17. Number of cases and controls are provided for all four studies, and the subset of Cases with an NDI-reported suicide is provided for Rcc and Calendar-2017. Cox-Visit and C17 were both nested cohort studies, downsampling controls by a factor of four, indiciated by the SubCohort row.

Variable	Coef.C17	Coef.MH	Coef.Rcc	Adj.OR	Cases	Controls	% Cases	%Cases/%Ctrls	EnrS	EnrSA	EnrOD
Total	-	-	-	-	260,579	961,423	21.3	1.000	2.56%	11.2%	7.6%
Female	0.0	0.0	-0.3	0.7	25130	151172	14.3	0.7	0.3	0.7	1.0
Rac	-0.1	-0.2	-0.1	0.9	40190	133355	23.2	1.2	0.4	1.0	1.9
Eth	0.0	0.0	0.0	1.0	13880	50630	21.5	1.1	0.8	1.1	1.4
Young	0.2	0.3	0.4	1.5	105855	404629	20.7	1.0	1.0	1.1	1.1
Old	-0.1	-0.1	0.2	1.2	61030	240695	20.2	1.0	2.2	0.4	1.7
Married	-0.2	-0.2	-0.2	0.8	92530	337922	21.5	1.1	1.5	0.8	1.6
MaritalChange	0.1	0.0	0.8	2.1	25205	46514	35.1	1.8	2.2	1.7	2.6
Glucose.NA	0.0	0.0	-0.4	0.7	25958	324274	7.4	0.3	0.6	0.3	0.3
Pain.NA	-0.2	0.0	-0.4	0.7	195335	844107	18.8	0.3	1.3	0.8	1.2
Glucose.NA	0.0	0.0	-0.4	0.7	25958	324274	7.4	0.3	0.6	0.3	0.3
Hypertension_2	0.0	0.0	0.0	1.0	101111	264431	27.7	1.6	2.0	1.0	2.2
90.lt.PulsePr	0.0	0.0	0.3	1.3	12908	23619	35.3	1.8	2.6	1.2	3.2
88.le.PO2.lt.91	0.0	0.0	0.1	1.1	2913	5087	36.4	1.8	3.4	1.0	3.4
Pain.ge.5	0.1	0.2	0.2	1.3	162581	329444	33.0	3.3	1.9	1.4	2.6
PHQ2.not.admin.	0.2	0.3	0.7	2.1	67185	400513	14.4	0.6	1.0	0.6	0.9
PHQ2.eq.1.2	0.0	0.0	0.3	1.4	63998	141338	31.2	1.7	2.0	1.4	2.3
PHQ2.eq.3.4	0.2	0.0	1.0	2.7	29812	51846	36.5	1.9	2.2	2.0	2.5
PHQ2.eq.5.6	0.5	0.0	1.7	5.5	38314	52022	42.4	2.3	2.8	2.5	2.8
PHQ9.Q9.score=1	0.0	0.0	-0.8	0.5	45039	103652	30.3	1.6	1.6	1.5	2.1
PHQ9.Q9.score=2	0.6	0.1	0.5	1.6	14856	18942	44.0	2.2	2.5	2.9	2.4
PHQ9.Q9.score=3	0.5	0.0	0.6	1.7	6028	6283	49.0	2.4	2.6	3.5	2.5
PHQ9.Q9.score=4	0.8	0.2	1.5	4.4	6060	4671	56.5	2.8	4.2	4.2	3.0
SuicideAttempt	5.1	3.2	0.2	1.3	4990	3759	57.0	2.8	11.0	2.8	4.2
SuicideIdeation	1.7	0.9	3.2	24.5	37101	15360	70.7	3.9	6.6	5.6	4.6
OpioidOverdose	3.8	1.1	1.8	6.1	2019	430	82.4	4.1	16.2	6.7	6.5
OUD	0.9	0.3	0.8	2.3	26429	12886	67.2	3.6	4.6	4.4	6.4
SAE_sed	0.1	0.1	0.4	1.5	2956	996	74.8	3.7	11.4	5.4	6.7
SAE_Acet	0.1	0.1	0.7	2.0	2526	950	72.7	3.6	8.4	4.9	7.1
MDD	0.1	0.2	-0.1	0.9	83102	118888	41.1	2.6	2.4	2.5	2.7
Schiz	0.0	0.0	0.1	1.1	15936	11649	57.8	3.0	4.1	3.9	4.2
BIPOLAR	0.2	0.2	0.4	1.5	31862	22247	58.9	3.2	4.9	4.1	4.0
PTSD	0.0	0.0	0.0	1.0	74863	117413	38.9	2.4	2.0	2.2	2.6
MH_CatchAll	0.1	0.0	0.5	1.6	156430	266865	37.0	3.7	2.4	1.8	2.8
reach_othanxdis	0.0	0.0	0.0	1.0	81354	127671	38.9	2.4	2.4	2.1	2.7
reach_persond	0.0	0.4	0.4	1.5	24819	12889	65.8	3.5	5.1	4.8	4.9
TBI_Dx	0.0	0.0	0.2	1.3	7735	8470	47.7	2.4	2.6	2.7	3.8
SAE_Falls	0.0	0.2	1.0	2.6	25595	24440	51.2	2.7	2.5	2.3	5.1
reach_othanxdis	0.0	0.0	0.0	1.0	81354	127671	38.9	2.4	2.4	2.1	2.7
reach_persond	0.0	0.4	0.4	1.5	24819	12889	65.8	3.5	5.1	4.8	4.9
Homeless	0.0	0.1	0.3	1.3	44682	41280	52.0	2.9	2.3	3.3	3.9
CAD	0.0	0.0	0.2	1.2	45264	94012	32.5	1.7	2.4	1.0	3.0
Insomnia_Rx	0.0	0.0	0.0	1.0	596	420	58.7	2.9	5.2	4.0	4.1
MoodStab_GE3_Rx	0.1	0.1	0.1	1.1	91761	126386	42.1	2.8	2.5	2.2	3.3
Antidepressant_Rx	0.3	0.2	0.3	1.4	78766	69849	53.0	3.5	3.6	3.2	4.2
Stimulant_Rx	0.0	0.0	-0.2	0.8	7129	10910	39.5	2.0	2.6	2.3	2.5
Thiamine_Rx	-0.1	0.0	-0.1	1.0	22947	16310	58.5	3.1	4.0	3.8	4.7
Anxiolytics_Rx	0.1	0.2	0.4	1.5	95430	118472	44.6	3.1	3.3	2.3	3.5
OpioidForPain_Rx	0.5	0.3	0.6	1.9	135784	219959	38.2	3.2	2.3	1.6	3.3
Reach_Statin_Rx	-0.1	-0.1	-0.2	0.8	90069	231230	28.0	1.6	2.0	1.0	2.3
PoC_ED	1.0	0.5	1.1	2.9	132770	208882	38.9	3.2	2.0	1.8	3.3
MHOC_SUD	0.1	0.0	0.4	1.5	60229	48827	55.2	3.3	3.4	3.5	4.3
MHOC_Homeless	0.3	0.0	0.0	1.0	50492	57024	47.0	2.7	2.1	2.9	3.4
MHOC_OtherMH	0.4	0.0	0.4	1.5	115530	167522	40.8	3.1	2.4	2.3	2.9

Table S3. Attributes of predictor variables associated with combined outcome for Rcc study design. Selected coefficients are shown in the first column, for which the following information is provided. Model selected logistic regression coefficients are provided for the C17, Cox-MH, and Rcc Cohorts, together with the adjusted odds ratio for Rcc ($exp(Coef.Rcc)$). Next are provided the number of cases and controls with each attribute, and the percentage of patients with each attribute that are cases. Note that the total number of casess and controls are provided for reference in the top row. This is followed by the unadjusted hazard ratio (%cases/%controls for each variable) for Rcc and the relative risks for each component of our combined outcome, also for Rcc cohort.

References

1. Arroll, B. *et al.* Validation of PHQ-2 and PHQ-9 to screen for major depression in the primary care population. *Ann. Fam. Med.* **8**, 348–353 (2010).
2. Simon, G. E. *et al.* Predicting suicide attempts and suicide deaths following outpatient visits using electronic health records. *Am. J. Psychiatry* **175** (2018).
3. Cox, E. *et al.* Good research practices for comparative effectiveness research: Approaches to mitigate bias and confounding in the design of nonrandomized studies of treatment effects using secondary data sources: The International Society for Pharmacoeconomics and Outcomes Research good research practices for retrospective database analysis task force report - Part [II]. *Value Heal.* **12**, 1053–1061 (2009).
4. van den Hondel, K. E., Punt, P., Dorn, T., Ceelen, M. & Reijnders, U. The rise of suicides using a deadly dose of barbiturates in Amsterdam and Rotterdam, the Netherlands, between 2006 and 2017. *J Forensic Leg Med.* **70**, 101916, DOI: 10.1016/j.jflm.2020.101916. (2020).
5. Goodman, W. K., Murphy, T. K. & Storch, E. A. Risk of adverse behavioral effects with pediatric use of antidepressants. *Psychopharmacol. (Berl).* **191**, 87–96, DOI: 10.1007/s00213-006-0642-6. (2007).
6. Dodds, T. J. Prescribed Benzodiazepines and Suicide Risk: A Review of the Literature. *Prim Care Companion CNS Disord.* **19**, DOI: 10.4088/PCC.16r02037. (2017).
7. Chen, Q. *et al.* Drug treatment for attention-deficit/hyperactivity disorder and suicidal behaviour: Register based study. *Biol Psychiatry.* **348**, g3769, DOI: 10.1136/bmj.g3769. (2014).
8. Wilcox, H. C., Conner, K. R. & Caine, E. D. Association of alcohol and drug use disorders and completed suicide: an empirical review of cohort studies. *Drug Alcohol Depend.* **76**, S11–9, DOI: 10.1016/j.drugalcdep.2004.08.003. (2004).
9. Mee, S., Bunney, B. G., Reist, C., Potkin, S. G. & Bunney, W. E. Psychological pain: A review of evidence. *J Psychiatr Res.* **40**, 680–90, DOI: 10.1016/j.jpsychires.2006.03.003. (2006).
10. Miller, T. R. *et al.* Incidence and lethality of suicidal overdoses by drug class. *JAMA Netw Open.* **3**, e200607, DOI: 10.1001/jamanetworkopen.2020.0607. (2020).
11. Adolphs, R. Neural systems for recognizing emotion. *Curr Opin Neurobiol.* **12**, 169–77, DOI: 10.1016/s0959-4388(02)00301-x. (2002).
12. Roberts, A. W., Eiffert, S., Wulff-Burchfield, E. M., Dusetzina, S. B. & Check, D. K. Opioid use disorder and overdose in older adults with breast, colorectal, or prostate cancer. *J. Natl. Cancer Inst.* **113**, 425–433 (2021).
13. Madsen, T. *et al.* Association Between Traumatic Brain Injury and Risk of Suicide. *JAMA.* **320**, 580–588, DOI: 10.1001/jama.2018.10211. (2018).
14. Lemogne, C. *et al.* Heart rate and completed suicide: evidence from the ipc cohort study. *Psychosom. Med.* **73**, 731–736 (2011).
15. Riblet, N. B. *et al.* Hypoxia-related risk factors for death by suicide in a national clinical sample. *Psychiatry Res.* **273**, 247–251, DOI: 10.1016/j.psychres.2019.01.040. (2019).
16. Wang, X. *et al.* An examination of the association between altitude and suicide deaths, suicide attempts, and suicidal ideation among veterans at both the patient and geospatial level. *J. psychiatric research* **153**, 276–283 (2022).
17. Rozanski, A., Blumenthal, J. A. & Kaplan, J. Impact of psychological factors on the pathogenesis of cardiovascular disease and implications for therapy. *Circulation.* **99**, 2192–217, DOI: 10.1161/01.cir.99.16.2192. (1999).
18. Artero, S., Astruc, B., Courtet, P. & Ritchie, K. Life-time history of suicide attempts and coronary artery disease in a community-dwelling elderly population. *Int J Geriatr Psychiatry.* **21**, 108–12, DOI: 10.1002/gps.1429. (2006).
19. Webb, W. L. J. & Gehi, M. Electrolyte and fluid imbalance: neuropsychiatric manifestations. *Psychosomatics.* **22**, 199–203, DOI: 10.1016/S0033-3182(81)73532-1. (1981).
20. US Census Bureau. American Community Survey (2022).
21. Leavitt, R. A. *et al.* Suicides among American Indian/Alaska natives — National Violent Death Reporting System, 18 states, 2003–2014. *Morb. Mortal. Wkly. Rep.* **67**, 237–242 (2018).
22. C, O.-O. Marital status, educational attainment, and suicide risk: a Norwegian register-based population study. *Popul. Heal. Metr.* **19**, 33 (2021).

23. Goldney, R. D., Dunn, K. I., Air, T. M., Dal Grande, E. & Taylor, A. W. Relationships between body mass index, mental health, and suicidal ideation: population perspective using two methods. *Aust N Z J Psychiatry.* **43**, 652–8, DOI: 10.1080/00048670902970825. (2009).

24. Graham, C. & Frisco, M. The relationship between obesity and suicide ideation among young adults in the United States. *SSM Popul Heal.* **18**, 101106, DOI: 10.1016/j.ssmph.2022.101106. (2022).
